# Palladium-Catalyzed Monoarylation of Cyclopropylamine

**DOI:** 10.1021/acs.joc.5c01233

**Published:** 2025-08-20

**Authors:** Nicolas Kaiser, Lea Räkow, Jens Handelmann, Viktoria H. Gessner

**Affiliations:** Faculty of Chemistry and Biochemistry, Ruhr-University Bochum, Universitätsstraße 150, 44801 Bochum, Germany

## Abstract

A novel palladium-catalyzed protocol for the monoarylation of cyclopropylamine using the sterically demanding and electron-rich adamantyl-substituted ylide-functionalized phosphine (YPhos) adYPhos is reported. This method enables the efficient coupling of a wide range of (hetero)­aryl chlorides at room temperature. The reported ligand enhances the scope for the application of YPhos ligands in amination reactions on more challenging substrates. The protocol is versatile, accommodating various functional groups and extending to larger cycloalkylamines, all accessible under feasible and mild conditions.

## Graphical abstract

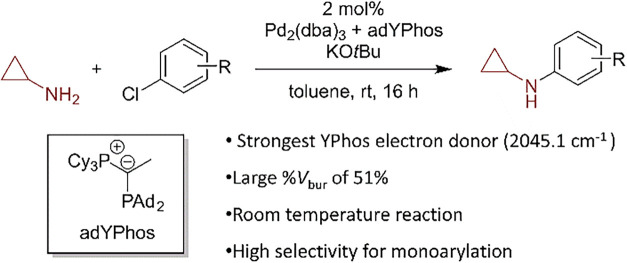

## Introduction

Palladium-catalyzed coupling reactions have become essential in both academic and industrial settings, undergoing rapid development and improvement in the past decades.^1–3^ Among them, amination reactions—pioneered by Buchwald^4^ and Hartwig^5^—have become indispensable tools in modern synthetic chemistry, particularly in the synthesis of agrochemicals, natural products, and pharmaceuticals.^6–8^ Significant advancements in this field have been driven largely by the development of increasingly sophisticated ligands. In particular, sterically demanding and electron-donating phosphines,^9–12^ as well as *N*-heterocyclic carbenes,^13–15^ have helped overcome limitations of early protocols, enabling milder conditions or the efficient conversion of challenging substrates.

Cyclopropylamines are, in particular, interesting substrates due to their role as key building blocks in pharmaceuticals^16^ like the antibiotics fluoroquinolone^17^ or reverse transcriptase inhibitors^18^ and in insecticides.^19^ Furthermore, they are used as spin traps in mechanistic studies of biological^20^ and chemical^21^ systems and as versatile precursors to reactive intermediates through ring-opening,^22,23^ which subsequently can undergo cycloaddition reactions to form valuable heterocyclic compounds.^24–28^

Despite these versatile applications, the synthesis of arylated cyclopropylamines remains a challenging and underdeveloped area. Aniline derivatives generally exhibit low reactivity with cyclopropyl halides,^29,30^ while the reductive amination of activated cyclopropyl electrophiles bearing alkoxy groups typically requires an impractical two-step procedure.^31^ Although this process can be streamlined into a one-step protocol using an excess of silylketene acetal reagents (Figure 1), it is often accompanied by undesirable overalkylation.^32,33^ The Smiles rearrangement represents another indirect approach to accessing cyclopropylamines but requires elevated temperatures of 110 °C.^34^ Similarly, Chan–Lam-type couplings can provide access to these compounds, yet their practicality is limited by the scarce availability of cyclopropyl-substituted boronic acids and the high costs of stoichiometric amounts of copper reagents.^35^ Despite extensive research in transition-metal catalyzed amination reactions, only a few examples exist that are specifically suited for the arylation of cyclopropylamine. Prior to 2016, Pd-catalyzed transformations were limited to aryl bromides, and only one example of a heteroaryl halide using the BINAP ligand was reported.^36^ *Colacot* broadened the reaction scope by facilitating the coupling of aryl bromides at room temperature and aryl chlorides at elevated temperatures.^37^ However, this method relies on the costly cationic precatalyst **A** ^38^ with the Buchwald-type ligand *t*BuBrettPhos^39^ (Figure 1B). More recently, *Stradiotto’s* group developed a nickel-catalyzed approach that enables the coupling of (hetero)­aryl chlorides at room temperature, using the CyPAd-Dalphos ligand **B**.^40^ While this represents an important advancement, the synthesis of the ligand involves a Pd-catalyzed coupling to introduce the caged phosphine, undermining the cost and sustainability advantages typically associated with first row transition-metal catalysts.^41^ Additionally, *Wan* reported a room-temperature method for the amination of aryl halides employing the CuI/*N*-carbazolyl-1*H*-pyrrole-2-carbohydrazide catalytic system **C**, though this approach is limited to aryl bromides.^42^

**Figure 1:**
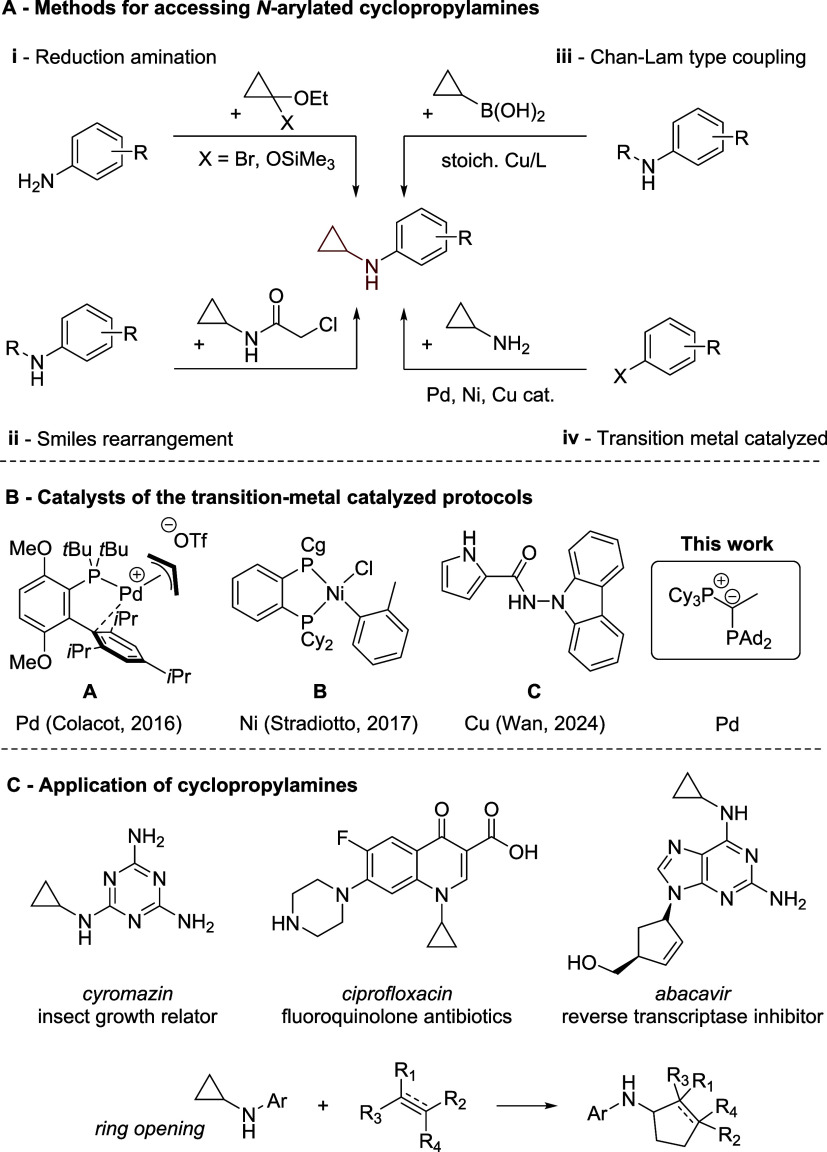
Top: classical preparation methods of *N*-arylated cyclopropylamines (i–iii) and Pd-, Cu-, and Ni-catalyzed cross-coupling procedures (iv). Bottom: overview of all effective ligands and precatalysts for the monoarylation of cyclopropylamine via the transition-metal-catalyzed version.

Recently, our group introduced the class of ylide-functionalized phosphines (YPhos),^11^ which can easily be prepared by stepwise substitution reactions from convenient starting materials in a cost-effective fashion compared to more sophisticated ligand systems requiring transition-metal-catalyzed coupling steps. YPhos ligands are highly electron-rich and enabled the C–N coupling of a broad range of substrates,^43^ including alkyl and aryl amines,^44^ small primary amines,^45^ ammonia,^46^ and hindered di- or triarylamines^47^ with aryl chlorides at mild conditions. However, despite these developments, the unique case of cyclopropylamine formation has remained inaccessible via YPhos-Pd catalysis to date.^48^

The mechanistic cycle for the amination using cyclopropylamine is assumed to proceed through the same mechanistic steps as for other C–N coupling reactions (Figure 2). The first step, the oxidative addition of the aryl halide, is typically facilitated by electron-donating ligands, while reductive elimination is faster from electron-poor and sterically encumbered complexes.^49^ Given the constrained and compact nature of the cyclopropylamine substrate, all ligands employed were relatively sterically demanding, likely promoting the otherwise challenging reductive elimination step involving small substrates.^50–52^

**Figure 2:**
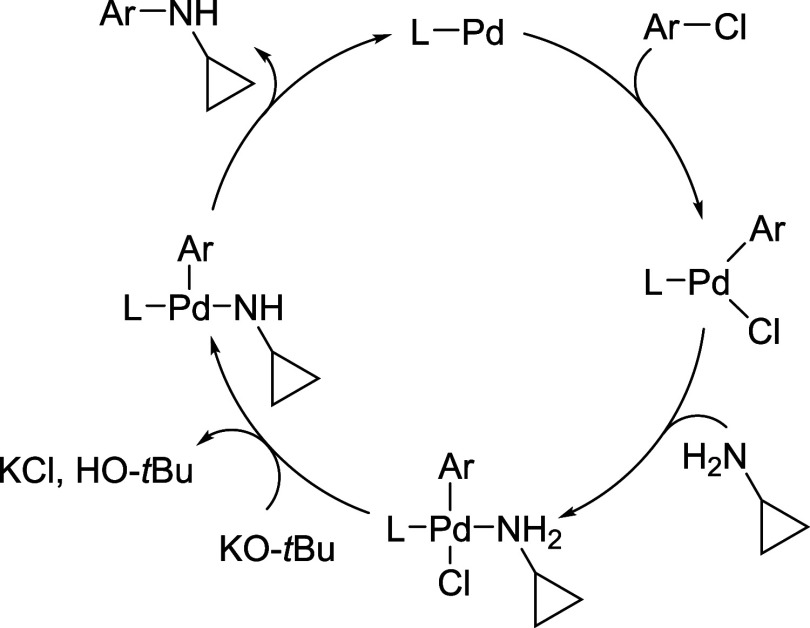
Proposed catalytic cycle for the monoarylation of ammonia using a Pd-phosphine catalyst.

In this work, we present a protocol for the arylation of cyclopropylamines, establishing the first Pd-catalyzed room-temperature reaction of aryl chlorides that eliminates the need for a preformed precatalyst and accommodates the substrate scope. This method not only enhances efficiency across a wide range of aryl chlorides but also applies to cycloalkylamines of varying ring sizes, thereby expanding its utility across a broader range of synthetic transformations. The key to this improved protocol lies in the favorable properties of the sterically encumbered adYPhos ligand, which plays a crucial role in achieving high reactivity and selectivity.

## Results and Discussion

### Reaction Optimization

Previous studies identified electron-rich aryl chlorides, such as the deactivated 4-chloroanisole, as challenging electrophiles in the Pd-catalyzed coupling of cyclopropylamines.^37^ Therefore, we selected this aryl chloride as a model substrate for optimization of the reaction conditions. Due to the excellent performance of YPhos-Pd catalysts in many coupling reactions at room temperature, we examined the coupling of cyclopropylamine at this temperature. Since the respective coupling product is prone to decomposition through reaction with ambient oxygen, the consumption of aryl chloride was used to monitor reaction success. Competing reaction pathways were ruled out as no side-products were detected in the measured GC-FID spectra. The complete screening tables can be found in the Supporting Information. In Table 1, only the best results are highlighted.

**Table 1:**
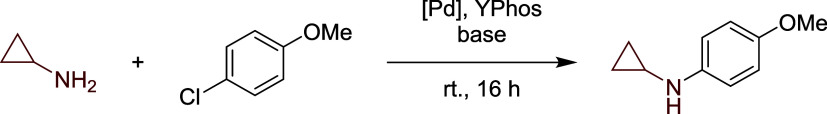

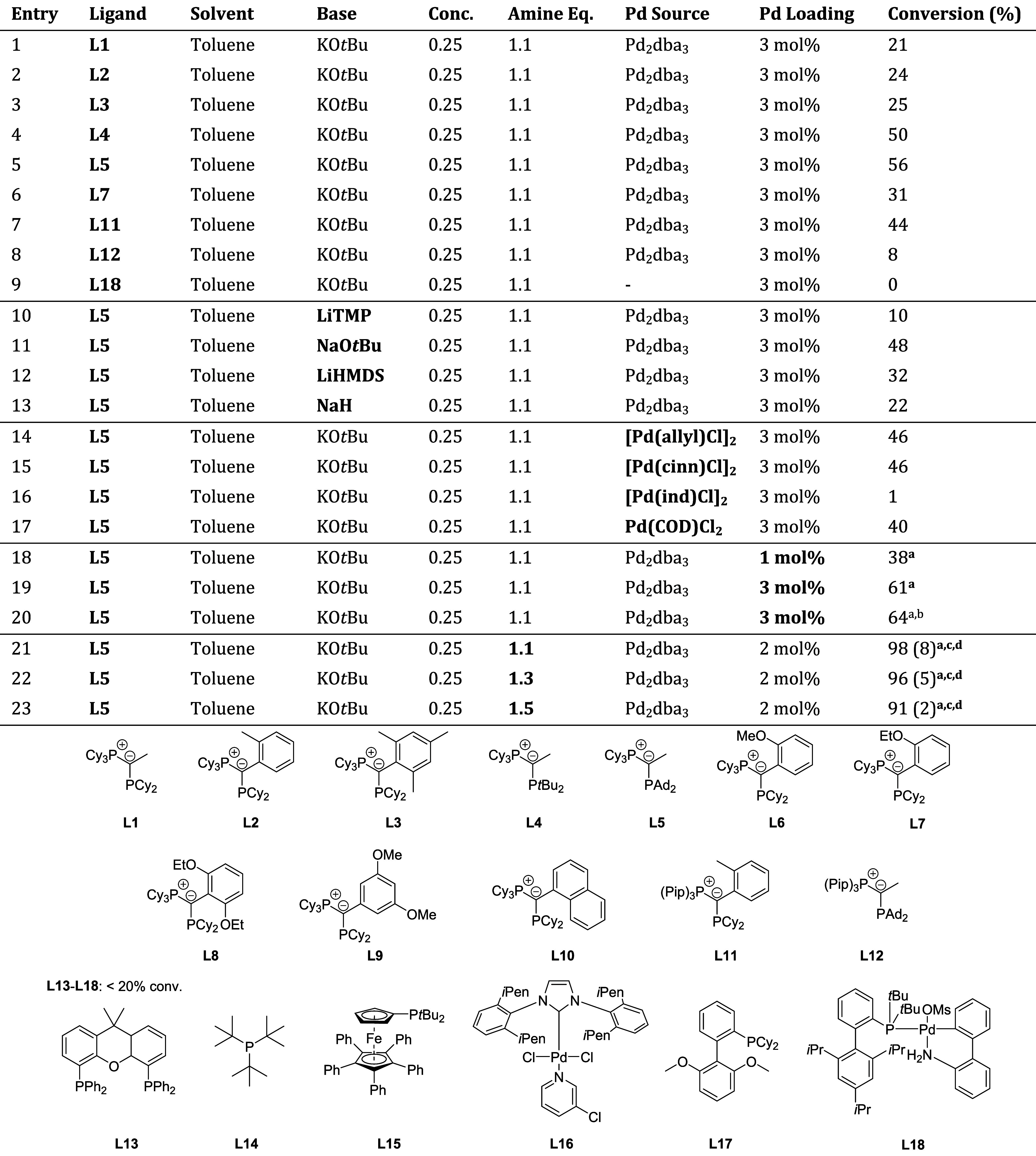
Optimization of the Reaction Conditions for the Arylation of Cyclopropylamine with 4-Chloroanisole^e^

a Longer preformation time of 6 h.

b At 60 °C.

c 4-Chlorotoluene used as an electrophile.

d Diarylated product in brackets.

e Reaction conditions: 30 min preformation time for [Pd]·L, 0.25 mmol ArCl, rt, 16 h. Conversion determined by calibrated GC analysis using tetradecane as internal standard.

The initial ligand screening with Pd_2_dba_3_ as the palladium source revealed that the YPhos ligands provided significant activity (>20%), while other well-established phosphines like XantPhos,^53^ P*t*Bu_3_,^54^ QPhos,^55^ and Buchwald-type ligands, including the *t*BuBrettPhos precatalyst^56^ **L18**, or NHC derivatives like the PEPPSI-IPent precatalyst^57^ gave no or only minor conversion. The best results were obtained for the sterically demanding trYPhos **L4** and adYPhos ligand **L5**, which achieved more than 50% conversion. The bulkier adamantyl-substituted ligand **L5** performed slightly better and was therefore used for further optimization studies. The screening of a series of different solvents and bases identified toluene and potassium *tert*-butoxide as the optimal solvent and base combination. This finding is surprising as other Buchwald–Hartwig amination reactions catalyzed by Pd-YPhos systems have typically performed best in THF.^44,45,47^ A slight excess of base and amine, as well as a high concentration of the reaction mixture, turned out to be advantageous, while a large excess of cyclopropylamine and the use of other allyl- ^58,59^ or dichloropalladium^60,61^ precatalysts had adverse effects on the conversion (entries 14–17). However, a longer preformation time of the LPd(0) species of 6 h enabled higher conversions of 61% (entry 19), which can presumably be attributed to a significantly slower preformation of the adYPhos Pd catalyst in less polar solvents (cf. < 30 min in THF, see Supporting Information). Attempts to isolate Pd complexes from adYPhos and Pd­(COD)­Cl_2_ ^62^ (COD = cyclooctadiene) or [Pd­(ind)­Cl]_2_ ^63–65^ (ind = 1-*tert*-butylindenyl) were unsuccessful due to their decomposition to several products.

Since no full conversion of the challenging substrate 4-chloroanisole was achieved, we also tested 4-chlorotoluene, a sterically similar but electronically less deactivated substrate. Indeed, this aryl chloride was fully consumed and led to only minor amounts of the diarylated product, which could be further suppressed by using a slight excess of amine (1.3 equiv). Under these conditions, the catalyst loading could be decreased to 2 mol %, while maintaining a high conversion of 91%. To analyze the steric effects, we examined the impact of *ortho*-substitution in aryl chlorides using five representative ligands. In line with trends observed for para-substituted substrates, ligand **L5** provided a respectable 70% yield with 2-chlorotoluene, while ligands **L2**, **L3**, **L4**, and **L7** all afforded yields below 20%. However, a markedly different outcome was observed with 2-chloro-1,3-dimethylbenzene. In this case, neither **L4** nor **L5**, the previously most effective ligand, produced any detectable product. Notably, the highest yield in this case was a moderate 47% with **L2**, indicating that the challenging reductive elimination is facilitated for *ortho*-hindered substrates and is favored by the comparatively smaller ligands. Additionally, substantial formation of the dehalogenated side product was observed with all ligands (see Supporting Information for details).

### Reaction Scope

To investigate the applicability and tolerance of our protocol toward different functional groups, we reacted various (hetero)­aryl chlorides with cyclopropylamine under our optimized reaction conditions. A wide range of functional groups (Scheme 1), including ether (**1** and **2**), cyano (**3**), ester (**7**), and ketone groups (**8**), as well as halides (**9** and **10**), are tolerated, giving the coupling products in moderate to high yields between 50 and 97%. Besides aryl chlorides, aryl bromides and iodides can also be coupled under the applied conditions (**1** and **10**), and it was possible to upscale the reaction up to 10 mmol with a slight decrease of the yield to 70%. The fluoro- and trifluoromethyl-substituted products (**4** and **5**) were obtained in poor yields because no complete conversion was reached, as previously observed in our group for other Buchwald–Hartwig amination reactions.^45^ However, other electron-poor aryl chlorides provided moderate to good isolated yields (**1**–**3** and **6**–**8**) although slightly elevated temperatures of 60 °C were needed for amide **6**. The use of polyhalogenated arenes led to the preferential transformation of the more reactive bromide compared to chloride (**9**) and the monoamination of the dichloro-substituted compound **10**. Even when higher temperatures and an excess of amine were employed, the formation of the diamination product remained minimal. Also, introducing steric hindrance by *ortho-*substitution of the substrate could diminish the yield significantly (**11**) although smaller substituents are still comparably tolerated (**12** and **13**).

**Scheme 1:**
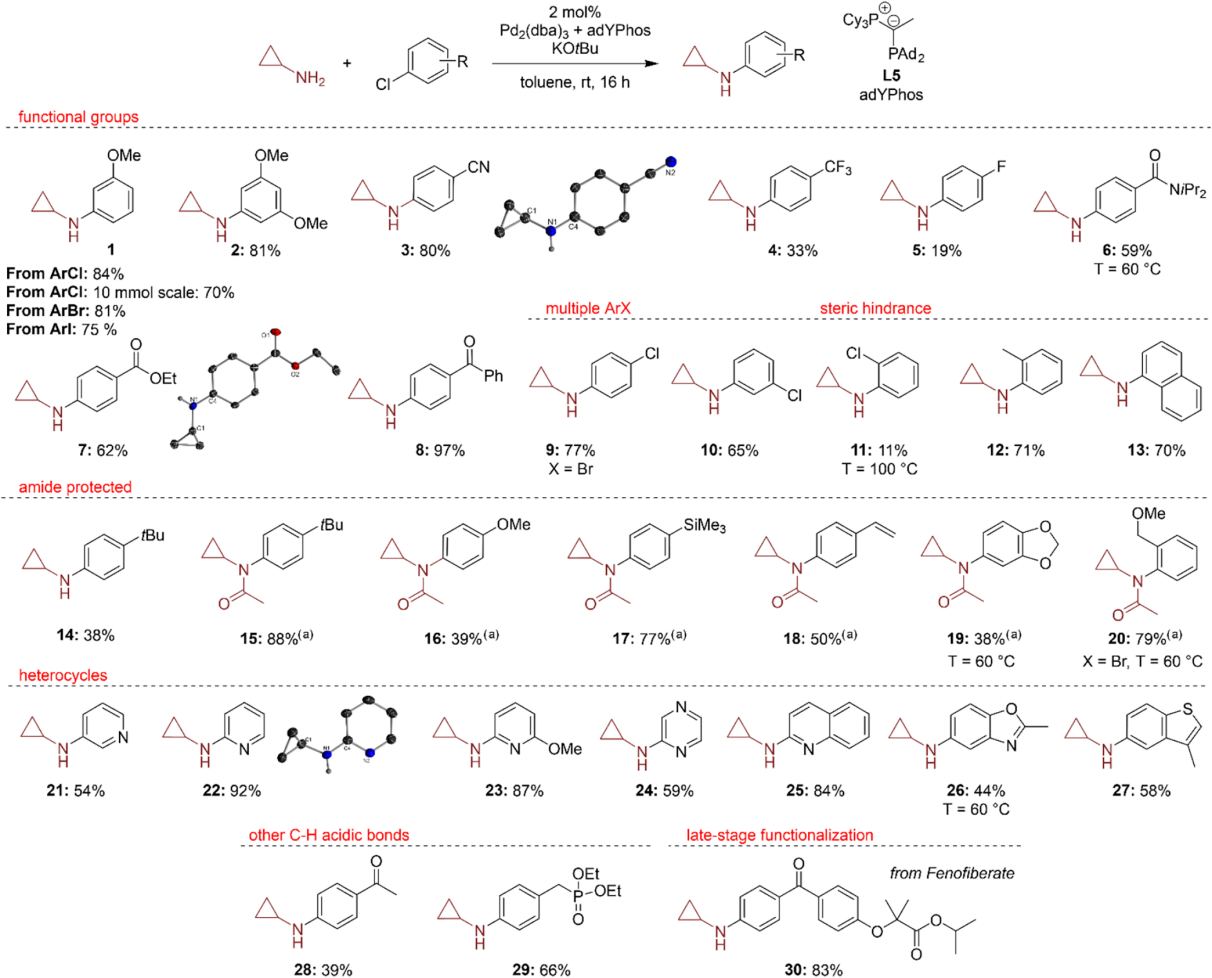
Scope for the Arylation of Cyclopropylamine^a^

a Reaction conditions: 1.0 mmol ArCl, 1.3 mmol cyclopropylamine, 0.02 mmol [Pd], Pd/L ratio 1:1, rt, 16 h in toluene. Isolated yields are reported. Acylation (3 mmol acetic anhydride, 60 °C, 24 h).

Aryl halides bearing electron-donating functional groups were converted in high yields but underwent rapid decomposition during workup, as reported before by Colacot et al.^37^ Crystallization attempts of the obtained compounds in DCM allowed the identification of two molecular structures of decomposition products, confirming the sensitivity of the compounds toward molecular oxygen. Oxidation results in the opening of the three-membered ring, leading to the formation of an amide and a primary alcohol (Figure 3).^23^ Although this oxidative decomposition reaction may seem to be an undesired side reaction, it also provides access to valuable reagents for further functionalization. For example, the products shown in Figure 4 bear structural similarities to key building blocks used in the synthesis of pigment yellow dyes, potentially facilitating the expansion of the scope of aromatic substituents in this chemistry.^66^

**Figure 3:**
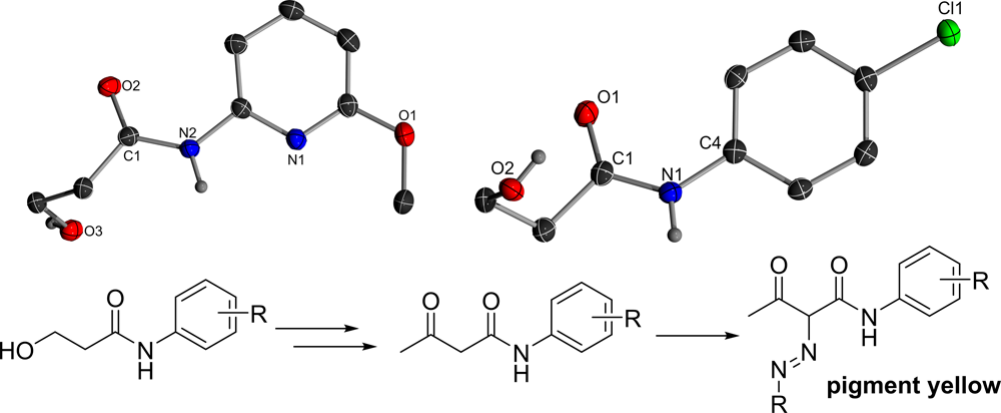
Molecular structures of the decomposition products of **10** and **24** after oxidation with molecular oxygen and a synthetic pathway to pigment yellow dyes from structurally similar building blocks.

**Figure 4:**
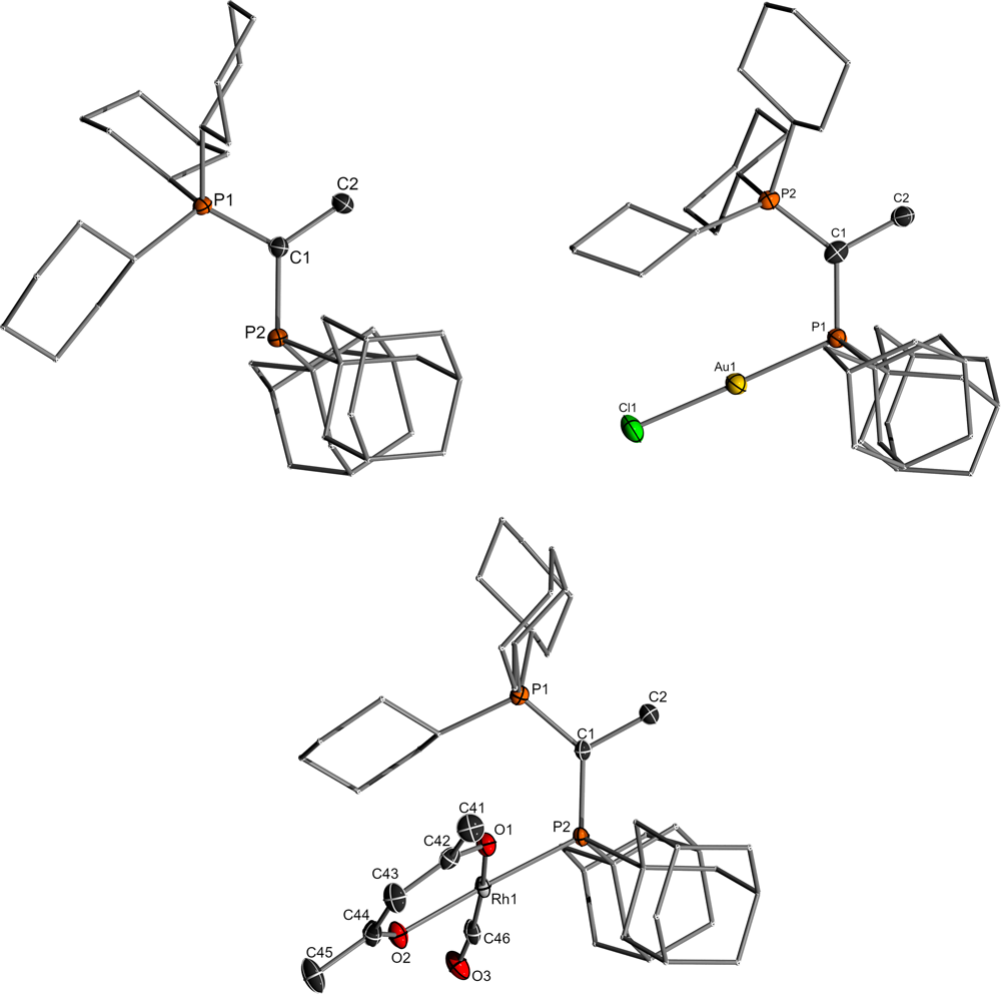
Molecular structures of **L5** (top, left) and its respective AuCl (top, right) and Rh complexes (bottom) used for the determination of %*V*_bur_ and TEP value.

For isolation of these more sensitive products, we directly acylated the formed arylated cyclopropylamine, which enabled a significant increase in the isolated yield, as illustrated by the comparison between products **14** and **15**. Using this acylation procedure, deactivated substrates including substrates with ether (**16**), trimethylsilyl (**17**), or vinyl (**18**) groups could successfully be coupled. Furthermore, several heteroaryl chlorides, such as pyridine (**21**–**23**), pyrazine (**24**), quinoline (**25**), benzoxazole (**26**), and benzothiophene (**27**), could be applied in this reaction. To some extent, substrates bearing acidic protons could be utilized; however, only moderate yields were observed due to side reactions. For example, **28** underwent α-arylation side reactions. Furthermore, late-stage functionalization was evaluated using the drug Fenofiberate, which resulted in a good 83% yield.

The diminished yields of some substrates can be attributed to incomplete conversion, as observed for compounds **16**, **19**, and **28**. For the electron-deficient aryl chloride **24**, also significant amounts of diarylated product were observed. Additionally, the formation of dehalogenated byproducts was detected in certain GC analyses, such as for compounds **6**, **26**, and **27**. Further limitations of the protocol with unsuccessful substrates are provided in the Supporting Information and could be probably attributed to highly electron-rich or electron-poor substrates (vide infra).

To further probe the generality of our protocol, it was applied in the monoarylation of larger cycloalkylamines up to cycloheptylamine. To our delight, excellent results were obtained for all tested ring sizes (Scheme 2). Cyclobutylamine exhibits the highest reactivity, leading to yields ranging from 70 to 95% (**31**–**34**). However, it was found that the yield slightly decreased with an increase in the ring size of the used cycloalkylamine. For example, the coupling of cycloheptylamine with aryl chlorides containing either an acyl (**43**) or methoxy group (**44**) resulted in the lowest yields of 30–55%. Furthermore, unlike with arylated cyclopropylamines, we did not observe an oxidative ring opening. Similar to the smaller three-membered counterpart, the other amines were also coupled with diverse aryl chlorides bearing a broad range of functional groups, such as amino (**37**) and thioether (**38**) groups, which were previously inaccessible. Additional aryl chlorides featured ether (**41** and **44**), fluoro (**36**), nitrile (**42**), and carbonyl functionalities (**43**). We further demonstrated that polyhalogenated arenes enabled the selective transformation of the more reactive bromide compared to that of chloride (**40**). Additionally, various heterocyclic aryl chlorides were successfully applied in this reaction, including pyridines (**33** and **41**), benzothiophene (**34**), and quinoline (**39**).

**Scheme 2:**
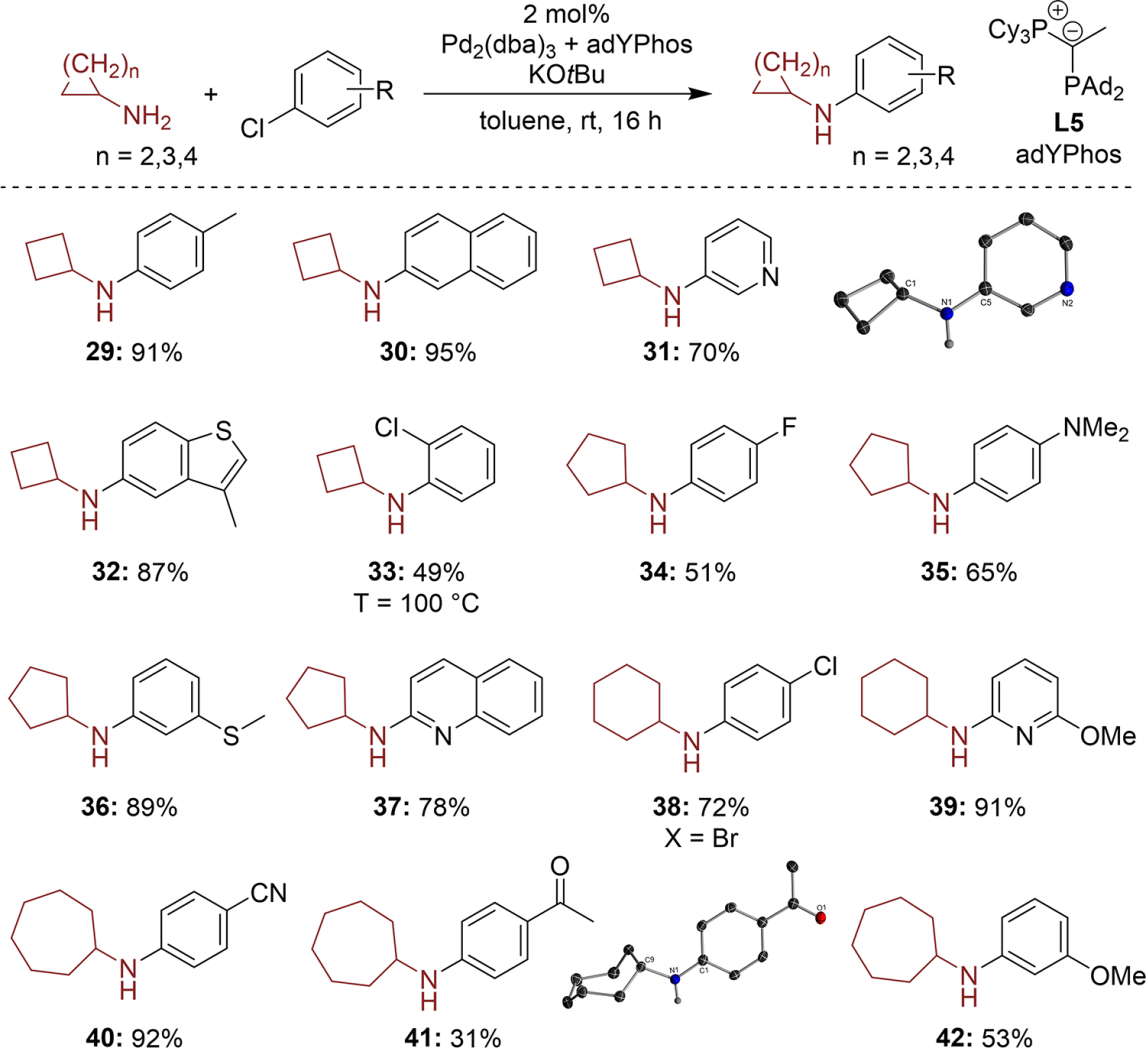
Scope for the Arylation of Cycloalkylamines of Different Ring Sizes^a^

a Reaction conditions: 1.0 mmol ArCl, 1.3 mmol cycloalkylamine, 0.02 mmol [Pd], Pd/L ratio 1:1, rt, 16 h in toluene. Isolated yields are reported.

### Ligand Properties and Mechanistic Implications

The adYPhos ligand (**L5**) was previously reported as part of a ligand set for screening purposes; however, its properties were not experimentally examined.^67^ The ligand is obtained as a colorless solid and is characterized by a set of doublets at 35.5 and 26.1 ppm in the ^31^P­{^1^H} NMR spectrum, with a coupling constant of 144.8 Hz. To quantify the donor properties, the Tolman Electronic Parameter (TEP) was determined from the CO vibration of the *in situ* prepared **L5Rh­(acac)­CO** complex.^10^ To confirm the identity of the complex, we determined its structure in the solid-state by X-ray diffraction (XRD) analysis (Figure 2 and Table 2). The rhodium complex features a CO vibration at 1936.0 cm^–1^ in DCM, correlating with a TEP value of 2045.1 cm^–1^ (see Supporting Information for details). Compared to related PCy_2_ containing YPhos ligands such as prYPhos (2053.6 cm^–1^),^68^ keYPhos (**L1**, 2050.1 cm^–1^),^44^ or trYPhos (**L4**, 2048.6 cm^–1^),^69^ this TEP value is significantly lower, underscoring the substantial electronic influence of the adamantyl substituents, making adYPhos the strongest electron-donating ligand among the YPhos ligands.

**Table 2:** Overview About Selected Bond Angles and Bond Lengths from the Molecular Structures of **L5**, **L5AuCl**, and **L5Rh­(acac)­CO** Complexes

	**L5**	**L5AuCl**	**L5Rh(acac)CO**
P^+^–C^–^–P [deg]	117.9(2)	127.9(3)	129.2(2)
P^+^–C^–^ [Å]	1.704(4)	1.745(5)	1.724(3)
C^–^–P [Å]	1.781(3)	1.756(5)	1.774(4)
P–M [Å]		2.266(1)	2.342(1)

Next, we measured the steric demand of **L5** by determining the percent buried volume %*V*_bur_ based on the geometry of its gold chloride complex. **L5AuCl** was isolated by filtration of the reaction mixture of Au­(tht)Cl and **L5** in pentane. The complex features two doublets at 63.2 and 30.6 ppm with a ^2^ *J*_PP_ coupling constant of 48.1 Hz in the ^31^P­{^1^H} NMR spectrum. Single crystals of the complex were obtained by slow evaporation of a saturated benzene solution, and the molecular structure was determined by XRD analysis (Figure 4). The %*V*_bur_ value calculated with the Sambvca program^70,71^ amounts to 50.9%, which is comparable to one of the *tert*-butyl-substituted trYPhos ligand **L4** (51.3%)^69^ and slightly higher than those of prYPhos (47.2%)^68^ and keYPhos (**L1**) (48.5%).^44^

A comparison of the molecular structure of **L5** with its gold and rhodium complex reveals that the ligand maintains a *syn*-arrangement of the methyl and adamantyl groups in all structures, similar to other reported YPhos ligands (Table 2). This arrangement places the metal in the pocket of the phosphonium group, thus resulting in its steric shielding. The P^+^–C and C^–^–P distances of **L5** are comparable to those of keYPhos (**L1**) (1.707(3) and 1.777(3) Å, respectively). The same applies to the P^+^–C^–^–P bond angle (119.1(2)°),^44^ resulting in an overall similar geometry of the two ligands. Interestingly, the comparison of the solid-state structures of the complexes with those of the free ligand shows that the P^+^–C^–^–P angle varies significantly, changing from 117.9° (**L5**) to 129.2° (**L5Rh­(acac)­CO**) upon complexation with a transition metal. This observation demonstrates that the ligand—despite the introduction of the bulky adamantyl substituents—maintains a significant degree of flexibility, which is important to accommodate substrates of different steric demand.

Overall, the study of ligand properties demonstrates that adYPhos is a highly electron-rich ligand but also a very bulky ligand. While the high donor strength accelerates oxidative addition, the steric demand facilitates reductive elimination. Since **L5** was superior to all other ligands tested, this suggests that both properties are decisive in the coupling of cyclopropylamine with aryl chlorides. The fact that the smaller ligands, **L1** and **L2**, only become active with the sterically more encumbered bis-ortho-substituted 2-chloro-m-xylene (Tables S7 and S8) argues for reductive elimination being the rate-limiting step.

To further investigate the nature of the rate-limiting step, we performed competition experiments. We investigated the conversion of two electronically different aryl chlorides in a parallel and one-pot reaction with a decreased catalyst loading of 1 mol %. For this purpose, we selected electron-rich 1-(*t*-butyl)-4-chlorobenzene and electron-poor 4-chlorobenzophenone as test substrates, as they provided excellent isolated yields. In the parallel experiments, both substrates achieved full conversion to **14** and **8** (Scheme 3). However, in the one-pot experiments, the electron-poor aryl chloride was preferentially consumed, suggesting that oxidative addition is rate-limiting since it substantially benefits from electron-deficient substituents. However, also, reductive elimination is in general assumed to be faster from electron-poor complexes. Therefore, we performed kinetic studies under standard conditions, employing an excess of aryl chloride (2 equiv) as well as an excess of the amine (2 equiv) (see Supporting Information for more details). The higher aryl chloride concentration resulted in an almost doubled initial reaction rate compared to standard conditions, consistent with first-order aryl chloride and oxidative addition being rate-limiting. However, the studies are complicated due to the increased production of diarylated product, which was only observed in traces when using 1 equiv of aryl chloride. Conversely, an increased concentration of cyclopropylamine led to a decreased reaction rate, indicating that amine coordination inhibits the catalyst activity.

**Scheme 3:**
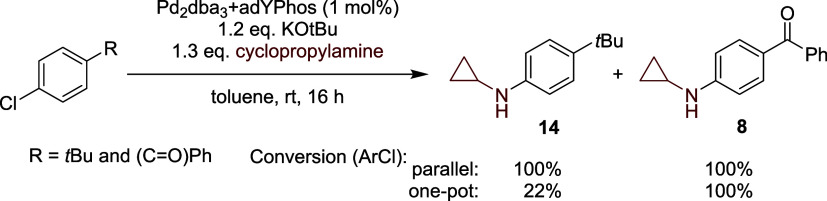
Competition Experiments, Comparing Electron-Rich and -Poor Aryl Chlorides

Overall, the kinetic studies clearly suggest that oxidative addition is rate-limiting, which is well in line with the superior performance of the most electron-rich ligand, adYPhos. However, we cannot exclude the possibility that reductive elimination also proceeds at a similar speed and might become rate-limiting depending on the nature of the substrate. A similar speed of oxidative addition and reductive elimination would be in line with the observed higher activity of the smaller ligands for sterically demanding substrates. However, the fact that excess cyclopropylamine slows product formation shows that the mechanism is more complicated and that product yield is affected by not only the rates of the individual elementary steps.

## Conclusions

In this study, we successfully developed a palladium-catalyzed protocol for the *N*-arylation of cyclopropylamine using a YPhos ligand. The inherent challenge of this coupling reaction was overcome with adYPhos, an electron-rich and sterically demanding adamantyl-substituted YPhos ligand, which was used for the *in situ* preparation of the Pd catalyst. This catalyst system enabled the coupling of a diverse range of substrates at mild reaction conditions, including those containing challenging functional groups such as amides, trimethylsilyl, and carbonyl substituents, which were previously inaccessible from (hetero)­aryl chlorides. Moreover, the protocol proved to be applicable to larger cycloalkylamines, demonstrating its versatility beyond cyclopropylamine.

## Supplementary Material



## Data Availability

The data underlying this study are available in the published article, in its Supporting Information, and openly available in the repository RESOLVdata at https://doi.org/10.17877/RESOLV-2025-MANYIQLI.

## Notes

Experimental procedures, full analysis data for new compounds, NMR spectra of all isolated compounds, details of the catalysis optimization, and crystal structure analysis. (PDF)
