# Supplementary material for: Palladium-Catalyzed Monoarylation of Cyclopropylamine
Source: J Org Chem. 2025 Aug 20;90(34):12153–62. doi: 10.1021/acs.joc.5c01233 (PMC12400431; doi:10.1021/acs.joc.5c01233)
Supplement: Supplementary file 1 [file jo5c01233_si_001.pdf]

Supporting Information

Palladium-Catalyzed Monoarylation of Cyclopropylamine

Nicolas Kaiser, Lea Räkow, Jens Handelmann, Viktoria H. Gessner*

Chair of Inorganic Chemistry II, Faculty of Chemistry and Biochemistry, Ruhr-University Bochum

Universitätsstr. 150, 44801 Bochum (Germany)

Table of Contents

1 Experimental Procedures S3

1.1 General Methods S3

1.2 Synthesis of adYPhos-AuCl S4

1.3 Determination of TEP values S4

1.4 Reaction Optimization S5

1.5 Catalyst Preformation Studies S8

1.6 Competition and Kinetic Experiments S10

1.7 General Experimental Procedures for Coupling Reactions S14

2 Characterization of the Catalysis Products S14

3 Further Substrates Tested in the Arylation of Cyclopropylamine S27

4 NMR Spectra S28

5 Crystal Structure Determination S78

6 References S87

The original files to all analysis methods have been deposited on a repository and can be downloaded via the following link. Sole exception are the single-crystal X-ray diffraction experiments. Cif files have been deposited with the CCDC (see chapter 1 and 5 for details).

Link to the data discussed in this manuscript: https://doi.org/10.17877/RESOLV-2025-MANYIQLI

1 Experimental Procedures

1.1 General Methods

Chemicals and conditions: If not stated otherwise, all experiments were carried out using standard Schlenk techniques under an argon atmosphere, which was dry and free of oxygen. Argon (99.999%) was a product of *Air Liquide* and was used without any further drying. An MBraun SPS 800 was used to dry solvents before their usage (THF, toluene, DCM, MeCN, *n*-pentane, *n*-hexane). All solvents were stored over molecular sieves under an argon atmosphere. Commercial substrates were used as received unless stated otherwise. Ligands **L1**[1], **L2** and **L3**[2], **L4**[3], **L5**[4], **L6**[5], **L7**-**L10**[4], **L11**[6] were prepared according to literature procedures.

NMR spectroscopy: ^{1}H, $^{13}C\{^{1}H\}$ and $^{31}P\{^{1}H\}$ NMR spectra were recorded on an Avance-III spectrometer at 25 °C if not stated otherwise. All values of the chemical shift are in ppm regarding the δ-scale. All spin-spin coupling constants (*J*) are printed in Hertz (Hz). To display multiplicities and signal forms correctly the following abbreviations were used: s = singlet, d = doublet, m = multiplet, br = broad signal. Signal assignment was supported by APT, HSQC and HMBC experiments.

IR-Spectra were recorded on a Thermo Nicolet iS5 FT-IR in transmission mode with a Specac "Omni-cell" with KBr plates and a 0.1 mm spacer for TEP values and with an iD7 ATR module at 22 °C

Melting points were measured with the SMP30 melting point apparatus from Stuart.

GC-MS/FID analyses were carried out using an HP-5 capillary column (Phenyl methyl siloxane, 30 m × 320 × 0.25, 100/2.3-30-300/3, 2 min at 60 °C, heating rate 30 °C/min, 3 or 10 min at 300 °C). Yields were determined by GC-FID using tetradecane as internal standard.

Single-Crystal XRD: Data collection of the compounds was conducted using an Rigaku Oxford Synergy diffractometer equipped with a PhotonJet (Cu) X-ray Source and a Hybrid Pixel array detector. The crystals of all compounds were mounted in an inert oil (perfluoropolyalkylether). Crystal structure determination were affected at 100 K. The structures were solved using dual space FT and direct methods, refined on F^2 using the ShelXL program and expanded using Fourier techniques.[7–10] All non-hydrogen atoms were refined anisotropically. Hydrogen atoms were included in structure factor calculations. If not stated otherwise in section 5, all hydrogen atoms were assigned to idealized geometric positions.

Crystallographic data (including structure factors) have been deposited with the Cambridge Crystallographic Data Centre as supplementary publication no. CCDC **2449584**-**2449593**. Copies of the data can be obtained free of charge on application to Cambridge Crystallographic Data Centre, 12 Union Road, Cambridge CB2 1EZ, UK; [fax: (+44) 1223-336-033; email: deposit@ccdc.cam.ac.uk]. Further information is given in section 5 of this document.

HRMS-ESI: An LTQ Orbitrap Velos (Thermo Fisher Scientific, Bremen, Germany) was used for direct infusion via a syringe pump. The heated desolvation capillary was set to 200°C and a spray voltage of 1.8 kV was supplied. In the tune file the LTQ Orbitrap was set to the following parameters (R = 30,000; IT = 500 ms; AGC Target = 1,000,000).

Column chromatography: Automated column chromatography was performed on a Reveleris X2 (BÜCHI) flash chromatography system.

1.2 Synthesis of adYPhos-AuCl

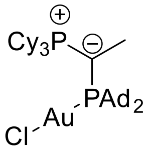

L5 (50.0 mg, 8.2 µmol) and chlorotetrahydrothiophenegold (25 mg, 7.8 µmol) were dissolved in pentane (2 ml) and the solution was stirred for 24 h. The solution was filtered, and the colorless solid was dried *in vacuo*. (30.1 mg, 3.62 µmol, 43% yield) Colorless crystals suitable for single crystal X-ray diffraction experiments were obtained by slow evaporation of a saturated benzene solution.

^{1}H NMR (400 MHz, C_6D_6): δ = 2.91 (s, 3H, PCy$_3$, *H*1), 2.52 – 2.44 (m, 6H, PAd$_2$, *H*2), 2.42 – 2.32 (m, 6H, PAd$_2$, *H*2), 2.00 – 1.93 (m, 12H, PCy$_3$, *H*2), 1.93 – 1.87 (dd, $^3J_{HP}$ = 13.16, 9.13 Hz, 3H, C*H*$_3$), 1.75 – 1.53 (m, 27H, PAd$_2$, *H*3+4, PCy$_3$, *H*3+4), 1.45 – 1.33 (m, 6H, PCy$_3$, *H*3), 1.17 – 1.05 (m, 3H, PCy$_3$, *H*4). **^{13}C{^{1}H} NMR (101 MHz, C_6D_6):** δ = 46.9 (dd, $^1J_{CP}$ = 30.9, $^3J_{CP}$ = 2.1 Hz, PAd$_2$, *C*1), 43.9 (d, $^2J_{CP}$ = 1.8 Hz, PAd$_2$, *C*2), 37.2 (s, PAd$_2$, *C*4), 35.1 (d, $^1J_{CP}$ = 48.8 Hz, PCy$_3$, *C*1), 29.6 (d, $^3J_{CP}$ = 9.5 Hz, PAd$_2$, *C*3), 28.8 (s, PCy$_3$, *C*2), 27.6 (d, $^3J_{CP}$ = 11.2 Hz, PCy$_3$, *C*3), 26.8 (s, PCy$_3$, *C*4), 19.2 (dd, $^2J_{CP}$ = 4.4, $^2J_{CP}$ = 2.0 Hz, *C*H$_3$), -1.9 (dd, $^1J_{CP}$ = 101.4, $^1J_{CP}$ = 62.9 Hz, P*C*P). **^{31}P{^{1}H} NMR (162 MHz, C_6D_6):** δ = 63.2 (d, $^2J_{PP}$ = 48.1 Hz, *P*Ad$_2$), 30.6 (d, $^2J_{PP}$ = 48.1 Hz, *P*Cy$_3$). **M.p.:** 200-202 °C (decomposition). **HRMS (ESI)** m/z: Calcd for $C_{40}H_{67}AuClP_2$ $[M+H]^+$ 841.4072; Found 841.4055.

1.3 Determination of TEP values

Procedure for ν_{CO}(Rh) determination: Rh(acac)(CO)$_2$ (5.00 mg, 38.8 µmol) and the phosphine (38.8 µmol) we dissolved in DCM (1 ml) in a screw cap vial. The solution was stirred for 15 min until gas evolution ceased. The solution was filled into the IR cell using a syringe. The cell was closed, and an IR spectrum was recorded, showing a strong band between 1950-1920 cm^{-1} corresponding to the carbonyl stretching frequency. From ν_{CO}(Rh) the TEP value was calculated based on a correlation reported by *Carrow et. al.*.[11] Colorless crystals suitable for single crystal X-ray diffraction experiments were obtained by slow evaporation of a saturated DCM solution.

$$TEP = 0.571611 \cdot \nu(CO)_{Rh} + 938.4736$$

$$TEP(adYPhos) = 0.571611 \cdot 1936.0\ 2045\ cm^1 + 938.4736 = 2045\ cm^1$$

1.4 Reaction Optimization

General Procedure for small-scale optimization of the model reaction:

An 8 ml screwcap vials with a teflon-coated stir bar and a septum cap was charged in a glovebox with KO*t*Bu (33.7 mg, 0.30 mmol). The vial was taken outside of the glovebox and cyclopropylamine (0.019 ml, 16.0 mg, 0.275 mmol, 1.1 eq.) and 4-chloroanisole (0.031 ml, 36.4 mg, 0.25 mmol, 1 eq.) were added as stock solutions in toluene (0.55 ml). In the glovebox, a second vial was charged with an equimolar amount (3 mol%) of the free ligand and Pd_2dba_3. The vial was also taken out of the glovebox and the catalyst was allowed to preform in toluene (0.5 ml) by stirring for 30 min/6 h. The catalyst solution was added to the reaction mixture and stirred for 16 h (unoptimized) at room temperature. The reaction mixture was quenched with water (0.5 ml) and was diluted with ethyl acetate (5 ml) and tetradecane (50.1 mg, 0.065 ml, 0.25 mmol) as internal standard. Small aliquots were removed and filtered through silica with ethyl acetate and analyzed by GC-FID.

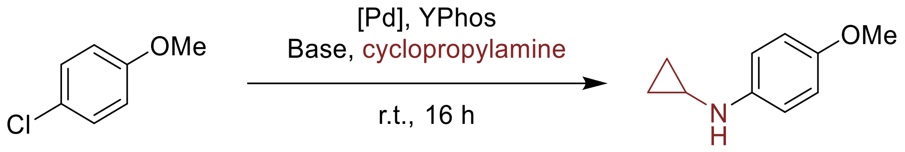

Table S1. Screening results for the optimal ligand for the arylation of cyclopropylamine with 4-chloroanisole.

Entry	Ligand	Solvent	Base	Base eq.	Amine eq.	conc. [M]	Pd Source	Pd Loading	Conversion %
1	**L1**	Toluene	KO*t*Bu	1.2	1.1	0.25	Pd_2dba_3	3 mol%	21
2	**L2**	Toluene	KO*t*Bu	1.2	1.1	0.25	Pd_2dba_3	3 mol%	24
3	**L3**	Toluene	KO*t*Bu	1.2	1.1	0.25	Pd_2dba_3	3 mol%	25
4	**L4**	Toluene	KO*t*Bu	1.2	1.1	0.25	Pd_2dba_3	3 mol%	50
5	**L5**	Toluene	KO*t*Bu	1.2	1.1	0.25	Pd_2dba_3	3 mol%	56
6	**L6**	Toluene	KO*t*Bu	1.2	1.1	0.25	Pd_2dba_3	3 mol%	27
7	**L7**	Toluene	KO*t*Bu	1.2	1.1	0.25	Pd_2dba_3	3 mol%	31
8	**L8**	Toluene	KO*t*Bu	1.2	1.1	0.25	Pd_2dba_3	3 mol%	21
9	**L9**	Toluene	KO*t*Bu	1.2	1.1	0.25	Pd_2dba_3	3 mol%	23
10	**L10**	Toluene	KO*t*Bu	1.2	1.1	0.25	Pd_2dba_3	3 mol%	21
11	**L11**	Toluene	KO*t*Bu	1.2	1.1	0.25	Pd_2dba_3	3 mol%	44
12	**L12**	Toluene	KO*t*Bu	1.2	1.1	0.25	Pd_2dba_3	3 mol%	8
13	**L13**	Toluene	KO*t*Bu	1.2	1.1	0.25	Pd_2dba_3	3 mol%	8
14	**L14**	Toluene	KO*t*Bu	1.2	1.1	0.25	Pd_2dba_3	3 mol%	0
15	**L15**	Toluene	KO*t*Bu	1.2	1.1	0.25	Pd_2dba_3	3 mol%	18
16	**L16**	Toluene	KO*t*Bu	1.2	1.1	0.25	Pd_2dba_3	3 mol%	9
17	**L17**	Toluene	KO*t*Bu	1.2	1.1	0.25	Pd_2dba_3	3 mol%	16
18	**L18**	Toluene	KO*t*Bu	1.2	1.1	0.25	Pd_2dba_3	3 mol%	0

Reaction conditions: 30 min preformation time for [Pd]·L, 0.25 mmol ArCl, rt, 16 h. Conversion determined by calibrated GC analysis using tetradecane as internal standard.

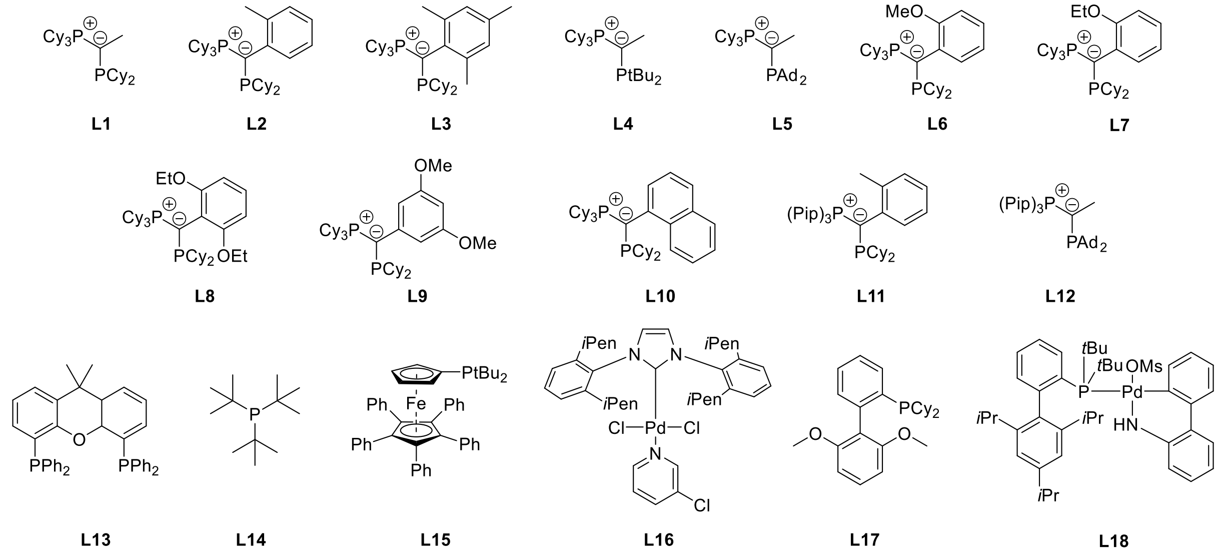

Figure S1. Overview about all screened ligands and Pd precursors.

Table S2. Screening results for the optimal solvent for the arylation of cyclopropylamine with 4-chloroanisole.

Entry	Ligand	Solvent	Base	Base eq.	Amine eq.	conc. [M]	Pd Source	Pd loading	Conversion %
19	**L5**	Toluene	KO*t*Bu	1.2	1.1	0.25	Pd_2dba_3	3 mol%	59
20	**L5**	Dioxane	KO*t*Bu	1.2	1.1	0.25	Pd_2dba_3	3 mol%	29
21	**L5**	THF	KO*t*Bu	1.2	1.1	0.25	Pd_2dba_3	3 mol%	10
22	**L5**	Et_2O	KO*t*Bu	1.2	1.1	0.25	Pd_2dba_3	3 mol%	30
23	**L5**	CPME	KO*t*Bu	1.2	1.1	0.25	Pd_2dba_3	3 mol%	26

Reaction conditions: 30 min preformation time for [Pd]·L, 0.25 mmol ArCl, rt, 16 h. Conversion determined by calibrated GC analysis using tetradecane as internal standard.

Table S3. Screening results for the optimal base for the arylation of cyclopropylamine with 4-chloroanisole.

Entry	Ligand	Solvent	Base	Base eq.	Amine eq.	conc. [M]	Pd Source	Pd loading	Conversion %
24	**L5**	Toluene	LiTMP	1.2	1.1	0.25	Pd_2dba_3	3 mol%	10
25	**L5**	Toluene	LDA	1.2	1.1	0.25	Pd_2dba_3	3 mol%	8
26	**L5**	Toluene	NaO*t*Bu	1.2	1.1	0.25	Pd_2dba_3	3 mol%	48
27	**L5**	Toluene	KO*t*Bu	1.2	1.1	0.25	Pd_2dba_3	3 mol%	55
28	**L5**	Toluene	LiHMDS	1.2	1.1	0.25	Pd_2dba_3	3 mol%	32
29	**L5**	Toluene	NaHMDS	1.2	1.1	0.25	Pd_2dba_3	3 mol%	8
30	**L5**	Toluene	KHMDS	1.2	1.1	0.25	Pd_2dba_3	3 mol%	8
31	**L5**	Toluene	DBU	1.2	1.1	0.25	Pd_2dba_3	3 mol%	2
32	**L5**	Toluene	NaOH	1.2	1.1	0.25	Pd_2dba_3	3 mol%	9
33	**L5**	Toluene	NaH	1.2	1.1	0.25	Pd_2dba_3	3 mol%	22

Reaction conditions: 30 min preformation time for [Pd]·L, 0.25 mmol ArCl, rt, 16 h. Conversion determined by calibrated GC analysis using tetradecane as internal standard.

Table S4. Screening results for the optimal concentration, amounts of base and amine for the arylation of cyclopropylamine with 4-chloroanisole.

Entry	Ligand	Solvent	Base	Base eq.	Amine eq.	conc. [M]	Pd Source	Pd loading	Conversion %
34	**L5**	Toluene	NaO*t*Bu	1.0	1.2	0.5	Pd_2dba_3	3 mol%	45
35	**L5**	Toluene	NaO*t*Bu	1.2	1.2	0.5	Pd_2dba_3	3 mol%	55
36	**L5**	Toluene	NaO*t*Bu	1.5	1.2	0.5	Pd_2dba_3	3 mol%	53
37	**L5**	Toluene	NaO*t*Bu	3	1.2	0.5	Pd_2dba_3	3 mol%	53
38	**L5**	Toluene	NaO*t*Bu	1.2	1.0	0.5	Pd_2dba_3	3 mol%	64
39	**L5**	Toluene	NaO*t*Bu	1.2	1.2	0.5	Pd_2dba_3	3 mol%	54
40	**L5**	Toluene	NaO*t*Bu	1.2	1.5	0.5	Pd_2dba_3	3 mol%	54
41	**L5**	Toluene	NaO*t*Bu	1.2	3	0.5	Pd_2dba_3	3 mol%	38
42	**L5**	Toluene	NaO*t*Bu	1.2	1.1	1	Pd_2dba_3	3 mol%	61
42	**L5**	Toluene	NaO*t*Bu	1.2	1.1	0.5	Pd_2dba_3	3 mol%	60
44	**L5**	Toluene	NaO*t*Bu	1.2	1.1	0.25	Pd_2dba_3	3 mol%	56
45	**L5**	Toluene	NaO*t*Bu	1.2	1.1	0.1	Pd_2dba_3	3 mol%	48

Reaction conditions: 30 min preformation time for [Pd]·L, 0.5 mmol ArCl, rt, 24 h. Conversion determined by calibrated GC analysis using tetradecane as internal standard.

Table S5. Screening results for the optimal Pd precursor, catalyst loading and temperature for the arylation of cyclopropylamine with 4-chloroanisole.

Entry	Ligand	Solvent	Base	Base eq.	Amine eq.	conc. [M]	Pd Source	Pd loading	Conversion %
46	**L5**	Toluene	KO*t*Bu	1.2	1.1	0.25	Pd_2dba_3	3 mol%	53
47	**L5**	Toluene	KO*t*Bu	1.2	1.1	0.25	$[Pd(allyl)Cl]_2$	3 mol%	46
48	**L5**	Toluene	KO*t*Bu	1.2	1.1	0.25	$[Pd(cinn)Cl]_2$	3 mol%	46
49	**L5**	Toluene	KO*t*Bu	1.2	1.1	0.25	$[Pd(ind)Cl]_2$	3 mol%	1
50	**L5**	Toluene	KO*t*Bu	1.2	1.1	0.25	$Pd(COD)Cl_2$	3 mol%	40
51	**L5**	Toluene	KO*t*Bu	1.2	1.1	0.25	Pd_2dba_3	1 mol%	38[a]
52	**L5**	Toluene	KO*t*Bu	1.2	1.1	0.25	Pd_2dba_3	3 mol%	61[a]
53	**L5**	Toluene	KO*t*Bu	1.2	1.1	0.25	Pd_2dba_3	3 mol%	64[a,b]

Reaction conditions: 30 min preformation time for [Pd]·L, 0.25 mmol ArCl, rt, 16 h. Conversion determined by calibrated GC analysis using tetradecane as internal standard. [a]longer preformation time of 6 h. [b]at 60 °C.

Table S6. Screening results for the catalyst loading and amine eq. for the arylation of cyclopropylamine with 4-chlorotoluene.

Entry	Ligand	Solvent	Base	Base eq.	Amine eq.	conc. [M]	Pd Source	Pd loading	Conversion %
54	**L5**	Toluene	KO*t*Bu	1.2	1.1	0.25	Pd_2dba_3	1 mol%	52
55	**L5**	Toluene	KO*t*Bu	1.2	1.1	0.25	Pd_2dba_3	2 mol%	98 (8)[a,b]
56	**L5**	**Toluene**	**KO*t*Bu**	**1.2**	**1.3**	**0.25**	**Pd_2dba_3**	**2 mol%**	**96 (5)[a,b]**
57	**L5**	Toluene	KO*t*Bu	1.2	1.5	0.25	Pd_2dba_3	2 mol%	91 (2)[a,b]

Reaction conditions: 30 min preformation time for [Pd]·L, 0.25 mmol ArCl, rt, 16 h. Conversion determined by calibrated GC analysis using tetradecane as internal standard. [a]longer preformation time of 6 h. [b]diarylated product.

Table S7. Screening results for the arylation of cyclopropylamine with 2-chlorotoluene.

Entry	Ligand	Solvent	Base	Base eq.	Amine eq.	conc. [M]	Pd Source	Pd loading	Yield %
58	**L1**	Toluene	KO*t*Bu	1.2	1.3	0.25	Pd_2dba_3	2 mol%	0
59	**L2**	Toluene	KO*t*Bu	1.2	1.3	0.25	Pd_2dba_3	2 mol%	0
60	**L4**	Toluene	KO*t*Bu	1.2	1.3	0.25	Pd_2dba_3	2 mol%	17
61	**L5**	Toluene	KO*t*Bu	1.2	1.3	0.25	Pd_2dba_3	2 mol%	70
62	**L7**	Toluene	KO*t*Bu	1.2	1.3	0.25	Pd_2dba_3	2 mol%	20

Reaction conditions: 6 h preformation time for [Pd]·L, 0.25 mmol ArCl, rt, 16 h. Yields determined by calibrated GC analysis using tetradecane as internal standard. [a]Dehalogenated product could not be monitored, because toluene was used as solvent.

Table S8. Screening results for the arylation of cyclopropylamine with 2-chloro-*m*-xylene.

Entry	Ligand	Solvent	Base	Base eq.	Amine eq.	conc. [M]	Pd Source	Pd loading	Yield %
63	**L1**	Toluene	KO*t*Bu	1.2	1.3	0.25	Pd_2dba_3	2 mol%	29 (16)[a]
64	**L2**	Toluene	KO*t*Bu	1.2	1.3	0.25	Pd_2dba_3	2 mol%	47 (7)[a]
65	**L4**	Toluene	KO*t*Bu	1.2	1.3	0.25	Pd_2dba_3	2 mol%	0 (12)[a]
66	**L5**	Toluene	KO*t*Bu	1.2	1.3	0.25	Pd_2dba_3	2 mol%	0 (12)[a]
67	**L7**	Toluene	KO*t*Bu	1.2	1.3	0.25	Pd_2dba_3	2 mol%	22 (21)[a]

Reaction conditions: 6 h preformation time for [Pd]·L, 0.25 mmol ArCl, rt, 16 h. Yields determined by calibrated GC analysis using tetradecane as internal standard. [a]dehalogenated product.

1.5 Catalyst Preformation Studies

A *J. Young* NMR tube was charged with adYphos (**L5**) (1 eq.), Pd_2dba_3 (1 eq.) and 0.6 ml of either THF or toluene. The mixture was thoroughly shaken for a few seconds. To monitor the reaction progress, $^{31}P\{^1H\}$ NMR spectra were recorded at various time intervals. Triphenylphosphine oxide (1 eq.) was added as an internal standard to ensure that no significant amounts of precipitate were overlooked in the dark-colored solution.

The preformation of the catalyst **L5-Pd(dba)** was significantly faster in THF compared to toluene (Figure S2 and Figure S3). In THF, the reaction was almost complete after 15 min, whereas in toluene, approximately 50% of the ligand was coordinated after 2 hours. After 16 h, the reaction was almost finished, but black precipitate was observed in the solution, indicating partial decomposition of **L5-Pd(dba)**. Consequently, an intermediate preformation time of 6 h was chosen as balance between optimal preformation and avoiding decomposition.

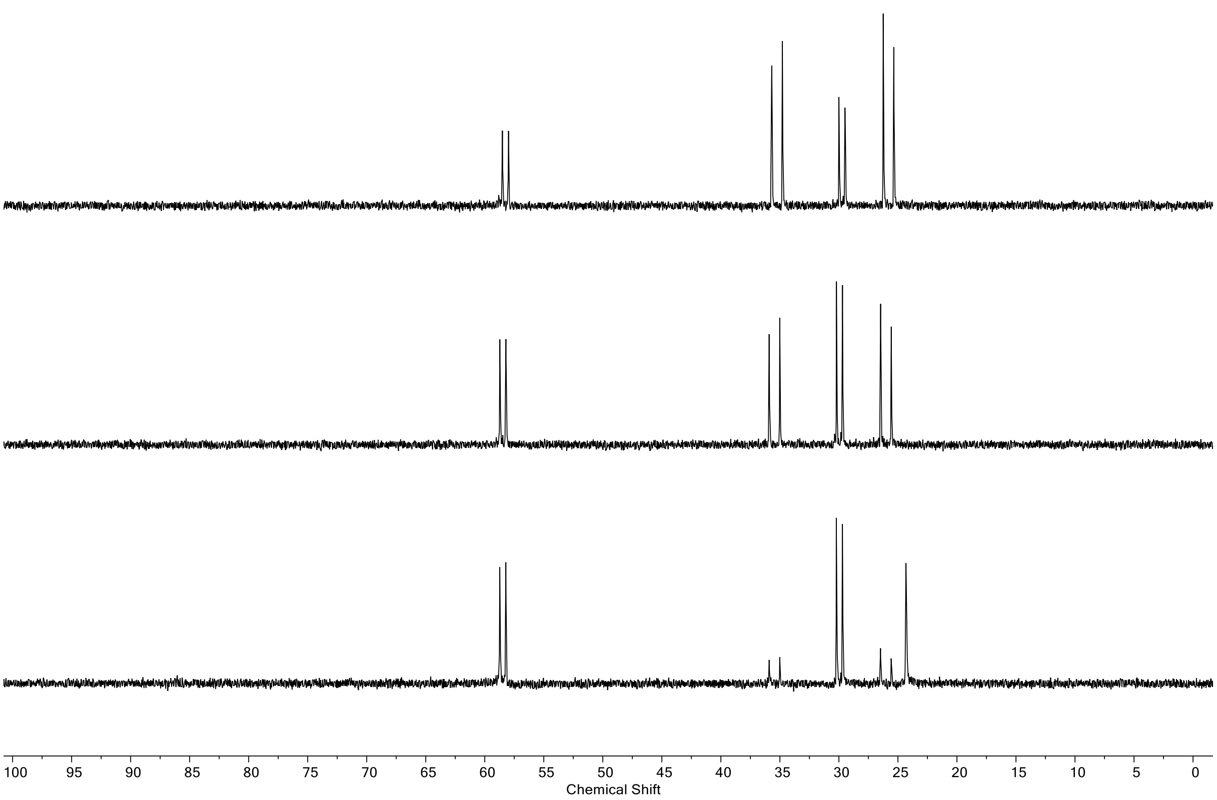

Figure S2. $^{31}P\{^{1}H\}$ NMR spectrum preformation of **L5-Pd(dba)** in toluene after 40 min (top), 2 h (middle) and 16 h (bottom).

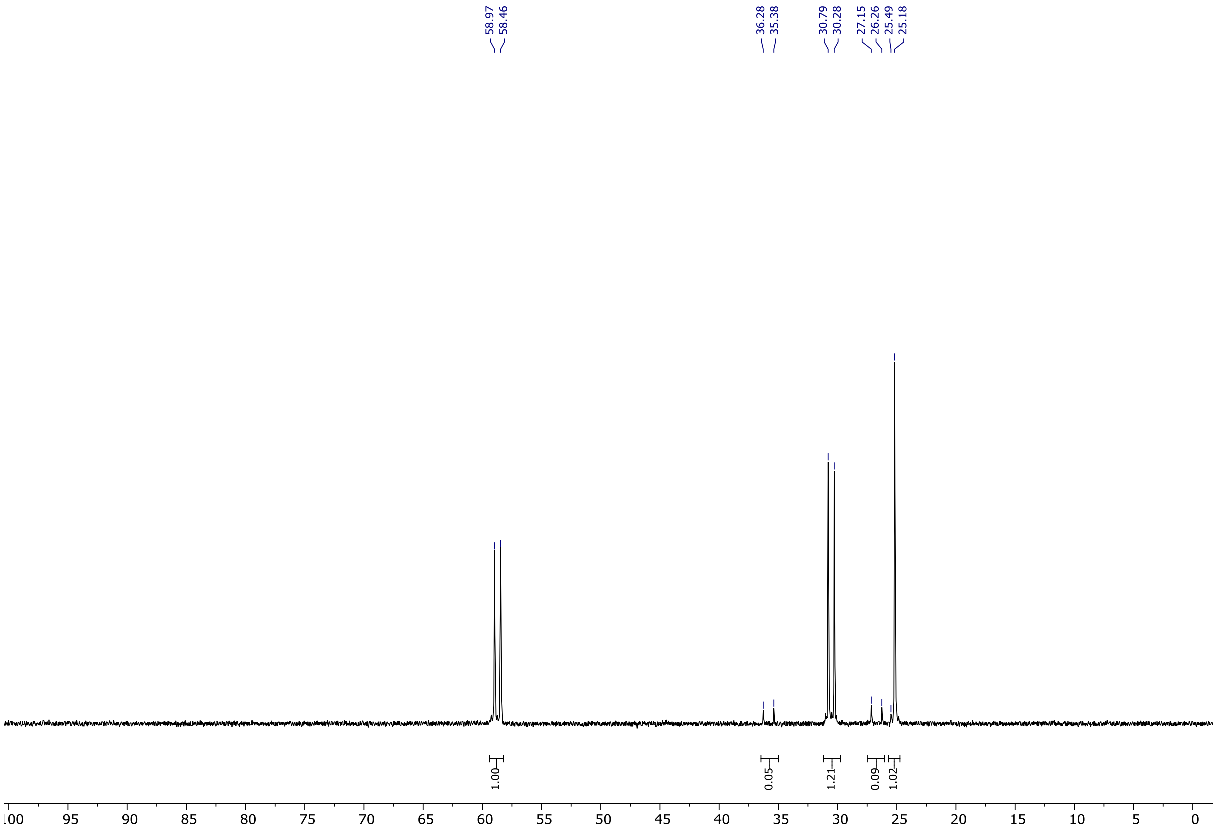

Figure S3. $^{31}P\{^{1}H\}$ NMR spectrum preformation of **L5-Pd(dba)** in THF after 15 min.

1.6 Competition and Kinetic Experiments

Competition experiment. The competition experiment was performed following the general procedure *A.* The reactions were analyzed by GC analysis with *n*-tetradecane as internal standard.

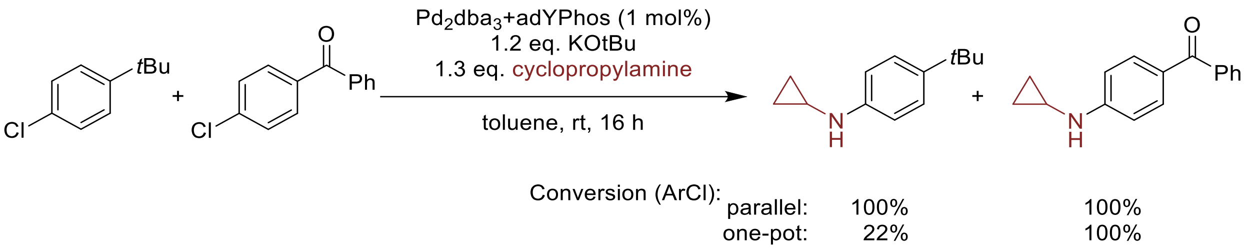

The results demonstrate that in a one-pot reaction the electron-deficient aryl chloride is predominantly coupled.

Kinetic studies. The kinetic experiments were performed in Schlenk flasks following the general procedure *A* on a 2 mmol reaction scale and diluted to 0.125 M (16 mL) to ensure consistent stirring and homogeneity of the mixture, due to the precipitation of potassium chloride and *tert*-butanol during the reaction. The reactions were analyzed by GC analysis using *n*-tetradecane as an internal standard.

Experiment 1: 2.00 mmol *tert*-butylchlorobenzene, 2.6 mmol cyclopropylamine, 2.4 mmol KO*t*Bu, 2 mol% catalyst, 16 ml toluene.

Experiment 2: 4.00 mmol *tert*-butylchlorobenzene, 2.6 mmol cyclopropylamine, 2.4 mmol KO*t*Bu, 2 mol% catalyst, 16 ml toluene.

Experiment 3: 2.00 mmol *tert*-butylchlorobenzene, 5.2 mmol cyclopropylamine, 2.4 mmol KO*t*Bu, 2 mol% catalyst, 16 ml toluene.

Table S9. Results of the kinetic studies for the amination of *tert*-butylchlorobenzene with cyclopropylamine.

Time [min]	Yield E1 [%]	Yield E2 [%] (monoarylation)	Yield E2 [%] (diarylation)	Yield E3 [%]
0	0	0	0	0
10	20	32	1	13
20	42	41	1	25
30	49	45	3	35
40	52	47	6	40
50	55	45	12	42
60	57	40	18	44
70	58	37	22	45
80	62	36	23	46
90	63	34	25	49
100	64	33	26	49

110	67	32	27	51
120	68	31	28	49
180	74	30	30	55

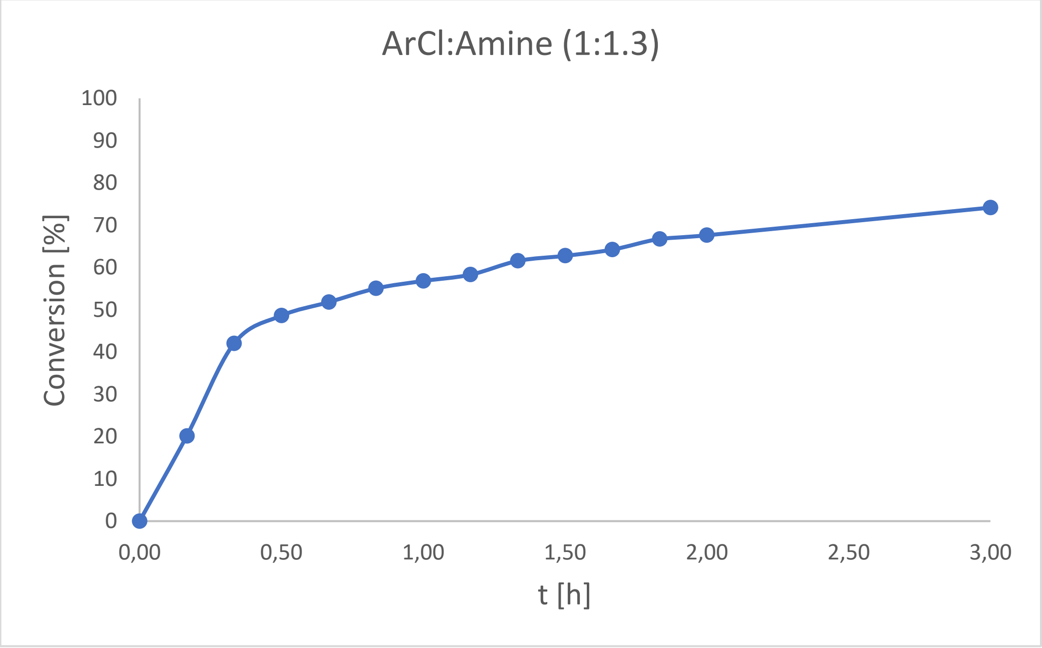

Figure S4. Conversion-time plot for the amination of *tert*-butylchlorobenzene (1 eq.) with cyclopropylamine (1.3 eq.).

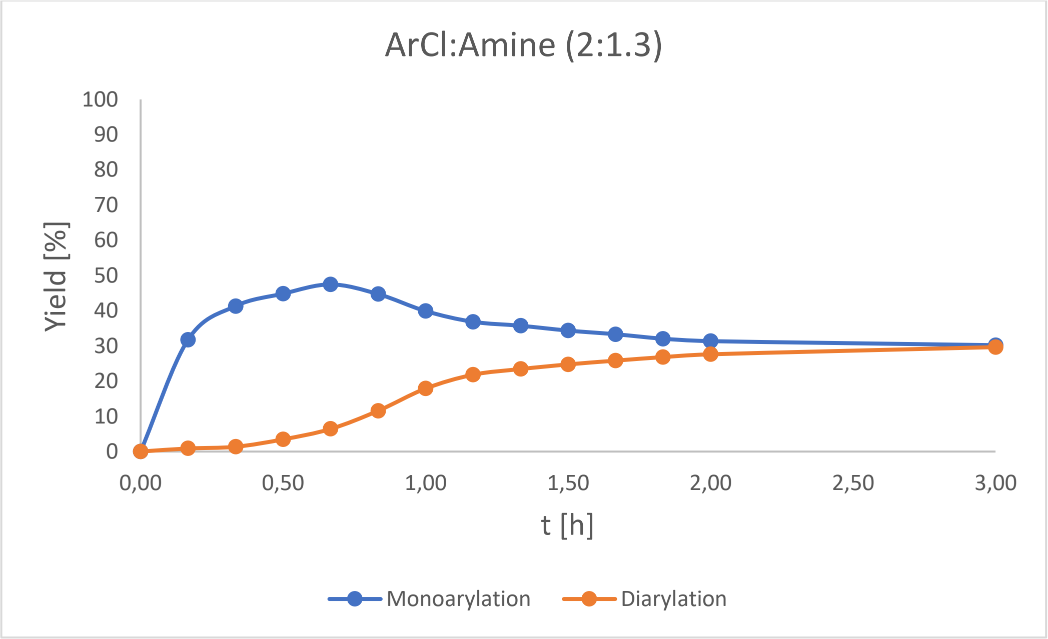

Figure S5. Conversion-time plot for the amination of *tert*-butylchlorobenzene (2 eq.) with cyclopropylamine (1.3 eq.). Yield is calculated with respect to the amine in this experiment.

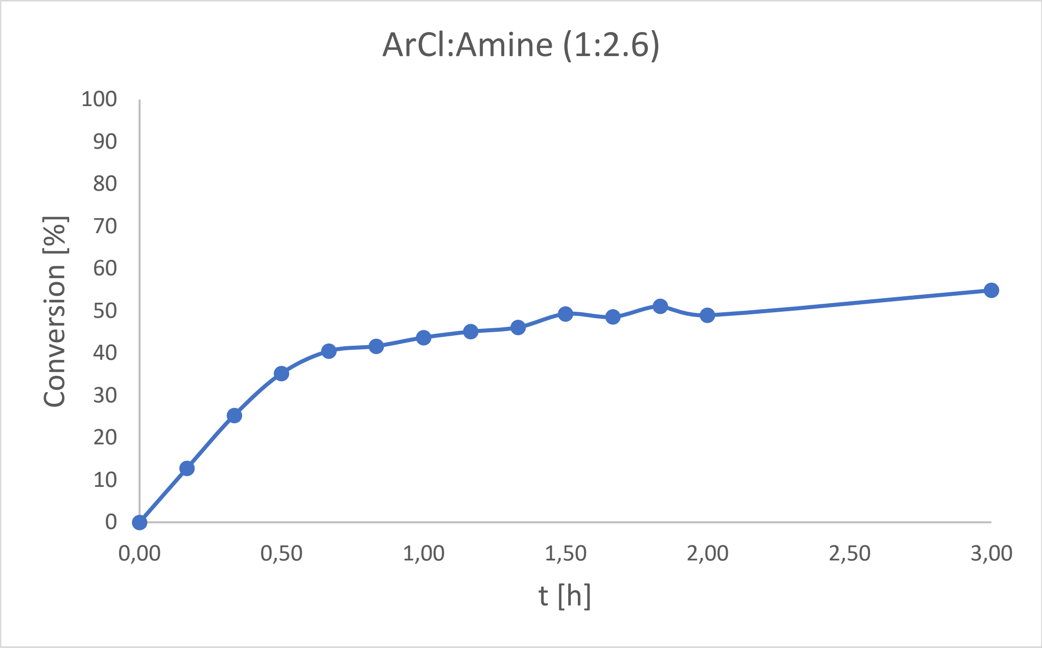

Figure S6. Conversion-time plot for the amination of *tert*-butylchlorobenzene (1 eq.) with cyclopropylamine (2.6 eq.).

Table S10. Reaction rates of the kinetic studies for the amination of *tert*-butylchlorobenzene with cyclopropylamine.

Time [min]	Reaction Rate E3 [mmol/(l*min)]	Reaction Rate E2 [mmol/(l*min)]	Reaction Rate E3 [mmol/(l*min)]
0	0,00	0,00	0,00
10	2,52	5,16	1,59
20	2,73	1,55	1,57
30	0,82	0,57	1,24
40	0,40	0,43	0,66
50	0,41	-0,44	0,15
60	0,22	-0,79	0,25
70	0,19	-0,50	0,18
80	0,41	-0,18	0,13
90	0,15	-0,22	0,40
100	0,18	-0,17	-0,09
110	0,32	-0,21	0,32

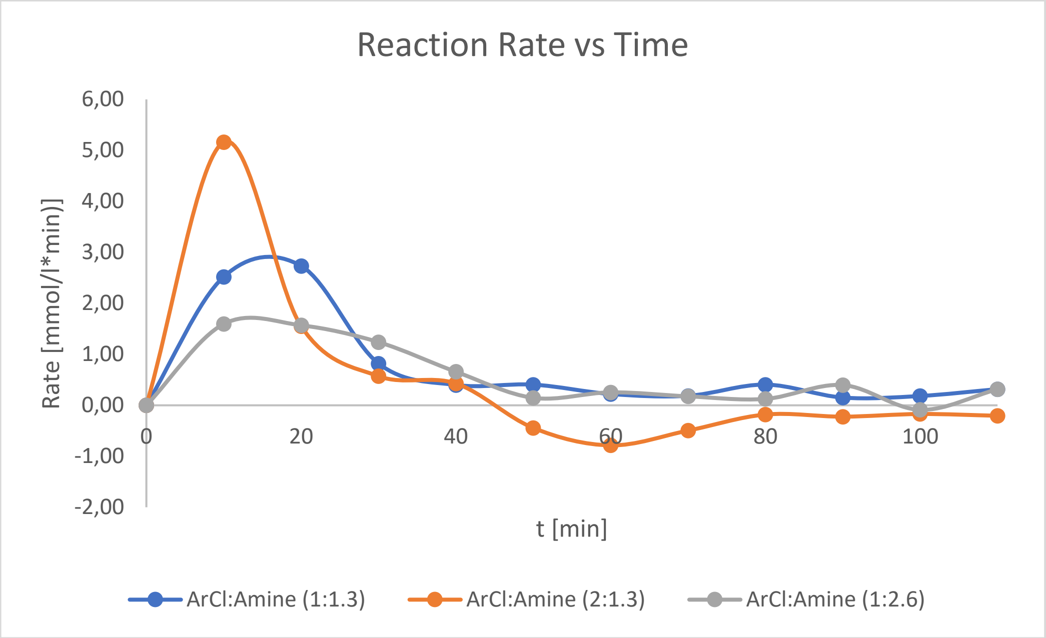

Figure S7. Plot of the different excess experiments: reaction rate [mmol/(l*min)] versus time [min]. Blue (I: no excess), orange (II, excess ArCl), grey (III: excess amine). Negative reaction rate results from the consumption for the diarylation.

Kinetic studies were performed under optimized reaction conditions, employing an excess of aryl chloride (2 eq.) as well as an excess of the amine (2 eq.) (see above for exact concentrations). The higher aryl chloride concentration (orange plot, Figure S7) resulted in an almost doubled initial reaction rate compared to standard conditions, consistent with a first order in aryl chloride and oxidative addition being rate-limiting. However, the studies are complicated due to the increased production of diarylated product (orange plot, Figure S5), which was only observed in traces when using 1 equiv. of aryl chloride. This finding is particularly intriguing, as the diarylated product begins to form within minutes, rather than after the complete consumption of the naked cyclopropylamine. This observation underscores the profound influence of aryl chloride concentration on the reaction's behavior.

In stark contrast, an excess of cyclopropylamine decelerates the reaction, indicating a potential negative reaction order with respect to the amine. This kinetic behavior may help elucidate the limitations observed with certain substrates, as the arylated cyclopropylamines could potentially inhibit the catalyst's activity.

1.7 General Experimental Procedures for Coupling Reactions

General procedure *A* for synthesizing monoarylated cycloalkylamines:

An 8 ml screwcap vial equipped with a Teflon-coated stir bar and a septum cap was charged with (hetero)aryl halide (1 mmol, 1 eq.) if solid and KO*t*Bu (135 mg, 1.2 mmol, 1.2 eq.) in the glovebox. The vial was taken outside of the glovebox and (hetero)aryl halide (1 mmol, 1 eq.) if liquid, cycloalkylamine (1.3 mmol, 1.3 eq.) and toluene (2 ml) were added via syringe. In the glovebox, a second vial was charged with adYPhos (12.2 mg, 0.02 mmol, 0.02 eq.) and $Pd_2(dba)_3$ (13.6 mg, 0.02 mmol, 0.02 eq.) and taken out of the glovebox. The catalyst was allowed to preform in toluene (2 ml) by stirring for 6 h. The catalyst solution was added to the reaction mixture and stirred at room temperature for 16 h. After that, the vial was opened to air and the reaction mixture was diluted with approx. 4 ml EtOAc, filtered through a pad of Celite and concentrated in vacuo. The crude product was purified via automated column chromatography.

General procedure *B* for synthesizing monoarylated and acylated cyclopropylamines:

An 8 ml screwcap vial equipped with a Teflon-coated stir bar and a septum cap was charged with (hetero)aryl halide (1 mmol, 1 eq.) if solid and KO*t*Bu (135 mg, 1.2 mmol, 1.2 eq.) in the glovebox. The vial was taken outside of the glovebox and (hetero)aryl halide (1 mmol, 1 eq.) if liquid, cyclopropylamine (0.0948 ml, 1.3 mmol, 1.3 eq.) and toluene (2 ml) were added via syringe. In the glovebox, a second vial was charged with adYPhos (12.2 mg, 0.02 mmol, 0.02 eq.) and $Pd_2(dba)_3$ (13.6 mg, 0.02 mmol, 0.02 eq.) and taken out of the glovebox. The catalyst was allowed to preform in toluene (2 ml) by stirring for 6 h at room temperature. The catalyst solution was added to the reaction mixture and stirred at room temperature for 16 h. After that, acetic anhydride (0.285 ml, 3 mmol, 3 eq.) was added via syringe and the mixture was stirred at 60 °C for another 24 h. The vial was then opened to air and the reaction mixture was diluted with approx. 4 ml EtOAc, filtered through a pad of Celite and concentrated in vacuo. The crude product was purified via automated column chromatography.

2 Characterization of the Catalysis Products

1: *N*-cyclopropyl-3-methoxyaniline

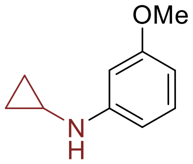

Amine **1** was synthesized according to general procedure *A* using the corresponding aryl chloride/bromide/iodide and cyclopropylamine. Purification by column chromatography (5% EtOAc/hexane) resulted in yellow oils (from aryl chloride 137.8 mg, 84% yield; from aryl bromide: 131.9 mg, 81% yield; from aryl iodide: 121.9 mg, 75% yield; The analytical data is in accordance with the reported literature.[12] **^{1}H NMR (400 MHz, $CDCl_3$):** δ = 7.13 – 7.06 (m, 1H), 6.42 – 6.36 (m, 2H), 6.34 – 6.29 (m, 1H), 4.21 (s, 1H), 3.79 (s, 3H), 2.46 – 2.39 (m, 1H), 0.76 – 0.70 (m, 2H), 0.55 – 0.49 (m, 2H). **$^{13}C\{^1H\}$ NMR (101 MHz, $CDCl_3$):** δ = 160.9, 150.2, 130.0, 106.5, 102.9, 99.3, 55.2, 25.4, 7.5. **MS (EI):** m/z (%) = 163 (100), 162 (85), 148 (34), 134 (45), 132,1 (32), 107 (30), 77 (28).

Scale-up: To test the scalability of the protocol, the coupling of *m*-anisole chloride was performed on a 10 mmol scale, with all other reagents being scaled-up accordingly. Product **1** could be isolated in 70% yield (1.14 g).

2: *N*-cyclopropyl-3,5-dimethoxyaniline

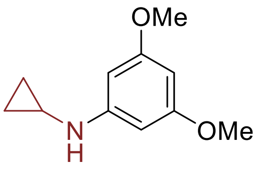

Amine **2** was synthesized according to general procedure *A* using the corresponding aryl chloride and cyclopropylamine. Purification by column chromatography (5% EtOAc/hexane) resulted in 156.8 mg of a yellow oil (81% yield). **^{1}H NMR (400 MHz, $CDCl_3$):** δ = 5.99 (d, *J* = 2.1 Hz, 2H), 5.93 – 5.91 (m, 1H), 4.26 (s, 1H), 3.76 (s, 6H), 2.45 – 2.39 (m, 1H), 0.75 – 0.67 (m, 2H), 0.54 – 0.48 (m, 2H). **$^{13}C\{^1H\}$ NMR (101 MHz, $CDCl_3$):** δ = 161.8, 150.8, 92.2, 90.2, 55.3, 25.4, 7.6. **IR (cm^{-1}):** 3354 (w), 2936 (w), 2838 (w), 1672 (m), 1594 (s), 1508 (m), 1482 (m), 1450 (s), 1421 (s), 1363 (m), 1261 (w), 1231 (m), 1193 (s), 1146 (s) 1058 (s), 965 (w), 932 (m), 808 (s), 683 (m), 538 (m). **HRMS (ESI)** m/z: Calcd for $C_{11}H_{15}NO_2$ $[M]^+$ 193.1103; Found 193.1101. **MS (EI):** m/z (%) = 193 (100), 192 (47), 178 (52), 164 (23), 162 (24), 137 (26), 122 (17).

3: 4-(cyclopropylamino)benzonitrile

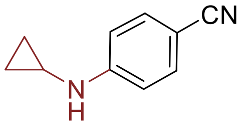

Amine **3** was synthesized according to general procedure *A* using the corresponding aryl chloride and cyclopropylamine. Purification by column chromatography (7% EtOAc/hexane) resulted in 126.6 mg of a yellow solid (80% yield). The analytical data is in accordance with the reported literature.[13] **^{1}H NMR (400 MHz, $CDCl_3$):** δ = 7.48 – 7.39 (m, 2H), 6.79 – 6.70 (m, 2H), 4.65 (s, 1H), 2.51 – 2.42 (m, 1H), 0.85 – 0.77 (m, 2H), 0.58 – 0.51 (m, 2H). **$^{13}C\{^1H\}$ NMR (101 MHz, $CDCl_3$):** δ = 151.9, 133.6, 120.4, 112.8, 99.4, 24.6, 7.6. **MS (EI):** m/z (%) = 158 (73), 157 (100), 142 (27), 131 (45), 130 (34), 129 (57), 102 (45).

4: *N*-cyclopropyl-4-(trifluoromethyl)aniline

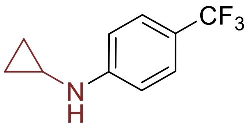

Amine **4** was synthesized according to general procedure *A* using the corresponding aryl chloride and cyclopropylamine. Purification by column chromatography (5% EtOAc/hexane) resulted in 65.7 mg of a yellow oil (33% yield). The analytical data is in accordance with the reported literature.[14] **^{1}H NMR (400 MHz, $CDCl_3$):** δ = 7.50 – 7.37 (m, 2H), 6.84 – 6.71 (m, 2H), 4.44 (s, 1H), 2.51 – 2.39 (m, 1H), 0.83 – 0.73 (m, 2H), 0.57 – 0.49 (m, 2H). **$^{13}C\{^1H\}$ NMR (101 MHz, $CDCl_3$):** δ = 151.3, 126.6 (q, $^2J_{CF}$ = 3.8 Hz), 119.5 (q, $^1J_{CF}$ = 32.5 Hz), 112.5, 25.0, 7.7. **MS (EI):** m/z (%) = 201 (100), 200 (100), 174 (35), 172 (67), 145 (74), 132 (32), 130 (48).

5: *N*-cyclopropyl-4-fluoroaniline

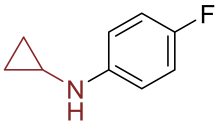

Amine **5** was synthesized according to general procedure *A* using the corresponding aryl chloride and cyclopropylamine. Purification by column chromatography (5% EtOAc/hexane) resulted in 28.8 mg of a colourless oil (19% yield). The analytical data is in accordance with the reported literature.[15] **^{1}H NMR (400 MHz, $CDCl_3$):** δ = 6.97 – 6.83 (m, 2H), 6.79 – 6.65 (m, 2H), 4.13 (s, 1H), 2.44 – 2.32 (m, 1H), 0.77 – 0.65 (m, 2H), 0.55 –

0.44 (m, 2H). **$^{13}C\{^1H\}$ NMR (101 MHz, $CDCl_3$):** δ = 156.6 (d, $^1J_{CF}$ = 234.9 Hz), 145.36 (d, $^4J_{CF}$ = 1.8 Hz), 116.0 (d, $^2J_{CF}$ = 22.3 Hz), 114.3 (d, $^3J_{CF}$ = 7.3 Hz), 26.2, 7.8. **MS (EI):** m/z (%) = 151 (88), 150 (100), 135 (39), 124 (33), 122 (80), 95 (55), 75 (28).

6: 4-(cyclopropylamino)-*N*,*N*-diisopropylbenzamide

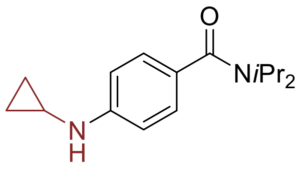

Amine **6** was synthesized according to general procedure *A* at 60 °C using the corresponding aryl chloride and cyclopropylamine. Purification by column chromatography (25% EtOAc/hexane) resulted in 153.4 mg of a yellow solid (59% yield). **^1H NMR (400 MHz, $CDCl_3$):** δ = 7.30 – 7.24 (m, 2H), 6.86 – 6.79 (m, 2H), 4.51 (s, 1H), 3.84 (s, 2H), 2.56 – 2.48 (m, 1H), 1.40 (s, 12H), 0.86 – 0.79 (m, 2H), 0.63 – 0.57 (m, 2H). **$^{13}C\{^1H\}$ NMR (101 MHz, $CDCl_3$):** δ = 171.8, 127.7, 114.3, 21.0, 149.3, 112.6, 25.2, 21.1, 7.6. **IR (cm^{-1}):** 3309 (m), 2968 (m), 1599 (s), 1522 (m), 1471 (w), 1444 (m), 1369 (m), 1341 (s), 1318 (m), 1299 (w), 1270 (w), 1211 (w), 1155 (w), 1099 (w), 1038 (m), 1020 (w), 949 (w), 826 (m), 814 (m), 772 (w), 629 (w), 605 (w), 572 (m), 519 (m). **M.p.**: 153-155 °C. **HRMS (ESI)** m/z: Calcd for $C_{16}H_{24}N_2O$ $[M]^+$ 260.1889; Found 260.1887. **MS (EI):** m/z (%) = 260 (13), 217 (14), 161 (13), 160 (100), 132 (7), 130 (6), 117 (11).

7: ethyl 4-(cyclopropylamino)benzoate

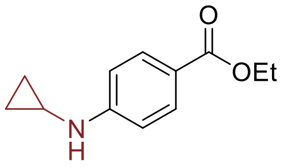

Amine **7** was synthesized according to general procedure *A* using the corresponding aryl chloride and cyclopropylamine. Purification by column chromatography (5% EtOAc/hexane) resulted in 127.0 mg of a yellow solid (62% yield). The analytical data is in accordance with the reported literature.[16] **^1H NMR (400 MHz, $CDCl_3$):** 7.92 – 7.84 (m, 2H), 6.78 – 6.70 (m, 2H), 4.62 (s, 1H), 4.32 (q, *J* = 7.1 Hz, 2H), 2.53 – 2.41 (m, 1H), 1.36 (t, *J* = 7.2 Hz, 3H), 0.86 – 0.73 (m, 2H), 0.59 – 0.50 (m, 2H). **$^{13}C\{^1H\}$ NMR (101 MHz, $CDCl_3$):** δ = 167.0, 152.5, 131.5, 119.5, 112.2, 60.3, 25.0, 14.6, 7.7. **MS (EI):** m/z (%) = 205 (70), 204 (42), 176 (65), 160 (94), 132 (100), 130 (43), 117 (34).

8: (4-(cyclopropylamino)phenyl)(phenyl)methanone

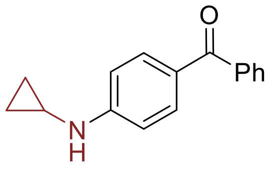

Amine **8** was synthesized according to general procedure *A* using the corresponding aryl chloride and cyclopropylamine. Purification by column chromatography (10% EtOAc/hexane) resulted in 230.8 mg of a yellow oil (97% yield). **^1H NMR (400 MHz, $CDCl_3$):** δ = 7.78 – 7.71 (m, 4H), 7.55 – 7.43 (m, 3H), 6.81 – 6.74 (m, 2H), 4.64 (s, 1H), 2.57 – 2.46 (m, 1H), 0.85 – 0.77 (m, 2H), 0.61 – 0.52 (m, 2H). **$^{13}C\{^1H\}$ NMR (101 MHz, $CDCl_3$):** δ = 195.7, 153.1, 139.6, 133.2, 131.7, 130.0, 128.5, 127.2, 112.4, 25.2, 8.1. **IR (cm^{-1}):** 3334 (w), 2980 (w), 1636 (w), 1587 (s), 1520 (m), 1446 (m), 1418 (w), 1311 (s), 1276 (s), 1173 (m), 1146 (s), 1025 (w), 937 (w), 919 (m), 837 (m), 792 (m), 741 (m), 699 (s), 624 (m). **HRMS (ESI):** m/z: Calcd for $C_{16}H_{15}NO$ $[M]^+$ 237.1153; Found 237.1151. **MS (EI):** m/z (%) = 237 (100), 236 (34), 160 (95), 132 (64), 117 (26), 105 (82), 77 (75).

9: 4-chloro-*N*-cyclopropylaniline

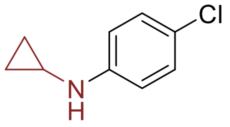

Amine **9** was synthesized according to general procedure *A* using the corresponding aryl bromide and cyclopropylamine. Purification by column chromatography (10% EtOAc/hexane) resulted in 128.8 mg of a yellow oil (77% yield). The analytical data is in accordance with the reported literature.[17] **^{1}H NMR (400 MHz, $CDCl_3$):** δ = 7.17 – 7.09 (m, 2H), 6.74 – 6.67 (m, 2H), 4.18 (s, 1H), 2.44 – 2.36 (m, 1H), 0.76 – 0.70 (m, 2H), 0.53 – 0.48 (m, 2H). **$^{13}C\{^1H\}$ NMR (101 MHz, $CDCl_3$):** δ = 147.3, 129.0, 122.5, 114.3, 25.4, 7.6. **MS (EI):** m/z (%) = 167 (87), 166 (86), 140 (61), 138 (90), 132,1 (100), 130 (99), 111 (51).

10: 3-chloro-*N*-cyclopropylaniline

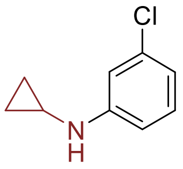

Amine **10** was synthesized according to general procedure *A* using the corresponding aryl chloride and cyclopropylamine. Purification by column chromatography (3% EtOAc/hexane) resulted in 109.6 mg of a colorless oil (65% yield). The analytical data is in accordance with the reported literature.[17] **^{1}H NMR (400 MHz, $CDCl_3$):** δ = 7.08 (t, *J* = 8.0 Hz, 1H), 6.79 (t, *J* = 2.1 Hz, 1H), 6.69 (dd, *J* = 7.8, 1.1 Hz, 1H), 6.61 (dd, *J* = 8.2, 1.3 Hz, 1H), 4.26 (s, 1H), 2.46 – 2.35 (m, 1H), 0.80 – 0.71 (m, 2H), 0.55 – 0.48 (m, 2H). **$^{13}C\{^1H\}$ NMR (101 MHz, $CDCl_3$):** δ = 150.0, 135.0, 130.2, 117.7, 112.9, 111.7, 25.2, 7.6. **MS (EI):** m/z (%) = 167 (89), 166 (100), 140 (55), 138 (70), 132 (70), 131 (57), 130 (84).

11: 2-chloro-*N*-cyclopropylaniline

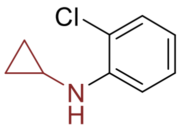

Amine **11** was synthesized according to general procedure *A* at 100 °C using the corresponding aryl chloride and cyclopropylamine. Purification by column chromatography (0-3% EtOAc/hexane) resulted in 17.7 mg of a colorless oil (11% yield). The analytical data is in accordance with the reported literature.[18] **^{1}H NMR (400 MHz, $CDCl_3$):** δ = 7.23 (dd, *J* = 7.9, 1.5 Hz, 1H), 7.19 – 7.15 (m, 1H), 7.08 (dd, *J* = 8.2, 1.6 Hz, 1H), 6.66 (td, *J* = 7.6, 1.6 Hz, 1H), 4.72 (s, 1H), 2.50 – 2.41 (m, 1H), 0.81 – 0.75 (m, 2H), 0.60 – 0.55 (m, 2H). **$^{13}C\{^1H\}$ NMR (101 MHz, $CDCl_3$):** δ = 144.8, 129.1, 127.8, 118.9, 117.8, 112.7, 25.1, 7.6. **MS (EI):** m/z (%) = 167 (24), 166 (19), 140 (23), 138 (32), 132 (100), 131 (17), 130 (44).

12: *N*-cyclopropyl-2-methylaniline

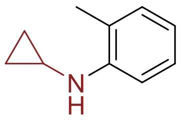

Amine **12** was synthesized according to general procedure *A* using the corresponding aryl chloride and cyclopropylamine. Purification by column chromatography (5% EtOAc/hexane) resulted in 104.5 mg of a colorless oil (71% yield). The analytical data is in accordance with the reported literature.[19] **^{1}H NMR (400 MHz, $CDCl_3$):** δ = 7.21 – 7.14 (m, 1H), 7.12 – 7.01 (m, 2H), 6.77 – 6.66 (m, 1H), 4.05 (s, 1H), 2.51 – 2.41 (m, 1H), 2.11 (s, 3H), 0.82 – 0.72 (m, 2H), 0.60 – 0.52 (m, 2H). **$^{13}C\{^1H\}$ NMR (101 MHz, $CDCl_3$):** δ = 147.1, 130.4, 127.4, 122.1, 117.8, 111.5, 25.7, 17.8, 8.0. **MS (EI):** m/z (%) = 147 (38), 146 (25), 132 (31), 130 (59), 118 (100), 117 (23), 91 (49).

13: *N*-cyclopropylnaphthalen-1-amine

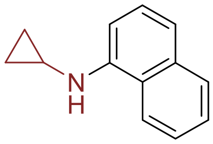

Amine **13** was synthesized according to general procedure *A* using the corresponding aryl chloride and cyclopropylamine. Purification by column chromatography (5% EtOAc/hexane) resulted in 127.8 mg of a red oil (70% yield). The analytical data is in accordance with the reported literature.[13] **^{1}H NMR (400 MHz, $CDCl_3$):** δ = 7.75 – 7.65 (m, 1H), 7.65 – 7.59 (m, 1H), 7.38 – 7.25 (m, 3H), 7.22 – 7.16 (m, 1H), 7.02 – 6.94 (m, 1H), 4.81 (s, 1H), 2.54 – 2.41 (m, 1H), 0.80 – 0.66 (m, 2H), 0.62 – 0.48 (m, 2H). **$^{13}C\{^1H\}$ NMR (101 MHz, $CDCl_3$):** δ = 143.6, 134.0, 128.5, 126.4, 125.4, 124.5, 123.0, 119.5, 117.6, 105.7, 25.3, 7.3. **MS (EI):** m/z (%) = 183 (100), 182 (79), 167 (71), 165 (76), 154 (92), 127 (89), 115 (56).

14: 4-(*tert*-butyl)-*N*-cyclopropylaniline

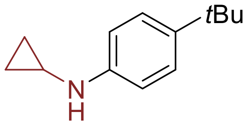

Amine **14** was synthesized according to general procedure *A* using the corresponding aryl chloride and cyclopropylamine. Purification by column chromatography (5% EtOAc/hexane) resulted in 72.2 mg of a red oil (38% yield). The analytical data is in accordance with the reported literature.[20] **^{1}H NMR (400 MHz, $CDCl_3$):** δ = 7.26 – 7.18 (m, 2H), 6.82 – 6.74 (m, 2H), 4.14 (s, 1H), 2.47 – 2.37 (m, 1H), 1.30 (s, 9H), 0.75 – 0.68 (m, 2H), 0.55 – 0.49 (m, 2H). **$^{13}C\{^1H\}$ NMR (101 MHz, $CDCl_3$):** δ = 146.4, 126.0, 117.5, 113.0, 34.0, 31.7, 25.5, 7.5. **MS (EI):** m/z (%) = 189 (33), 188 (15), 175 (16), 174 (100), 132 (71), 117 (14), 91 (16).

15: *N*-(4-(tert-butyl)phenyl)-*N*-cyclopropylacetamide

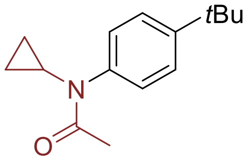

Amide **15** was synthesized according to general procedure *B* using the corresponding aryl chloride and cyclopropylamine. Purification by column chromatography (45% EtOAc/hexane) resulted in 205.0 mg of a yellow oil (88% yield). **^{1}H NMR (400 MHz, $CDCl_3$):** δ = 7.41 – 7.33 (m, 2H), 7.03 – 6.95 (m, 2H), 3.22 – 3.11 (m, 1H), 1.97 (s, 3H), 1.35 – 1.30 (m, 9H), 0.81 – 0.74 (m, 2H), 0.52 – 0.44 (m, 2H). **$^{13}C\{^1H\}$ NMR (101 MHz, $CDCl_3$):** δ = 172.8, 150.9, 140.0, 127.9, 126.5, 35.1, 31.9, 31.6, 23.9, 8.1. ^{1}H and $^{13}C\{^1H\}$ NMR spectra were measured at 60 °C to resolve peaks. **IR (cm^{-1}):** 2980 (m), 2964 (m), 1667 (s), 1606 (w), 1509 (s), 1458 (w), 1375 (s), 1329 (s), 1306 (m), 1268 (m), 1204 (w), 1028 (m), 964 (w), 833 (m), 588 (s). **HRMS (ESI)** m/z: Calcd for $C_{15}H_{21}NO$ $[M]^+$ 231.1623; Found 231.1624. **MS (EI):** m/z (%) = 231 (15), 216 (27), 188 (64), 174 (100), 157 (31), 132 (81), 117 (18), 91 (17).

16: *N*-cyclopropyl-*N*-(4-methoxyphenyl)acetamide

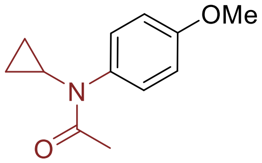

Amide **16** was synthesized according to general procedure *B* using the corresponding aryl chloride and cyclopropylamine. Purification by column chromatography (65% EtOAc/hexane) resulted in 79.9 mg of a yellow oil (39% yield). The analytical data is in accordance with the reported literature.[19] **^{1}H NMR (400 MHz, $CDCl_3$):** δ = 7.03 – 6.92 (m, 2H), 6.93 – 6.84 (m, 2H), 3.82 (s, 3H), 3.24 – 3.10 (m, 1H), 1.94 (s, 3H), 0.82 – 0.70 (m, 2H), 0.54 – 0.39 (m, 2H). **$^{13}C\{^1H\}$ NMR (101 MHz, $CDCl_3$):** δ = 172.9, 159.3, 135.5, 129.6, 115.1, 56.0, 31.7, 23.8, 7.8. ^{1}H and $^{13}C\{^1H\}$ NMR spectra were measured at 60 °C to resolve peaks. **MS (EI):** m/z (%) = 205 (15), 163 (25), 162 (100), 148 (39), 146 (38), 134 (45), 121 (23), 77 (21).

17: *N*-cyclopropyl-*N*-(4-(trimethylsilyl)phenyl)acetamide

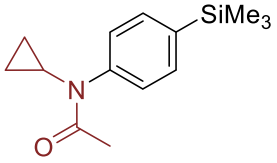

Amide **17** was synthesized according to general procedure *B* using the corresponding aryl chloride and cyclopropylamine. Purification by column chromatography (55% EtOAc/hexane) resulted in 190.7 mg of a yellow oil (77% yield). **^{1}H NMR (400 MHz, CDCl$_3$):** δ = 7.55 – 7.45 (m, 2H), 7.11 – 7.00 (m, 2H), 3.23 – 3.09 (m, 1H), 2.00 (s, 3H), 0.85 – 0.74 (m, 2H), 0.55 – 0.43 (m, 2H), 0.28 (s, 9H). **^{13}C{^{1}H} NMR (101 MHz, CDCl$_3$):** δ = 172.3, 142.9, 139.8, 134.3, 127.2, 31.3, 23.6, 8.2, -1.0. ^{1}H and ^{13}C{^{1}H} NMR spectra were measured at 60 °C to resolve peaks. **IR (cm^{-1}):** 2981 (s), 2889 (w), 1667 (s), 1592 (m), 1499 (w), 1375 (s), 1327 (s), 1302 (m), 1248 (s), 1155 (w), 1109 (m), 951 (w), 836 (s), 756 (m), 693 (w), 565 (m). **HRMS (ESI)** m/z: Calcd for $C_{14}H_{21}NOSi$ [M]$^+$ 247.1392; Found 247.1391. **MS (EI):** 247 (13), 232 (35), 205 (25), 204 (73), 190 (100), 173 (23), 132 (47), 73 (67).

18: *N*-cyclopropyl-*N*-(4-vinylphenyl)acetamide

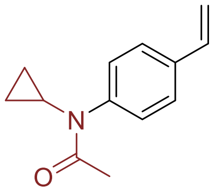

Amide **18** was synthesized according to general procedure *B* using the corresponding aryl chloride and cyclopropylamine. Purification by column chromatography (50% EtOAc/hexane) resulted in 136.2 mg of a yellow oil (50% yield). **^{1}H NMR (400 MHz, DMSO):** δ = 7.50 – 7.44 (m, 2H), 7.18 – 7.13 (m, 2H), 6.79 – 6.69 (m, 1H), 5.80 (dd, *J* = 17.6, 1.0 Hz, 1H), 5.27 (dd, *J* = 10.9, 1.0 Hz, 1H), 3.16 – 3.15 (m, 1H), 2.01 (s, 3H), 0.81 – 0.75 (m, 2H), 0.48 – 0.41 (m, 2H). **^{13}C{^{1}H} NMR (101 MHz, DMSO):** δ = 170.8, 141.7, 135.7, 135.2, 127.4, 126.2, 114.1, 30.6, 22.9, 8.1. ^{1}H and ^{13}C{^{1}H} NMR spectra were measured at 60 °C to resolve peaks. **IR (cm^{-1}):** 2980 (s), 1661 (s), 1602 (w), 1509 (m), 1374 (s), 1329 (s), 1299 (m), 1163 (w), 905 (m), 841 (m), 651 (w). 587 (m) 499 (w). **HRMS (ESI)** m/z: Calcd for $C_{13}H_{15}NO$ [M]$^+$ 201.1154; Found 201.1154. **MS (EI):** m/z (%) = 201 (10), 159 (30), 158 (100), 142 (39), 132 (22), 130 (40), 103 (28), 77 (33).

19: *N*-(benzo[*d*][1,3]dioxol-5-yl)-*N*-cyclopropylacetamide

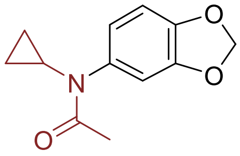

Amide **19** was synthesized according to general procedure *B* at 60 °C using the corresponding aryl chloride and cyclopropylamine. Purification by column chromatography (60% EtOAc/hexane) resulted in 83.1 mg of a yellow oil (38% yield). The analytical data is in accordance with the reported literature.[19] **^{1}H NMR (400 MHz, CDCl$_3$):** δ = 7.08 – 7.01 (m, 1H), 6.59 – 6.47 (m, 2H), 5.99 (s, 2H), 3.18 – 3.04 (m, 1H), 1.98 (s, 3H), 0.83 – 0.72 (m, 2H), 0.54 – 0.44 (m, 2H). **^{13}C{^{1}H} NMR (101 MHz, CDCl$_3$):** δ = 171.3, 148.4, 147.0, 136.3, 121.7, 109.2, 108.4, 101.8, 31.4, 23.4, 7.3. ^{1}H and ^{13}C{^{1}H} NMR spectra were measured at 60 °C to resolve peaks. **MS (EI):** m/z (%) = 219 (28), 177 (34), 176 (100), 160 (35), 148 (50), 146 (49), 118 (32).

20: *N*-cyclopropyl-*N*-(2-(methoxymethyl)phenyl)acetamide

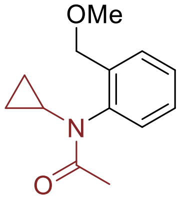

Amide **20** was synthesized according to general procedure *B* at 60 °C using the corresponding aryl bromide and cyclopropylamine. Purification by column chromatography (60% EtOAc/hexane) resulted in 157.9 mg of a yellow oil (79% yield). **^{1}H NMR (400 MHz, DMSO):** δ = 7.51 – 7.40 (m, 1H), 7.40 – 7.29 (m, 2H), 7.11 – 7.05 (m, 1H), 4.27 (s, 2H), 3.31 (s, 3H), 3.22 – 3.14 (m, 1H), 3.01 (s, 3H), 0.72 (s, 2H), 0.43 (s, 2H). ^{1}H spectrum was measured at 90 °C to resolve peaks. ^{13}C{^{1}H} NMR spectrum could not be resolved. **IR (cm^{-1}):** 2980 (m), 2889 (w), 1661 (s), 1601 (w), 1491 (m), 1453 (m), 1378 (s), 1331 (s), 1194 (m), 1156 (w), 1091 (s), 961 (m), 825 (w), 762 (m), 592 (m). **HRMS (ESI)** m/z: Calcd for $C_{13}H_{17}NO_2$ $[M]^+$ 219.1259; Found 219.1255. **MS (EI):** m/z (%) = 204 (19), 176 (24), 145 (35), 144 (100), 130 (42), 128 (27), 118 (32), 117 (65), 91 (26).

21: *N*-cyclopropylpyridin-3-amine

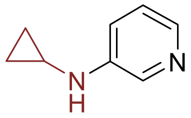

Amine **21** was synthesized according to general procedure *A* using the corresponding aryl chloride and cyclopropylamine. Purification by column chromatography (35% EtOAc/hexane) resulted in 73.0 mg of a colorless solid (54% yield). The analytical data is in accordance with the reported literature.[13] **^{1}H NMR (400 MHz, CDCl$_3$):** δ = 8.18 – 8.12 (m, 1H), 8.02 – 7.95 (m, 1H), 7.13 – 7.06 (m, 2H), 4.22 (s, 1H), 2.47 – 2.39 (m, 1H), 0.79 – 0.74 (m, 2H), 0.55 – 0.50 (m, 2H). **^{13}C{^{1}H} NMR (101 MHz, CDCl$_3$):** δ = 144.8, 139.2, 136.2, 123.8, 119.4, 25.0, 7.6. **MS (EI):** m/z (%) = 134 (54), 133 (100), 132 (27), 107 (34), 106 (30), 105 (32), 78 (48).

22: *N*-cyclopropylpyridin-2-amine

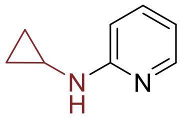

Amine **22** was synthesized according to general procedure *A* using the corresponding aryl chloride and cyclopropylamine. Purification by column chromatography (25% EtOAc/hexane) resulted in 123.0 mg of a yellow solid (92% yield). The analytical data is in accordance with the reported literature.[21] **^{1}H NMR (400 MHz, CDCl$_3$):** δ = 1H NMR (400 MHz, CDCl3) δ 8.10 – 8.03 (m, 1H), 7.52 – 7.46 (m, 1H), 6.78 – 6.71 (m, 1H), 6.65 – 6.59 (m, 1H), 5.09 (s, 1H), 2.53 – 2.47 (m, 1H), 0.81 – 0.75 (m, 2H), 0.58 – 0.53 (m, 2H). **^{13}C{^{1}H} NMR (101 MHz, CDCl$_3$):** δ = 159.5, 147.9, 137.5, 113.3, 106.1, 23.8, 7.4. **MS (EI):** m/z (%) = 134 (55), 133 (56), 119 (100), 79 (43), 78 (53), 52 (23), 51 (20).

23: *N*-cyclopropyl-6-methoxypyridin-2-amine

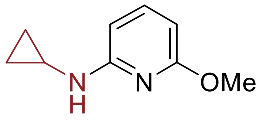

Amine **23** was synthesized according to general procedure *A* using the corresponding aryl chloride and cyclopropylamine. Purification by column chromatography (5% EtOAc/hexane) resulted in 143.3 mg of a yellow solid (87% yield). The analytical data is in accordance with the reported literature.[19] **^{1}H NMR (400 MHz, CDCl$_3$):** δ = 7.26 (t, *J* = 7.9 Hz, 1H), 6.14 (d, *J* = 7.9 Hz, 1H), 5.94 (d, *J* = 8.0 Hz, 1H), 4.70 (s, 1H), 3.67 (s, 3H), 2.37 – 2.28 (m, 1H), 0.63 – 0.56 (m, 2H), 0.44 – 0.37 (m, 2H). **^{13}C{^{1}H} NMR (101 MHz, CDCl$_3$):** δ = 163.7, 158.8, 140.3, 98.6, 97.4, 53.2, 24.3, 7.8, 7.6. **MS (EI):** m/z (%) = 164 (69), 163 (23), 149 (100), 136 (22), 134 (53), 109 (20), 93 (23).

24: *N*-cyclopropylpyrazin-2-amine

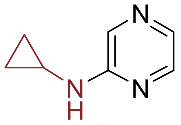

Amine **24** was synthesized according to general procedure *A* using the corresponding aryl chloride and cyclopropylamine. Purification by column chromatography (35% EtOAc/hexane) resulted in 78.9 mg of an orange solid (59% yield). The analytical data is in accordance with the reported literature.[19] **^{1}H NMR (400 MHz, $CDCl_3$):** δ = 8.21 (s, 1H), 7.98 (s, 1H), 7.90 (s, 1H), 5.09 (s, 1H), 2.64 – 2.55 (m, 1H), 0.90 – 0.82 (m, 2H), 0.64 – 0.56 (m, 2H). **^{13}C{^{1}H} NMR (101 MHz, $CDCl_3$):** δ = 155.4, 142.2, 134.2, 131.0, 23.8, 8.0. **MS (EI):** m/z (%) = 135 (57), 134 (38), 120 (100), 107 (20), 80 (21), 79 (23), 52 (24).

25: *N*-cyclopropylquinolin-2-amine

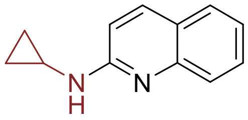

Amine **25** was synthesized according to general procedure *A* using the corresponding aryl chloride and cyclopropylamine. Purification by column chromatography (15% EtOAc/hexane) resulted in 153.9 mg of a yellow solid (84% yield). **^{1}H NMR (400 MHz, $CDCl_3$):** δ = 7.94 (d, *J* = 8.9 Hz, 1H), 7.68 – 7.58 (m, 2H), 7.58 – 7.51 (m, 1H), 7.26 – 7.19 (m, 1H), 7.09 (d, *J* = 8.9 Hz, 1H), 5.40 (s, 1H), 2.73 – 2.62 (m, 1H), 0.92 – 0.79 (m, 2H), 0.66 – 0.55 (m, 2H). **^{13}C{^{1}H} NMR (101 MHz, $CDCl_3$):** δ = 158.8, 148.2, 138.4, 130.2, 128.0, 126.3, 124.2, 122.7, 110.1, 24.5, 8.5. **IR (cm^{-1}):** 3220 (w), 2969 (w), 1609 (s), 1572 (m), 1505 (s), 1446 (w), 1422 (m), 1347 (s), 1141 (m), 1120 (w), 1049 (w), 1017 (m), 978 (w), 941 (w), 906 (w), 840 (m), 816 (s), 782 (m), 755 (s), 686 (m), 615 (m), 564 (m), 473 (m). **M.p.**: 118-120 °C. **HRMS (ESI)** m/z: Calcd for $C_{12}H_{12}N_2$ $[M]^+$ 184.1001; Found 184.0997. **MS (EI):** m/z (%) = 184 (61), 183 (49), 169 (100), 156 (23), 129 (38), 128 (48), 101 (17).

26: *N*-cyclopropyl-2-methylbenzo[*d*]oxazol-5-amine

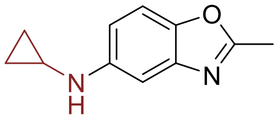

Amine **26** was synthesized according to general procedure *A* at 60 °C using the corresponding aryl chloride and cyclopropylamine. Purification by column chromatography (15% EtOAc/hexane) resulted in 63.7 mg of a yellow solid (44% yield). **^{1}H NMR (400 MHz, $CDCl_3$):** δ = 7.28 – 7.25 (m, 1H), 7.16 – 7.11 (m, 1H), 6.73 – 6.69 (m, 1H), 4.31 (s, 1H), 2.61 (s, 3H), 2.52 – 2.42 (m, 1H), 0.80 – 0.75 (m, 2H), 0.58 – 0.53 (m, 2H). **^{13}C{^{1}H} NMR (101 MHz, $CDCl_3$):** δ = 164.2, 146.3, 144.7, 142.7, 111.5, 110.2, 102.5, 26.1, 14.7, 7.5. **IR (cm^{-1}):** 3298 (m), 2952 (w), 1621 (m), 1571 (s), 1483 (s), 1448 (m), 1363 (s), 1337 (m), 1272 (m), 1220 (w), 1178 (s), 1151 (m), 1039 (w), 1021 (m), 926 (m), 832 (s), 800 (s), 663 (s), 623 (m), 436 (m). **M.p.**: 51-53 °C. **HRMS (ESI)** m/z: Calcd for $C_{11}H_{12}N_2O$ $[M]^+$ 188.0950; Found 188.0948. **MS (EI):** m/z (%) = 188 (100), 187 (93), 159 (81), 146 (60), 132 (47), 118 (39), 91 (35).

27: (4-(cyclopropylamino)phenyl)(phenyl)methanone

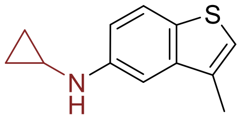

Amine **27** was synthesized according to general procedure *A* using the corresponding aryl chloride and cyclopropylamine. Purification by column chromatography (5% EtOAc/hexane) resulted in 117.0 mg of a yellow oil (58% yield). **^{1}H NMR (400 MHz, $CDCl_3$):** δ = 7.61 (d, *J* = 8.6 Hz, 1H), 7.11 (d, *J* = 2.2 Hz, 1H), 7.02 (d, *J* = 1.6 Hz, 1H), 6.84 (dd, *J* = 8.6, 2.2 Hz, 1H), 4.31 (s, 1H), 2.55 – 2.47 (m, 1H), 2.39 (d, *J* = 1.2 Hz, 3H), 0.82 – 0.76 (m, 2H), 0.61 – 0.54 (m, 2H). **^{13}C{^{1}H} NMR (101 MHz, $CDCl_3$):** δ = 146.2, 141.0, 131.6, 123.2, 122.2,

113.5, 104.2, 33.7, 25.8, 14.2, 10.0, 7.6. **IR (cm^{-1}):** 3312 (w), 3083 (w), 2913 (w), 1661 (w), 1602 (s), 1523 (m), 1444 (s), 1362 (m), 1296 (w), 1267 (m), 1169 (w), 1019 (m), 833 (s), 804 (s), 765 (s), 723 (m), 636 (m). **HRMS (ESI)** m/z: Calcd for $C_{12}H_{13}NS$ [M]$^+$ 203.0769; 203.0768. **MS (EI):** m/z (%) = 203 (100), 202 (70), 187 (27), 186 (30), 174 (54), 161 (19), 147 (62).

28: 1-(4-(cyclopropylamino)phenyl)ethan-1-one

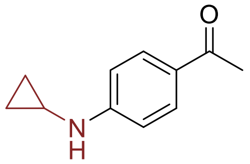

Amine **28** was synthesized according to general procedure *A* using the corresponding aryl chloride and cyclopropylamine. Purification by column chromatography (20% EtOAc/hexane) resulted in 67.9 mg of a white-yellow solid (39% yield). The analytical data is in accordance with the reported literature.[22] **^{1}H NMR (400 MHz, CDCl$_3$):** δ = 7.86 – 7.81 (m, 2H), 6.78 – 6.72 (m, 2H), 4.68 (s, 1H), 2.53 – 2.48 (m, 4H), 0.84 – 0.78 (m, 2H), 0.58 – 0.53 (m, 2H). **^{13}C{^{1}H} NMR (101 MHz, CDCl$_3$):** δ = 196.6, 152.8, 130.7, 127.5, 112.2, 26.2, 25.0, 7.8. **MS (EI):** m/z (%) = 175 (57), 174 (21), 160 (100), 146 (17), 132 (52), 130 (23), 117 (32).

29: diethyl (4-(cyclopropylamino)benzyl)phosphonate

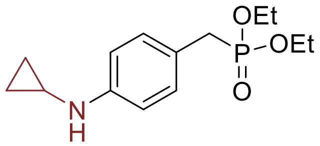

Amine **29** was synthesized according to general procedure *A* using the corresponding aryl chloride and cyclopropylamine. Purification by column chromatography (60% EtOAc/hexane) resulted in 185.8 mg of a yellow wax (66% yield). **^{1}H NMR (400 MHz, CDCl$_3$):** δ = 7.16 – 7.06 (m, 2H), 6.79 – 6.67 (m, 2H), 4.17 – 4.09 (m, 1H), 4.08 – 3.91 (m, 4H), 3.07 (s, 1H), 3.02 (s, 1H), 2.45 – 2.33 (m, 1H), 1.28 – 1.21 (m, 6H), 0.78 – 0.67 (m, 2H), 0.54 – 0.46 (m, 2H). **^{13}C{^{1}H} NMR (101 MHz, CDCl$_3$):** δ = 147.7 (d, J_{CP} = 3.2 Hz), 130.6 (d, J_{CP} = 6.6 Hz), 120.3 (d, J_{CP} = 9.3 Hz), 113.5 (d, J_{CP} = 2.9 Hz), 62.1 (d, J_{CP} = 6.8 Hz), 32.2, 25.5, 16.6 (d, J_{CP} = 5.9 Hz), 7.5. **IR (cm^{-1}):** 3315 (w), 2980 (w), 1615 (m), 1519 (s), 1365 (w), 1317 (w), 1234 (m), 1056 (s), 967 (m), 850 (w). **HRMS (ESI)** m/z: Calcd for $C_{14}H_{22}NO_3P$ [M]$^+$ 283.1337; Found 283.1332. **MS (EI):** m/z (%) = 283 (18), 256 (9), 254 (11), 147 (11), 146 (100), 145 (31), 144 (20), 132 (14), 91 (11), 90 (10).

30: isopropyl 2-(4-(4-(cyclopropylamino)benzoyl)phenoxy)-2-methylpropanoate

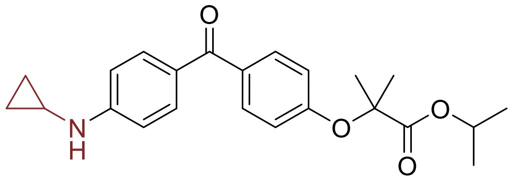

Amine **30** was synthesized according to general procedure *A* using the corresponding aryl chloride and cyclopropylamine. Purification by column chromatography (40% EtOAc/hexane) resulted in 67.9 mg of a yellow wax (83% yield). **^{1}H NMR (400 MHz, CDCl$_3$):** δ = 7.77 – 7.65 (m, 4H), 6.90 – 6.81 (m, 2H), 6.81 – 6.73 (m, 2H), 5.09 (hept, J = 6.3 Hz, 1H), 4.58 (s, 1H), 2.56 – 2.46 (m, 1H), 1.65 (s, 6H), 1.21 (d, J = 6.3 Hz, 6H), 0.88 – 0.77 (m, 2H), 0.61 – 0.53 (m, 2H). **^{13}C{^{1}H} NMR (101 MHz, CDCl$_3$):** δ = 194.4, 173.5, 158.7, 152.4, 132.7, 132.3, 131.6, 127.3, 117.4, 112.1, 79.4, 69.4, 25.5, 25.0, 21.7, 7.8. **IR (cm^{-1}):** 3348 (w), 2980 (w), 1727 (m), 1637 (w), 1592 (s), 1522 (w), 1312 (m), 1278 (s), 1243 (s), 1168 (m), 1144 (s), 1099 (s), 925 (m), 847 (w), 767 (m), 687 (w), 621 (m). **HRMS (ESI)** m/z: Calcd for $C_{23}H_{27}NO_4$ [M]$^+$ 381.1940; Found 381.1936. **MS (EI):** m/z (%) = 382 (17), 381 (67), 295 (16), 294 (75), 254 (18), 253 (99), 252 (96), 226 (19), 225 (15), 224 (44), 160 (91), 133 (17), 121 (89).

31: *N*-cyclobutyl-4-methylaniline

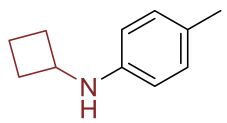

Amine **31** was synthesized according to general procedure *A* using the corresponding aryl chloride and cyclobutylamine. Purification by column chromatography (7% EtOAc/hexane) resulted in 146.1 mg of a yellow oil (91% yield). The analytical data is in accordance with the reported literature.[23] **^{1}H NMR (400 MHz, CDCl$_3$):** δ = 7.01 – 6.95 (m, 2H), 6.52 – 6.46 (m, 2H), 3.95 – 3.85 (m, 1H), 3.67 (s, 1H), 2.47 – 2.37 (m, 2H), 2.24 (s, 3H), 1.83 – 1.79 (m, 3H). **^{13}C{^{1}H} NMR (101 MHz, CDCl$_3$)** δ = 145.1, 129.9, 126.7, 113.3, 49.4, 31.4, 20.5, 15.4. **MS (EI):** m/z (%) = 161 (36), 133 (100), 132 (81), 118 (43), 117 (17), 106,1 (15), 91 (32).

32: *N*-cyclobutylnaphthalen-2-amine

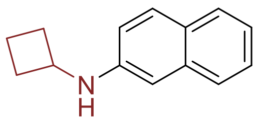

Amine **32** was synthesized according to general procedure *A* using the corresponding aryl chloride and cyclobutylamine. Purification by column chromatography (8% EtOAc/hexane) resulted in 186.8 mg of a red oil (95% yield). The analytical data is in accordance with the reported literature.[24] **^{1}H NMR (400 MHz, CDCl$_3$):** δ = 7.67 – 7.59 (m, 3H), 7.37 – 7.34 (m, 1H), 7.21 – 7.17 (m, 1H), 6.85 (dd, *J* = 8.8, 2.3 Hz, 1H), 6.74 (d, *J* = 2.3 Hz, 1H), 4.08 – 4.02 (m, 2H), 2.54 – 2.49 (m, 2H), 1.91 – 1.85 (m, 4H). **^{13}C{^{1}H} NMR (101 MHz, CDCl$_3$):** δ = 144.9, 135.3, 129.1, 127.8, 127.6, 126.4, 126.0, 122.1, 118.1, 105.1, 49.2, 31.3, 15.5. **MS (EI):** m/z (%) = 197 (45), 169 (100), 168 (92), 167 (25), 141 (28), 127 (22), 115 (23).

33: *N*-cyclobutylpyridin-3-amine

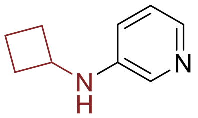

Amine **33** was synthesized according to general procedure *A* using the corresponding aryl chloride and cyclobutylamine. Purification by column chromatography (70% EtOAc/hexane) resulted in 103.0 mg of a yellow solid (70% yield). The analytical data is in accordance with the reported literature.[25] **^{1}H NMR (400 MHz, CDCl$_3$):** δ = 8.02 – 7.88 (m, 2H), 7.08 – 7.03 (m, 1H), 6.82 – 6.77 (m, 1H), 4.01 – 3.76 (m, 2H), 2.48 – 2.39 (m, 2H), 1.89 – 1.77 (m, 4H). **^{13}C{^{1}H} NMR (101 MHz, CDCl$_3$):** δ = 143.3, 139.0, 136.3, 123.8, 118.9, 48.8, 31.2, 15.3. **MS (EI):** m/z (%) = 148 (27), 120 (96), 119 (100), 93 (26), 78 (14), 67 (15), 51 (10).

34: *N*-cyclobutyl-3-methylbenzo[*b*]thiophen-5-amine

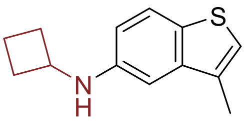

Amine **34** was synthesized according to general procedure *A* using the corresponding aryl chloride and cyclobutylamine. Purification by column chromatography (8% EtOAc/hexane) resulted in 188.6 mg of a yellow oil (87% yield). **^{1}H NMR (400 MHz, CDCl$_3$):** δ = 7.59 (d, *J* = 8.6 Hz, 1H), 7.03 – 6.98 (m, 1H), 6.79 (d, *J* = 2.3 Hz, 1H), 6.69 (dd, *J* = 8.6, 2.3 Hz, 1H), 4.07 – 3.98 (m, 1H), 3.94 (d, *J* = 30.5 Hz, 1H), 2.53 – 2.45 (m, 2H), 2.36 (d, *J* = 1.2 Hz, 3H), 1.92 – 1.80 (m, 4H). **^{13}C{^{1}H} NMR (101 MHz, CDCl$_3$):** δ = 144.7, 141.1, 131.5, 129.9, 123.3, 122.2, 113.7, 104.0, 49.6, 31.4, 15.5, 14.1. **IR (cm^{-1}):** 3393 (w), 2971 (m), 2932 (m), 1604 (s), 1568 (m), 1488 (m), 1451 (s), 1341 (m), 1269 (s) 1161 (m), 983 (w), 835 (m), 722 (w). **HRMS (ESI)** m/z: Calcd for $C_{13}H_{15}NS$ $[M]^+$ 217.0925; Found 217.0928. **MS (EI):** m/z (%) = 217 (45), 190 (17), 189 (100), 188 (67), 173 (27), 161 (21), 147 (16).

35: 2-chloro-*N*-cyclobutylaniline

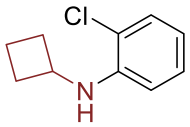

Amine **35** was synthesized according to general procedure *A* at 100 °C using the corresponding aryl chloride and cyclobutylamine. Purification by column chromatography (3% EtOAc/hexane) resulted in 88.8 mg of a colorless oil (49% yield). **^{1}H NMR (400 MHz, $CDCl_3$):** δ = 7.26 – 7.21 (m, 1H), 7.15 – 7.08 (m, 1H), 6.65 – 6.53 (m, 2H), 4.42 (s, 1H), 4.00 – 3.89 (m, 1H), 2.51 – 2.42 (m, 2H), 1.96 – 1.78 (m, 4H). **^{13}C{^{1}H} NMR (101 MHz, $CDCl_3$):** δ = 143.2, 129.2, 127.9, 119.0, 117.3, 111.8, 48.8, 31.2, 15.5. **IR (cm^{-1}):** 3412 (w), 2980 (s), 2889 (m), 1598 (s), 1502 (s), 1457 (m), 1382 (w), 1348 (w), 1321 (s), 1256 (m), 1167 (m), 1033 (m), 743 (s). **HRMS (ESI)** m/z: Calcd for $C_{10}H_{12}ClN$ $[M]^+$ 181.0658; Found 181.0661. **MS (EI):** m/z (%) = 181 (30), 155 (33), 153 (99), 152 (54), 118 (100), 117 (36), 91 (38).

36: *N*-cyclopentyl-4-fluoroaniline

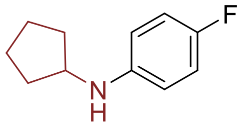

Amine **36** was synthesized according to general procedure *A* using the corresponding aryl chloride and cyclopentylamine. Purification by column chromatography (8% EtOAc/hexane) resulted in 91.4 mg of a yellow oil (51% yield). The analytical data is in accordance with the reported literature.[26] **^{1}H NMR (400 MHz, $CDCl_3$):** δ = 6.91 – 6.83 (m, 2H), 6.56 – 6.50 (m, 2H), 3.76 – 3.68 (m, 1H), 3.50 (s, 1H), 2.06 – 1.96 (m, 2H), 1.76 – 1.59 (m, 4H), 1.49 – 1.41 (m, 2H). **^{13}C{^{1}H} NMR (101 MHz, $CDCl_3$):** δ = 155.7 (d, $^{1}J_{CF}$ = 234.4 Hz), 144.6 (d, $^{4}J_{CF}$ = 1.8 Hz), 115.7 (d, $^{2}J_{CF}$ = 22.3 Hz), 114.1 (d, $^{3}J_{CF}$ = 7.3 Hz), 55.4, 33.7, 24.2. **MS (EI):** m/z (%) = 179 (47), 151 (14), 150 (100), 137 (33), 136 (20), 122 (17), 111 (22).

37: *N*^{1}-cyclopentyl-*N*^{4},*N*^{4}-dimethylbenzene-1,4-diamine

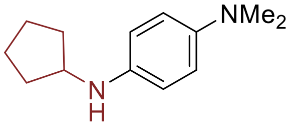

Amine **37** was synthesized according to general procedure *A* using the corresponding aryl chloride and cyclopentylamine. Purification by column chromatography (25% EtOAc/hexane) resulted in 132.7 mg of an orange oil (65% yield). **^{1}H NMR (400 MHz, $CDCl_3$):** δ = 6.76 – 6.71 (m, 2H), 6.63 – 6.58 (m, 2H), 3.73 (p, *J* = 5.5 Hz, 1H), 2.82 (s, 6H), 2.04 – 1.95 (m, 2H), 1.75 – 1.57 (m, 4H), 1.50 – 1.41 (m, 2H). NH proton was not visible. **^{13}C{^{1}H} NMR (101 MHz, $CDCl_3$):** δ = 144.0, 140.8, 116.1, 115.0, 55.8, 42.5, 33.8, 24.3. **IR (cm^{-1}):** 2985 (s), 2979 (s), 2969 (s), 2867 (w), 1518 (m), 1473 (w), 1382 (m), 1301 (w), 1254 (w), 1155 (w), 1136 (w), 1073 (w), 946 (w), 814 (m). **HRMS (ESI)** m/z: Calcd for $C_{13}H_{20}N_2$ $[M+H]^+$ 204.1627; Found 204.1631. **MS (EI):** m/z (%) = 205.2 (17), 204.2 (100), 175.1 (31), 162.1 (42), 147.1 (13), 136.1 (17), 135.1 (83).

38: *N*-cyclopentyl-3-(methylthio)aniline

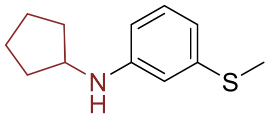

Amine **38** was synthesized according to general procedure *A* using the corresponding aryl chloride and cyclopentylamine. Purification by column chromatography (8% EtOAc/hexane) resulted in 184.1 mg of a yellow oil (89% yield). **^{1}H NMR (400 MHz, $CDCl_3$):** δ = 7.07 (t, *J* = 7.9 Hz, 1H), 6.60 – 6.56 (m, 1H), 6.50 (t, *J* = 2.1 Hz, 1H), 6.41 – 6.36 (m, 1H), 3.77 (p, *J* = 6.4 Hz, 1H), 3.66 (s, 1H), 2.46 (s, 3H), 2.07 – 1.97 (m, 2H), 1.76 – 1.58 (m, 4H), 1.50 – 1.42 (m, 2H). **^{13}C{^{1}H} NMR (101 MHz, $CDCl_3$):** δ = 148.5, 139.2, 129.6, 115.3, 111.2, 110.5, 54.7, 33.7, 24.2, 16.0. **IR (cm^{-1}):** 3649(w), 3393 (w), 2993 (m), 2984 (m), 2888 (s), 1590 (s), 1499 (m), 1478 (m), 1394 (s), 1387 (s), 1317 (m), 1154 (m), 1082 (m), 966 (w),

764 (w). **HRMS (ESI)** m/z: Calcd for $C_{12}H_{17}NS$ $[M]^+$ 207.1082; Found 207.1084. **MS (EI):** m/z (%) = 207,1 (100), 178,1 (98), 165 (46), 164,1 (18), 131,1 (30), 130,1 (58), 117,1 (21).

39: *N*-cyclopentylquinolin-2-amine

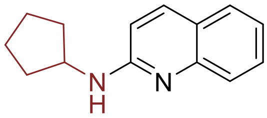

Amine **39** was synthesized according to general procedure *A* using the corresponding aryl chloride and cyclopentylamine. Purification by column chromatography (15% EtOAc/hexane) resulted in 165.4 mg of a white solid (78% yield). The analytical data is in accordance with the reported literature.[27] **^{1}H NMR (400 MHz, $CDCl_3$):** δ = 7.82 (d, *J* = 8.9 Hz, 1H), 7.66 – 7.61 (m, 1H), 7.61 – 7.54 (m, 1H), 7.54 – 7.48 (m, 1H), 7.23 – 7.15 (m, 1H), 6.67 (d, *J* = 8.9 Hz, 1H), 4.87 – 4.71 (m, 1H), 4.32 – 4.22 (m, 1H), 2.17 – 2.07 (m, 2H), 1.80 – 1.63 (m, 4H), 1.56 – 1.48 (m, 2H). **^{13}C{^{1}H} NMR (101 MHz, $CDCl_3$):** δ = 157.1, 148.4, 137.5, 129.7, 127.6, 126.2, 123.5, 122.0, 110.9, 53.4, 33.8, 24.0. **MS (EI):** m/z (%) = 212,1 (26), 183,1 (19), 169,1 (24), 145,1 (16), 144,1 (100), 129.0 (15), 128.0 (22).

40: 4-chloro-*N*-cyclohexylaniline

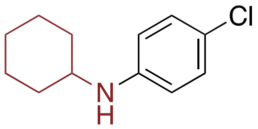

Amine **40** was synthesized according to general procedure *A* using the corresponding aryl bromide and cyclohexylamine. Purification by column chromatography (8% EtOAc/hexane) resulted in 149.2 mg of a yellow oil (72% yield). The analytical data is in accordance with the reported literature.[28] **^{1}H NMR (400 MHz, $CDCl_3$):** δ = 7.12 – 7.06 (m, 2H), 6.53 – 6.47 (m, 2H), 3.58 (s, 1H), 3.24 – 3.16 (m, 1H), 2.07 – 2.00 (m, 2H), 1.79 – 1.72 (m, 2H), 1.68 – 1.62 (m, 1H), 1.39 – 1.12 (m, 5H). **^{13}C{^{1}H} NMR (101 MHz, $CDCl_3$):** δ = 146.3, 129.5, 121.7, 114.7, 52.4, 33.8, 26.3, 25.4. **MS (EI):** m/z (%) = 211 (18), 209 (53), 168 (36), 166 (100), 131 (21), 130 (29), 127 (24).

41: *N*-cyclohexyl-6-methoxypyridin-2-amine

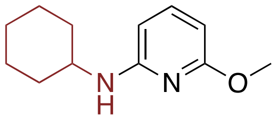

Amine **41** was synthesized according to general procedure *A* using the corresponding aryl chloride and cyclohexylamine. Purification by column chromatography (8% EtOAc/hexane) resulted in 187.6 mg of a yellow oil (91% yield). **^{1}H NMR (400 MHz, $CDCl_3$):** δ = 7.31 (t, *J* = 7.9 Hz, 1H), 5.97 (d, *J* = 8.6 Hz, 1H), 5.91 (d, *J* = 7.8 Hz, 1H), 4.44 – 4.21 (m, 1H), 3.83 (s, 3H), 3.54 – 3.43 (m, 1H), 2.09 – 2.01 (m, 2H), 1.80 – 1.72 (m, 2H), 1.66 – 1.61 (m, 1H), 1.42 – 1.16 (m, 5H). **^{13}C{^{1}H} NMR (101 MHz, $CDCl_3$)**: δ = 163.8, 157.2, 140.1, 97.8, 96.8, 53.2, 50.6, 33.5, 26.0, 25.1. **IR (cm^{-1}):** 3400 (w), 2980 (m), 2927 (s), 2851 (m), 1599 (s), 1581 (s), 1494 (m), 1456 (s), 1422 (s), 1404 (m), 1367 (w), 1343 (m), 1258 (s), 1143 (s), 1039 (m), 778 (s), 724 (m). **HRMS (ESI)** m/z: Calcd for $C_{12}H_{18}N_2O$ $[M]^+$ 206.1419; Found 206.1422. **MS (EI):** m/z (%) = 206.1 (63), 163.1 (55), 149.1 (100), 124.1 (78), 123.1 (32), 95.1 (17), 93.0 (17).

42: 4-(cycloheptylamino)benzonitrile

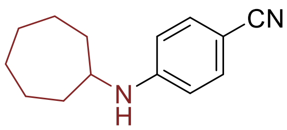

Amine **42** was synthesized according to general procedure *A* using the corresponding aryl chloride and cycloheptylamine. Purification by column chromatography (8% EtOAc/hexane) resulted in 196.1 mg of a yellow oil (92% yield). The analytical data is in accordance with the reported

literature.[29] **^{1}H NMR (400 MHz, $CDCl_3$):** δ = 7.42 – 7.37 (m, 2H), 6.50 – 6.45 (m, 2H), 4.19 – 4.11 (m, 1H), 3.52 – 3.42 (m, 1H), 2.03 – 1.94 (m, 2H), 1.70 – 1.46 (m, 10H). **^{13}C{^{1}H} NMR (101 MHz, $CDCl_3$):** δ = 150.4, 133.9, 120.7, 112.6, 98.1, 53.5, 34.8, 28.3, 24.4. **MS (EI):** m/z (%) = 214.1 (29), 171.1 (17), 158.1 (15), 157.1 (100), 144.1 (13), 131.0 (15), 118.0 (18).

43: 1-(4-(cycloheptylamino)phenyl)ethan-1-one

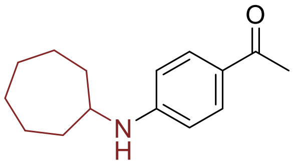

Amine **43** was synthesized according to general procedure *A* using the corresponding aryl chloride and cycloheptylamine. Purification by column chromatography (25% EtOAc/hexane) resulted in 70.8 mg of a yellow oil (31% yield). **^{1}H NMR (400 MHz, $CDCl_3$):** δ = 7.84 – 7.78 (m, 2H), 6.51 – 6.46 (m, 2H), 4.22 – 4.09 (m, 1H), 3.53 (s, 1H), 2.49 (s, 3H), 2.06 – 1.97 (m, 2H), 1.73 – 1.45 (m, 10H**). ^{13}C{^{1}H} NMR (101 MHz, $CDCl_3$):** δ = 196.3, 151.3, 131.0, 126.4, 111.8, 53.5, 34.9, 28.4, 26.1, 24.4. **IR (cm^{-1}):** 3360 (m), 2919 (m), 2854 (w) 1642 (s), 1587 (s), 1531 (s), 1490 (m), 1458 (w), 1355 (s), 1279 (s), 1229 (m), 1173 (s), 1074 (m), 952 (m), 831 (s), 591 (s), 437 (m). **HRMS (ESI)** m/z: Calcd for $C_{15}H_{21}NO$ $[M]^+$ 231.1623; Found 231.1626. **MS (EI):** m/z (%) = 231 (47), 188 (21), 174 (100), 146 (20), 132 (19), 130 (17), 120 (21).

44: *N*-(3-methoxyphenyl)cycloheptanamine

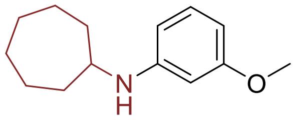

Amine **44** was synthesized according to general procedure *A* using the corresponding aryl chloride and cycloheptylamine. Purification by column chromatography (5% EtOAc/hexane) resulted in 115.5 mg of a yellow oil (53% yield). **^{1}H NMR (400 MHz, $CDCl_3$):** δ = 7.06 (t, *J* = 8.1 Hz, 1H), 6.23 (dd, *J* = 8.2, 0.9 Hz, 1H), 6.17 (dd, *J* = 8.1, 0.9 Hz, 1H), 6.10 (t, *J* = 2.3 Hz, 1H), 3.77 (s, 3H), 3.61 (s, 1H), 3.47 – 3.38 (m, 1H), 2.06 – 1.96 (m, 2H), 1.69 – 1.44 (m, 10H). **^{13}C{^{1}H} NMR (101 MHz, $CDCl_3$):** δ = 161.0, 148.9, 130.1, 106.6, 101.9, 99.3, 55.2, 53.9, 35.0, 28.5, 24.6. **IR (cm^{-1}):** 3395 (w), 2924 (s), 2852 (m), 1615 (s), 1509 (m), 1495 (m), 1462 (m), 1342 (w), 1301 (w), 1257 (w), 1212 (s), 1161 (s), 826 (w), 689 (m). **HRMS (ESI)** m/z: Calcd for $C_{14}H_{21}NO$ $[M]^+$ 219.1623; Found 219.1627. **MS (EI):** m/z (%) = 219.2 (44), 176.1 (17), 163.1 (13), 162.1 (100), 149.1 (15), 148.1 (13), 136.1 (15).

3 Further Substrates Tested in the Arylation of Cyclopropylamine

Substrates with minimal or no conversion:

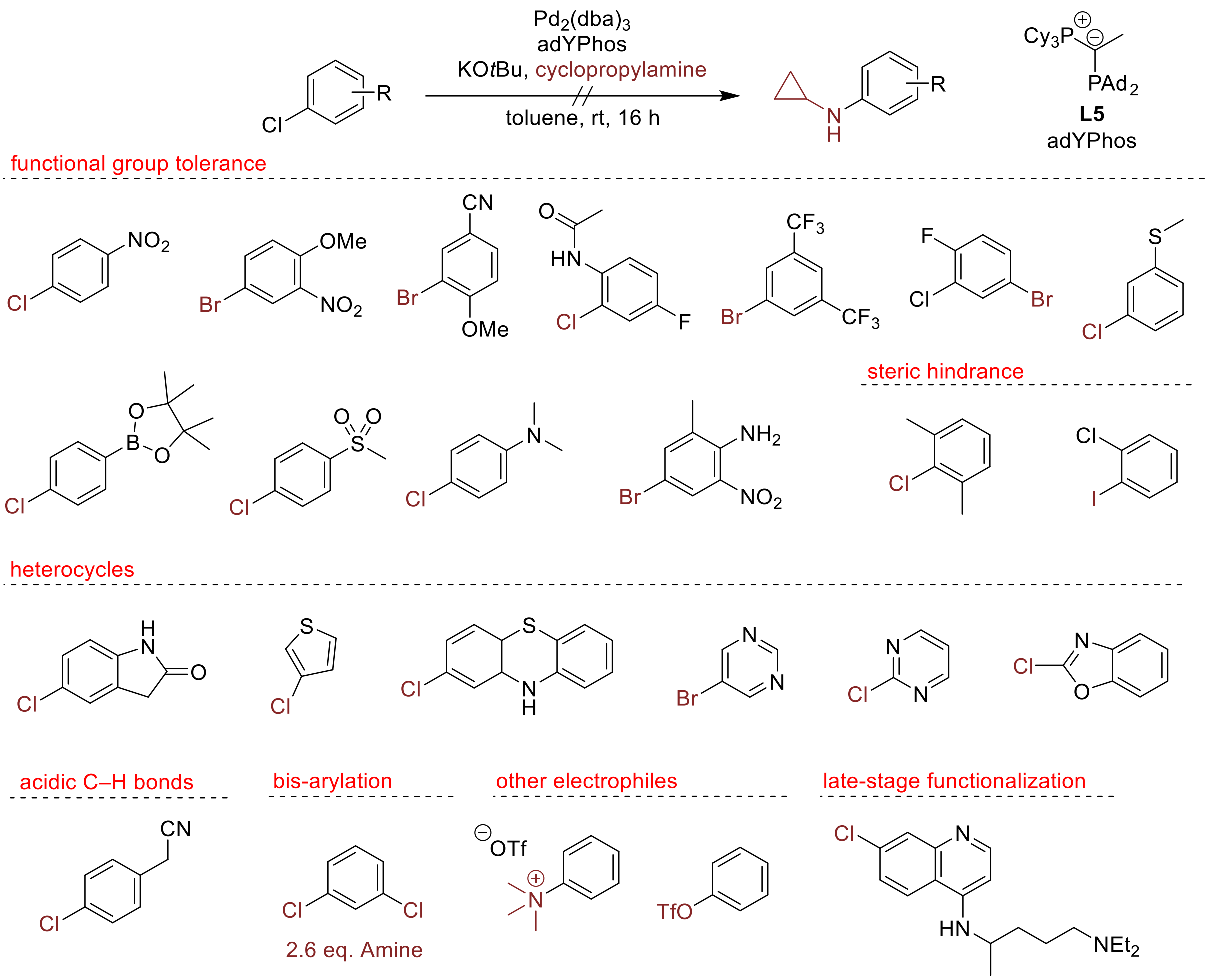

Figure S8. (Hetero)aryl (pseudo)halides, which could not be coupled with cyclopropylamine. Reaction conditions: 1.0 mmol ArCl, 1.3 mmol cyclopropylamine, 0.02 mmol [Pd], Pd/L ratio 1:1, rt, 16 h in toluene.

4 NMR Spectra

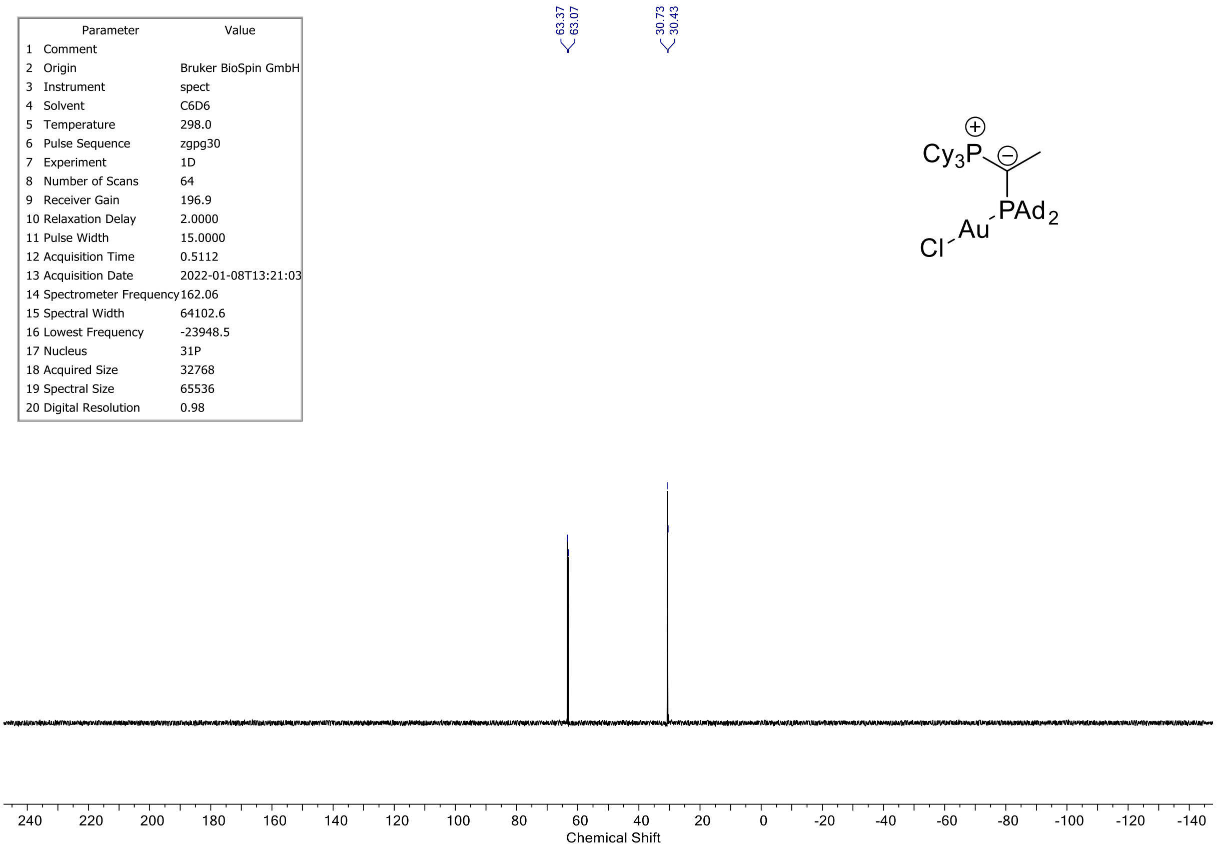

	Parameter	Value
1	Comment	
2	Origin	Bruker BioSpin GmbH
3	Instrument	spect
4	Solvent	C6D6
5	Temperature	298.0
6	Pulse Sequence	zgpg30
7	Experiment	1D
8	Number of Scans	64
9	Receiver Gain	196.9
10	Relaxation Delay	2.0000
11	Pulse Width	15.0000
12	Acquisition Time	0.5112
13	Acquisition Date	2022-01-08T13:21:03
14	Spectrometer Frequency	162.06
15	Spectral Width	64102.6
16	Lowest Frequency	-23948.5
17	Nucleus	31P
18	Acquired Size	32768
19	Spectral Size	65536
20	Digital Resolution	0.98

Figure S9. $^{31}P\{^{1}H\}$ NMR spectrum of **L5-AuCl**.

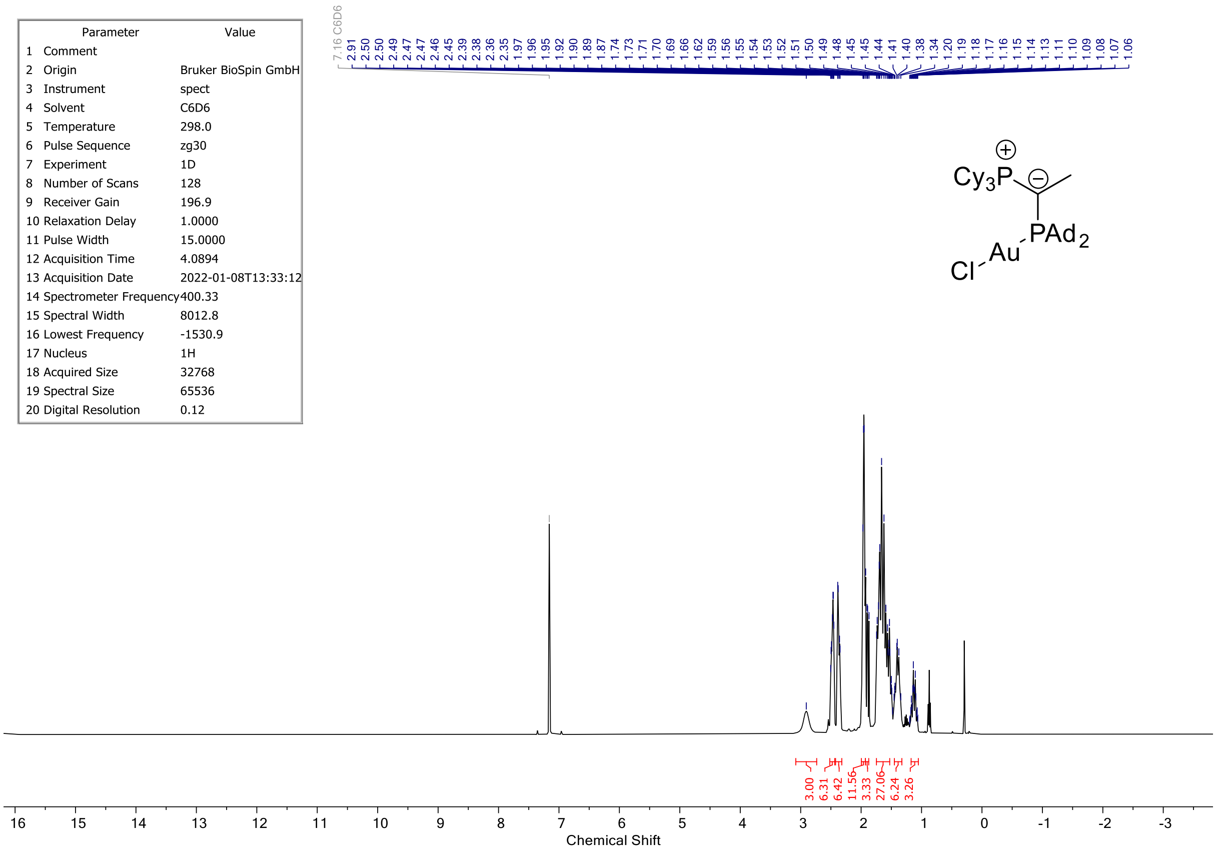

	Parameter	Value
1	Comment	
2	Origin	Bruker BioSpin GmbH
3	Instrument	spect
4	Solvent	C6D6
5	Temperature	298.0
6	Pulse Sequence	zg30
7	Experiment	1D
8	Number of Scans	128
9	Receiver Gain	196.9
10	Relaxation Delay	1.0000
11	Pulse Width	15.0000
12	Acquisition Time	4.0894
13	Acquisition Date	2022-01-08T13:33:12
14	Spectrometer Frequency	400.33
15	Spectral Width	8012.8
16	Lowest Frequency	-1530.9
17	Nucleus	1H
18	Acquired Size	32768
19	Spectral Size	65536
20	Digital Resolution	0.12

Figure S10. ^{1}H NMR spectrum of **L5-AuCl**.

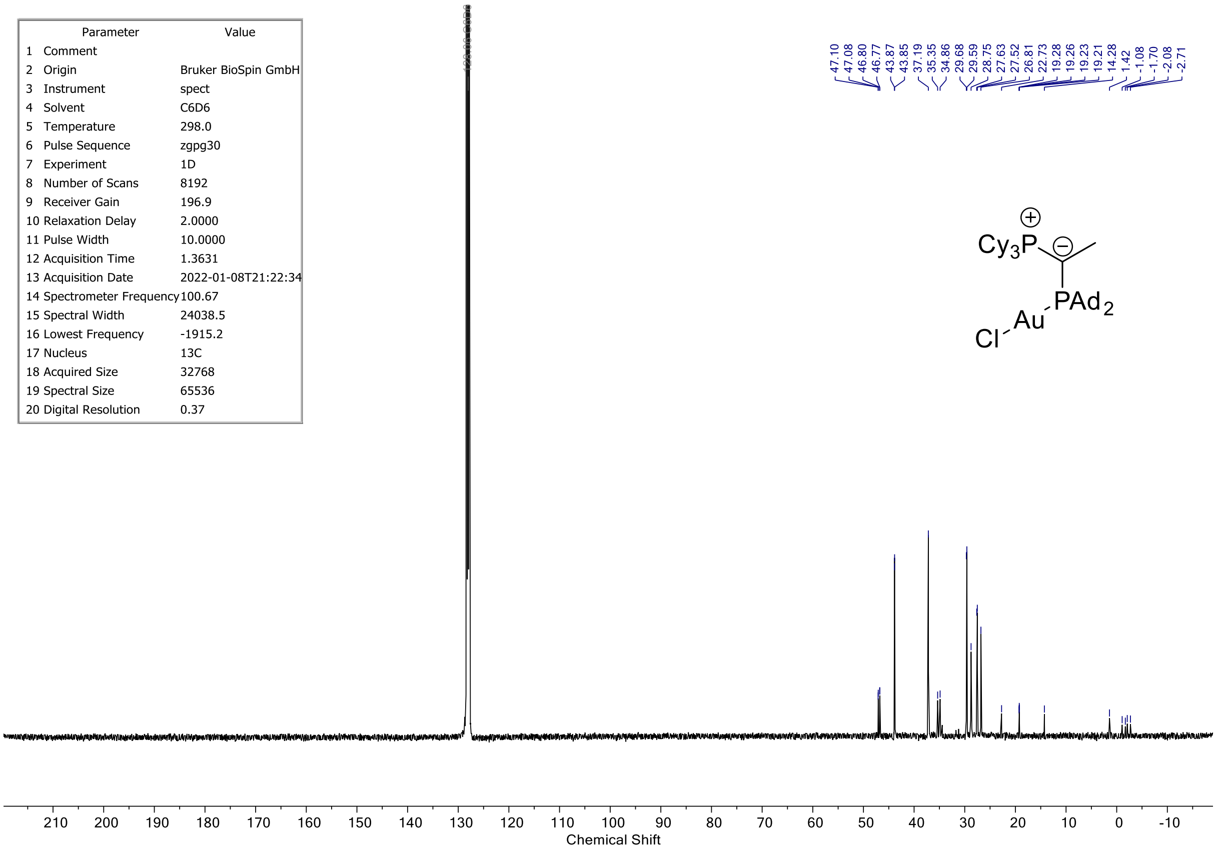

Figure S11. $^{13}C\{^{1}H\}$ NMR spectrum of **L5-AuCl**.

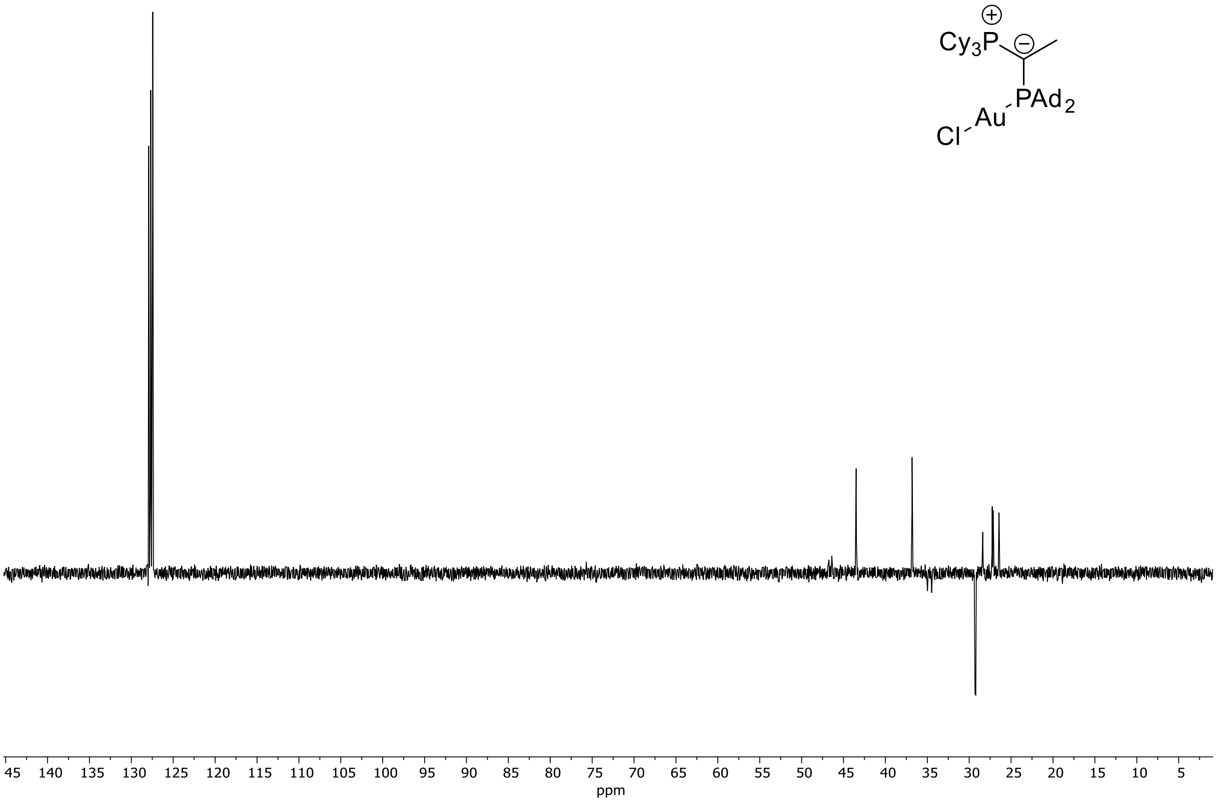

Figure S12. $^{13}C\{^{1}H\}$ APT NMR spectrum of **L5-AuCl**.

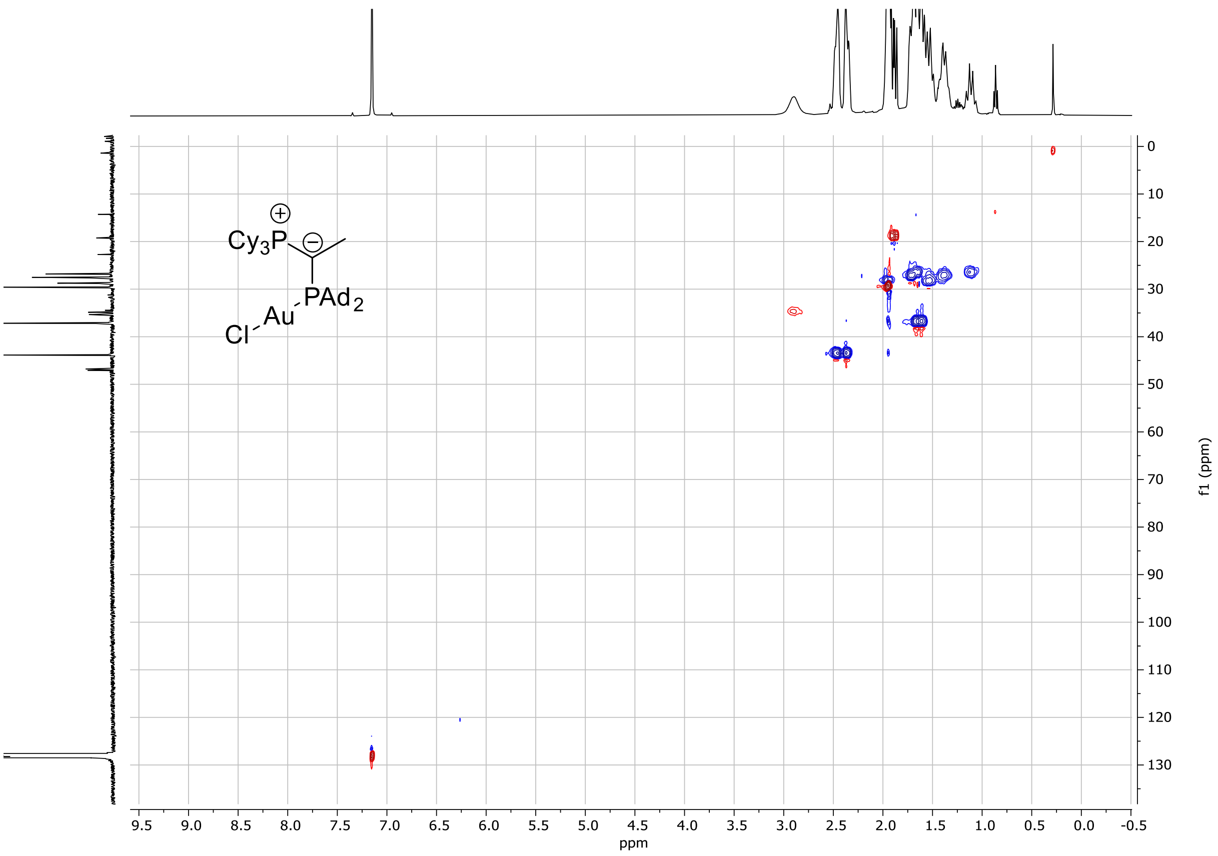

Figure S13. HSQC NMR spectrum of **L5-AuCl**.

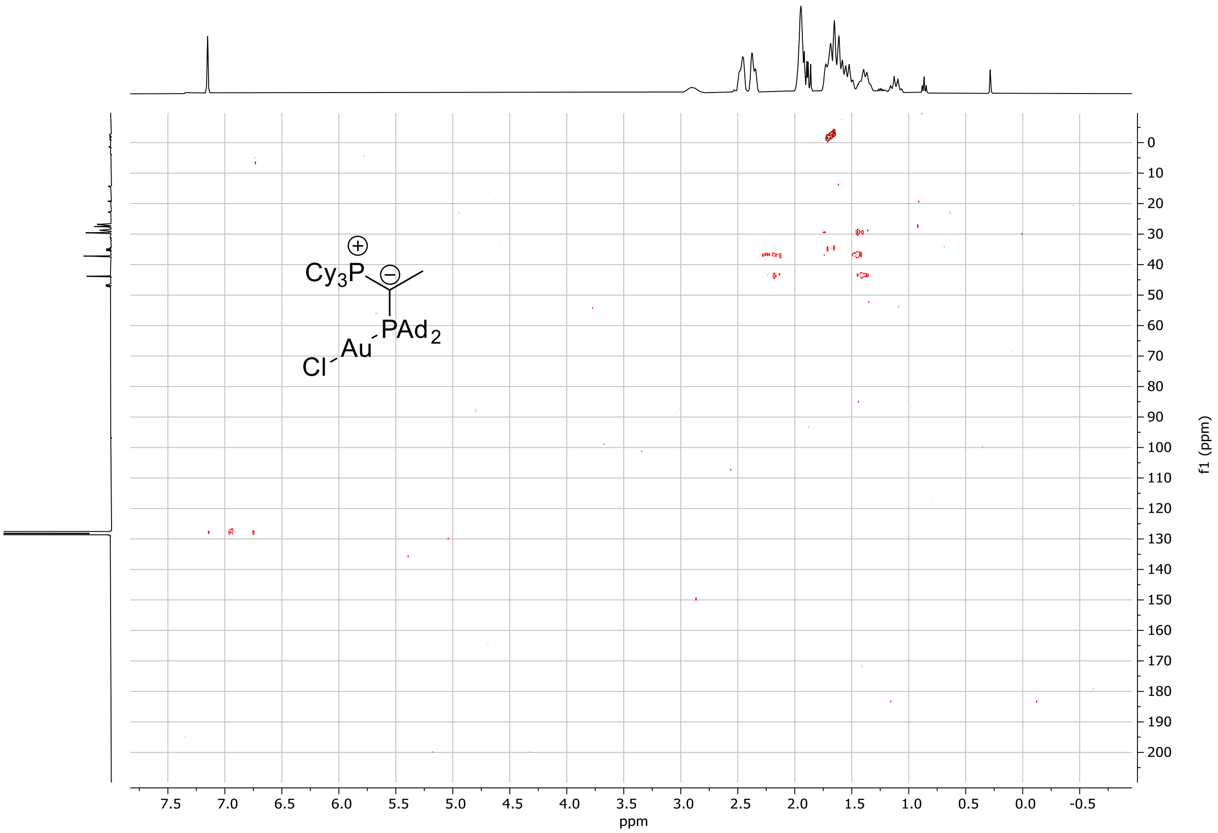

Figure S14. HMBC NMR spectrum of **L5-AuCl**.

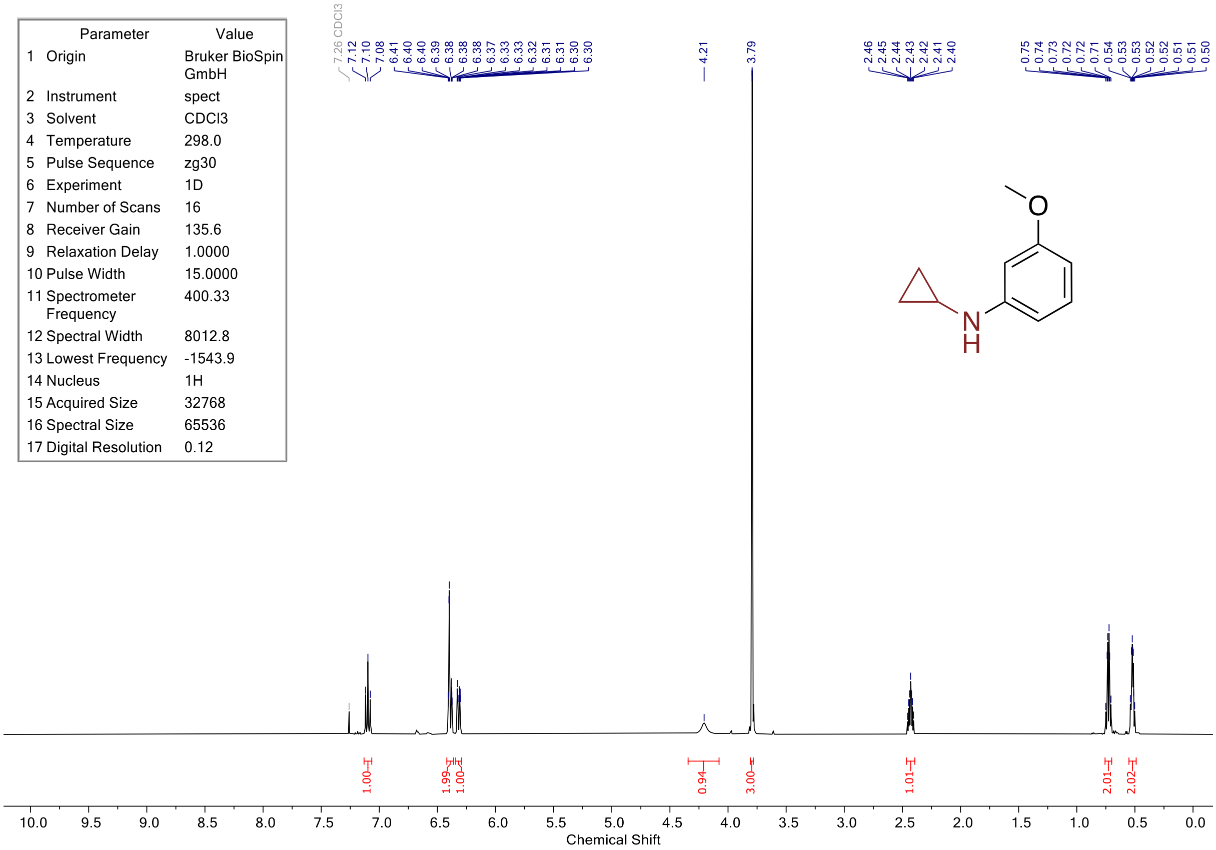

Figure S15. ^{1}H NMR spectrum of **1** from aryl chloride.

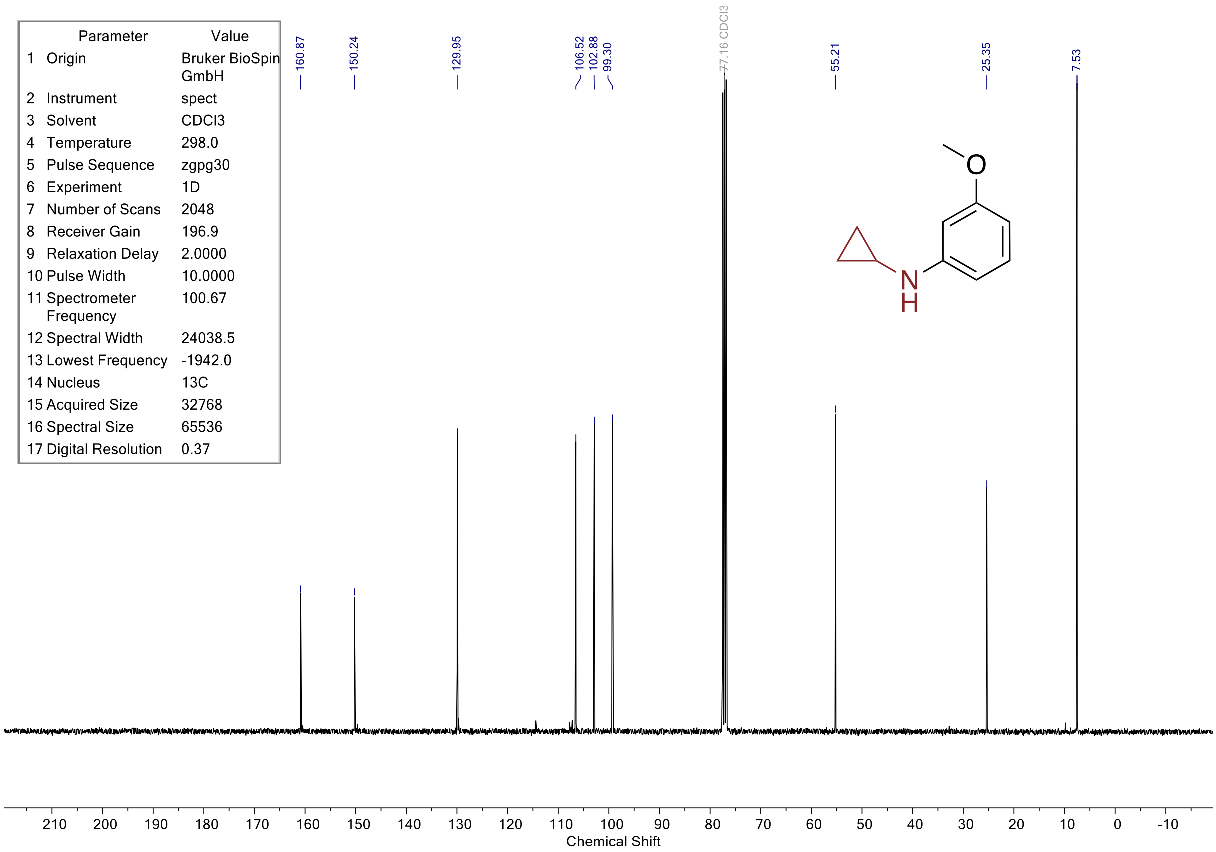

Figure S16. $^{13}C\{^{1}H\}$ NMR spectrum of **1** from aryl chloride.

Parameter		Value
1	Origin	Bruker BioSpin GmbH
2	Instrument	spect
3	Solvent	CDCl3
4	Temperature	298.0
5	Pulse Sequence	zg30
6	Experiment	1D
7	Number of Scans	16
8	Receiver Gain	48.9
9	Relaxation Delay	1.0000
10	Pulse Width	10.0000
11	Spectrometer Frequency	400.33
12	Spectral Width	8012.8
13	Lowest Frequency	-1544.0
14	Nucleus	1H
15	Acquired Size	32768
16	Spectral Size	65536
17	Digital Resolution	0.12

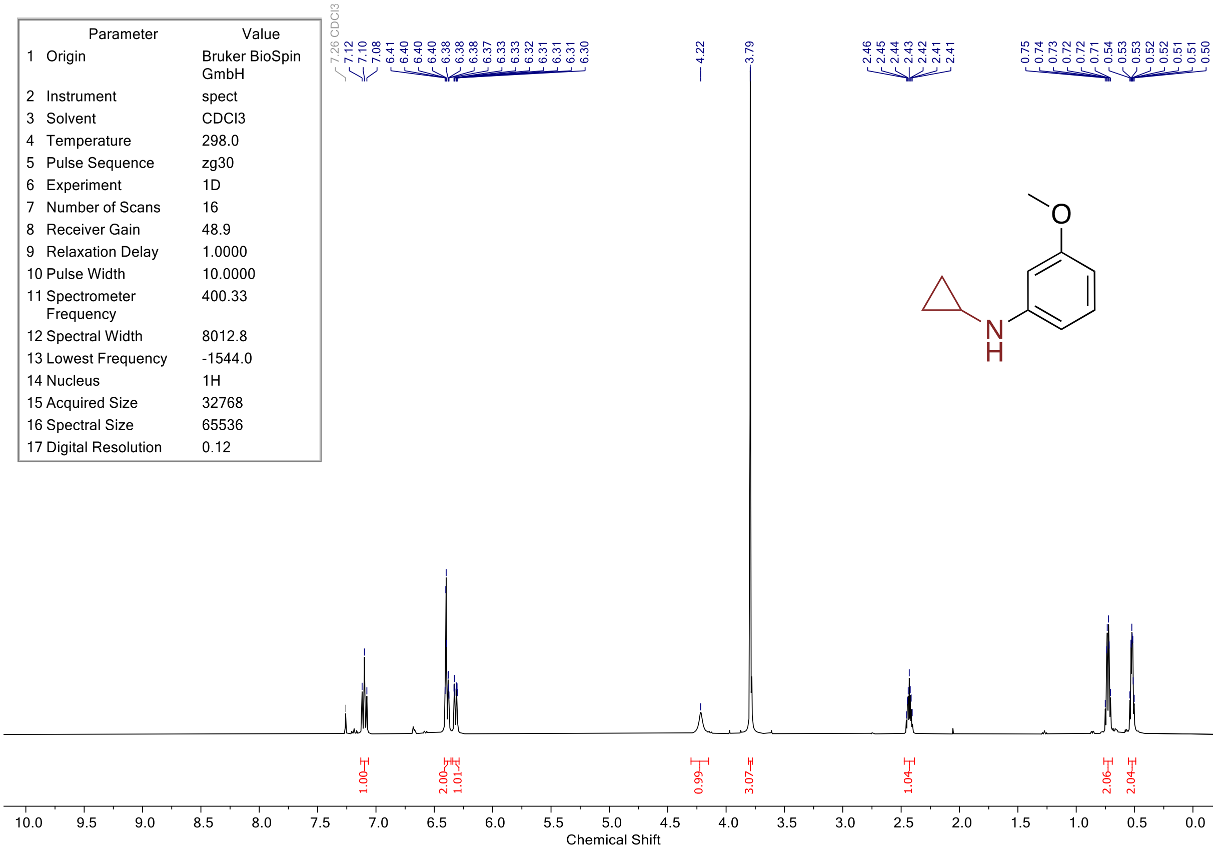

Figure S17. ^{1}H NMR spectrum of **1** from aryl bromide.

Parameter		Value
1	Origin	Bruker BioSpin GmbH
2	Instrument	spect
3	Solvent	CDCl3
4	Temperature	298.0
5	Pulse Sequence	zgpg30
6	Experiment	1D
7	Number of Scans	2048
8	Receiver Gain	196.9
9	Relaxation Delay	2.0000
10	Pulse Width	10.0000
11	Spectrometer Frequency	100.67
12	Spectral Width	24038.5
13	Lowest Frequency	-1910.1
14	Nucleus	13C
15	Acquired Size	32768
16	Spectral Size	65536
17	Digital Resolution	0.37

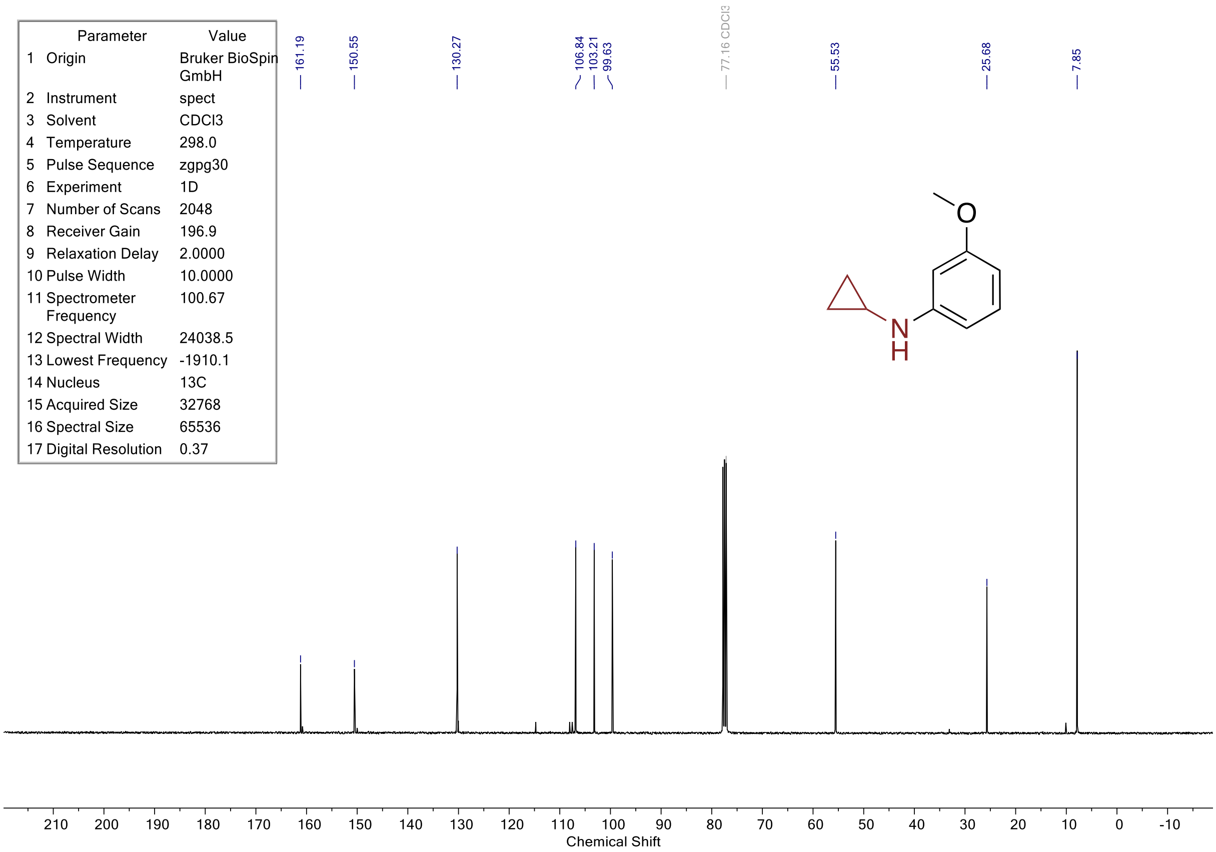

Figure S18. $^{13}C\{^1H\}$ NMR spectrum of **1** from aryl bromide.

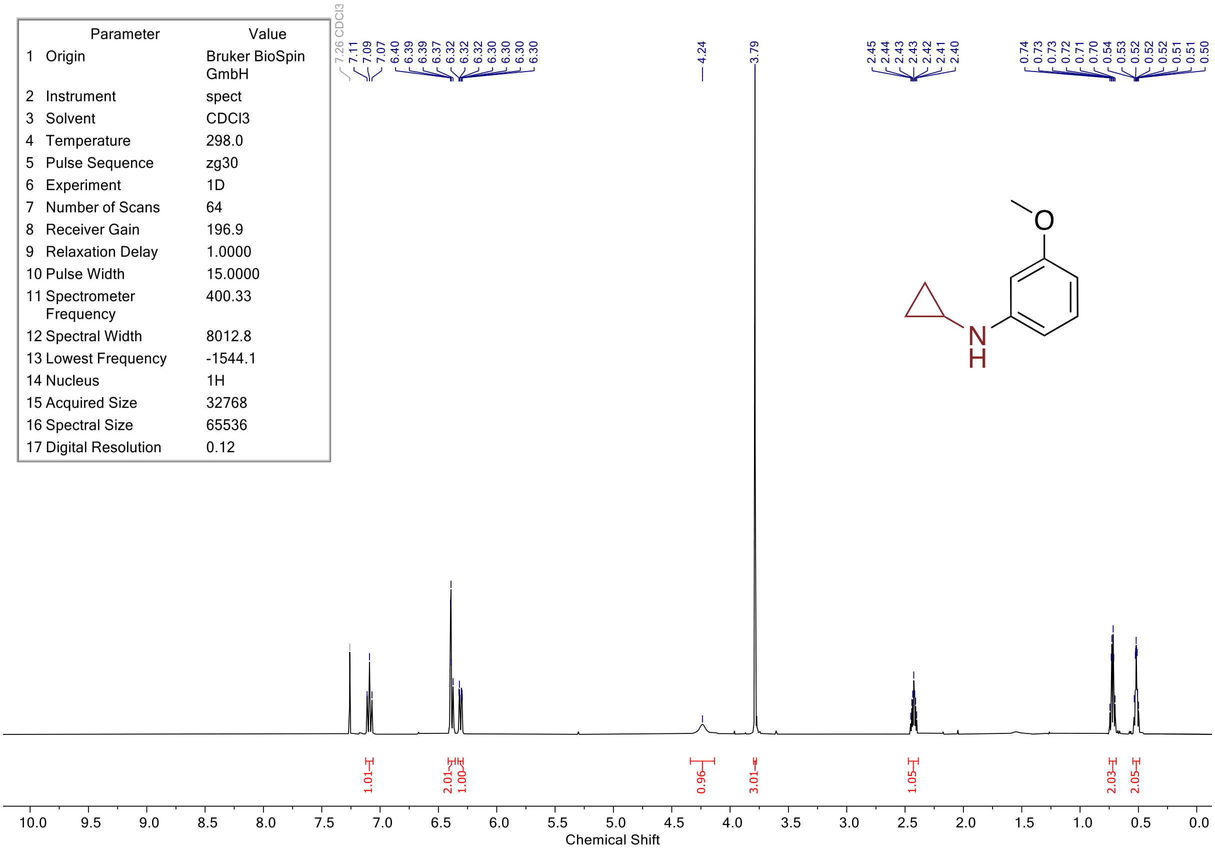

Figure S19. ^{1}H NMR spectrum of **1** from aryl iodide.

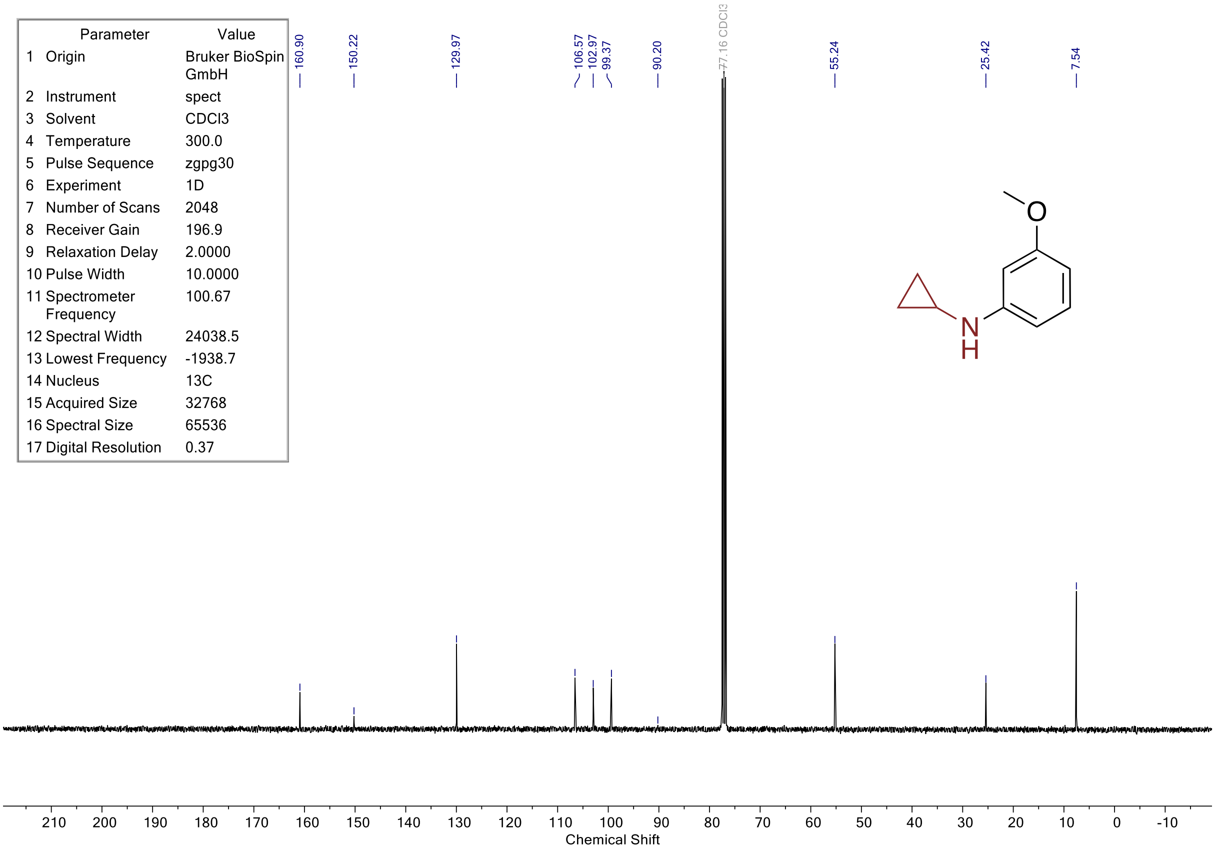

Figure S20. $^{13}C\{^1H\}$ NMR spectrum of **1** from aryl iodide.

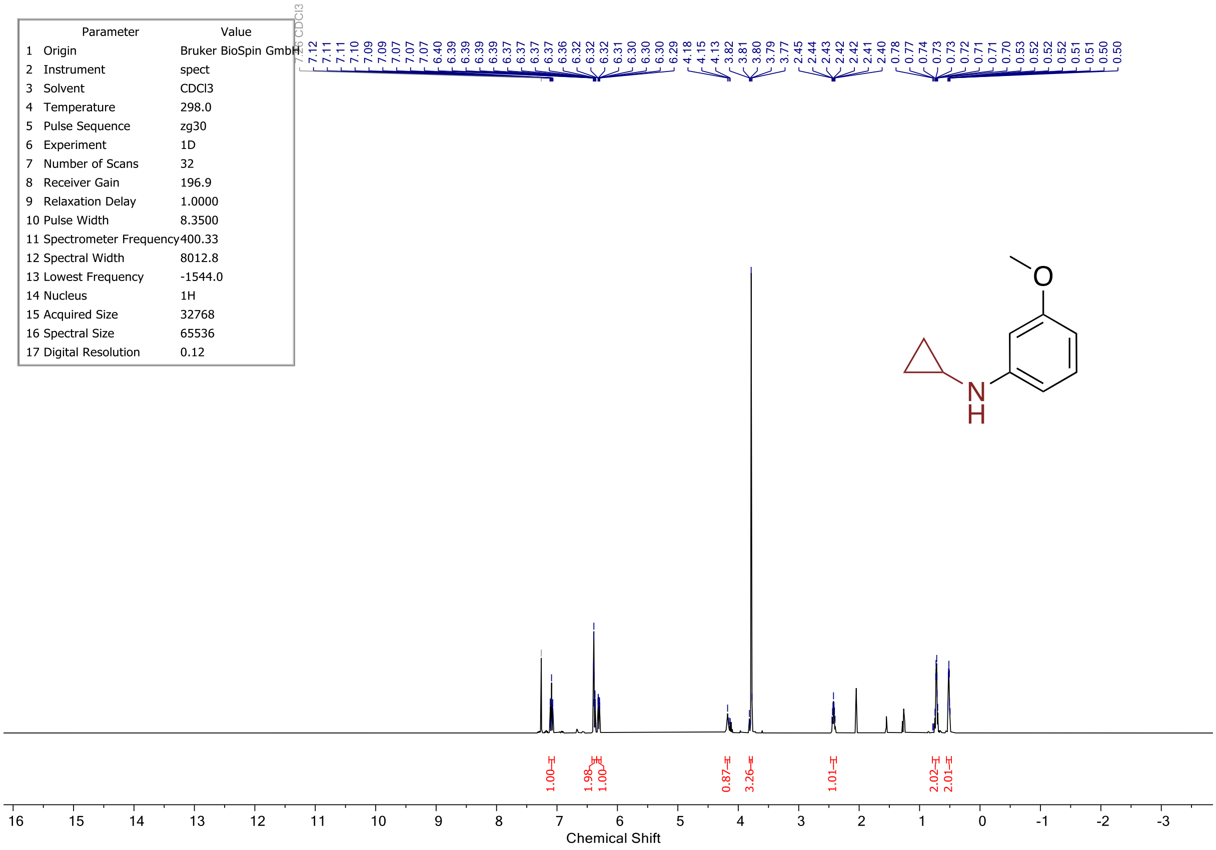

Figure S21. ^{1}H NMR spectrum of **1** from aryl chloride on a large-scale.

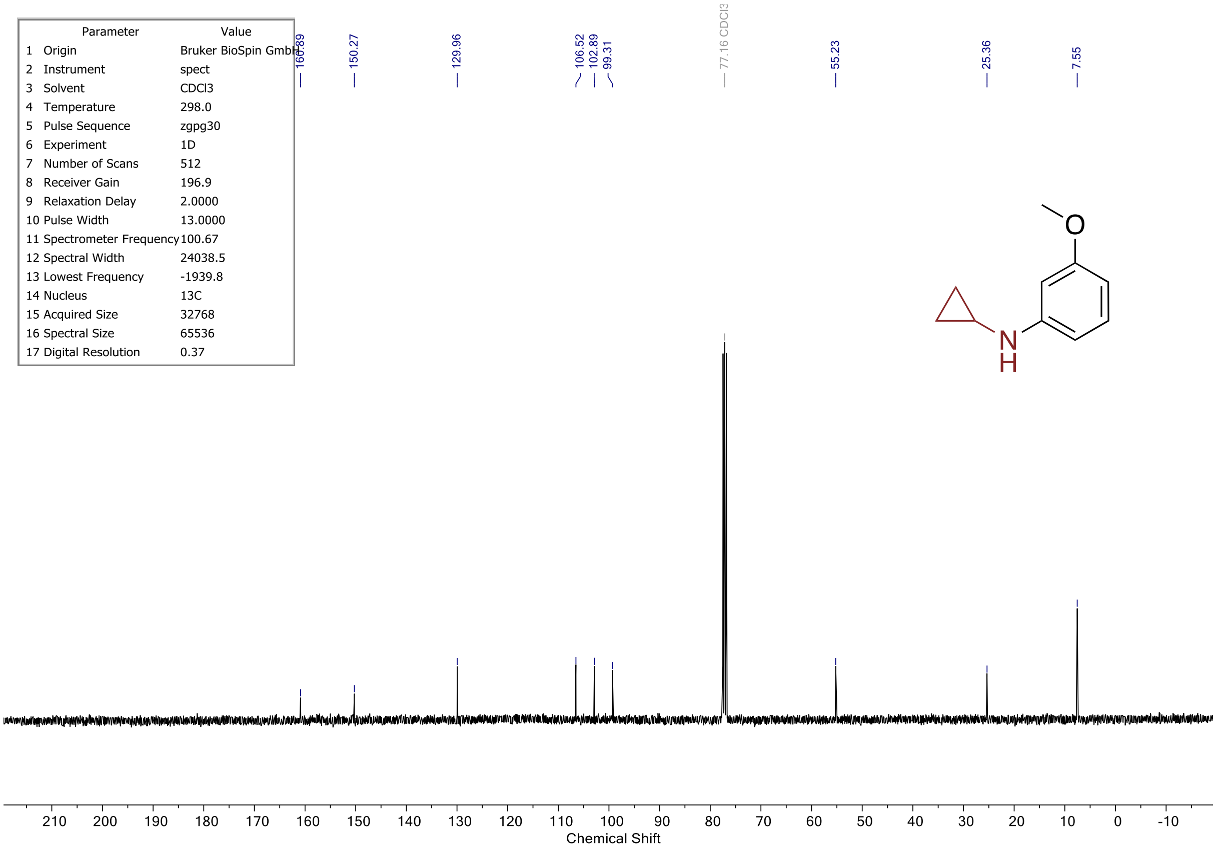

Figure S22. ^{13}C{^{1}H} NMR spectrum of **1** from aryl chloride on a large-scale.

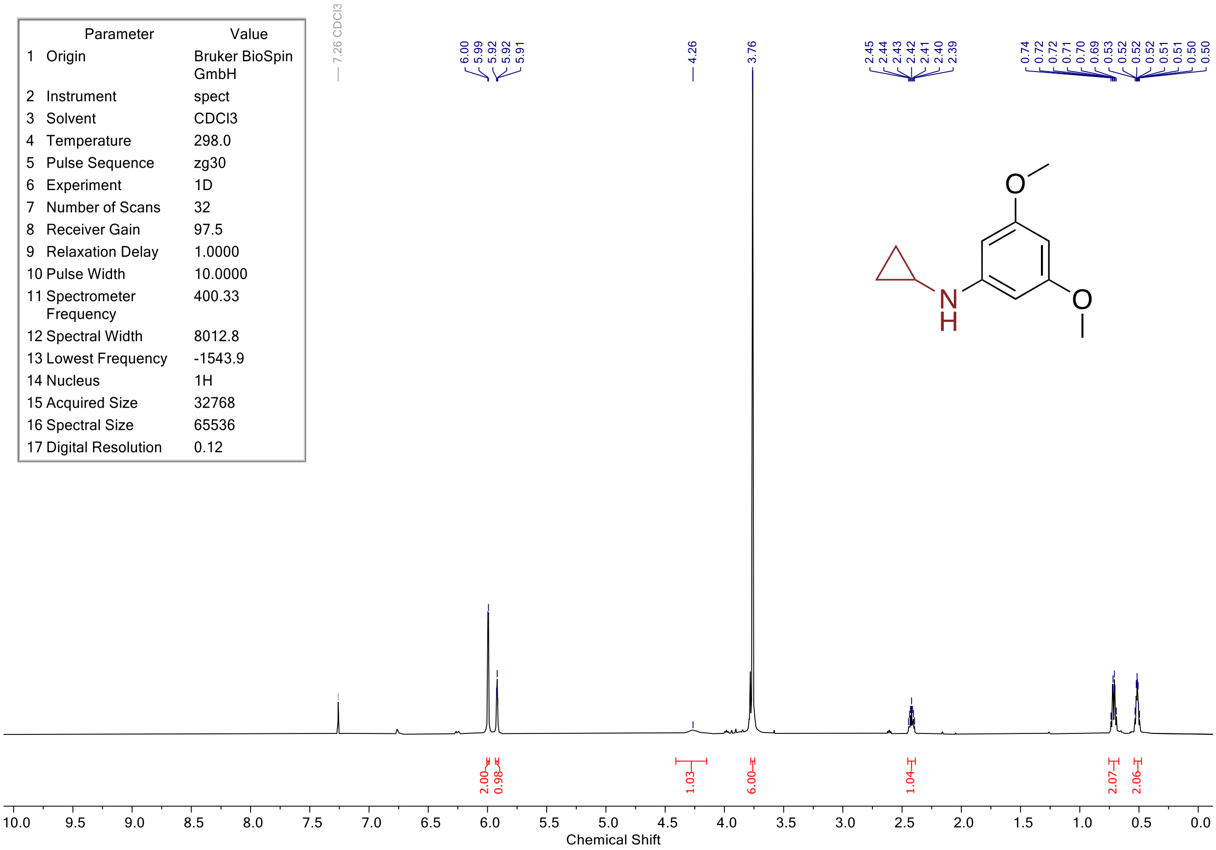

Figure S23. ^{1}H NMR spectrum of **2**.

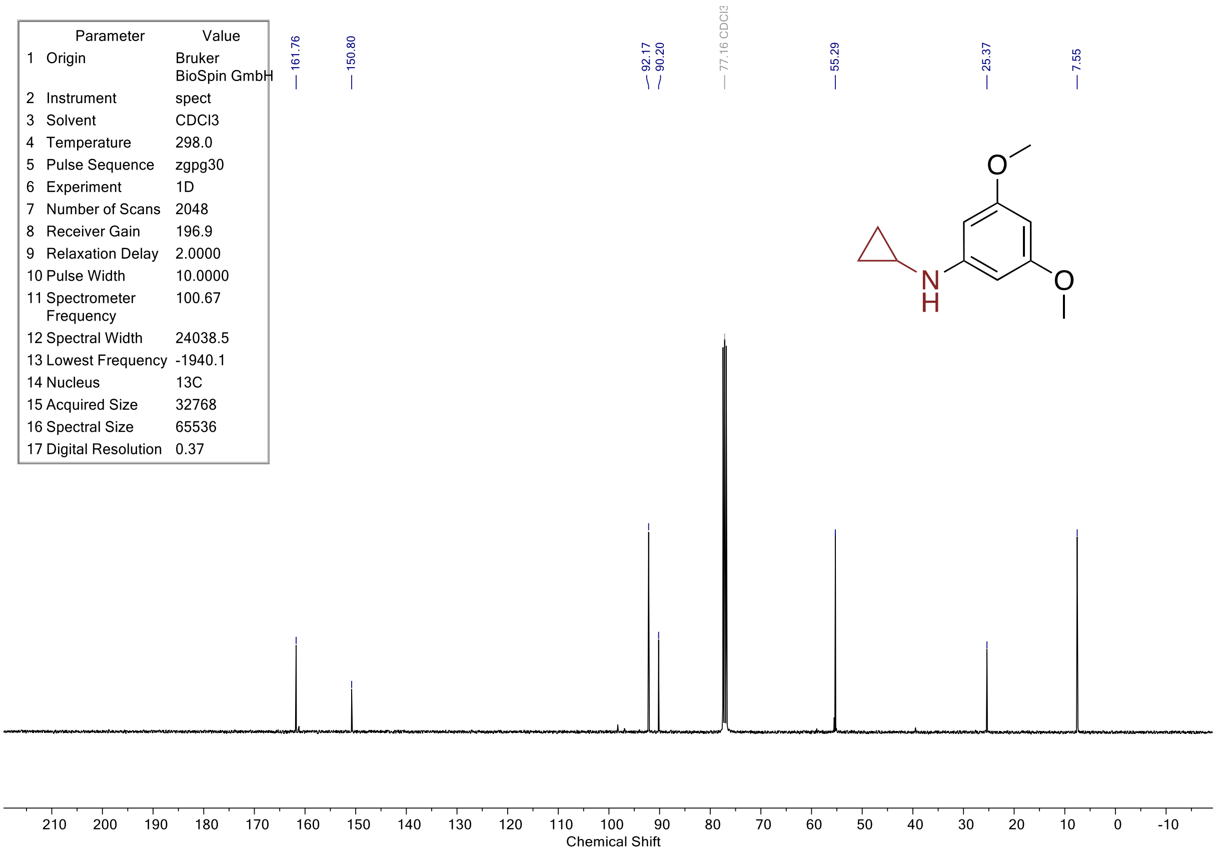

Figure S24. $^{13}C\{^{1}H\}$ NMR spectrum of **2**.

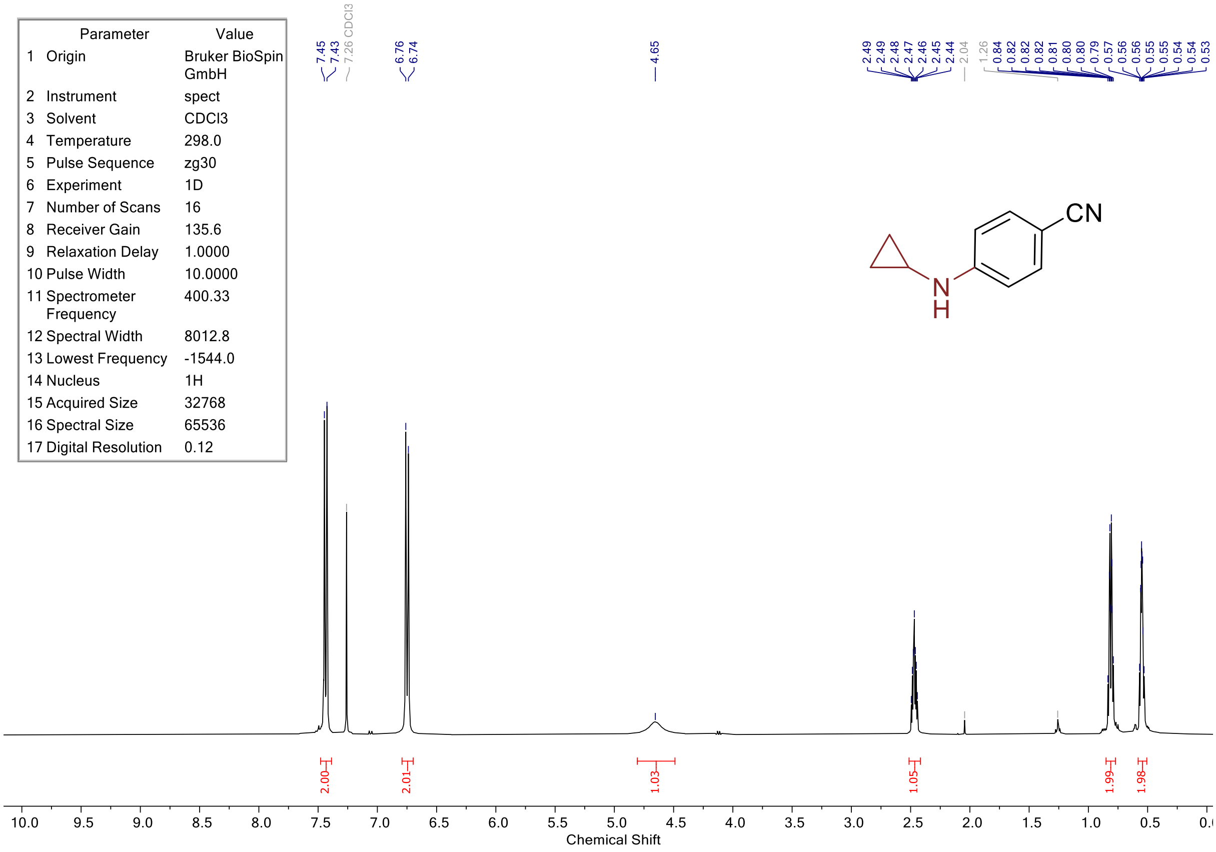

Figure S25. ^{1}H NMR spectrum of **3**.

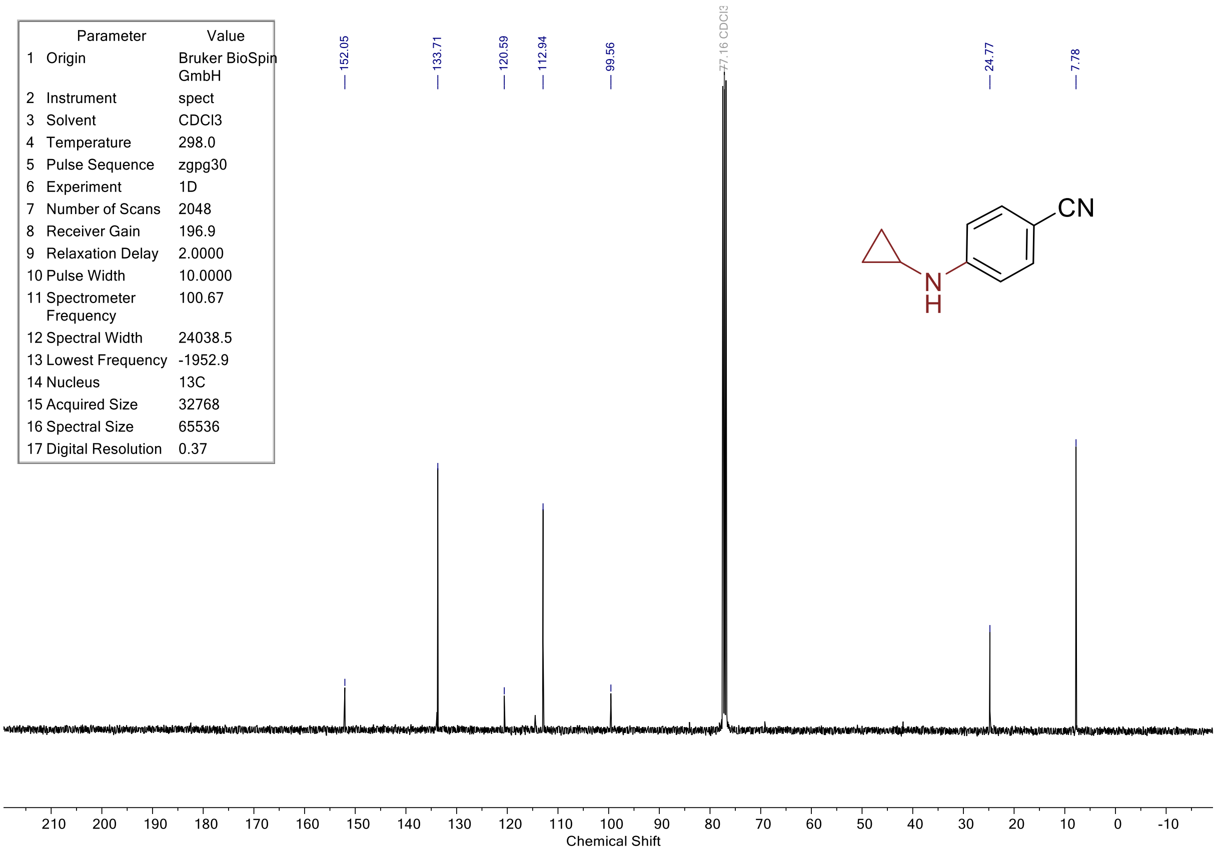

Figure S26. ^{13}C{^{1}H} NMR spectrum of **3**.

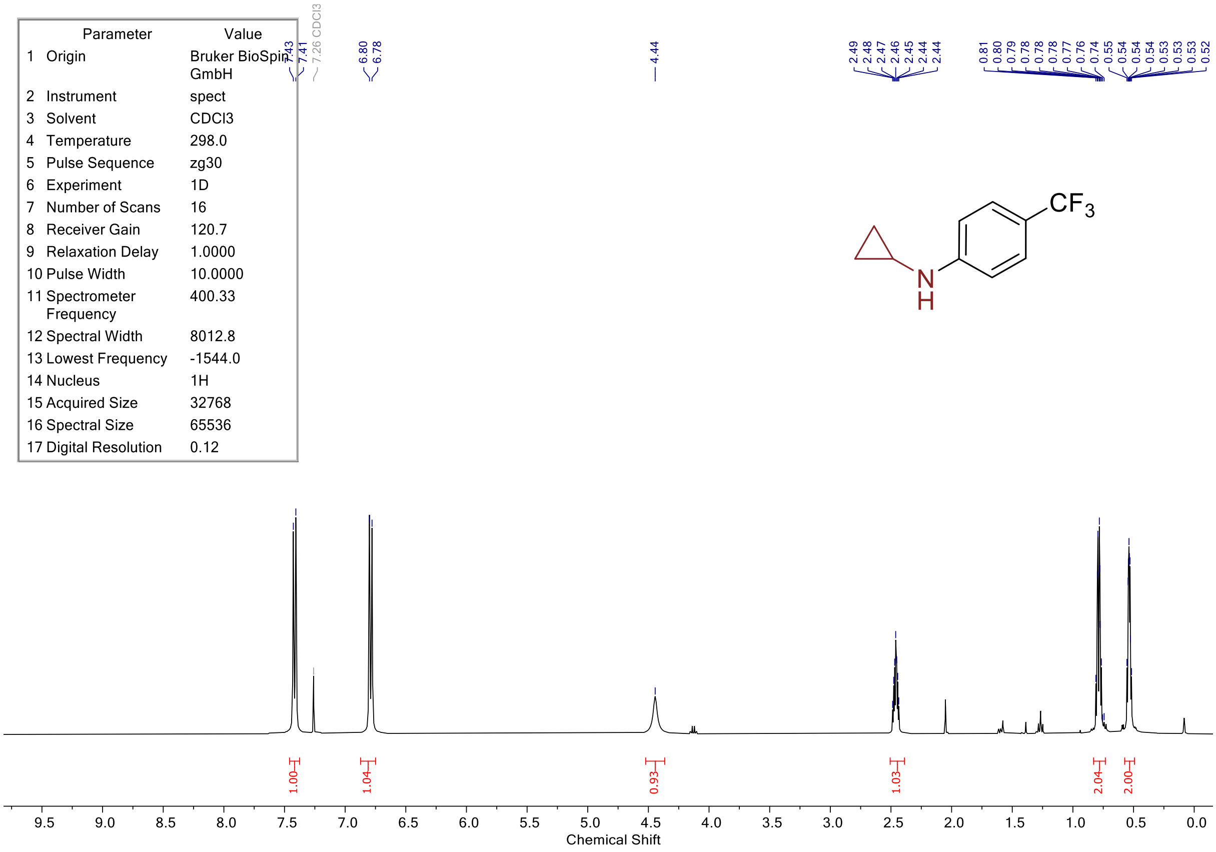

	Parameter	Value
1	Origin	Bruker BioSpin GmbH
2	Instrument	spect
3	Solvent	CDCl3
4	Temperature	298.0
5	Pulse Sequence	zg30
6	Experiment	1D
7	Number of Scans	16
8	Receiver Gain	120.7
9	Relaxation Delay	1.0000
10	Pulse Width	10.0000
11	Spectrometer Frequency	400.33
12	Spectral Width	8012.8
13	Lowest Frequency	-1544.0
14	Nucleus	1H
15	Acquired Size	32768
16	Spectral Size	65536
17	Digital Resolution	0.12

Figure S27. ^{1}H NMR spectrum of **4**.

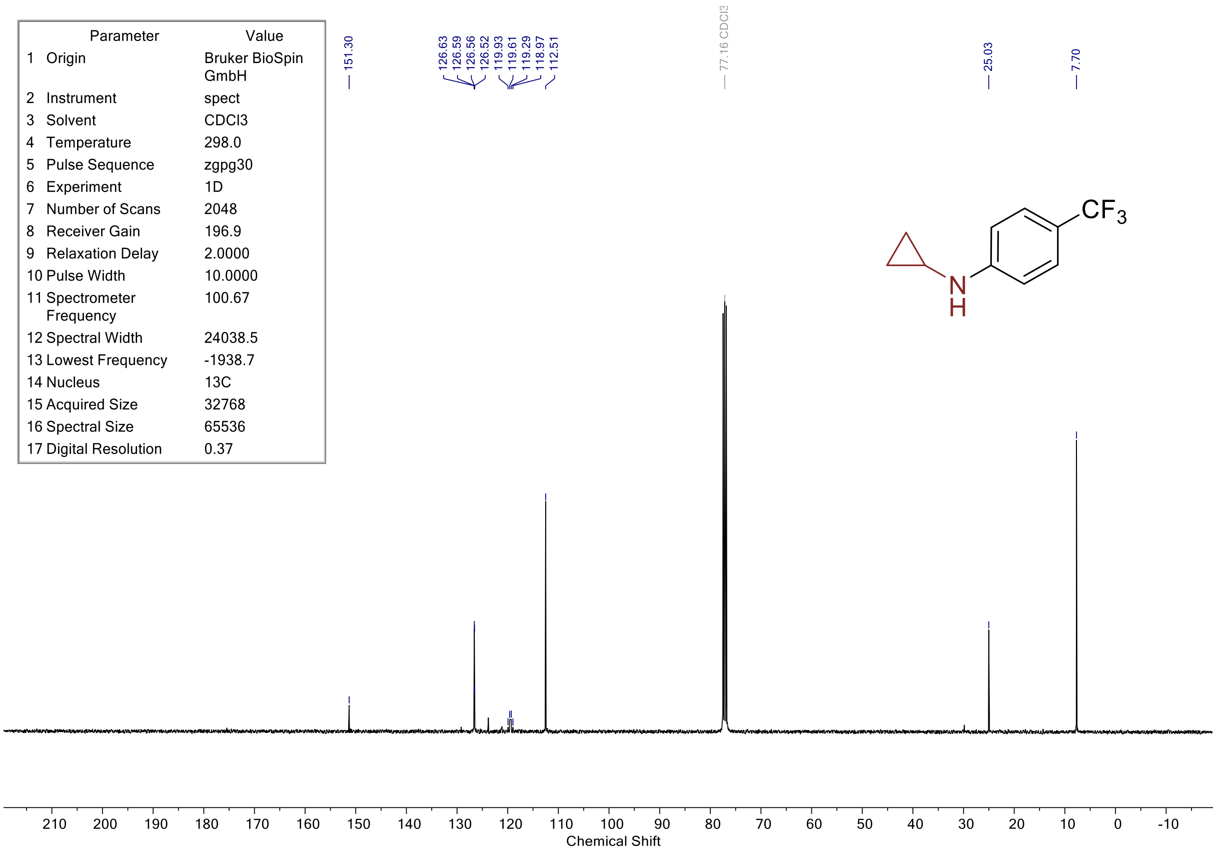

	Parameter	Value
1	Origin	Bruker BioSpin GmbH
2	Instrument	spect
3	Solvent	CDCl3
4	Temperature	298.0
5	Pulse Sequence	zgpg30
6	Experiment	1D
7	Number of Scans	2048
8	Receiver Gain	196.9
9	Relaxation Delay	2.0000
10	Pulse Width	10.0000
11	Spectrometer Frequency	100.67
12	Spectral Width	24038.5
13	Lowest Frequency	-1938.7
14	Nucleus	13C
15	Acquired Size	32768
16	Spectral Size	65536
17	Digital Resolution	0.37

Figure S28. $^{13}C\{^{1}H\}$ NMR spectrum of **4**.

	Parameter	Value
1	Origin	Bruker BioSpin GmbH
2	Instrument	spect
3	Solvent	CDCl3
4	Temperature	298.0
5	Pulse Sequence	zg30
6	Experiment	1D
7	Number of Scans	16
8	Receiver Gain	120.7
9	Relaxation Delay	1.0000
10	Pulse Width	10.0000
11	Spectrometer Frequency	400.33
12	Spectral Width	8012.8
13	Lowest Frequency	-1544.5
14	Nucleus	1H
15	Acquired Size	32768
16	Spectral Size	65536
17	Digital Resolution	0.12

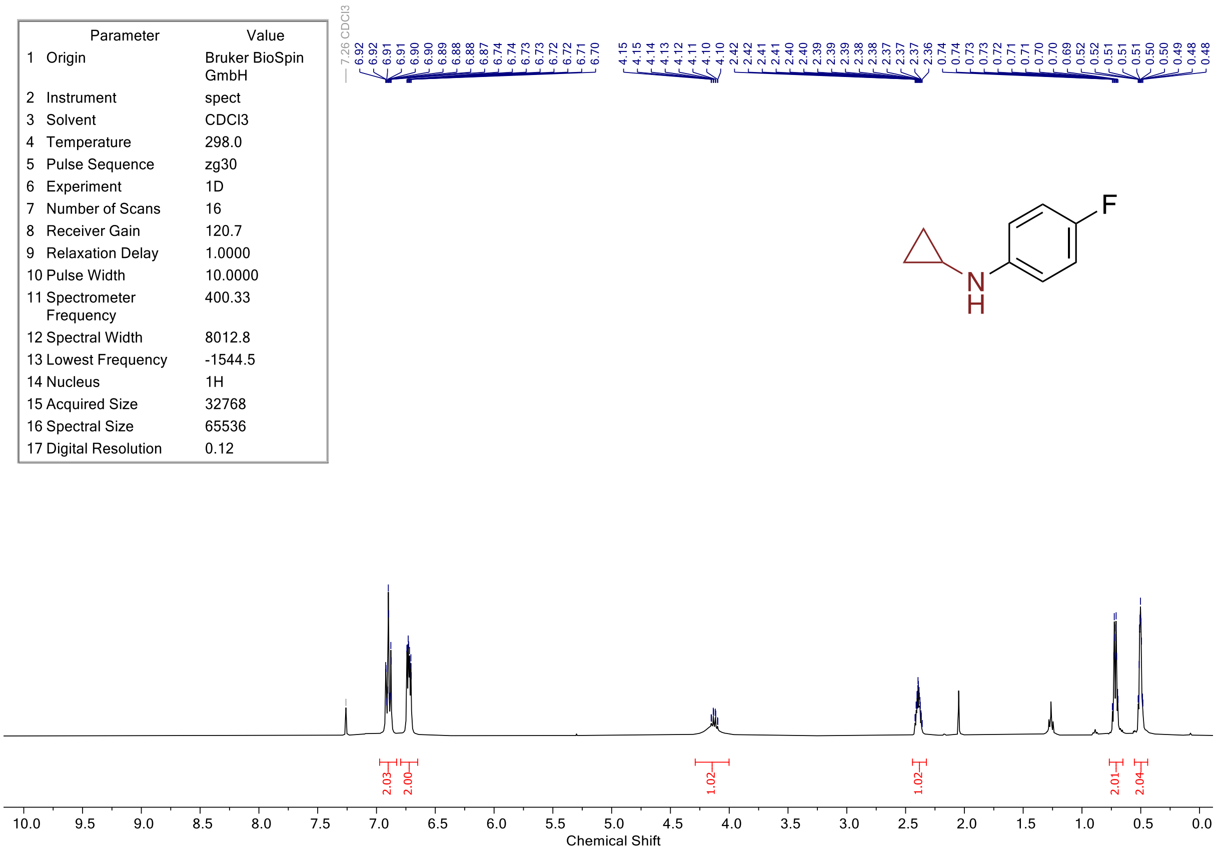

Figure S29. ^{1}H NMR spectrum of **5**.

	Parameter	Value
1	Origin	Bruker BioSpin GmbH
2	Instrument	spect
3	Solvent	CDCl3
4	Temperature	298.0
5	Pulse Sequence	zgpg30
6	Experiment	1D
7	Number of Scans	2048
8	Receiver Gain	196.9
9	Relaxation Delay	2.0000
10	Pulse Width	10.0000
11	Spectrometer Frequency	100.67
12	Spectral Width	24038.5
13	Lowest Frequency	-1906.8
14	Nucleus	13C
15	Acquired Size	32768
16	Spectral Size	65536
17	Digital Resolution	0.37

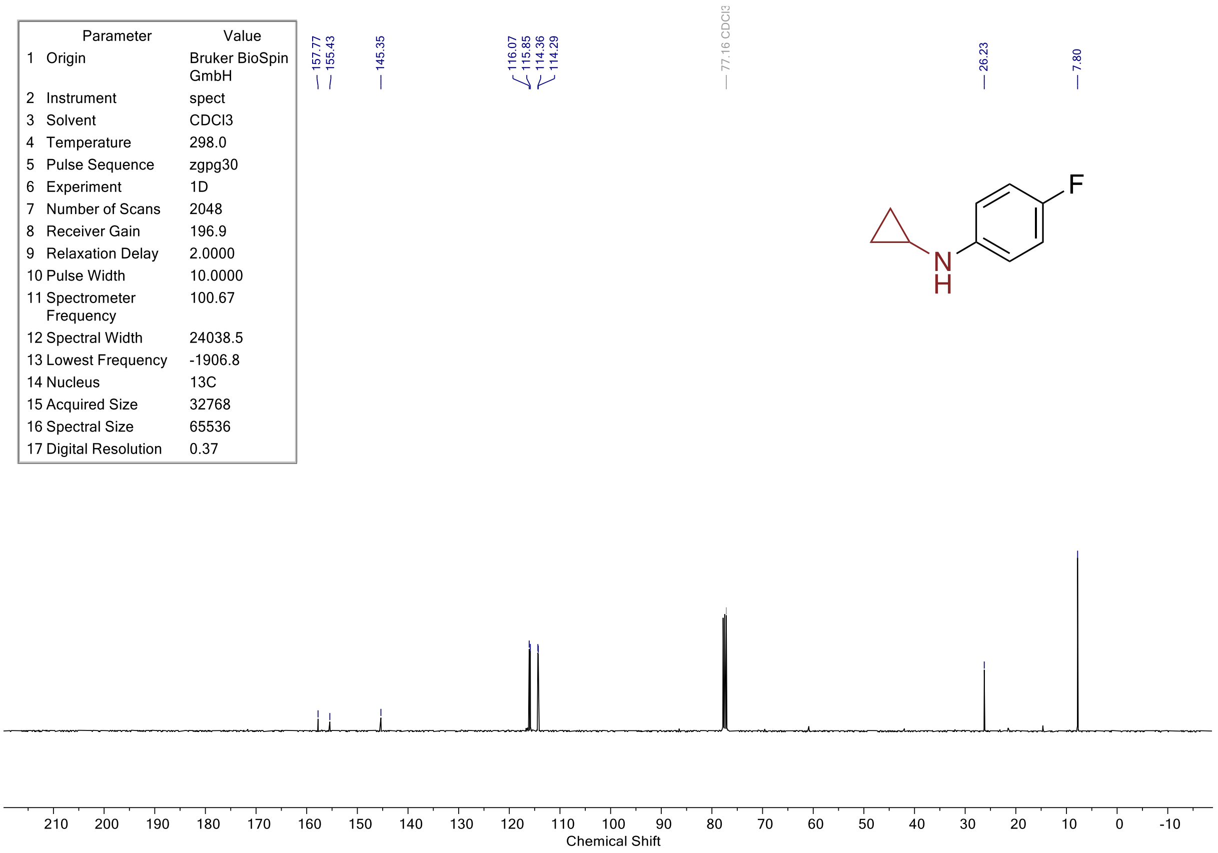

Figure S30. $^{13}C\{^{1}H\}$ NMR spectrum of **5**.

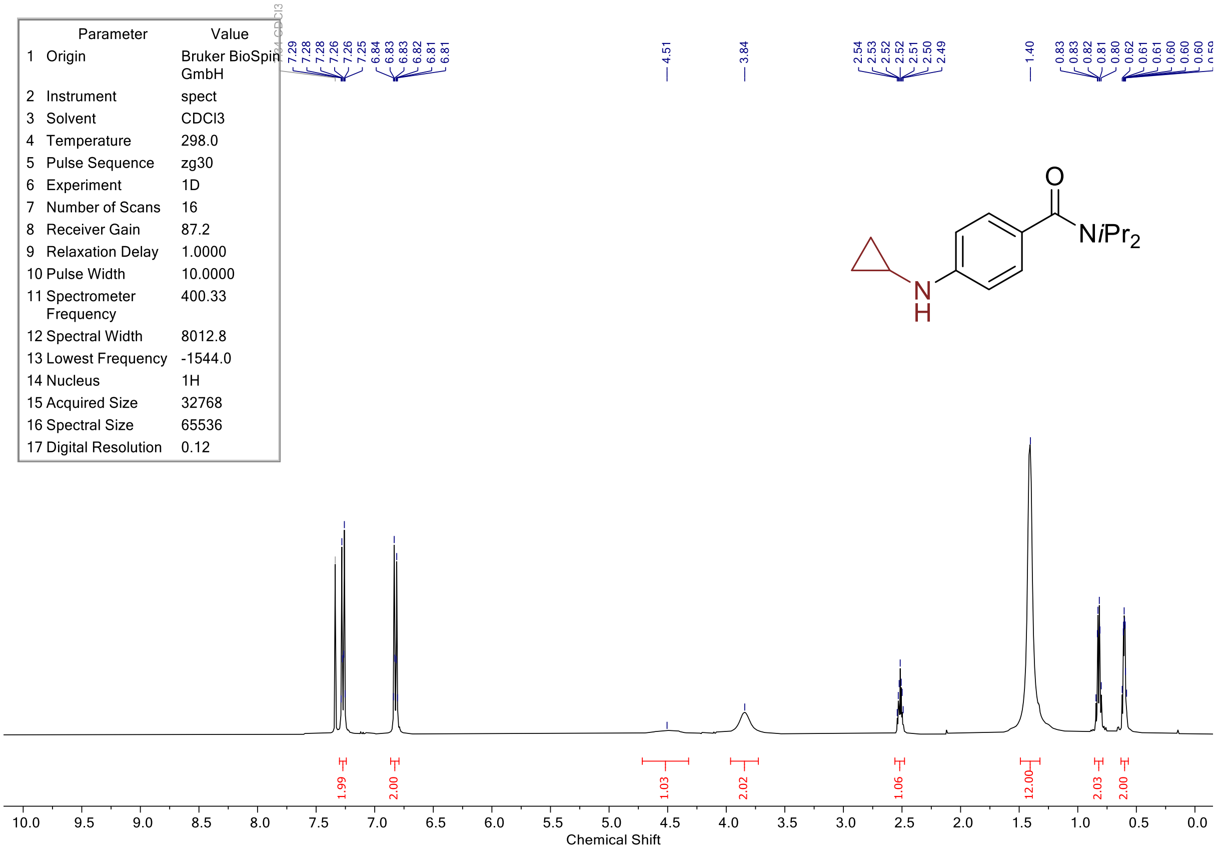

Figure S31. ^{1}H NMR spectrum of **6**.

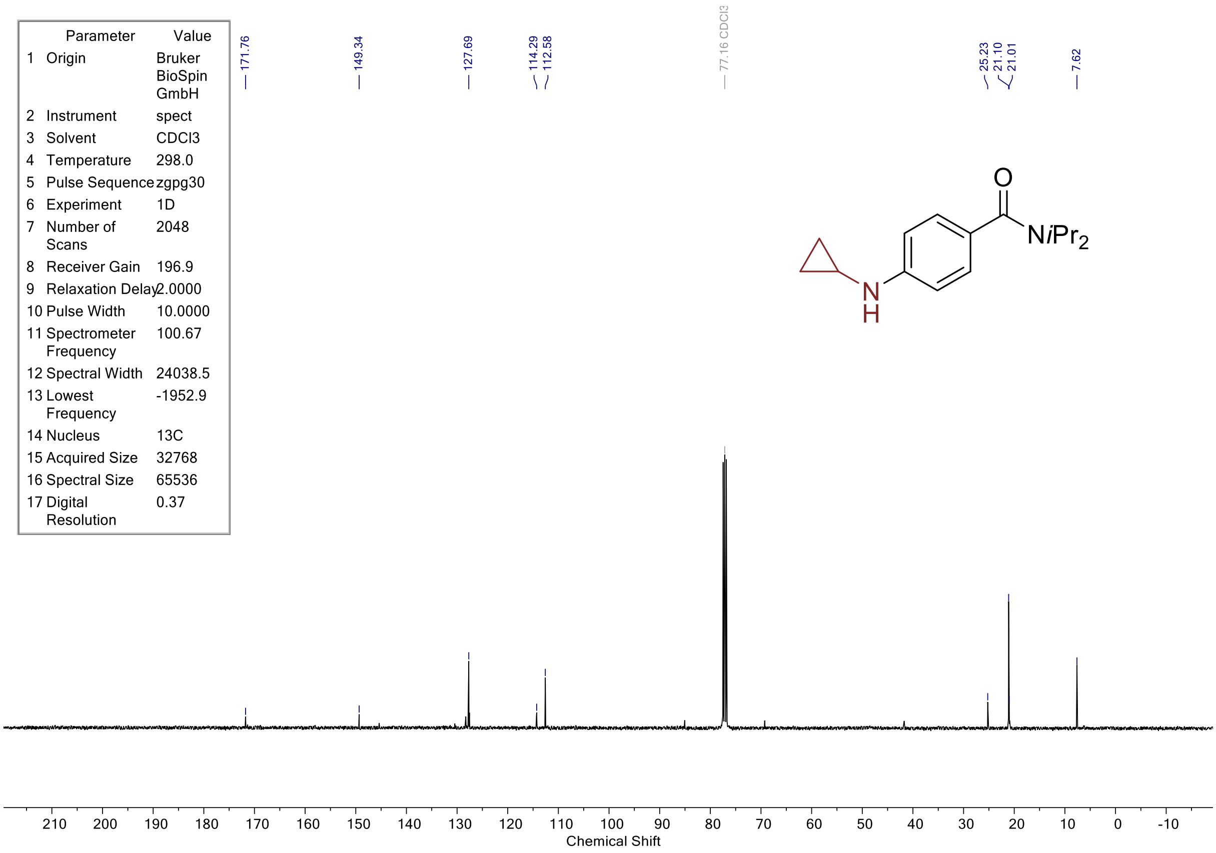

Figure S32. ^{13}C{^{1}H} NMR spectrum of **6**.

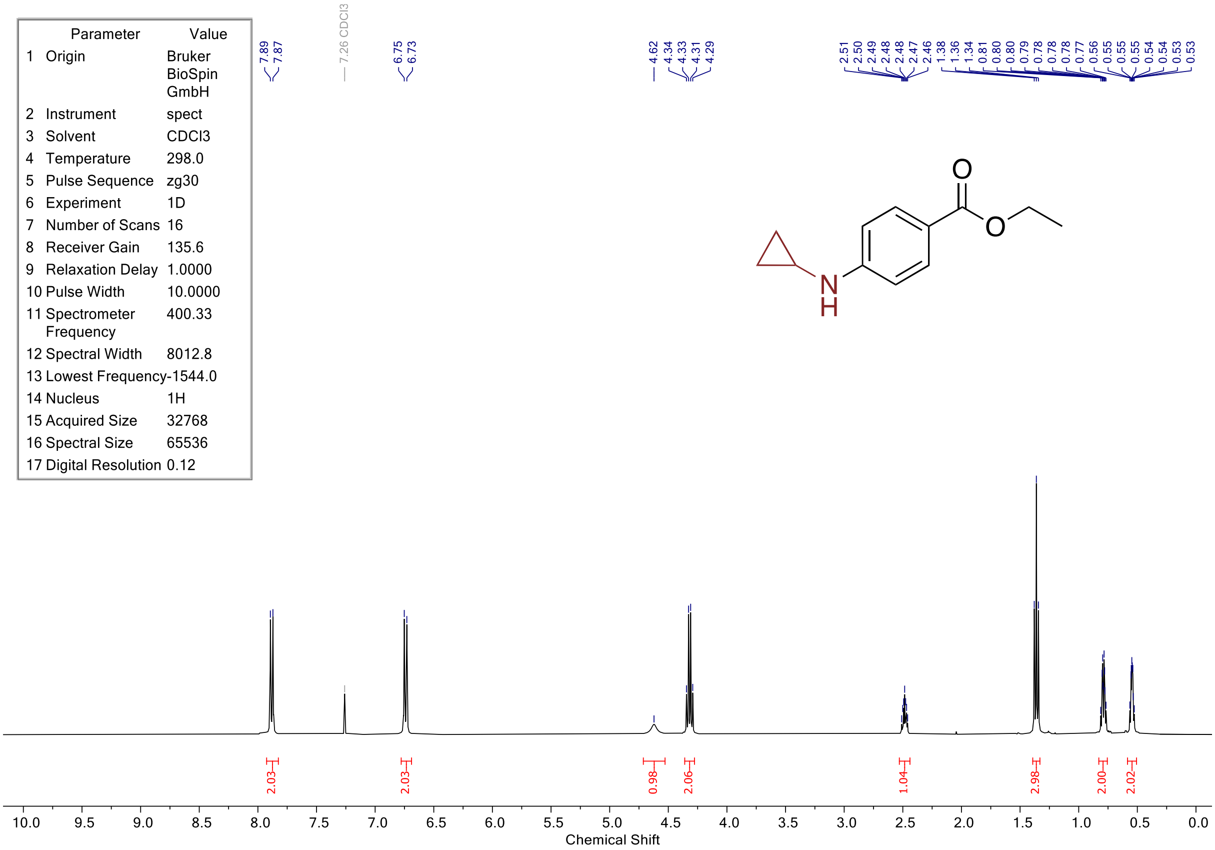

Figure S33. ^{1}H NMR spectrum of **7**.

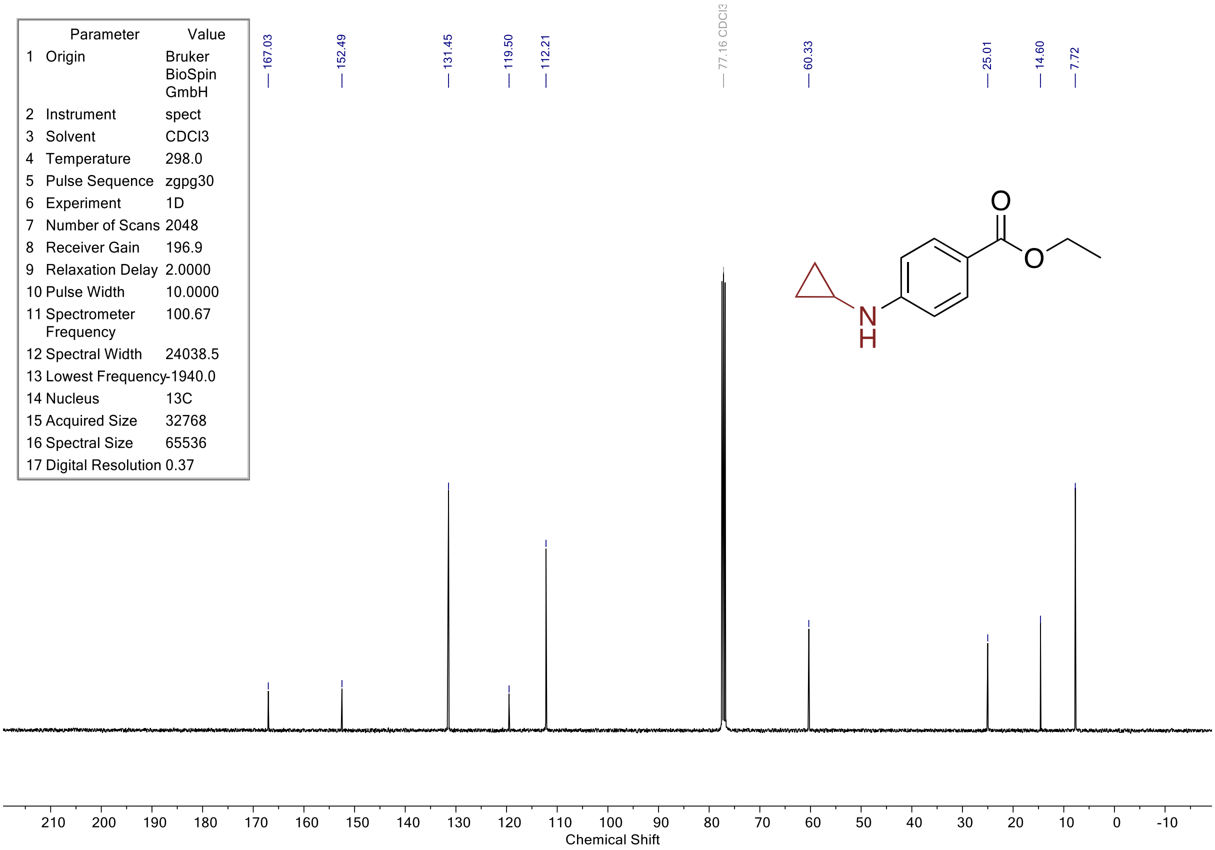

Figure S34. ^{13}C{^{1}H} NMR spectrum of **7**.

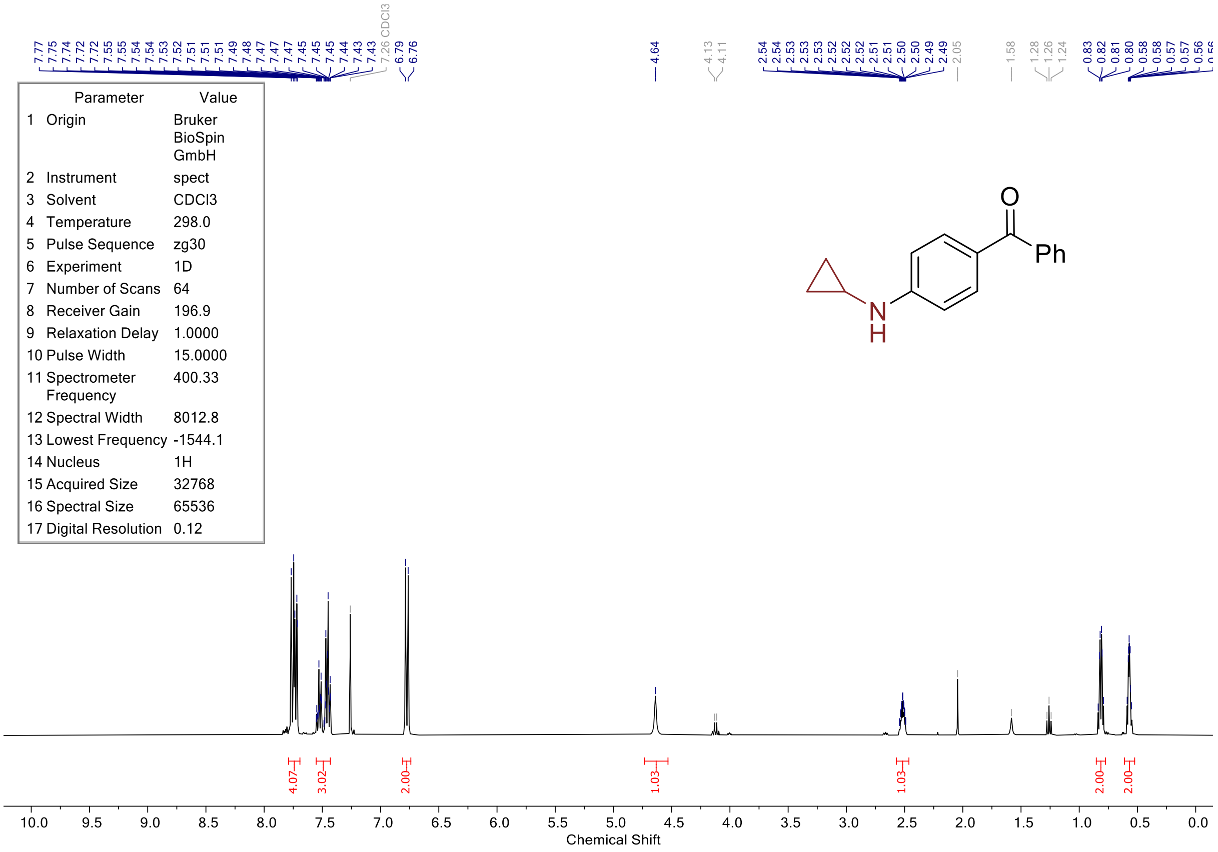

Figure S35. ^{1}H NMR spectrum of **8**.

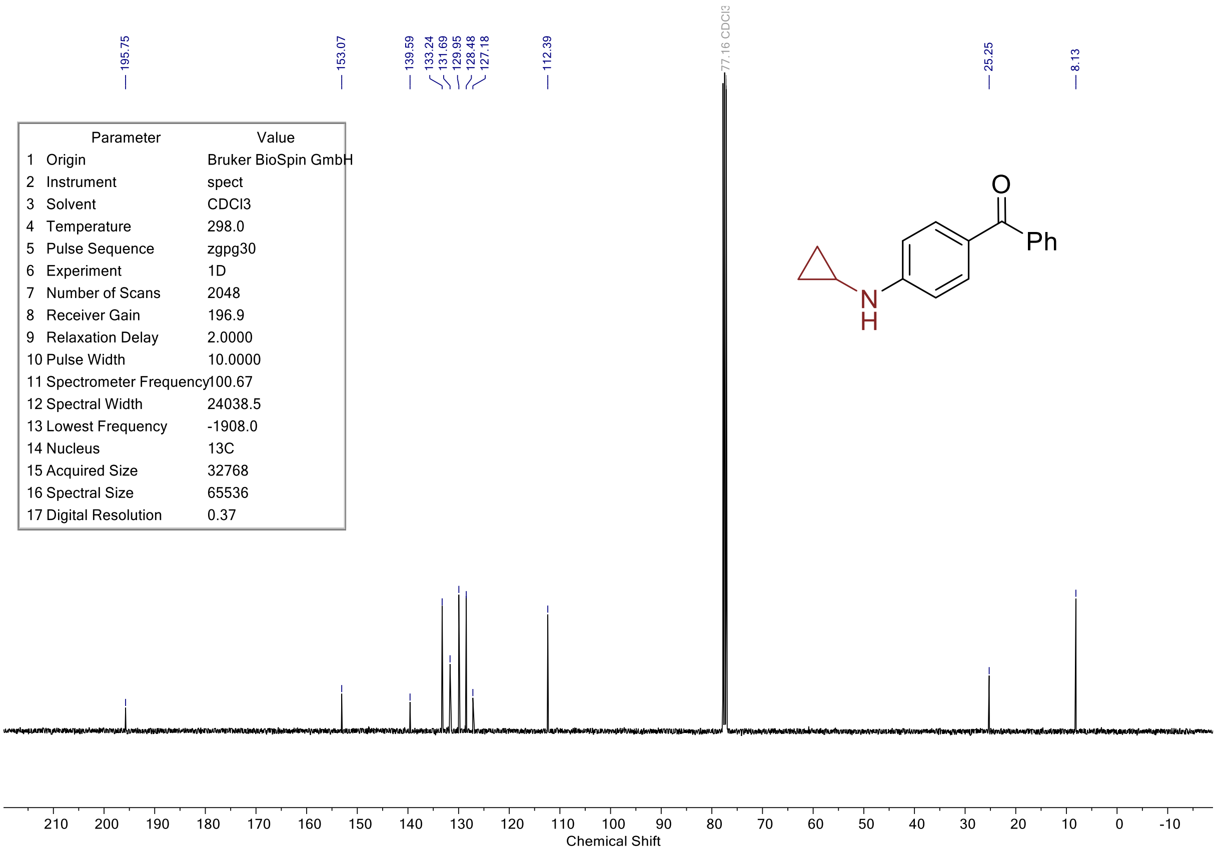

Figure S36. $^{13}C\{^{1}H\}$ NMR spectrum of **8**.

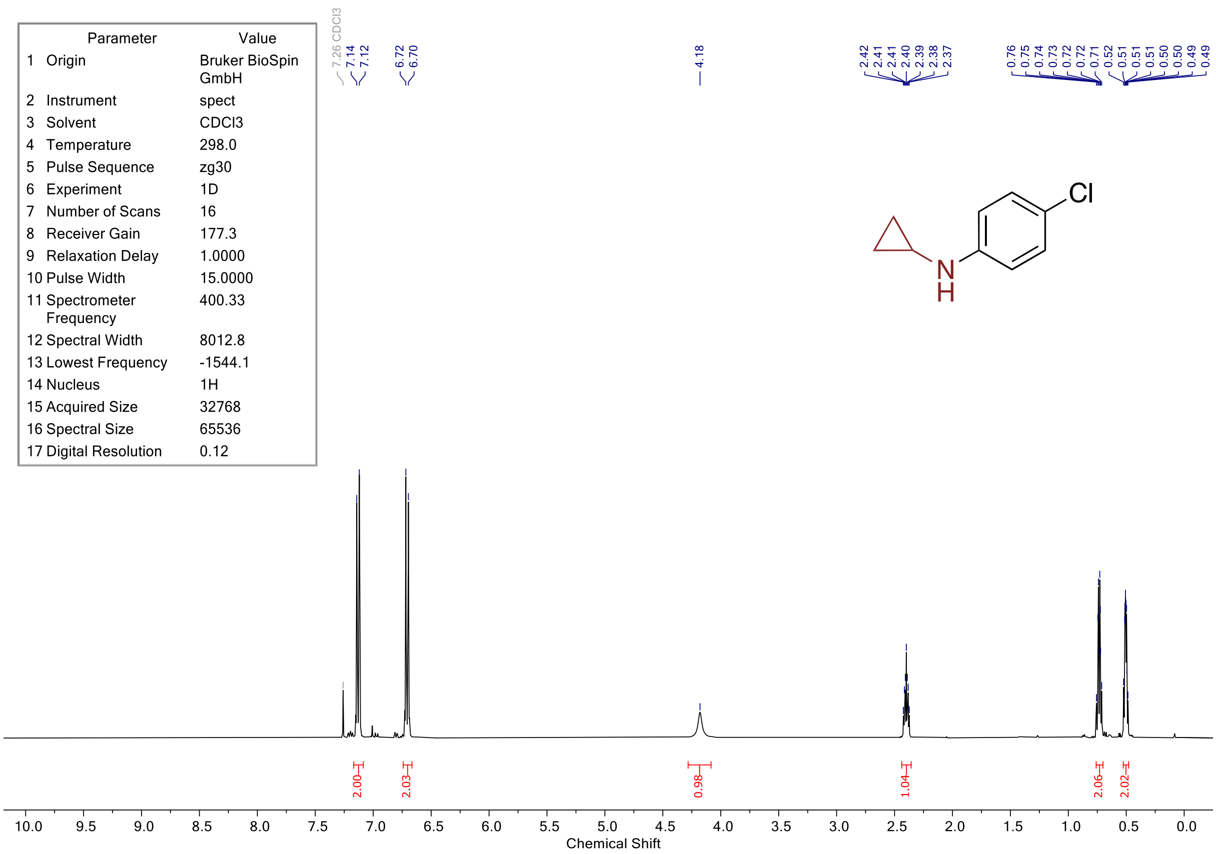

	Parameter	Value
1	Origin	Bruker BioSpin GmbH
2	Instrument	spect
3	Solvent	CDCl3
4	Temperature	298.0
5	Pulse Sequence	zg30
6	Experiment	1D
7	Number of Scans	16
8	Receiver Gain	177.3
9	Relaxation Delay	1.0000
10	Pulse Width	15.0000
11	Spectrometer Frequency	400.33
12	Spectral Width	8012.8
13	Lowest Frequency	-1544.1
14	Nucleus	1H
15	Acquired Size	32768
16	Spectral Size	65536
17	Digital Resolution	0.12

Figure S37. ^{1}H NMR spectrum of **9**.

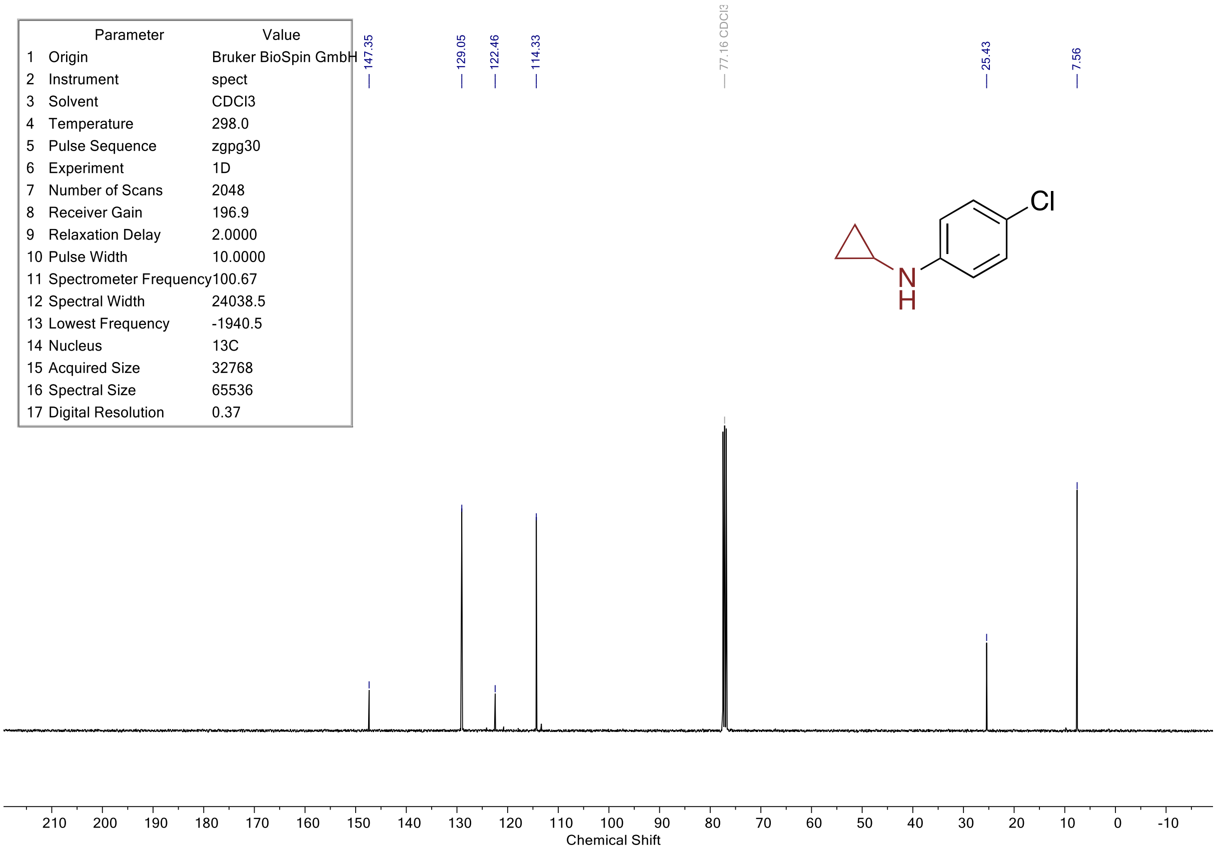

	Parameter	Value
1	Origin	Bruker BioSpin GmbH
2	Instrument	spect
3	Solvent	CDCl3
4	Temperature	298.0
5	Pulse Sequence	zgpg30
6	Experiment	1D
7	Number of Scans	2048
8	Receiver Gain	196.9
9	Relaxation Delay	2.0000
10	Pulse Width	10.0000
11	Spectrometer Frequency	100.67
12	Spectral Width	24038.5
13	Lowest Frequency	-1940.5
14	Nucleus	13C
15	Acquired Size	32768
16	Spectral Size	65536
17	Digital Resolution	0.37

Figure S38. $^{13}C\{^{1}H\}$ NMR spectrum of **9**.

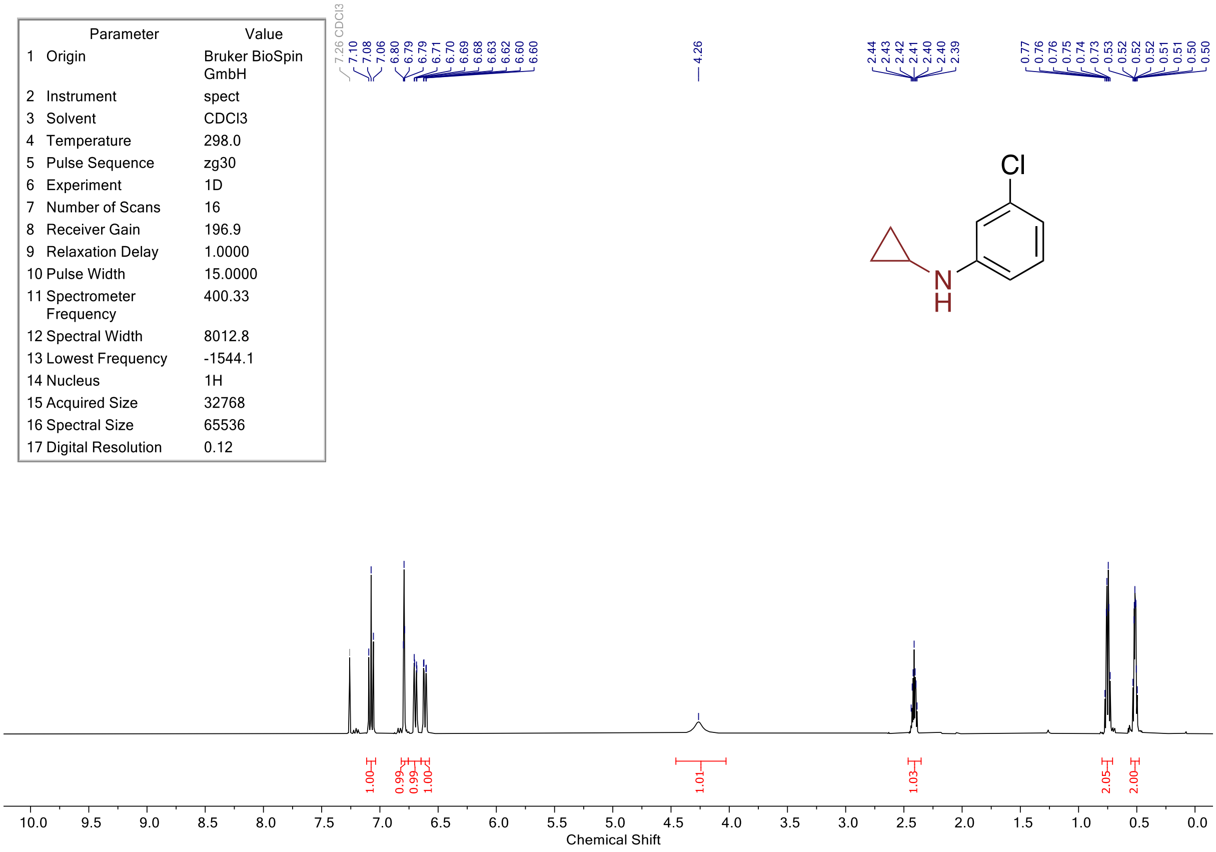

Parameter	Value
1 Origin	Bruker BioSpin GmbH
2 Instrument	spect
3 Solvent	CDCl3
4 Temperature	298.0
5 Pulse Sequence	zg30
6 Experiment	1D
7 Number of Scans	16
8 Receiver Gain	196.9
9 Relaxation Delay	1.0000
10 Pulse Width	15.0000
11 Spectrometer Frequency	400.33
12 Spectral Width	8012.8
13 Lowest Frequency	-1544.1
14 Nucleus	1H
15 Acquired Size	32768
16 Spectral Size	65536
17 Digital Resolution	0.12

Figure S39. ^{1}H NMR spectrum of **10**.

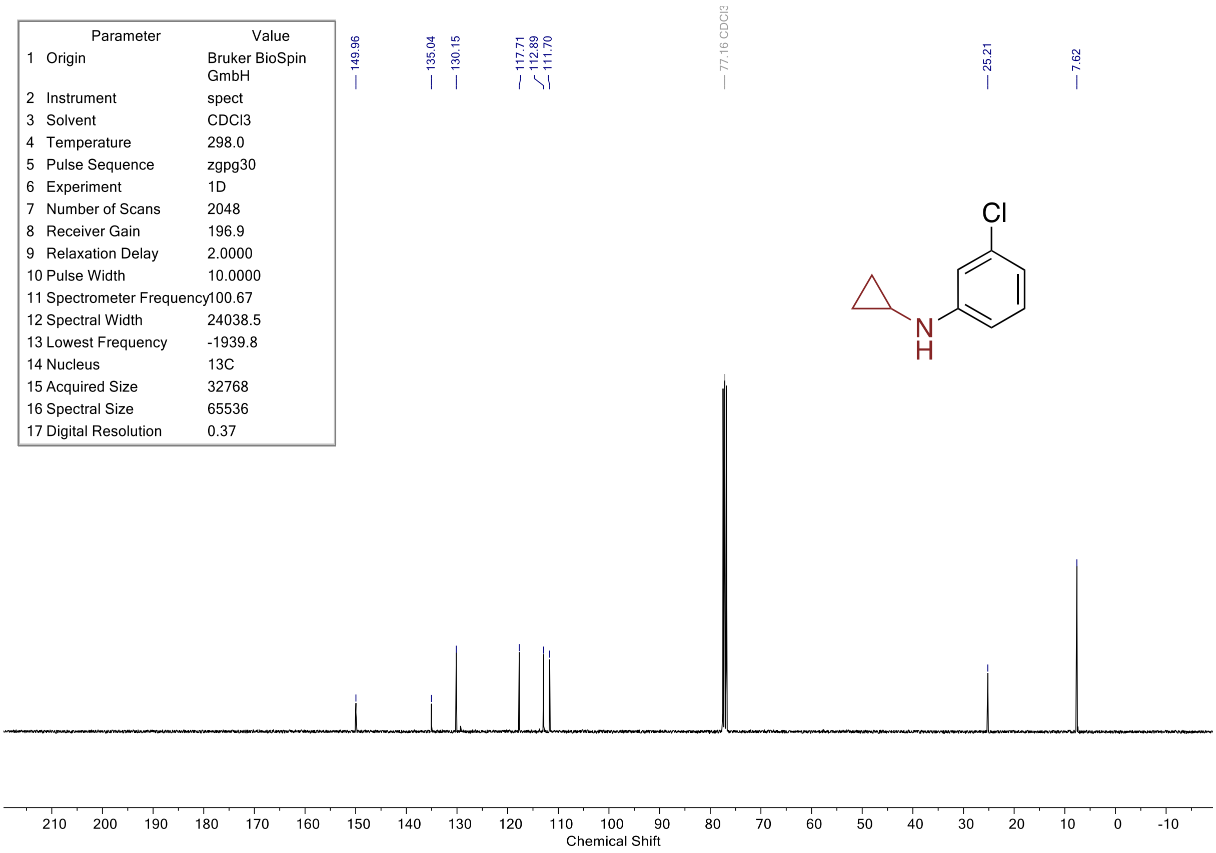

Parameter	Value
1 Origin	Bruker BioSpin GmbH
2 Instrument	spect
3 Solvent	CDCl3
4 Temperature	298.0
5 Pulse Sequence	zgpg30
6 Experiment	1D
7 Number of Scans	2048
8 Receiver Gain	196.9
9 Relaxation Delay	2.0000
10 Pulse Width	10.0000
11 Spectrometer Frequency	100.67
12 Spectral Width	24038.5
13 Lowest Frequency	-1939.8
14 Nucleus	13C
15 Acquired Size	32768
16 Spectral Size	65536
17 Digital Resolution	0.37

Figure S40. ^{13}C{^{1}H} NMR spectrum of **10**.

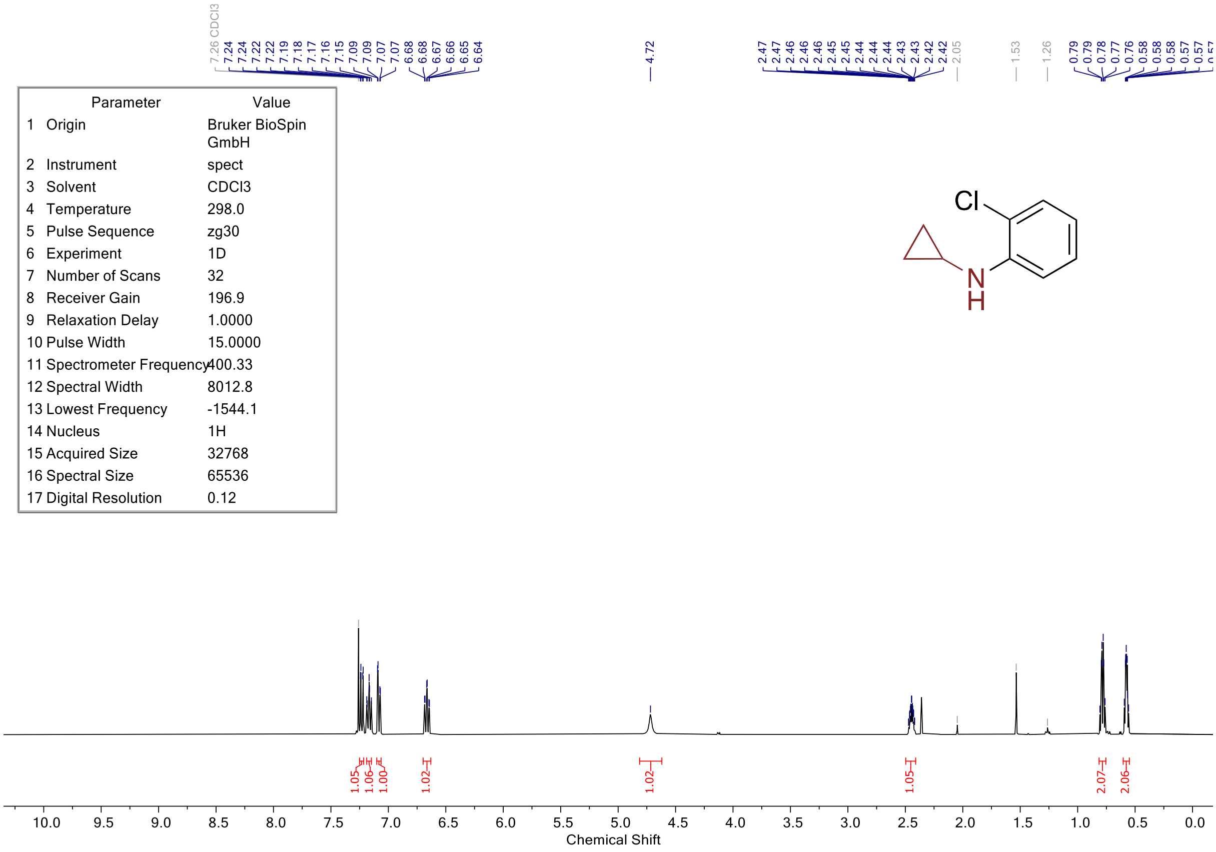

	Parameter	Value
1	Origin	Bruker BioSpin GmbH
2	Instrument	spect
3	Solvent	CDCl3
4	Temperature	298.0
5	Pulse Sequence	zg30
6	Experiment	1D
7	Number of Scans	32
8	Receiver Gain	196.9
9	Relaxation Delay	1.0000
10	Pulse Width	15.0000
11	Spectrometer Frequency	400.33
12	Spectral Width	8012.8
13	Lowest Frequency	-1544.1
14	Nucleus	1H
15	Acquired Size	32768
16	Spectral Size	65536
17	Digital Resolution	0.12

Figure S41. ^{1}H NMR spectrum of **11**.

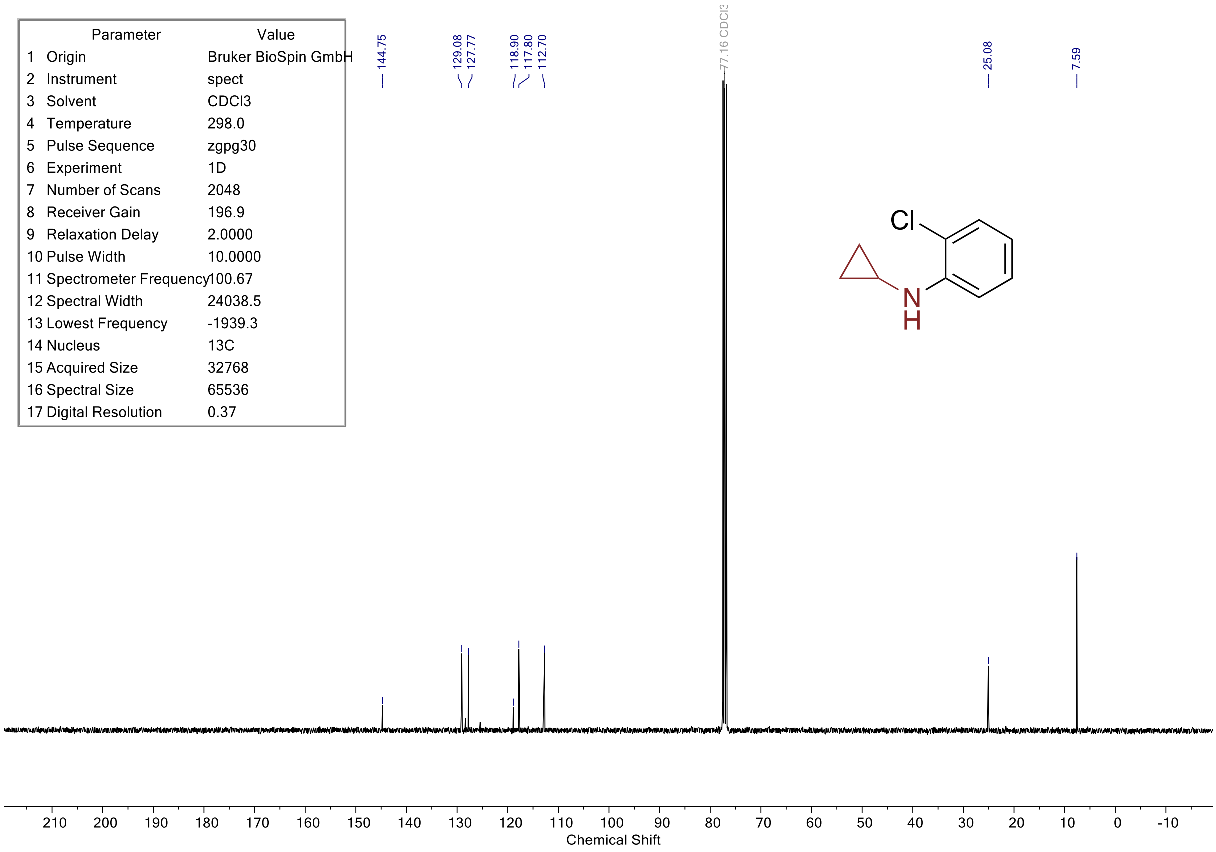

	Parameter	Value
1	Origin	Bruker BioSpin GmbH
2	Instrument	spect
3	Solvent	CDCl3
4	Temperature	298.0
5	Pulse Sequence	zgpg30
6	Experiment	1D
7	Number of Scans	2048
8	Receiver Gain	196.9
9	Relaxation Delay	2.0000
10	Pulse Width	10.0000
11	Spectrometer Frequency	100.67
12	Spectral Width	24038.5
13	Lowest Frequency	-1939.3
14	Nucleus	13C
15	Acquired Size	32768
16	Spectral Size	65536
17	Digital Resolution	0.37

Figure S42. $^{13}C\{^{1}H\}$ NMR spectrum of **11**.

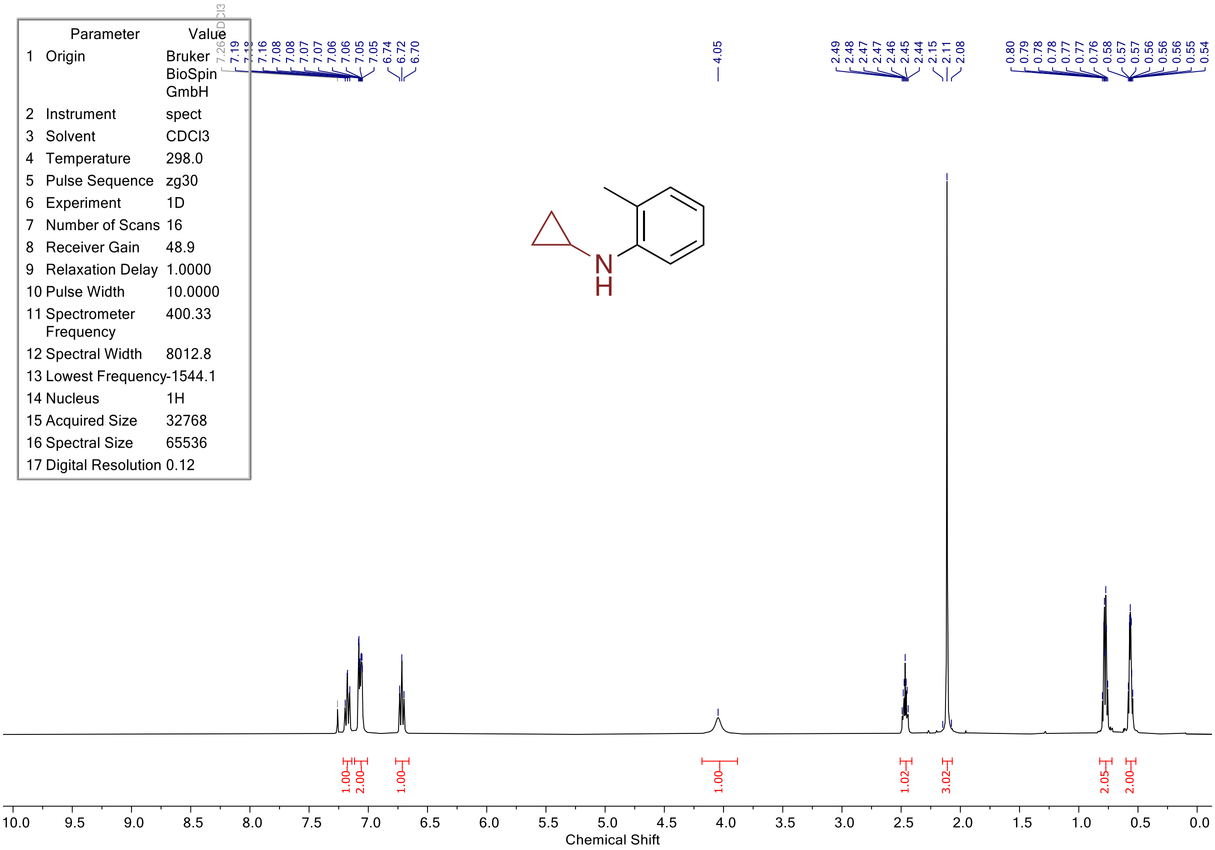

Figure S43. ^{1}H NMR spectrum of **12**.

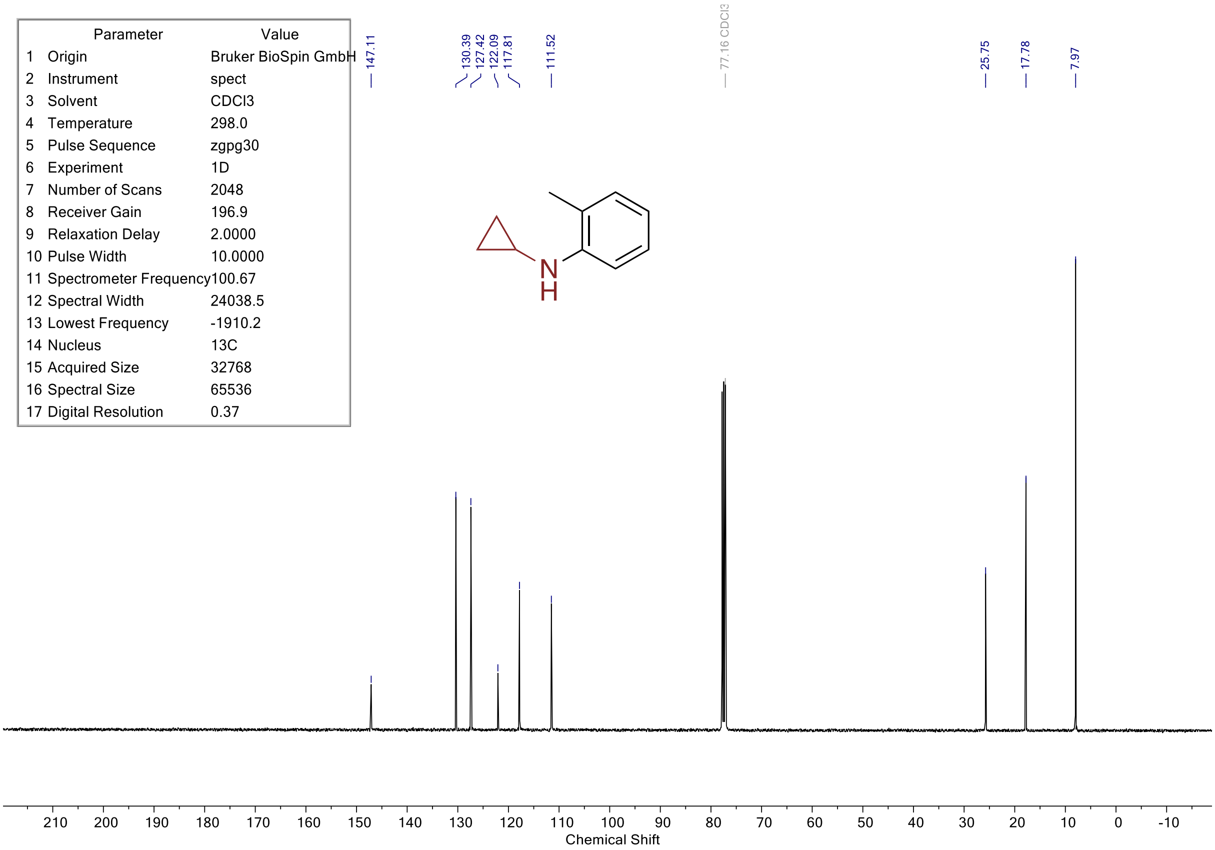

Figure S44. ^{13}C{^{1}H} NMR spectrum of **12**.

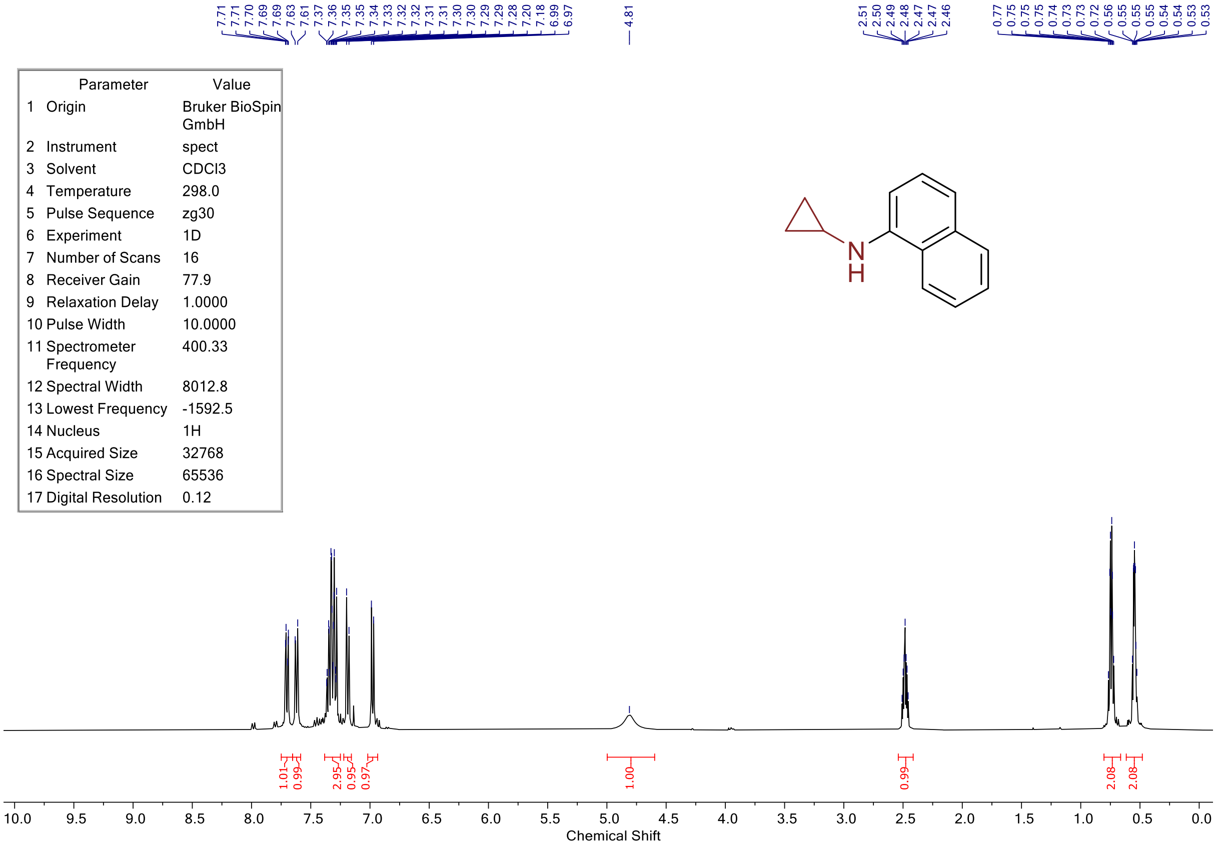

	Parameter	Value
1	Origin	Bruker BioSpin GmbH
2	Instrument	spect
3	Solvent	CDCl3
4	Temperature	298.0
5	Pulse Sequence	zg30
6	Experiment	1D
7	Number of Scans	16
8	Receiver Gain	77.9
9	Relaxation Delay	1.0000
10	Pulse Width	10.0000
11	Spectrometer Frequency	400.33
12	Spectral Width	8012.8
13	Lowest Frequency	-1592.5
14	Nucleus	1H
15	Acquired Size	32768
16	Spectral Size	65536
17	Digital Resolution	0.12

Figure S45. ^{1}H NMR spectrum of **13**.

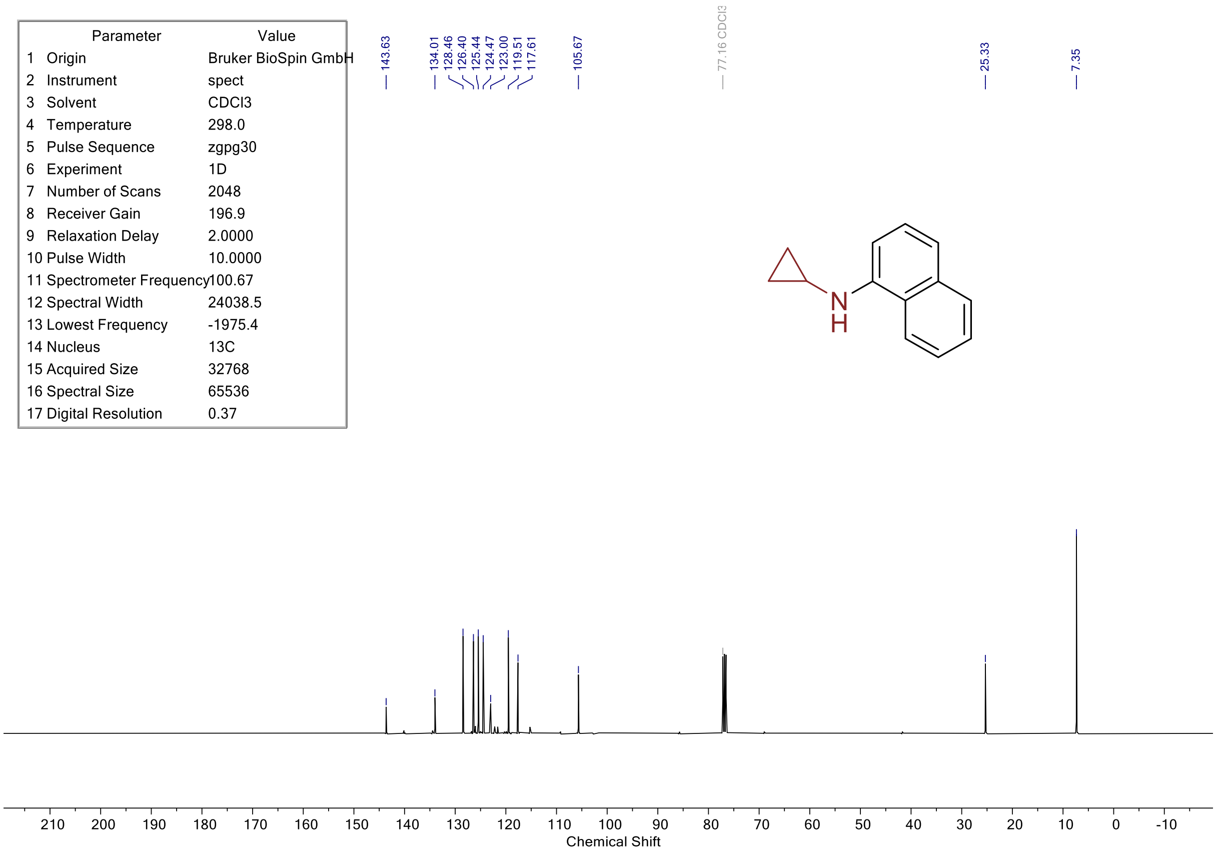

	Parameter	Value
1	Origin	Bruker BioSpin GmbH
2	Instrument	spect
3	Solvent	CDCl3
4	Temperature	298.0
5	Pulse Sequence	zgpg30
6	Experiment	1D
7	Number of Scans	2048
8	Receiver Gain	196.9
9	Relaxation Delay	2.0000
10	Pulse Width	10.0000
11	Spectrometer Frequency	100.67
12	Spectral Width	24038.5
13	Lowest Frequency	-1975.4
14	Nucleus	13C
15	Acquired Size	32768
16	Spectral Size	65536
17	Digital Resolution	0.37

Figure S46. ^{13}C{^{1}H} NMR spectrum of **13**.

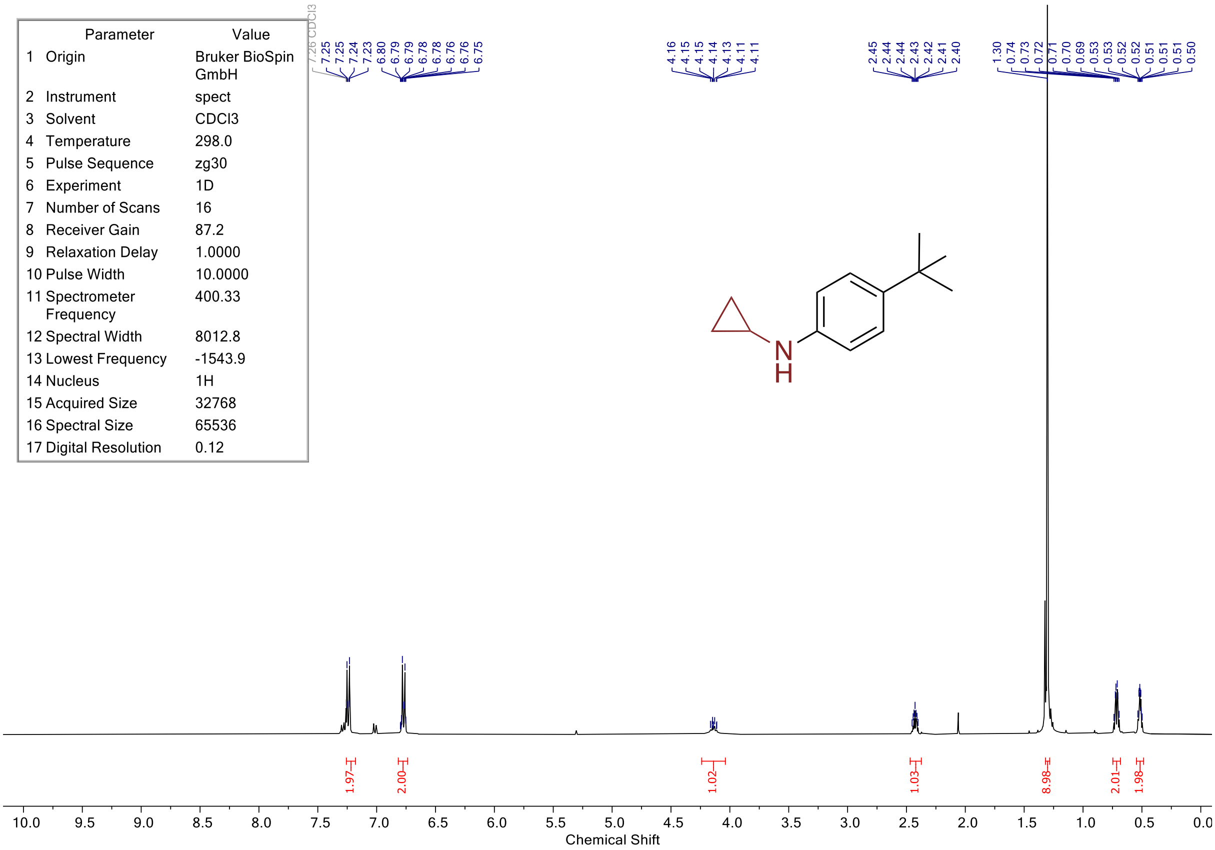

Figure S47. ^{1}H NMR spectrum of **14**.

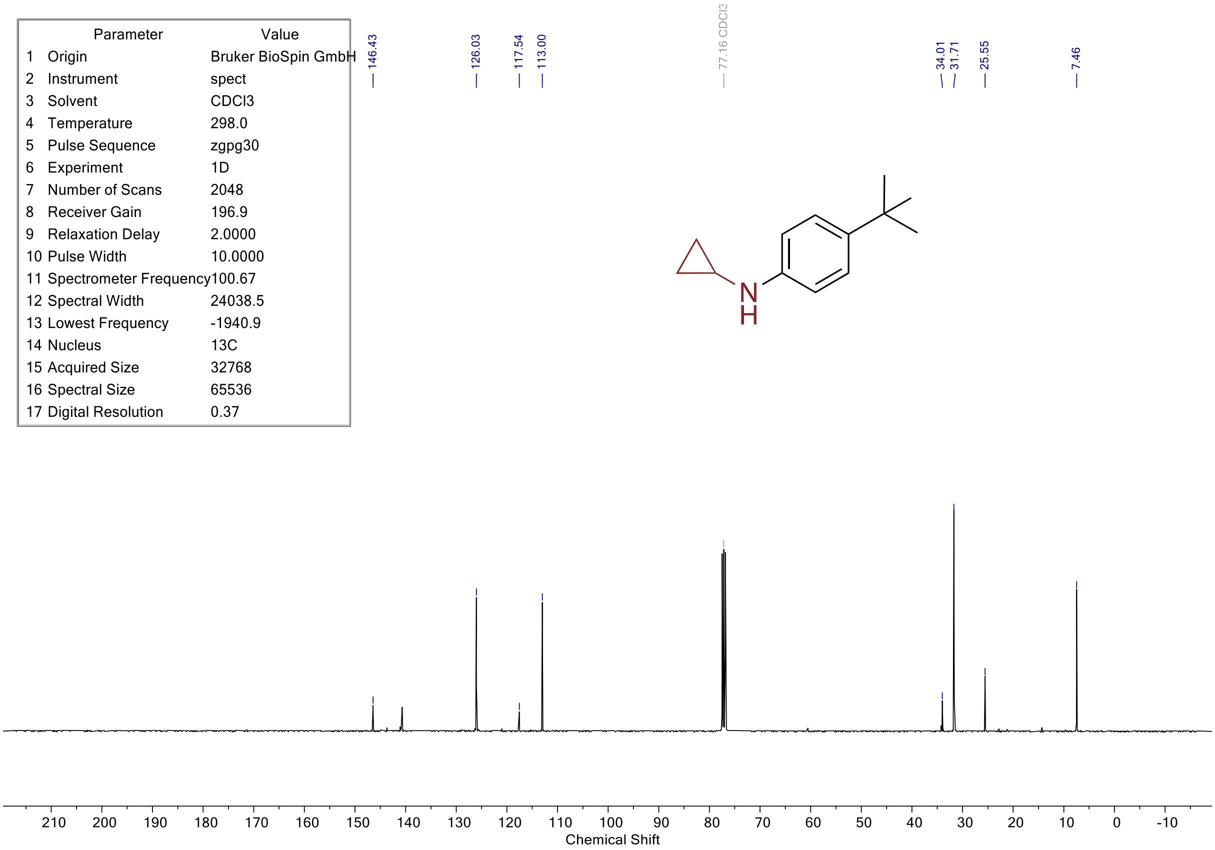

Figure S48. $^{13}C\{^{1}H\}$ NMR spectrum of **14**.

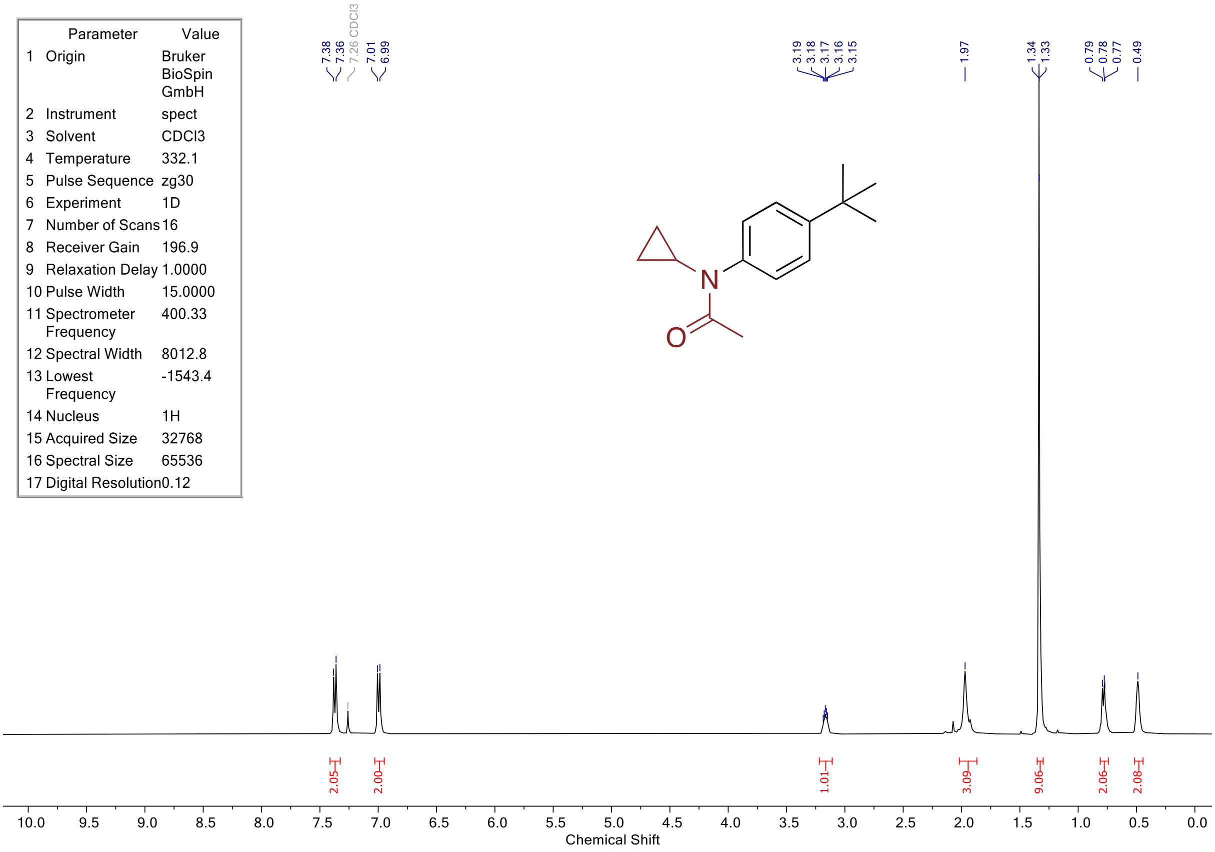

Figure S49. ^{1}H NMR spectrum of **15**.

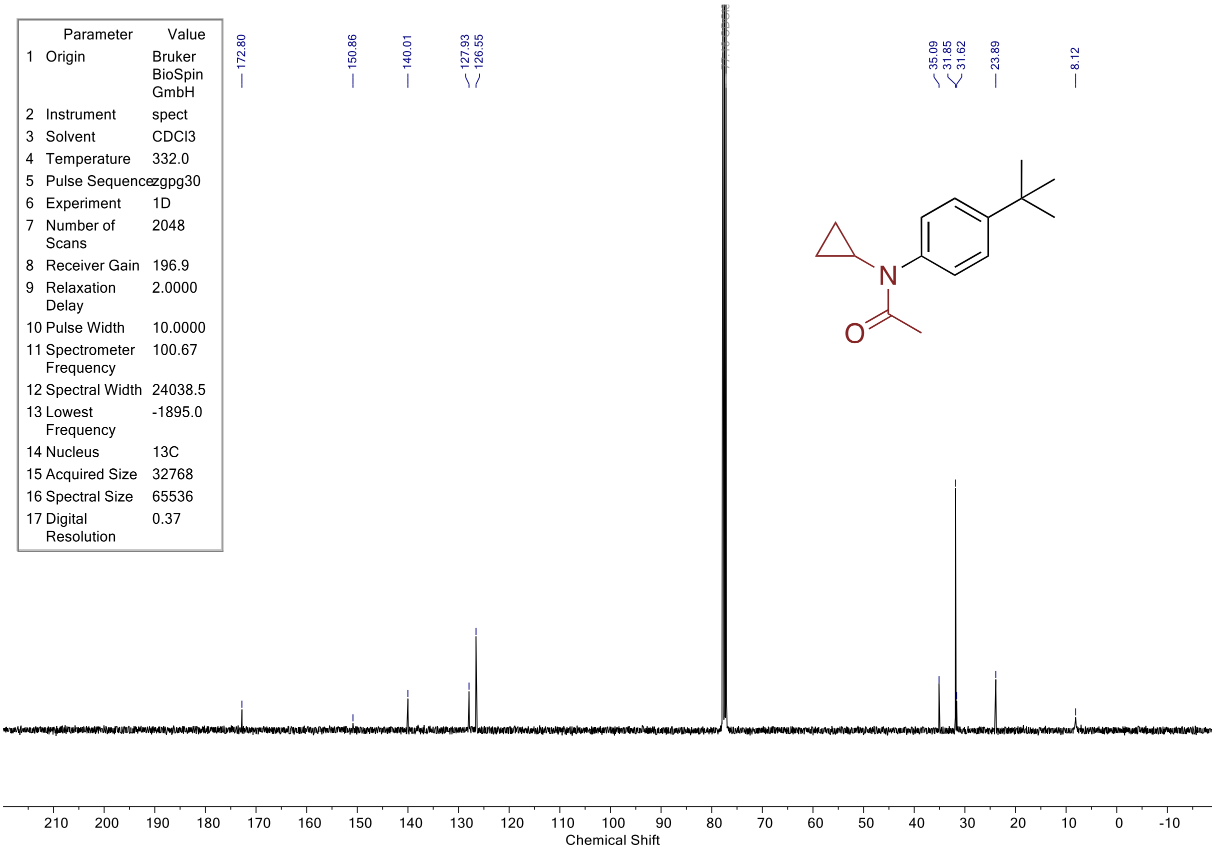

Figure S50. $^{13}C\{^1H\}$ NMR spectrum of **15**.

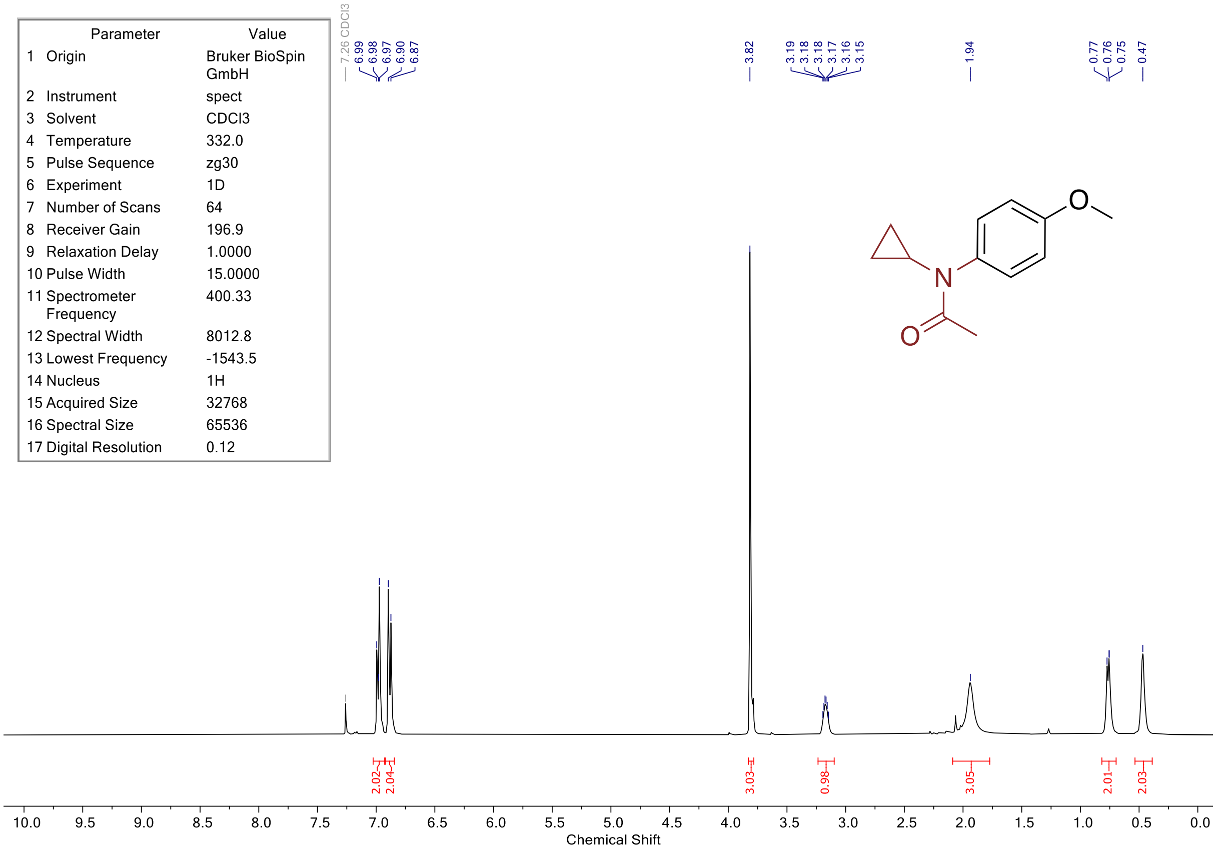

	Parameter	Value
1	Origin	Bruker BioSpin GmbH
2	Instrument	spect
3	Solvent	CDCl3
4	Temperature	332.0
5	Pulse Sequence	zg30
6	Experiment	1D
7	Number of Scans	64
8	Receiver Gain	196.9
9	Relaxation Delay	1.0000
10	Pulse Width	15.0000
11	Spectrometer Frequency	400.33
12	Spectral Width	8012.8
13	Lowest Frequency	-1543.5
14	Nucleus	1H
15	Acquired Size	32768
16	Spectral Size	65536
17	Digital Resolution	0.12

Figure S51. ^{1}H NMR spectrum of **16**.

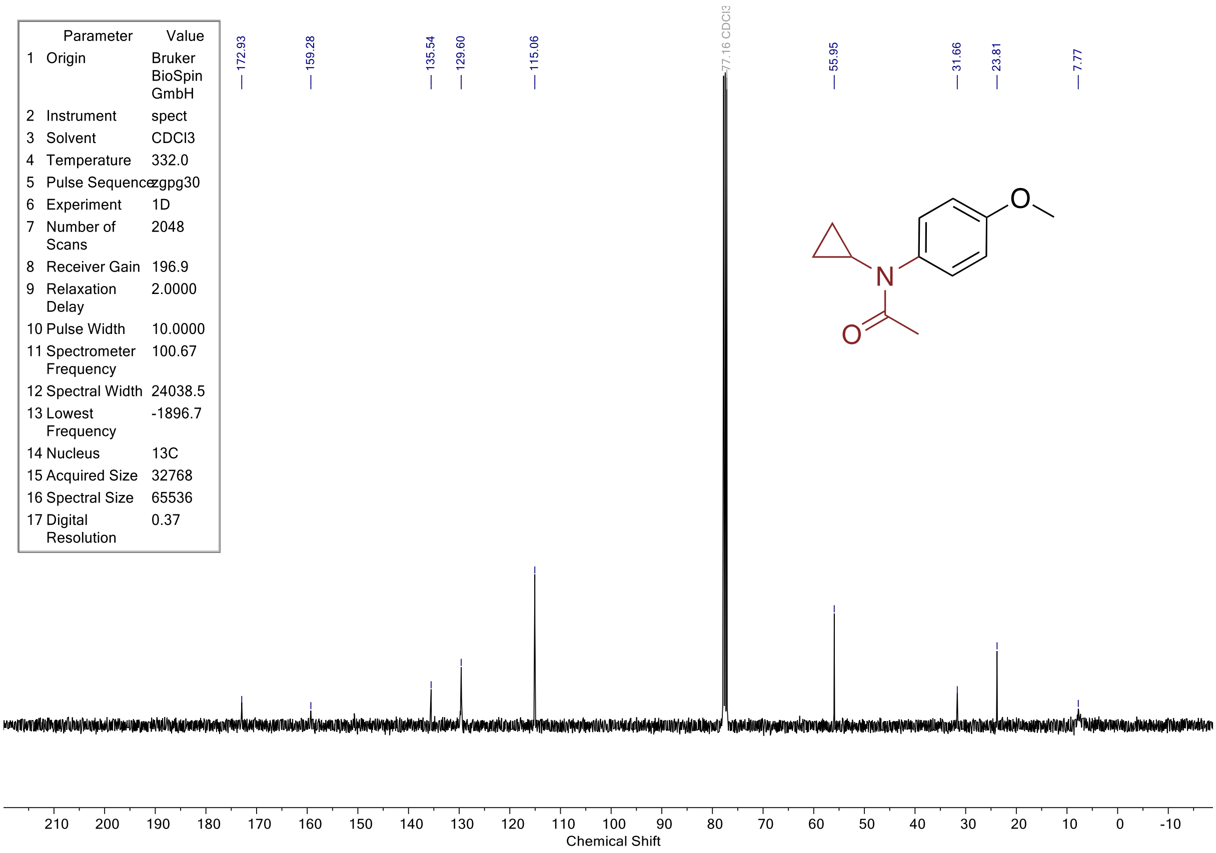

	Parameter	Value
1	Origin	Bruker BioSpin GmbH
2	Instrument	spect
3	Solvent	CDCl3
4	Temperature	332.0
5	Pulse Sequence	zgpg30
6	Experiment	1D
7	Number of Scans	2048
8	Receiver Gain	196.9
9	Relaxation Delay	2.0000
10	Pulse Width	10.0000
11	Spectrometer Frequency	100.67
12	Spectral Width	24038.5
13	Lowest Frequency	-1896.7
14	Nucleus	13C
15	Acquired Size	32768
16	Spectral Size	65536
17	Digital Resolution	0.37

Figure S52. ^{13}C{^{1}H} NMR spectrum of **16**.

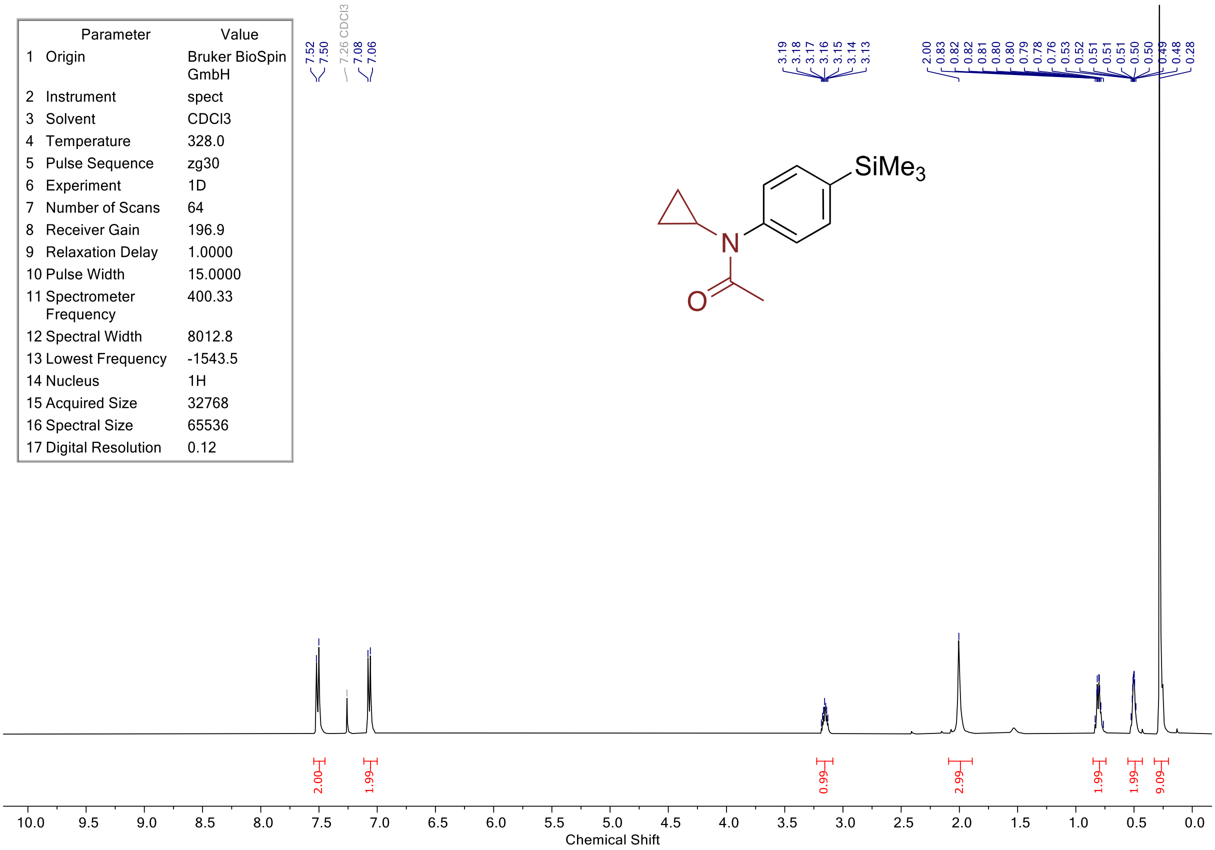

Figure S53. ^{1}H NMR spectrum of **17**.

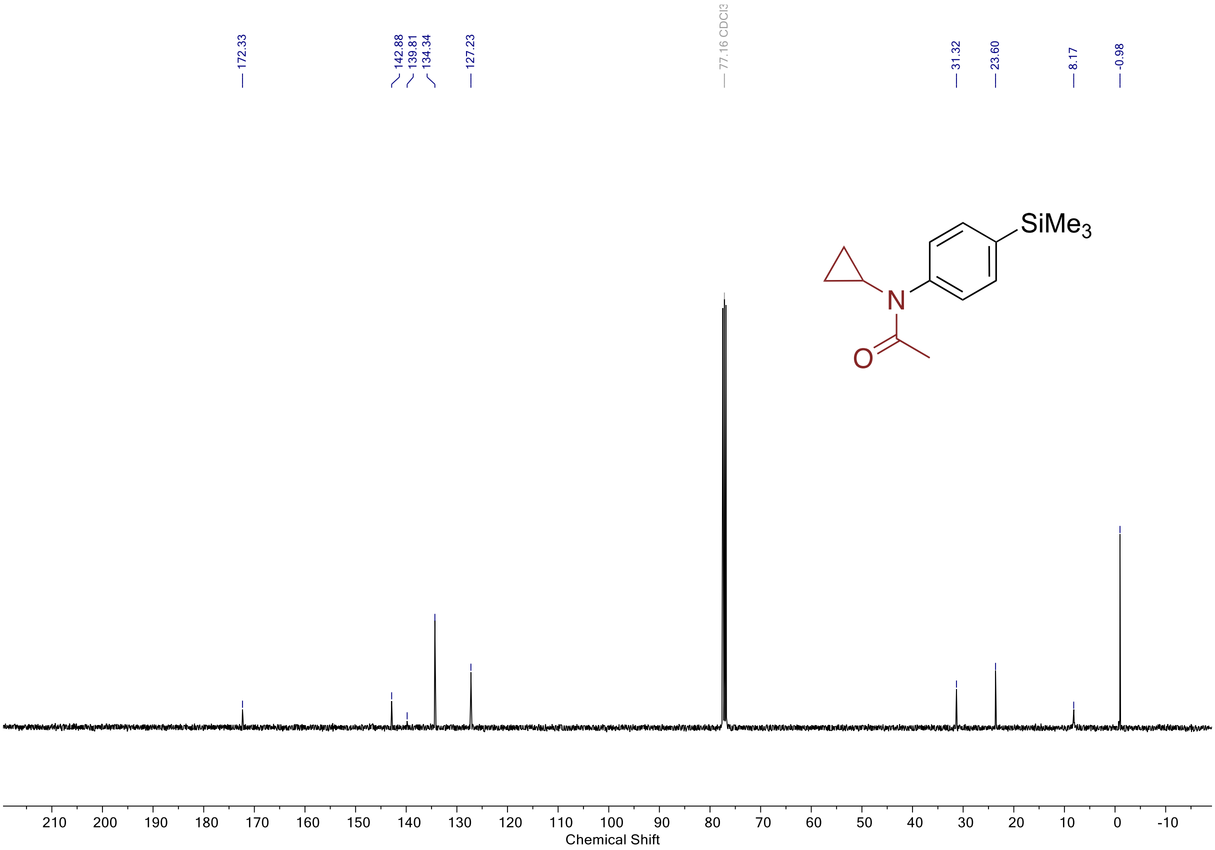

Figure S54. $^{13}C\{^{1}H\}$ NMR spectrum of **17**.

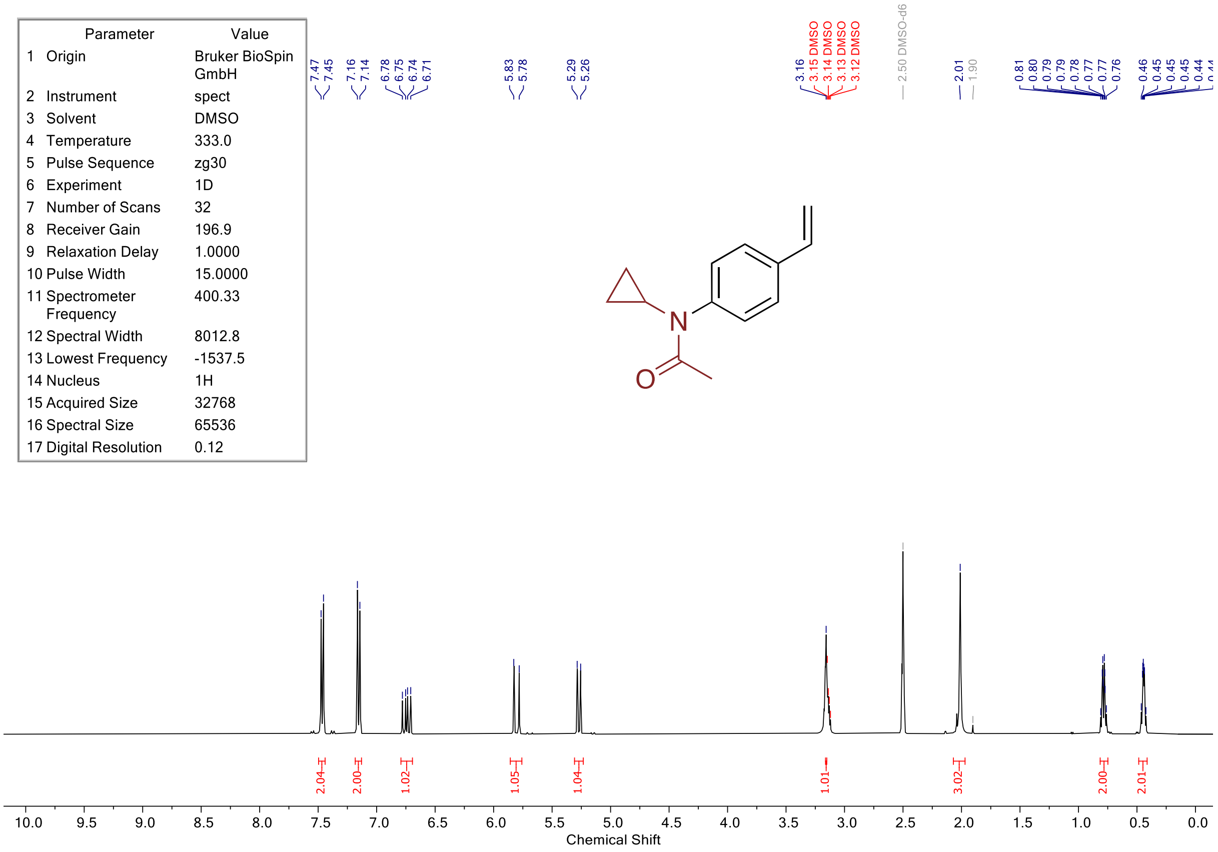

	Parameter	Value
1	Origin	Bruker BioSpin GmbH
2	Instrument	spect
3	Solvent	DMSO
4	Temperature	333.0
5	Pulse Sequence	zg30
6	Experiment	1D
7	Number of Scans	32
8	Receiver Gain	196.9
9	Relaxation Delay	1.0000
10	Pulse Width	15.0000
11	Spectrometer Frequency	400.33
12	Spectral Width	8012.8
13	Lowest Frequency	-1537.5
14	Nucleus	1H
15	Acquired Size	32768
16	Spectral Size	65536
17	Digital Resolution	0.12

Figure S55. ^{1}H NMR spectrum of **18**.

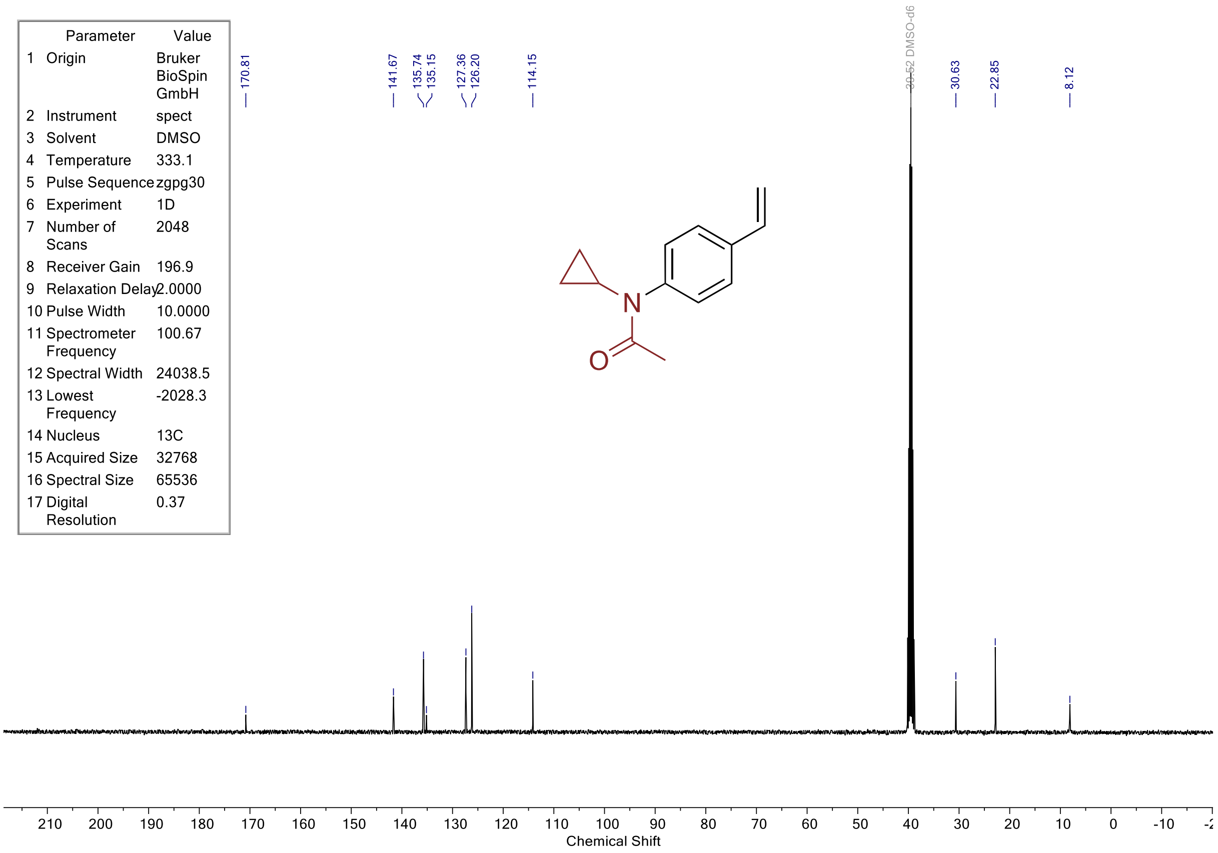

	Parameter	Value
1	Origin	Bruker BioSpin GmbH
2	Instrument	spect
3	Solvent	DMSO
4	Temperature	333.1
5	Pulse Sequence	zgpg30
6	Experiment	1D
7	Number of Scans	2048
8	Receiver Gain	196.9
9	Relaxation Delay	2.0000
10	Pulse Width	10.0000
11	Spectrometer Frequency	100.67
12	Spectral Width	24038.5
13	Lowest Frequency	-2028.3
14	Nucleus	13C
15	Acquired Size	32768
16	Spectral Size	65536
17	Digital Resolution	0.37

Figure S56. ^{13}C{^{1}H} NMR spectrum of **18**.

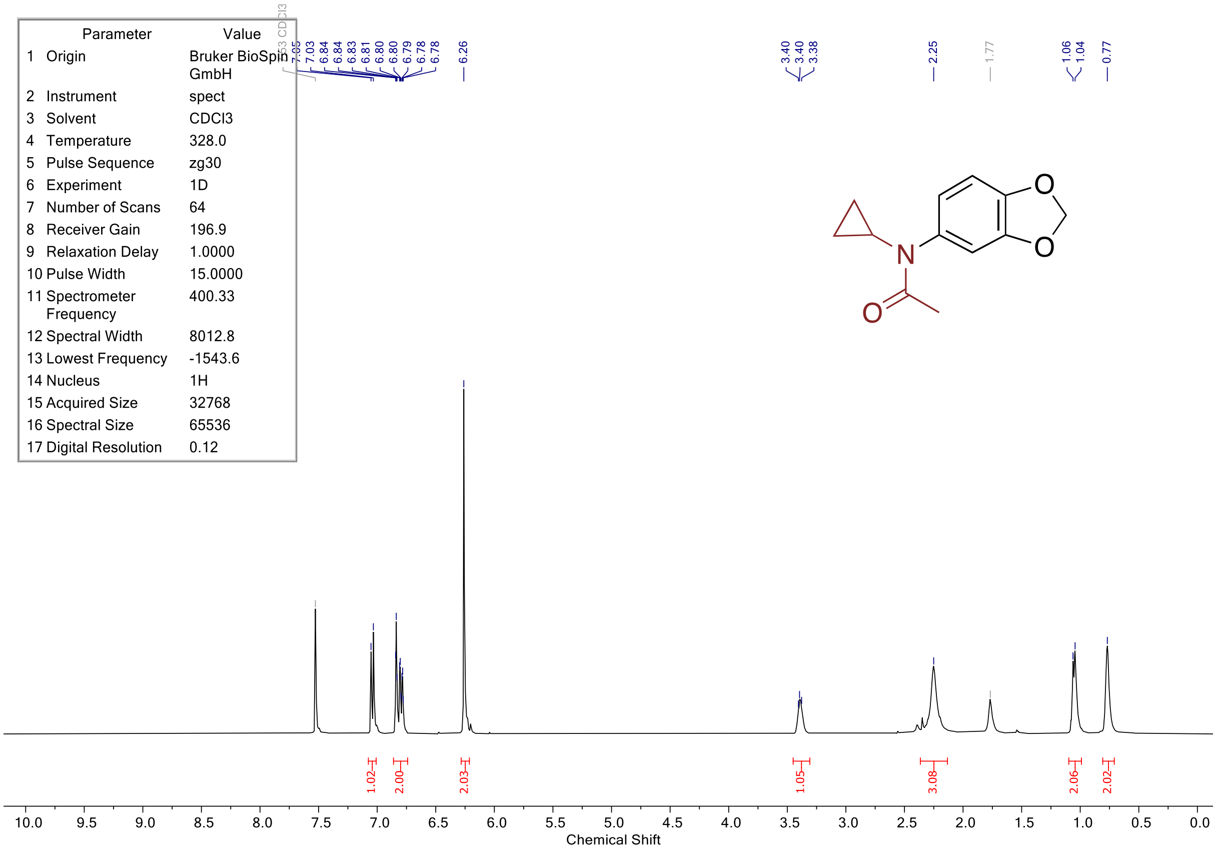

Figure S57. ^{1}H NMR spectrum of **19**.

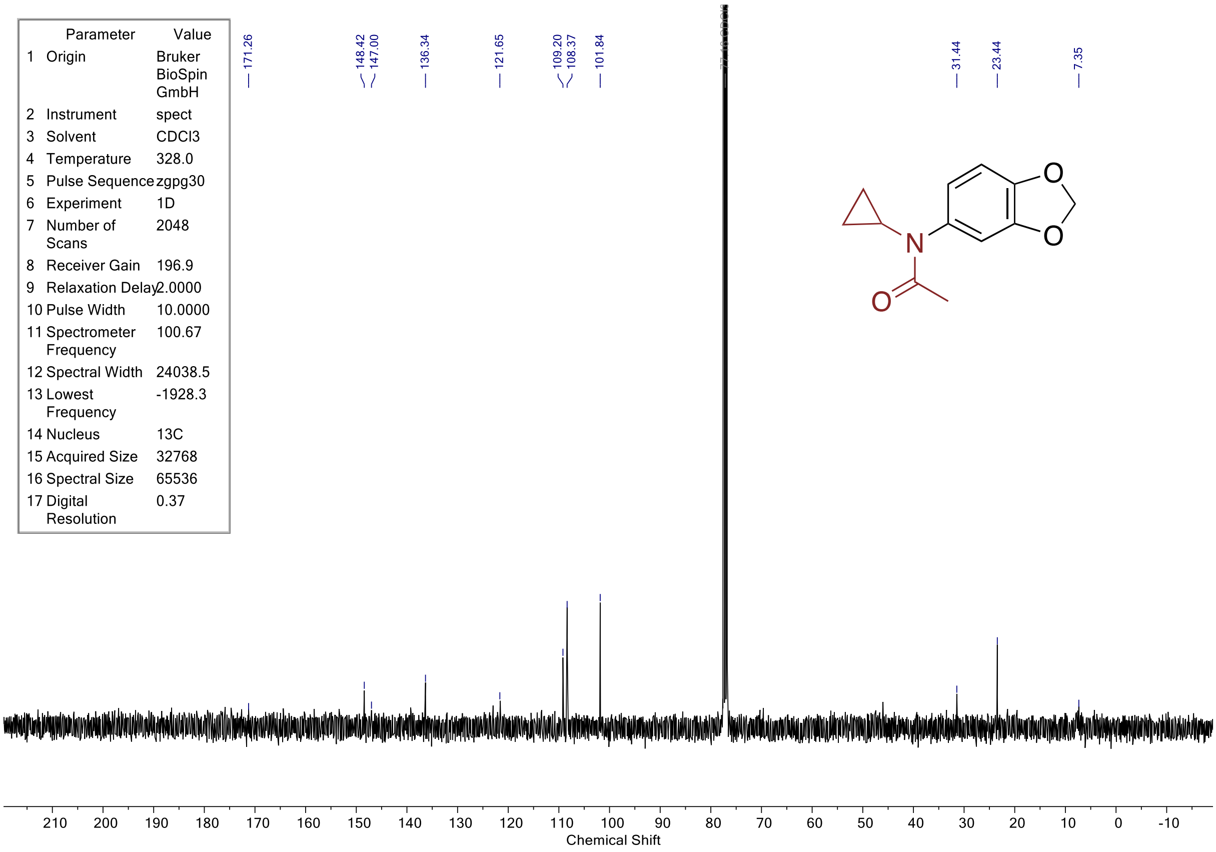

Figure S58. $^{13}C\{^{1}H\}$ NMR spectrum of **19**.

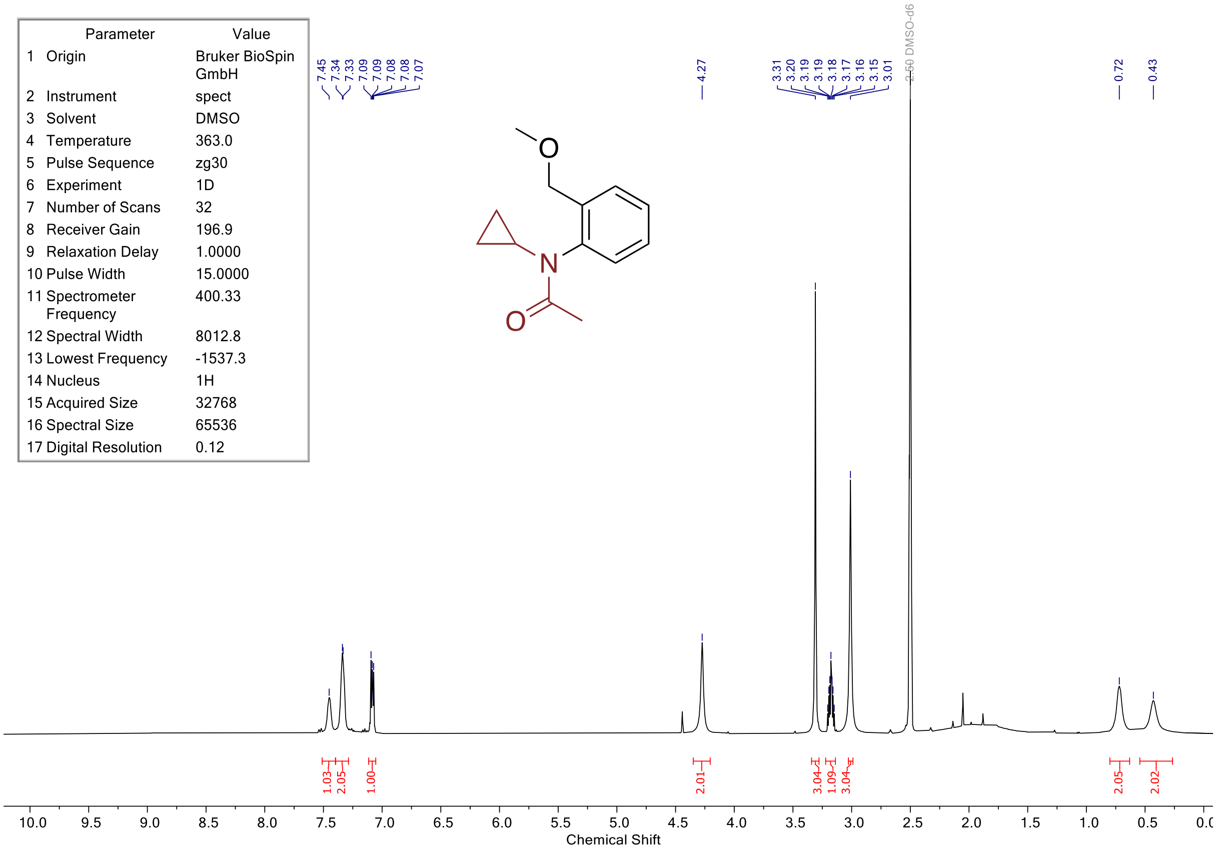

	Parameter	Value
1	Origin	Bruker BioSpin GmbH
2	Instrument	spect
3	Solvent	DMSO
4	Temperature	363.0
5	Pulse Sequence	zg30
6	Experiment	1D
7	Number of Scans	32
8	Receiver Gain	196.9
9	Relaxation Delay	1.0000
10	Pulse Width	15.0000
11	Spectrometer Frequency	400.33
12	Spectral Width	8012.8
13	Lowest Frequency	-1537.3
14	Nucleus	1H
15	Acquired Size	32768
16	Spectral Size	65536
17	Digital Resolution	0.12

Figure S59. ^{1}H NMR spectrum of **20**.

Measurement of the $^{13}C\{^{1}H\}$ NMR spectrum of **20** was not possible.

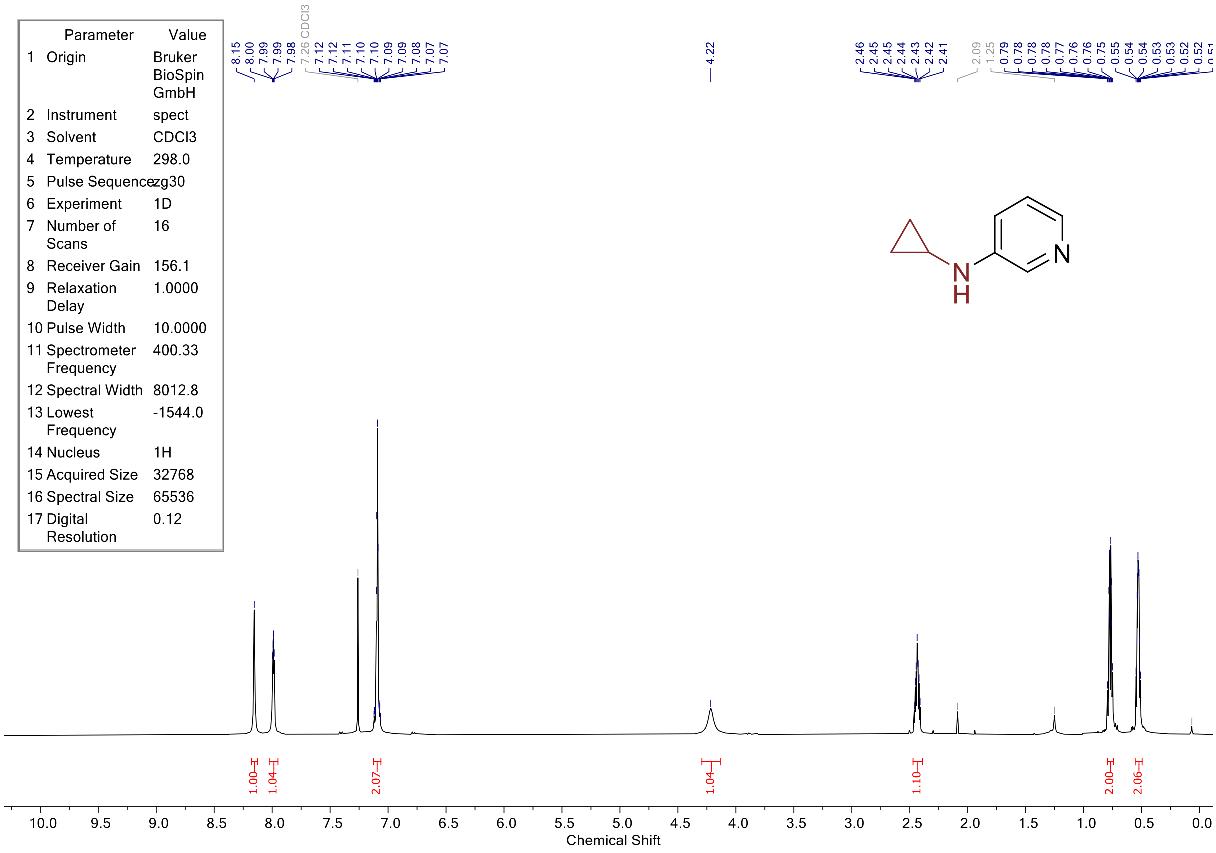

	Parameter	Value
1	Origin	Bruker BioSpin GmbH
2	Instrument	spect
3	Solvent	CDCl3
4	Temperature	298.0
5	Pulse Sequence	zg30
6	Experiment	1D
7	Number of Scans	16
8	Receiver Gain	156.1
9	Relaxation Delay	1.0000
10	Pulse Width	10.0000
11	Spectrometer Frequency	400.33
12	Spectral Width	8012.8
13	Lowest Frequency	-1544.0
14	Nucleus	1H
15	Acquired Size	32768
16	Spectral Size	65536
17	Digital Resolution	0.12

Figure S60. ^{1}H NMR spectrum of **21**.

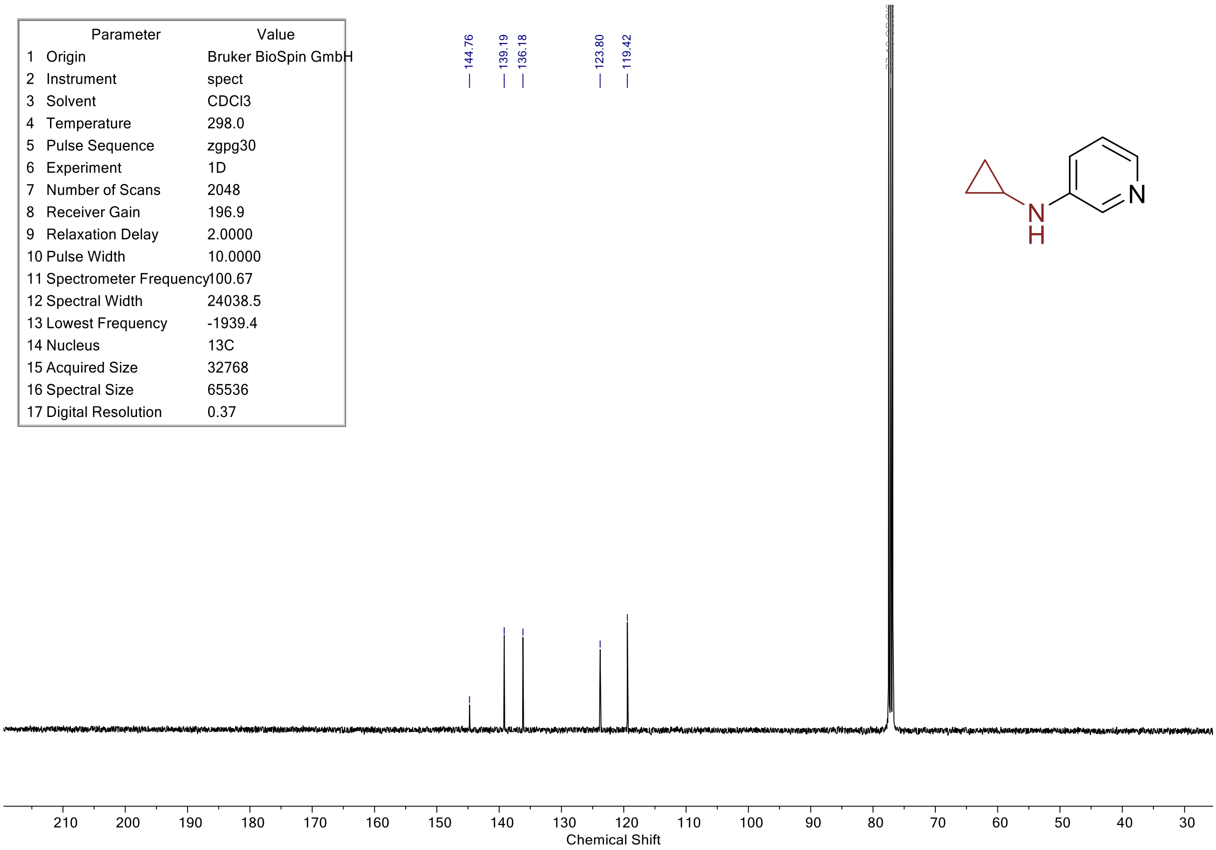

Figure S61. $^{13}C\{^{1}H\}$ NMR spectrum of **21**.

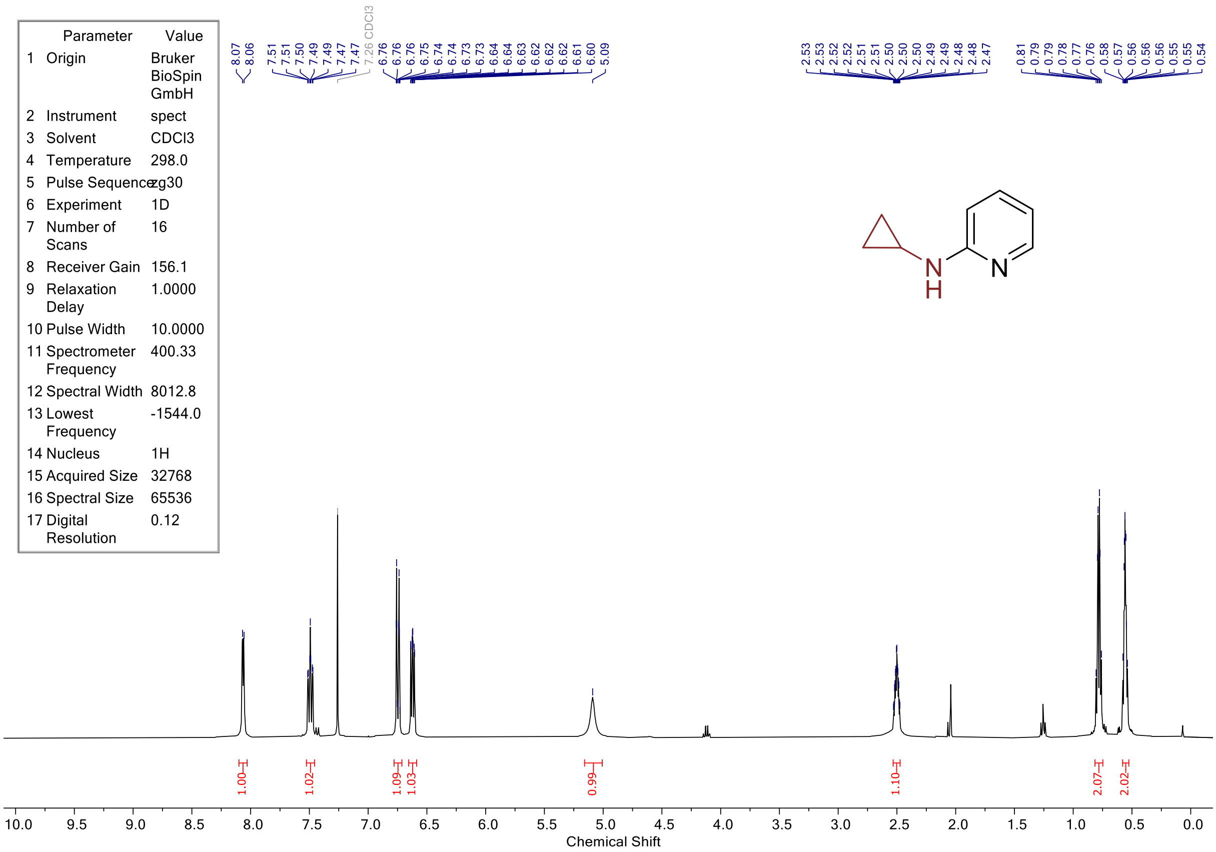

Figure S62. ^{1}H NMR spectrum of **22**.

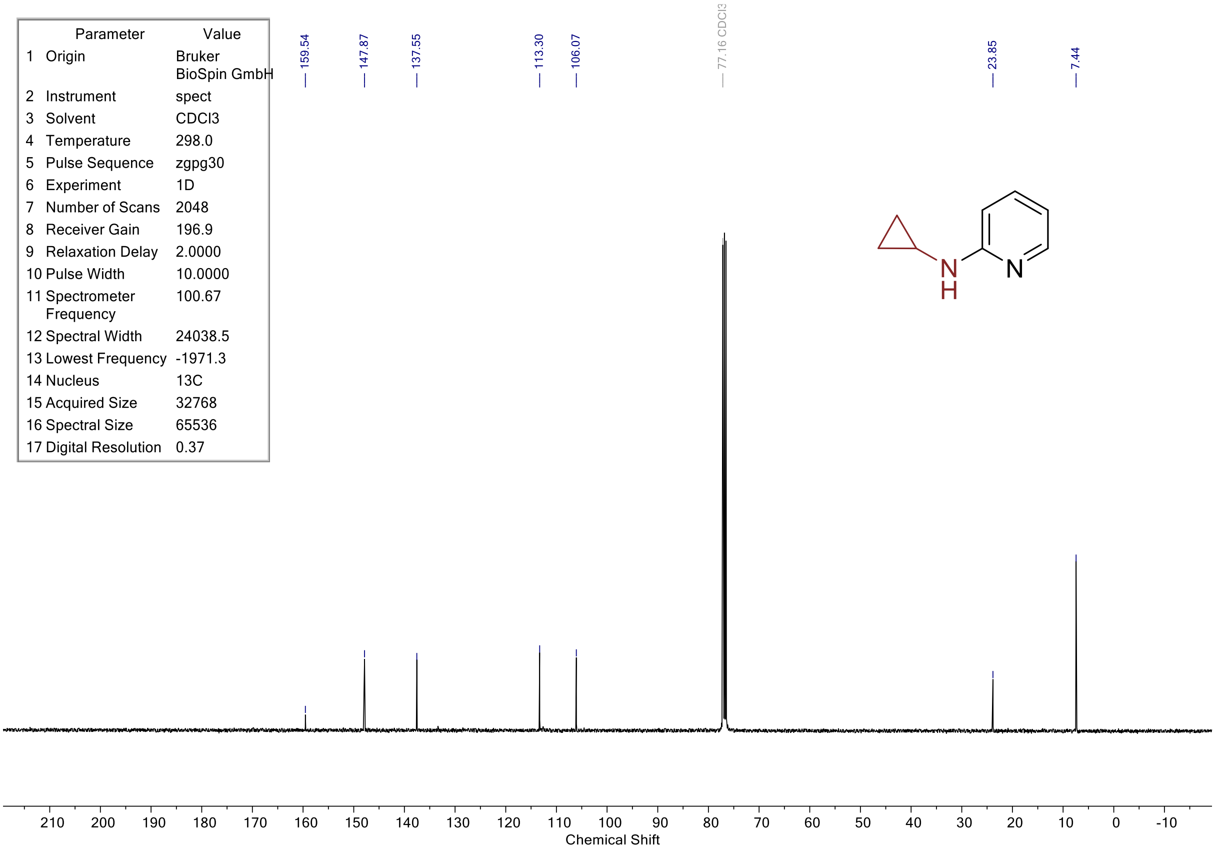

Figure S63. $^{13}C\{^{1}H\}$ NMR spectrum of **22**.

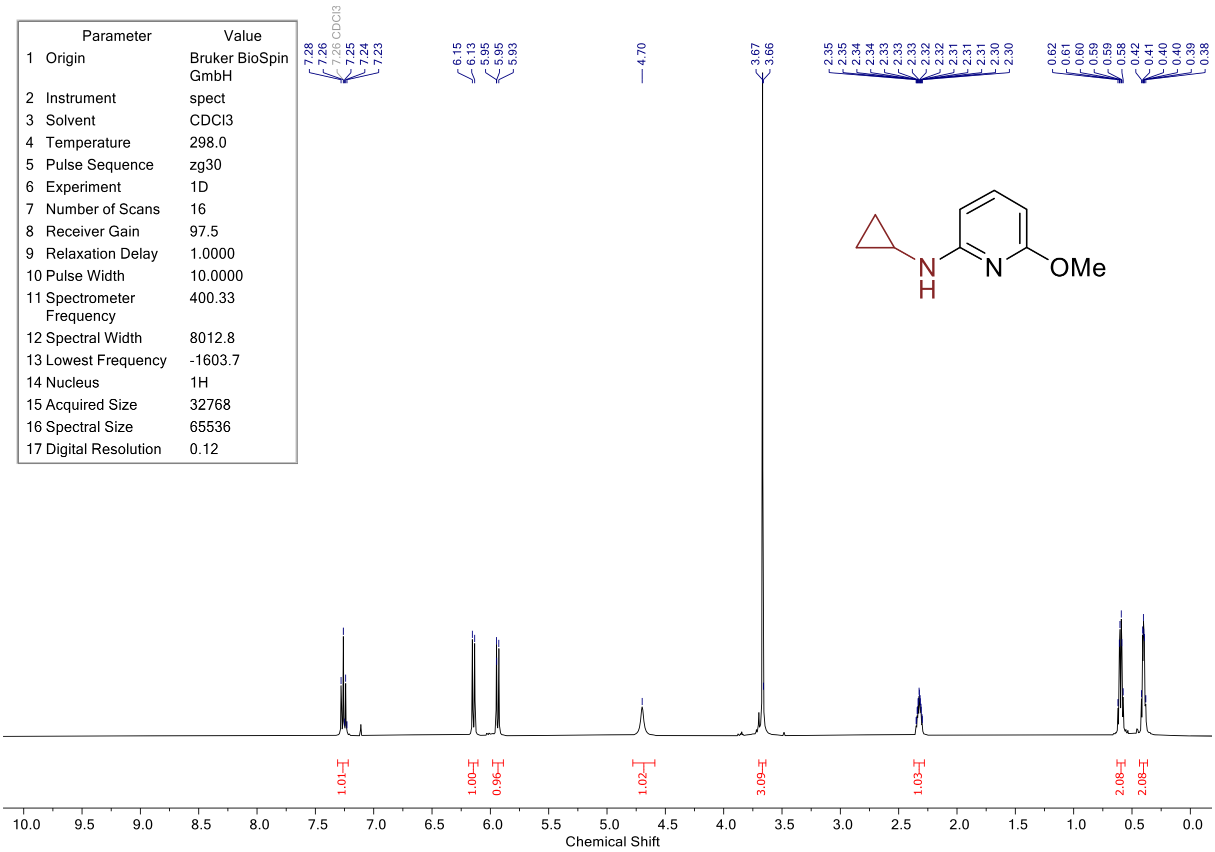

Figure S64. ^{1}H NMR spectrum of **23**.

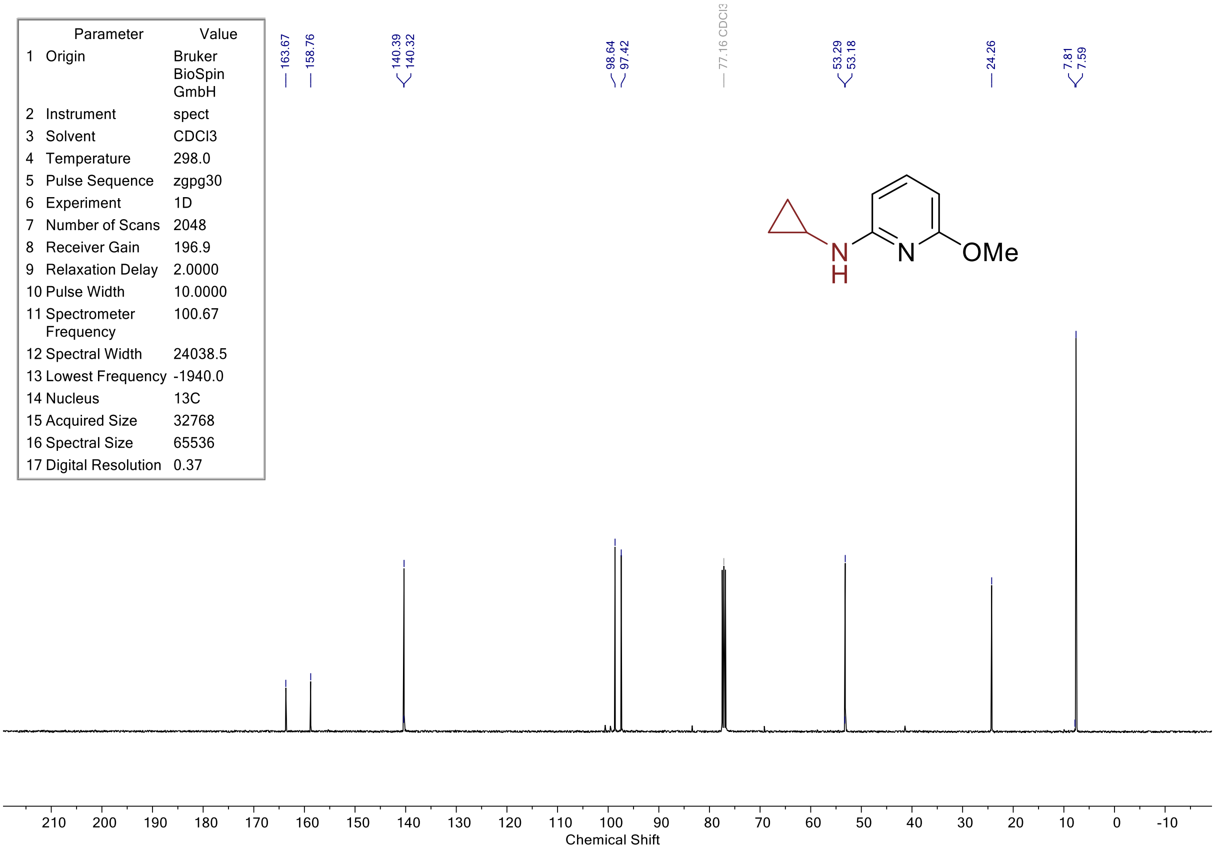

Figure S65. $^{13}C\{^{1}H\}$ NMR spectrum of **23**.

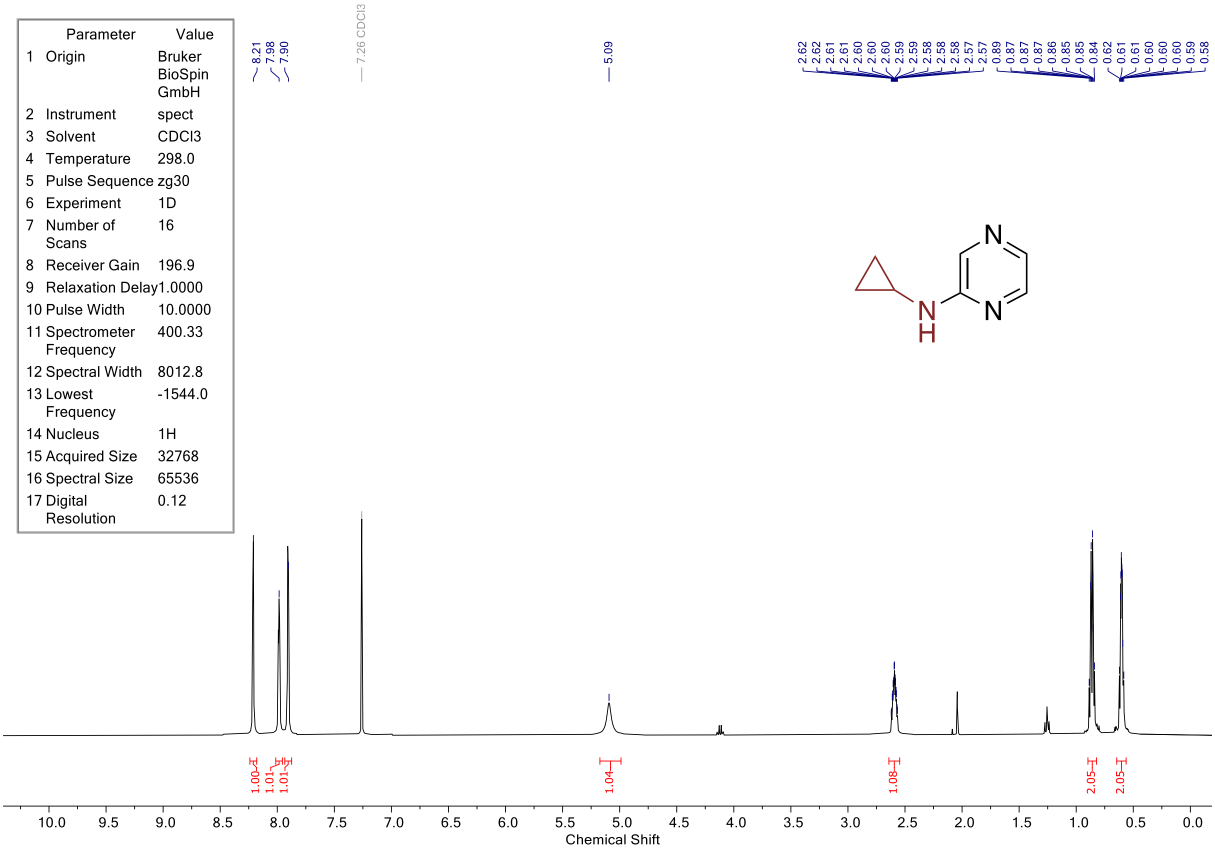

Figure S66. ^{1}H NMR spectrum of **24**.

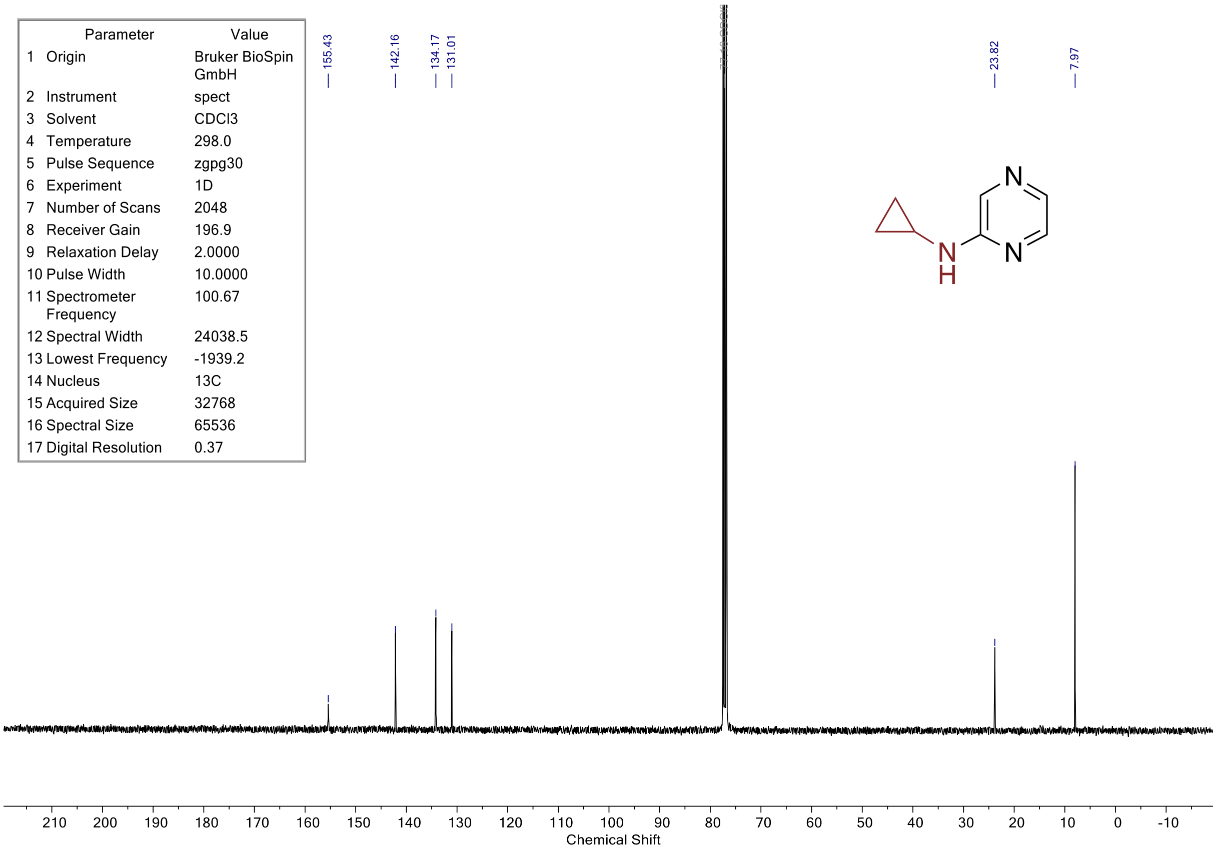

Figure S67. $^{13}C\{^{1}H\}$ NMR spectrum of **24**.

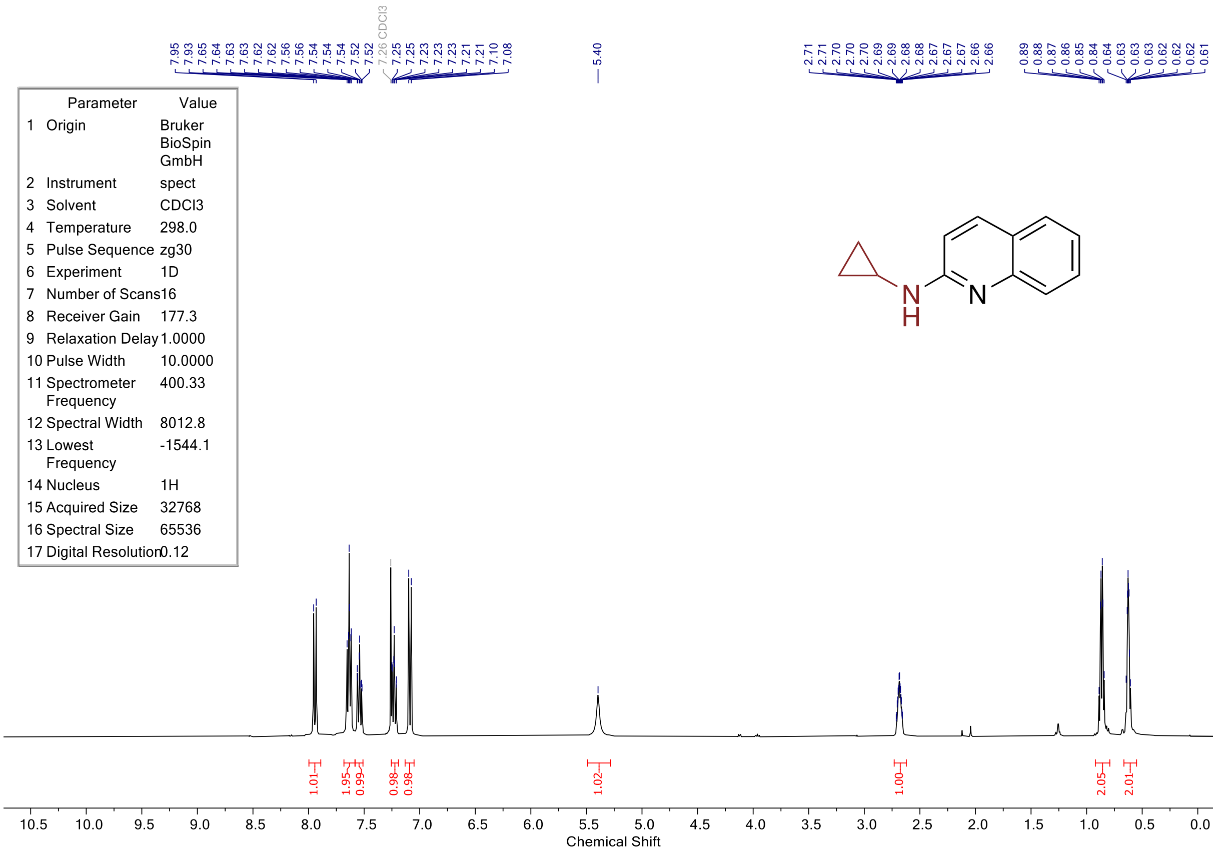

Figure S68. ^{1}H NMR spectrum of **25**.

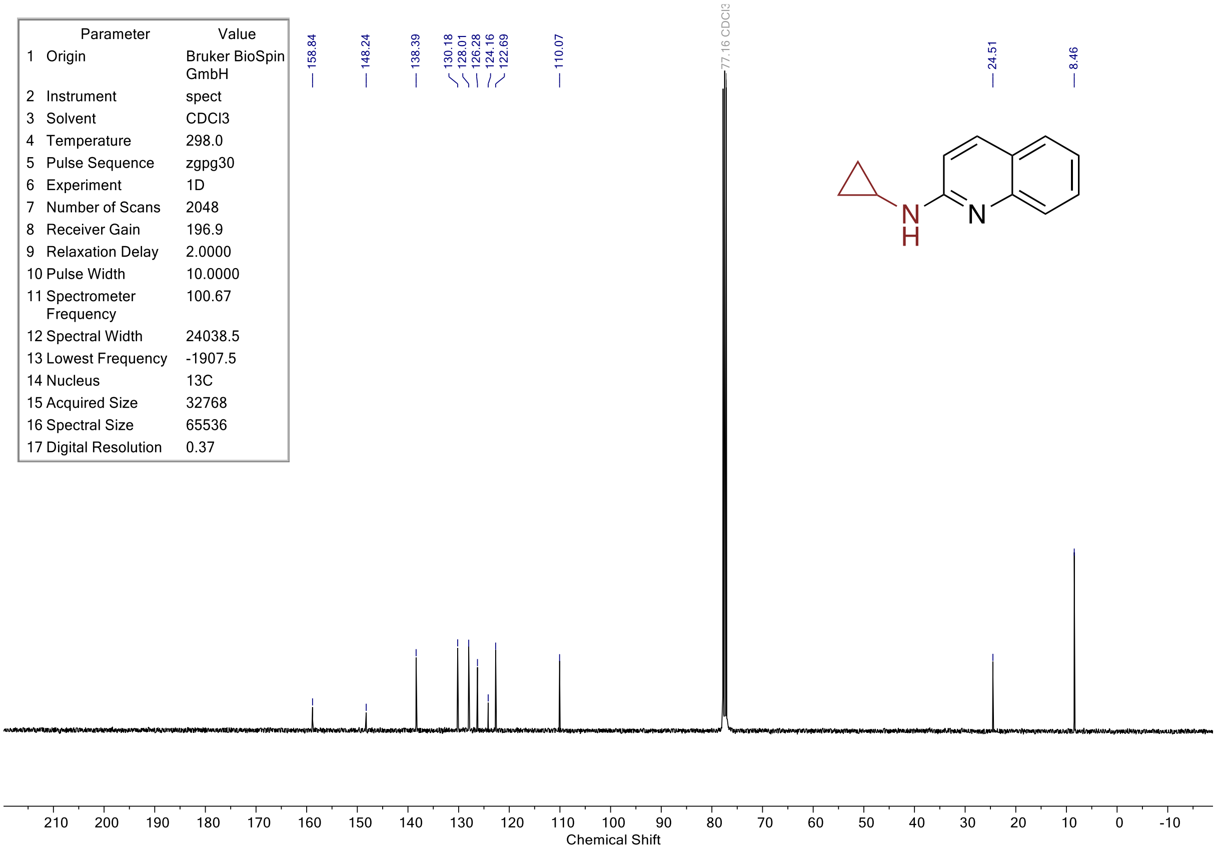

Figure S69. $^{13}C\{^{1}H\}$ NMR spectrum of **25**.

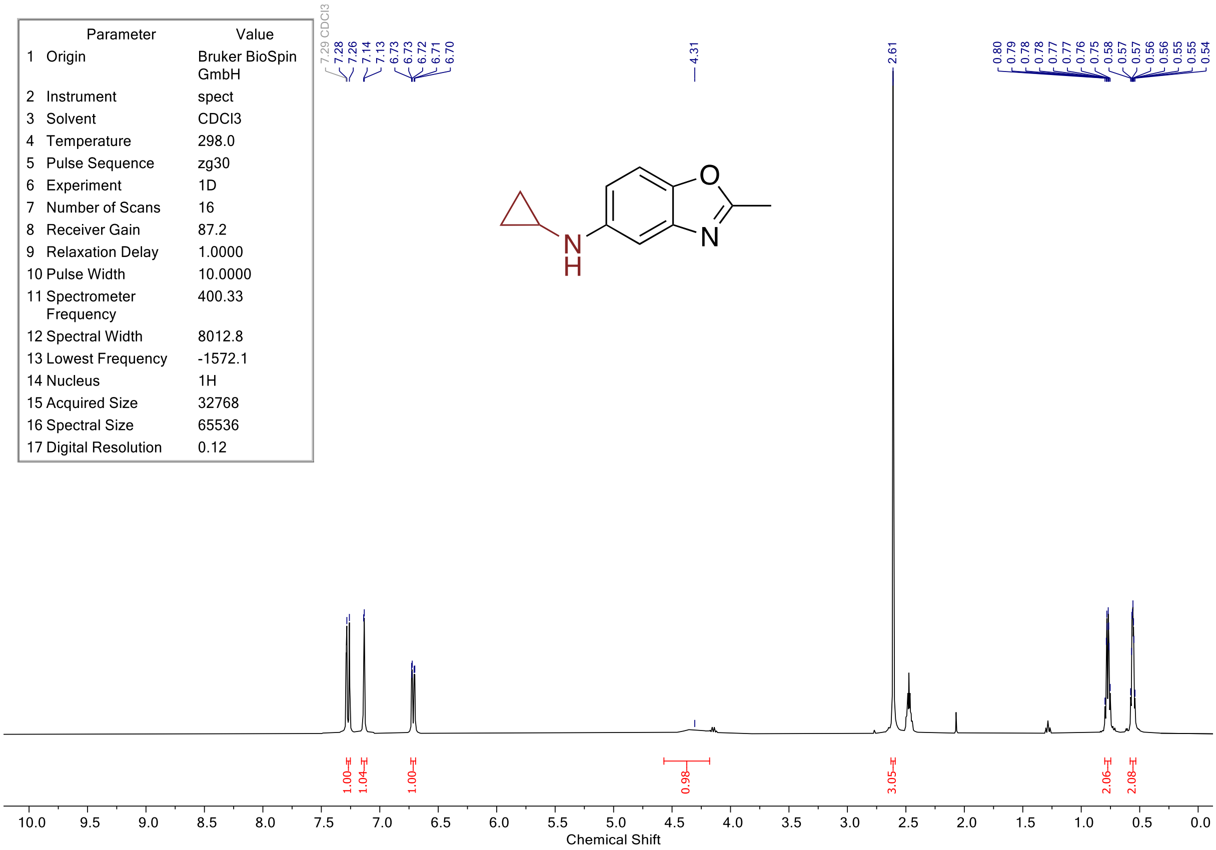

Figure S70. ^{1}H NMR spectrum of **26**.

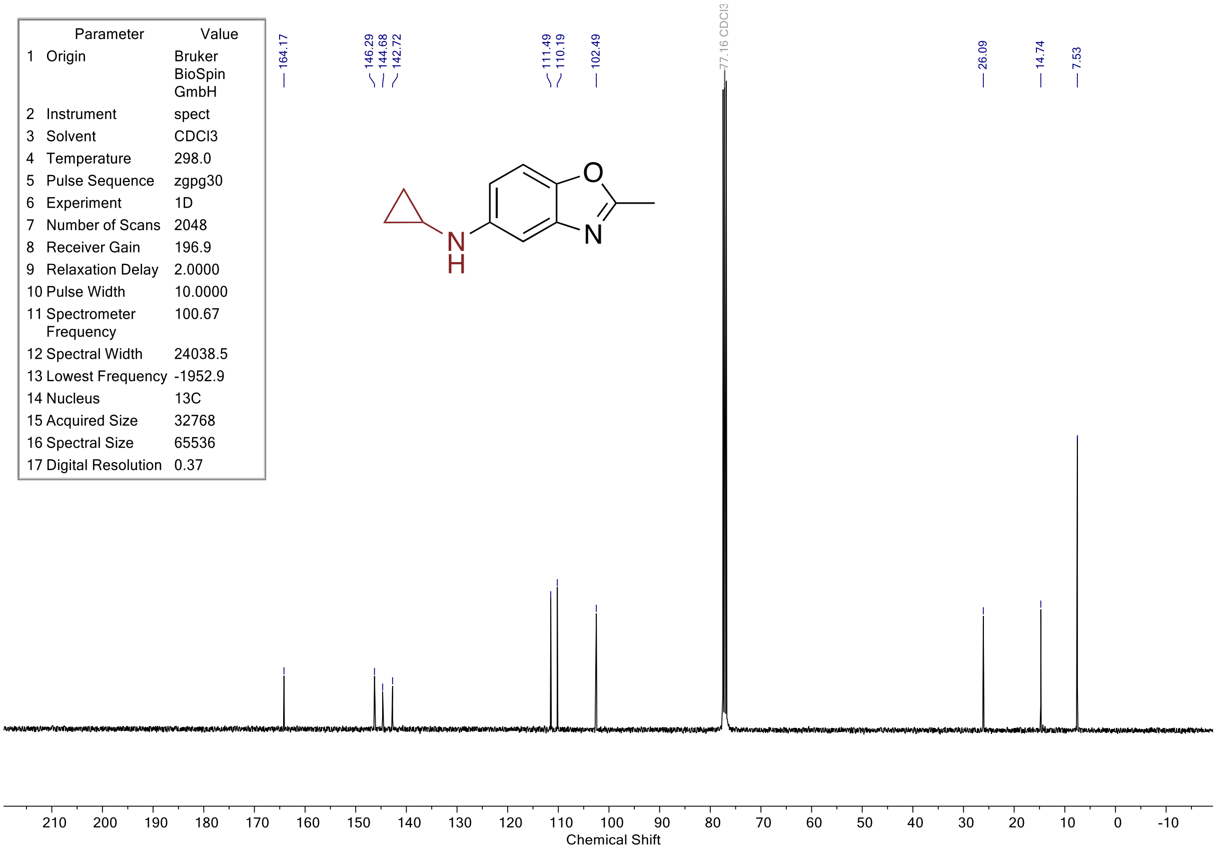

Figure S71. $^{13}C\{^{1}H\}$ NMR spectrum of **26**.

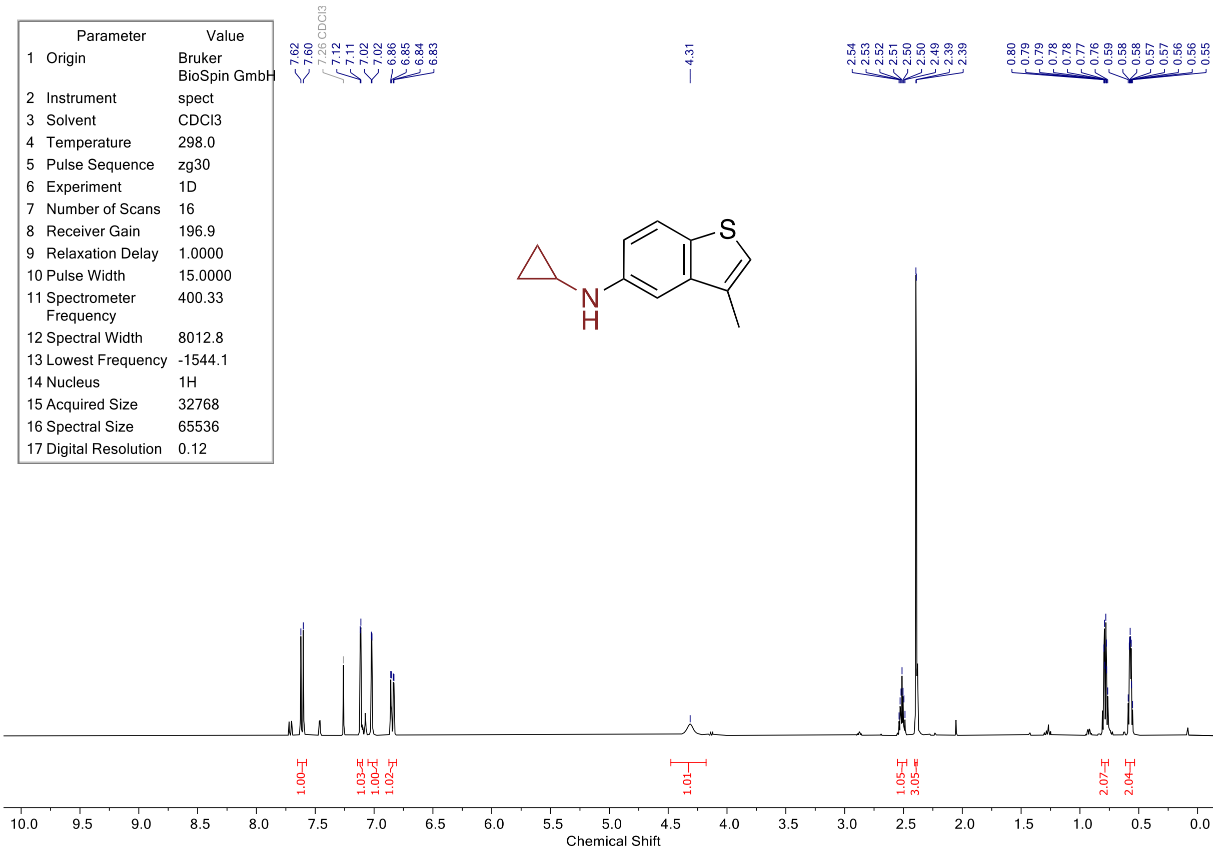

Figure S72. ^{1}H NMR spectrum of **27**.

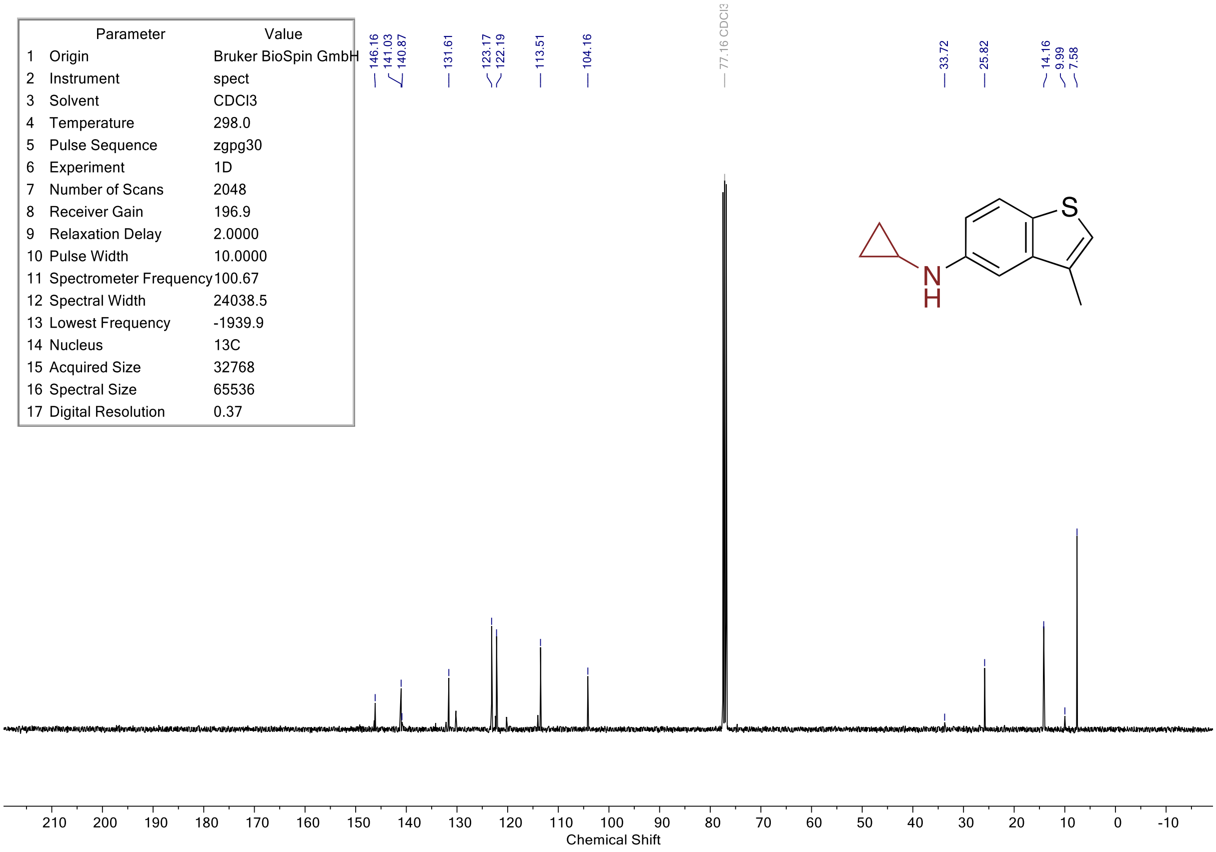

Figure S73. $^{13}C\{^1H\}$ NMR spectrum of **27**.

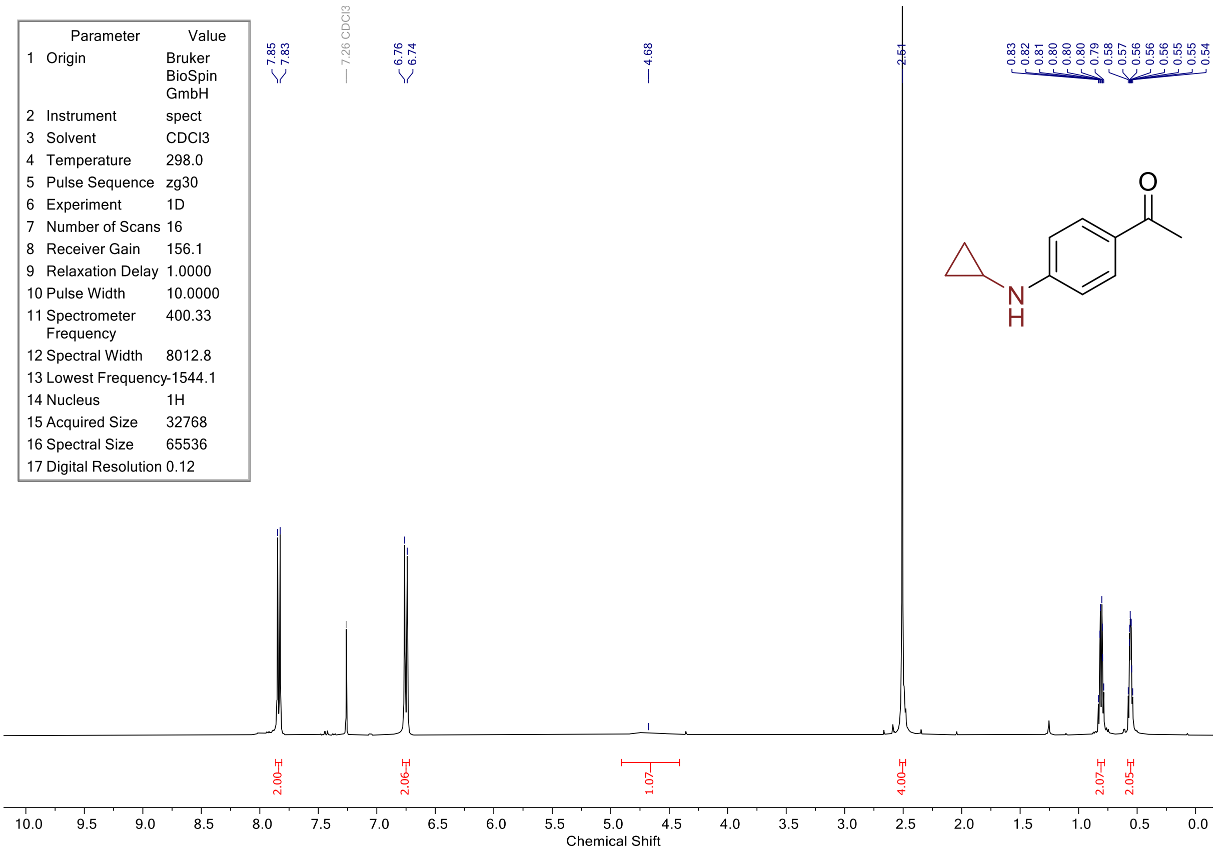

Figure S74. ^1H NMR spectrum of **28**.

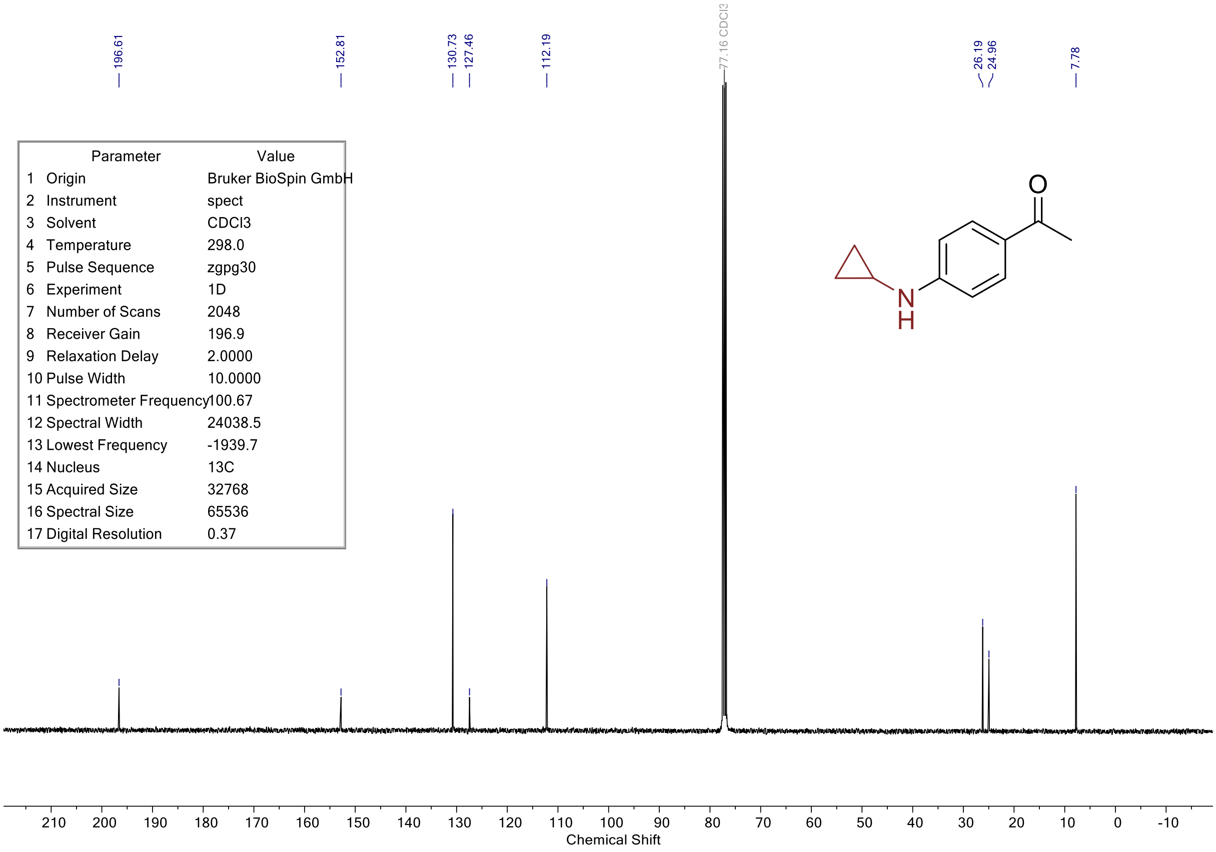

Figure S75. $^{13}C\{^1H\}$ NMR spectrum of **28**.

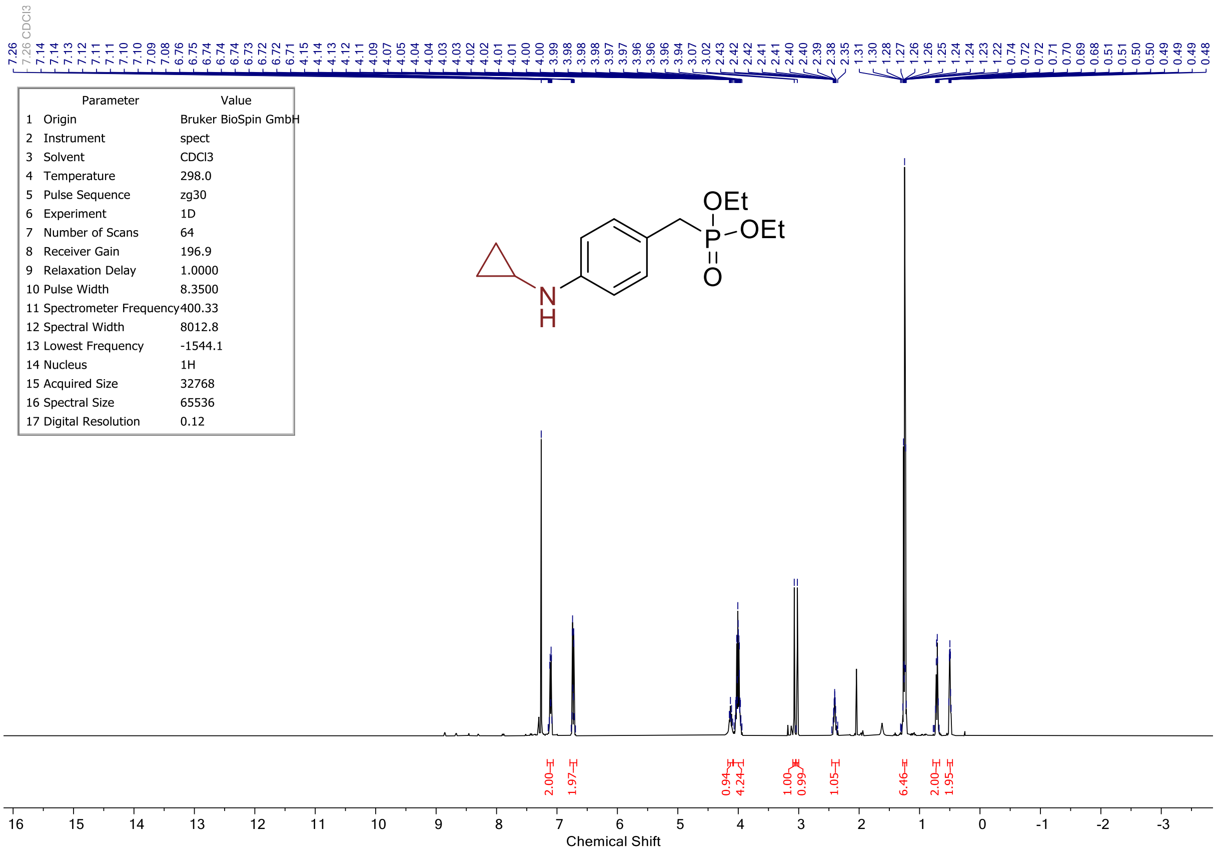

Figure S76. ^1H NMR spectrum of **29**.

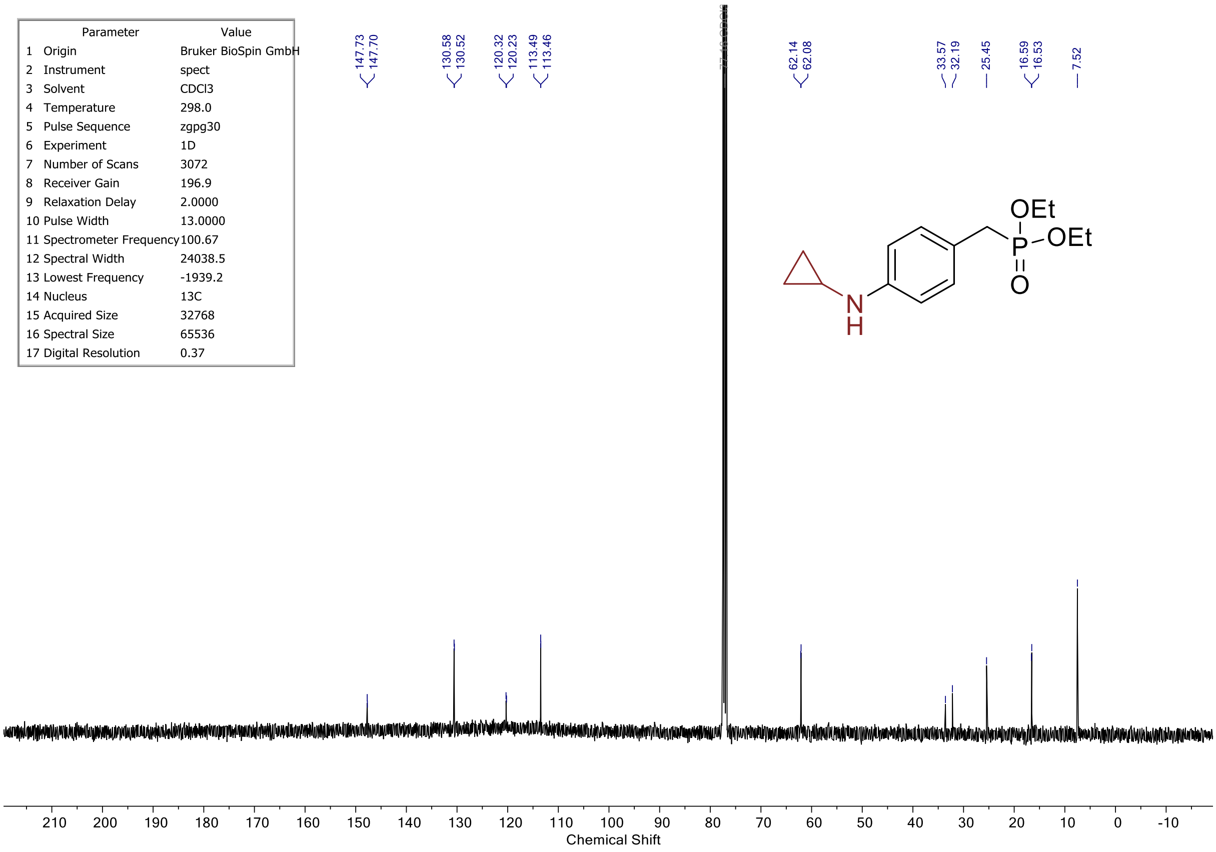

Figure S77. $^{13}C\{^{1}H\}$ NMR spectrum of **29**.

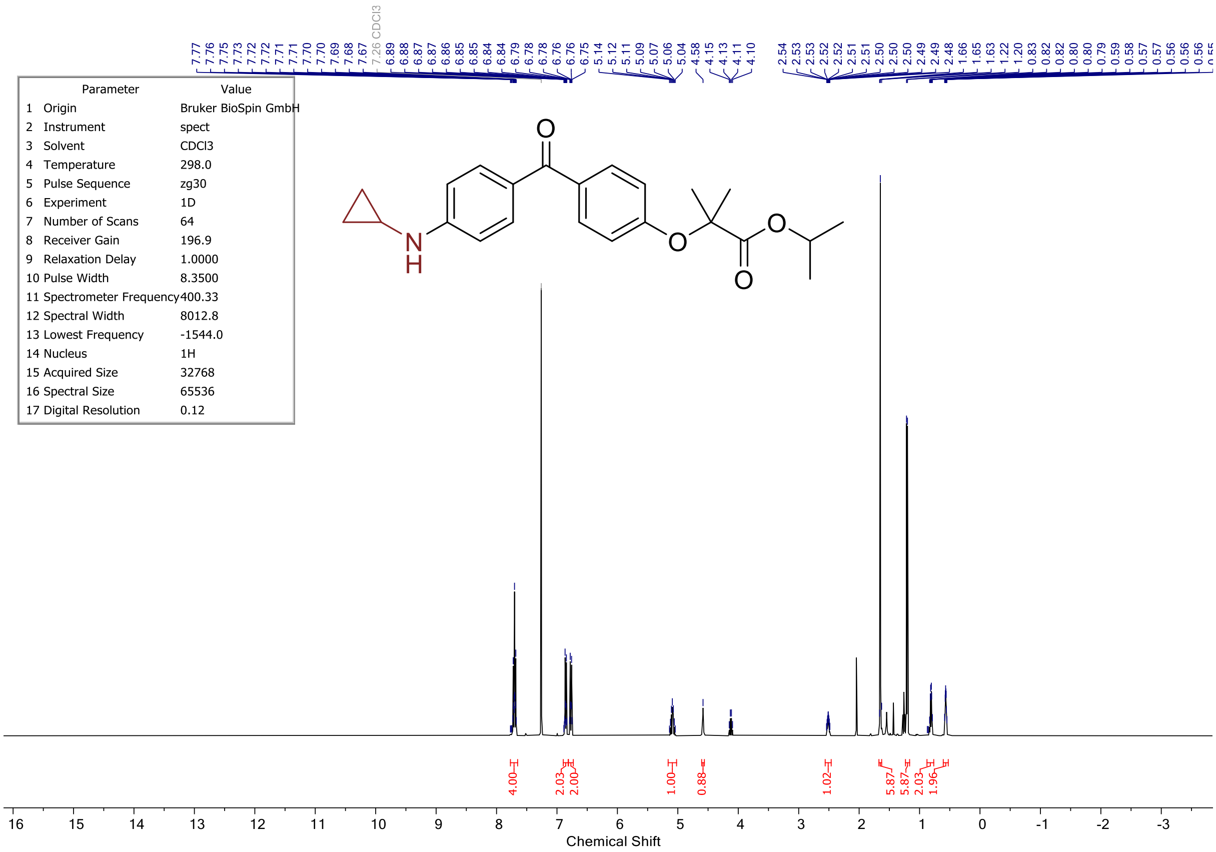

Figure S78. ^{1}H NMR spectrum of **30**.

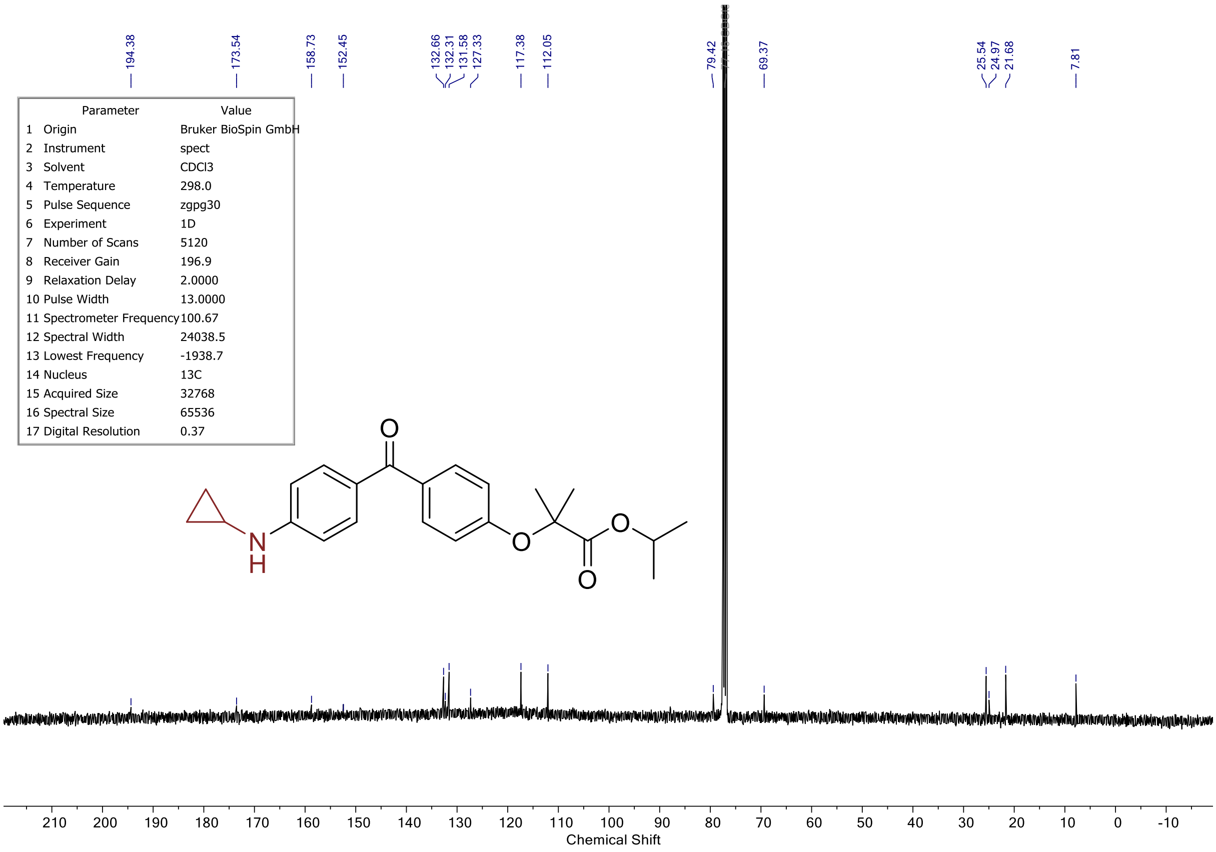

Figure S79. $^{13}C\{^{1}H\}$ NMR spectrum of **30**.

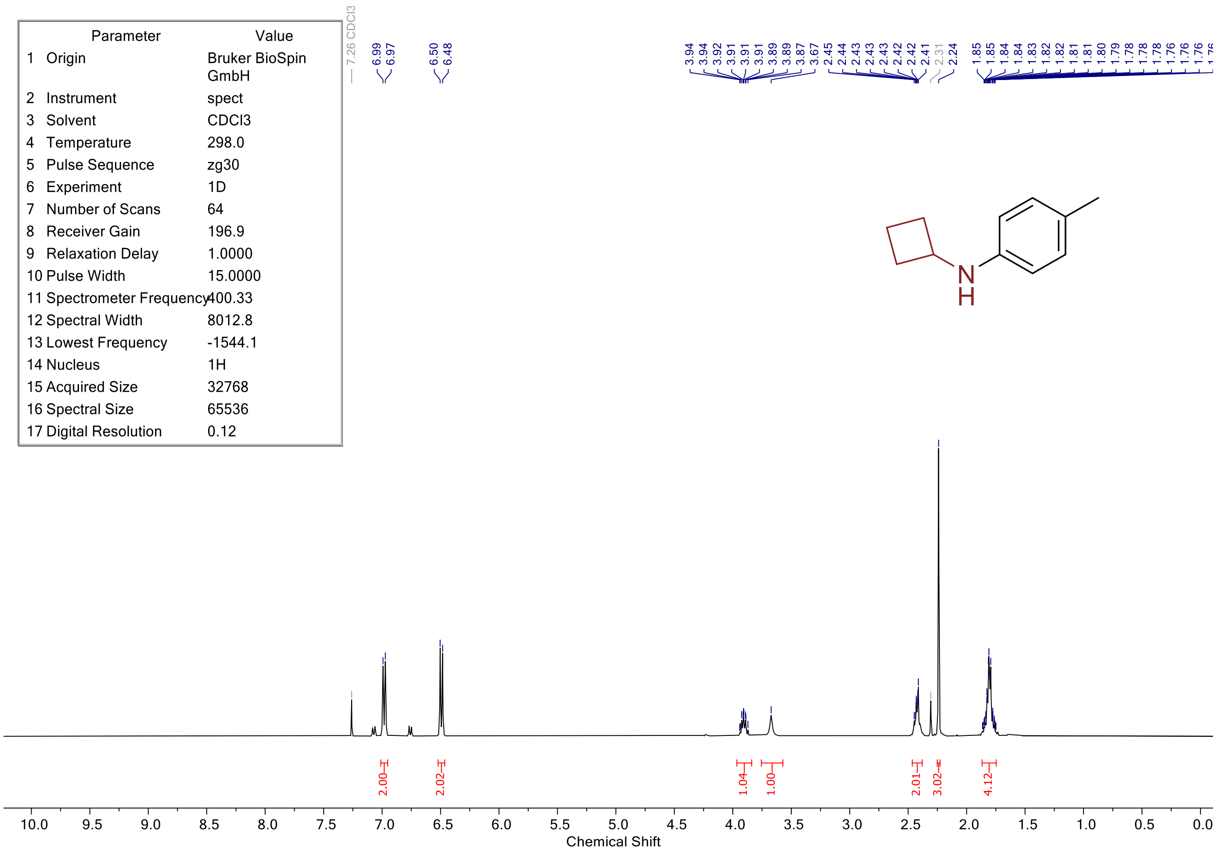

Figure S80. ^{1}H NMR spectrum of **31**.

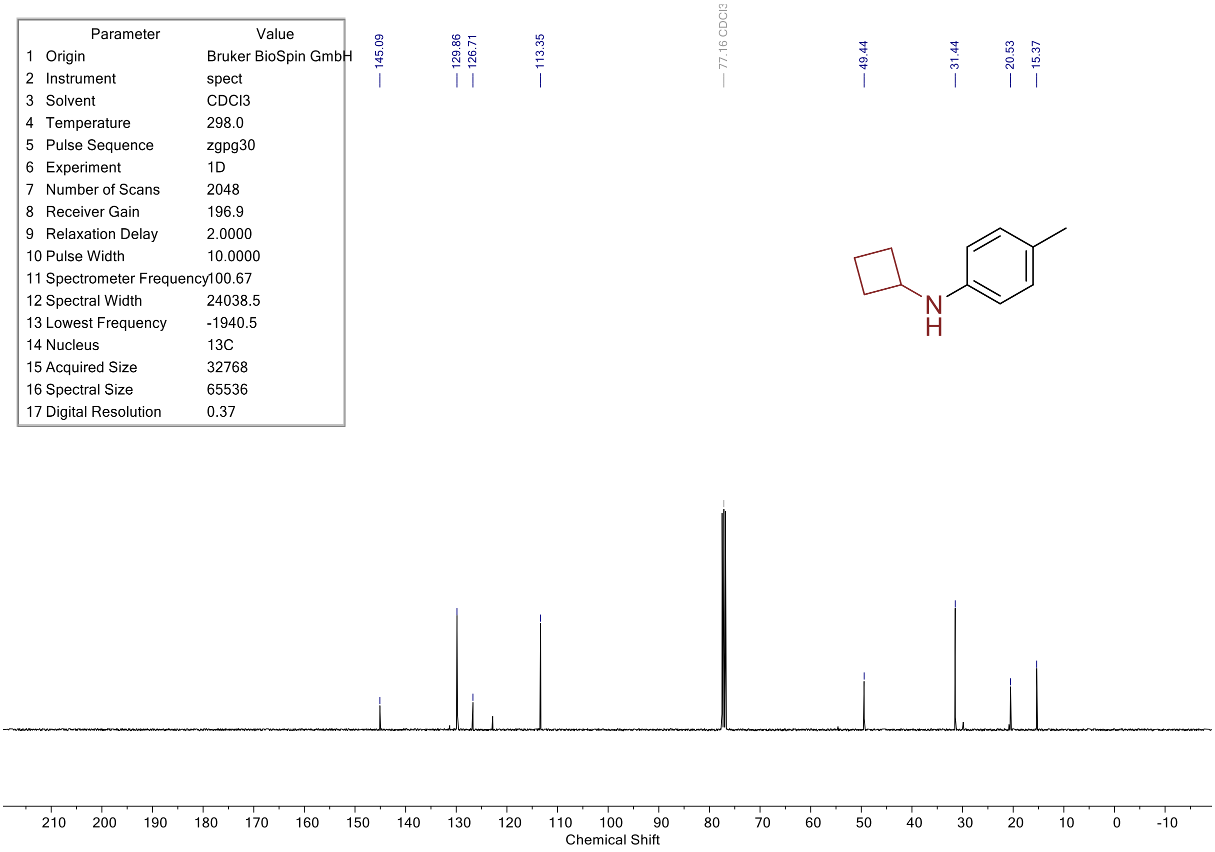

Figure S81. $^{13}C\{^{1}H\}$ NMR spectrum of **31**.

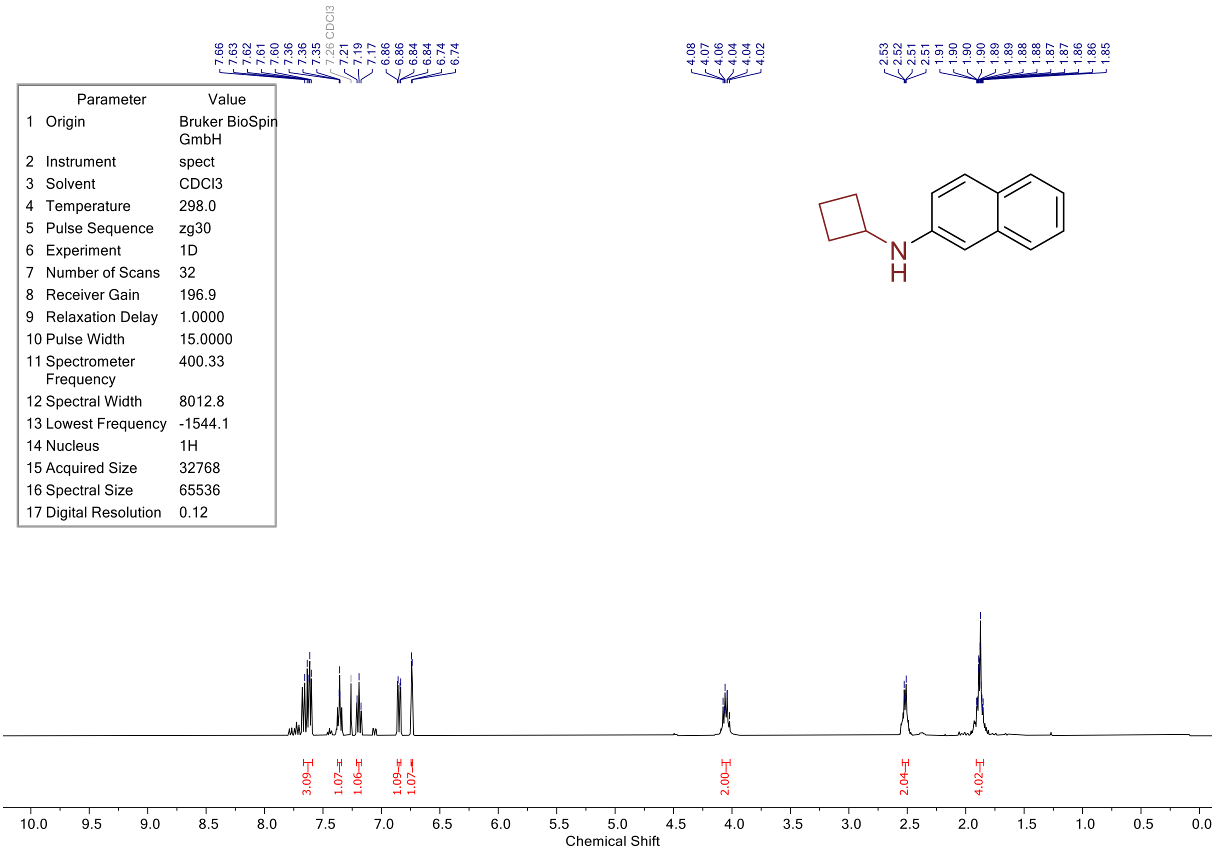

Figure S82. ^{1}H NMR spectrum of **32**.

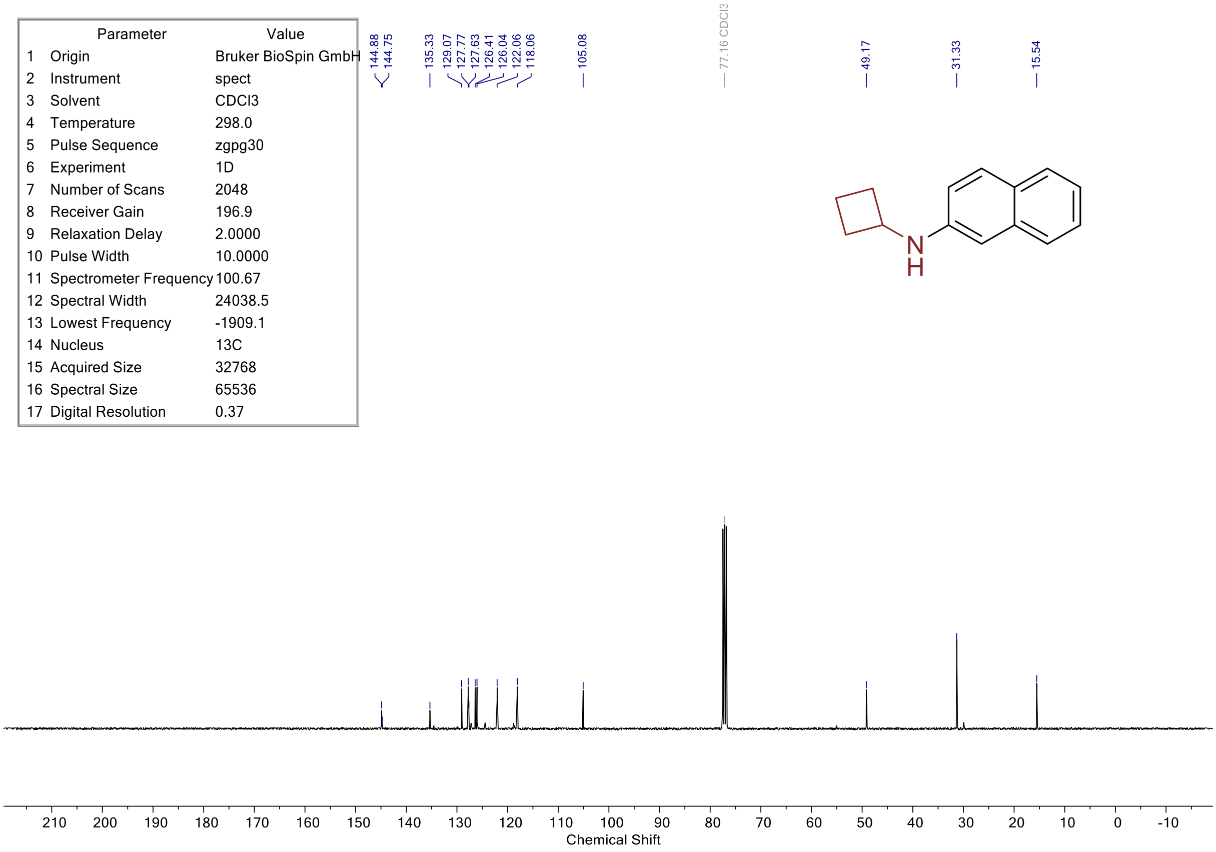

	Parameter	Value
1	Origin	Bruker BioSpin GmbH
2	Instrument	spect
3	Solvent	CDCl3
4	Temperature	298.0
5	Pulse Sequence	zgpg30
6	Experiment	1D
7	Number of Scans	2048
8	Receiver Gain	196.9
9	Relaxation Delay	2.0000
10	Pulse Width	10.0000
11	Spectrometer Frequency	100.67
12	Spectral Width	24038.5
13	Lowest Frequency	-1909.1
14	Nucleus	13C
15	Acquired Size	32768
16	Spectral Size	65536
17	Digital Resolution	0.37

Figure S83. $^{13}C\{^{1}H\}$ NMR spectrum of **32**.

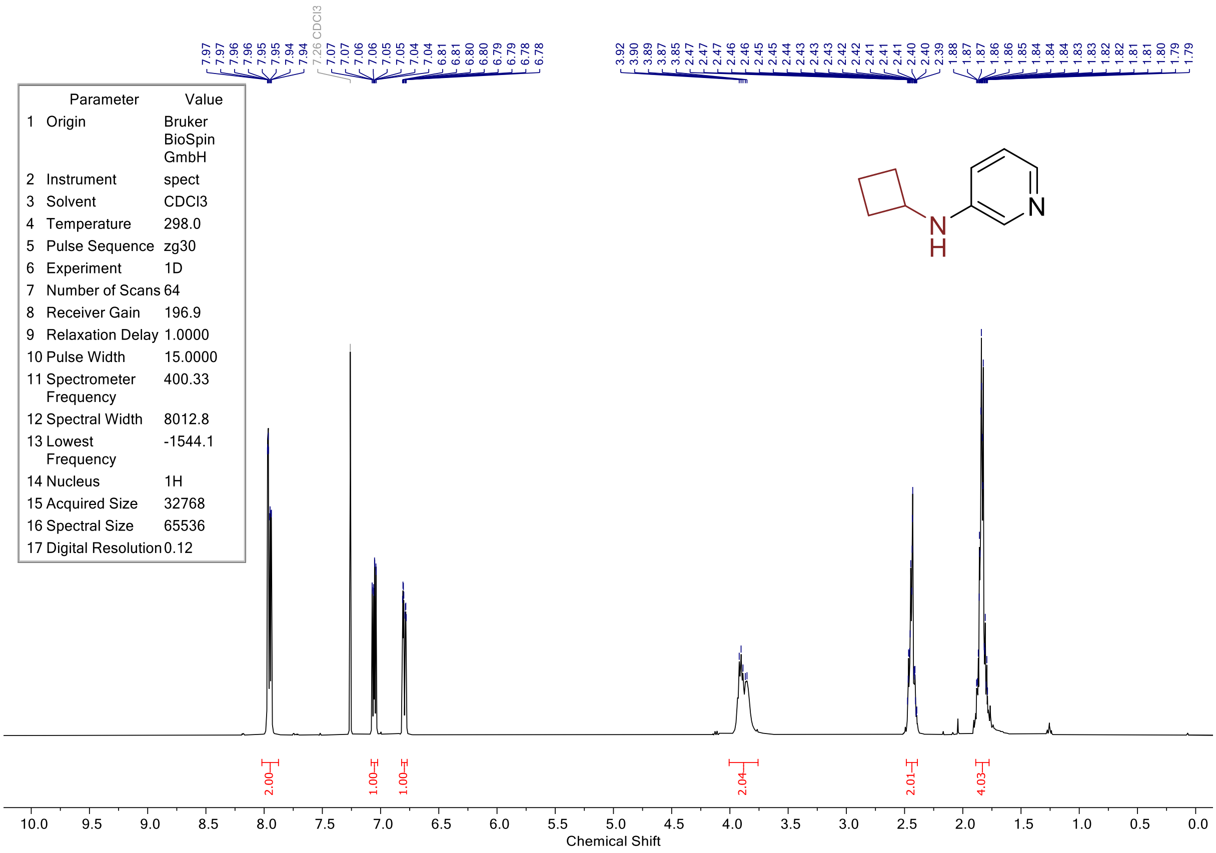

	Parameter	Value
1	Origin	Bruker BioSpin GmbH
2	Instrument	spect
3	Solvent	CDCl3
4	Temperature	298.0
5	Pulse Sequence	zg30
6	Experiment	1D
7	Number of Scans	64
8	Receiver Gain	196.9
9	Relaxation Delay	1.0000
10	Pulse Width	15.0000
11	Spectrometer Frequency	400.33
12	Spectral Width	8012.8
13	Lowest Frequency	-1544.1
14	Nucleus	1H
15	Acquired Size	32768
16	Spectral Size	65536
17	Digital Resolution	0.12

Figure S84. ^{1}H NMR spectrum of **33**.

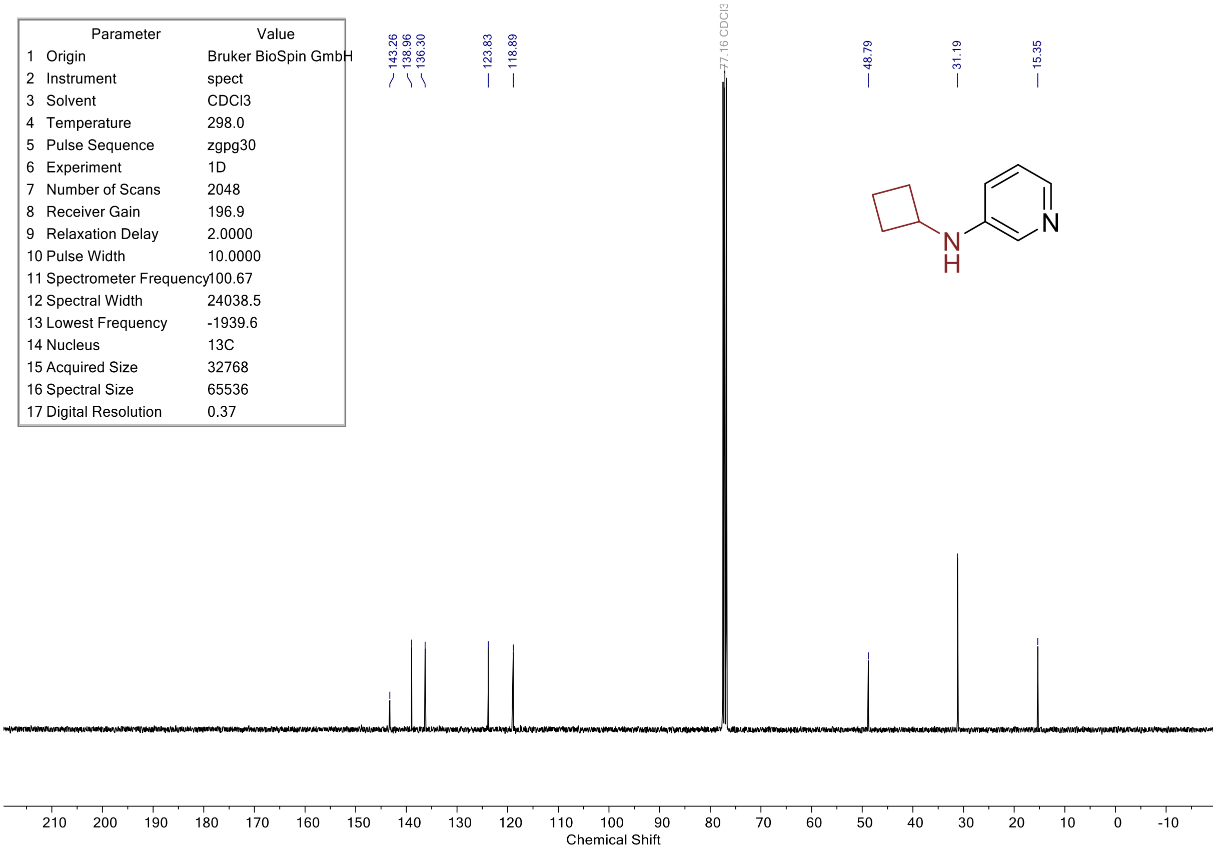

Figure S85. $^{13}C\{^{1}H\}$ NMR spectrum of **33**.

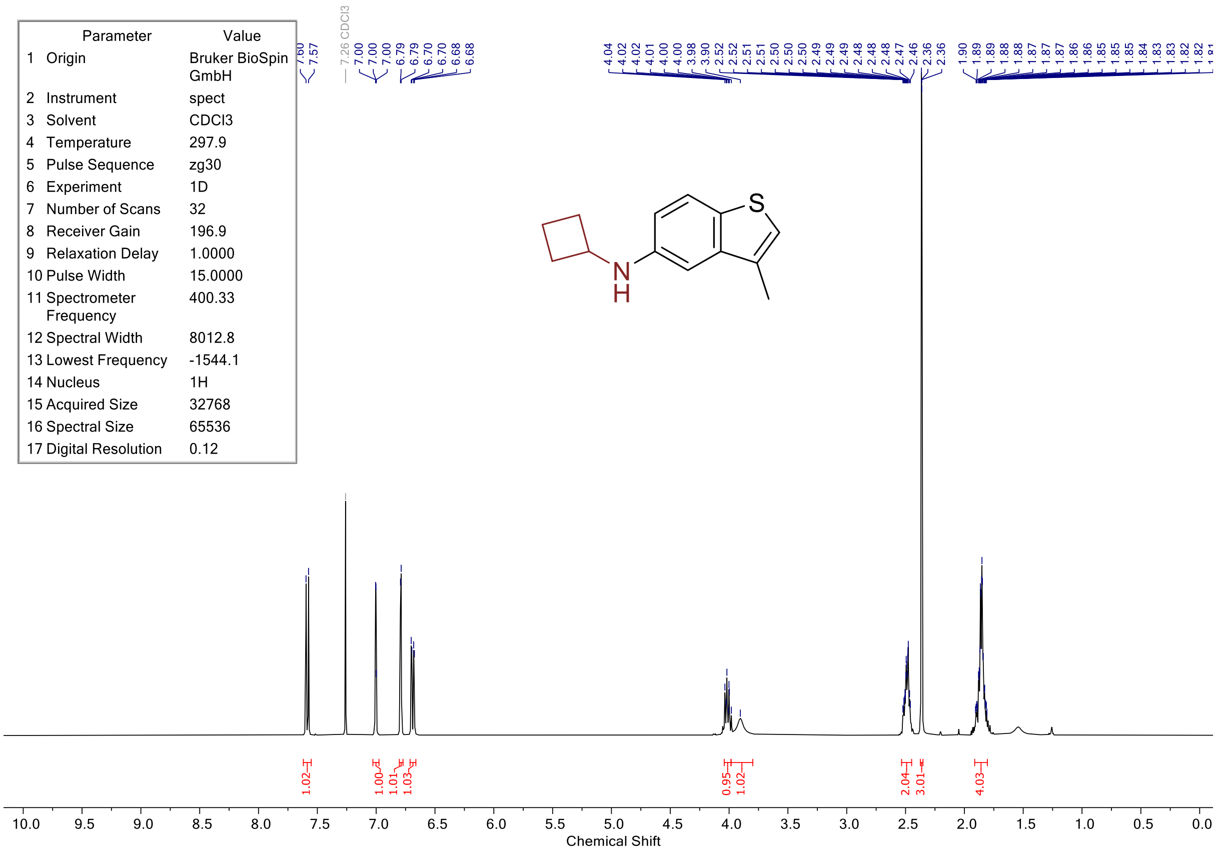

Figure S86. ^{1}H NMR spectrum of **34**.

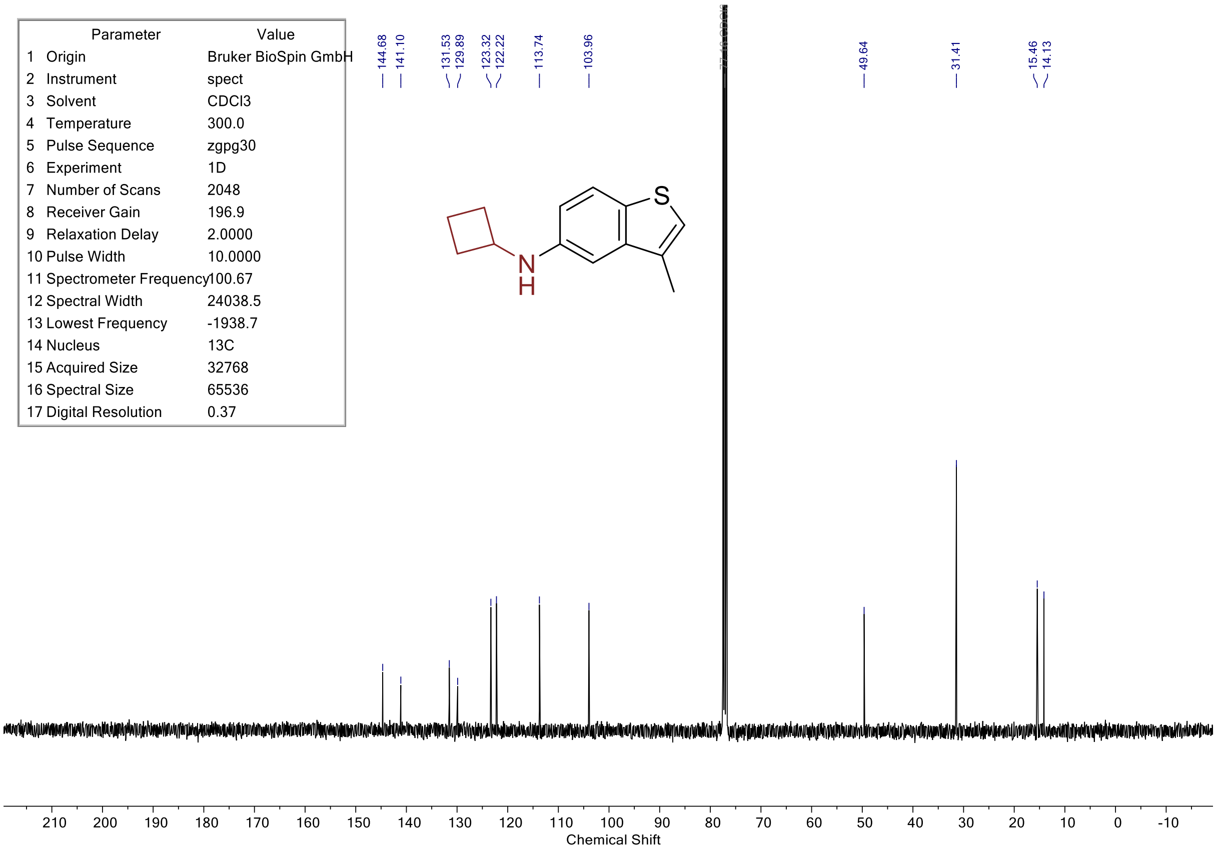

Figure S87. $^{13}C\{^1H\}$ NMR spectrum of **34**.

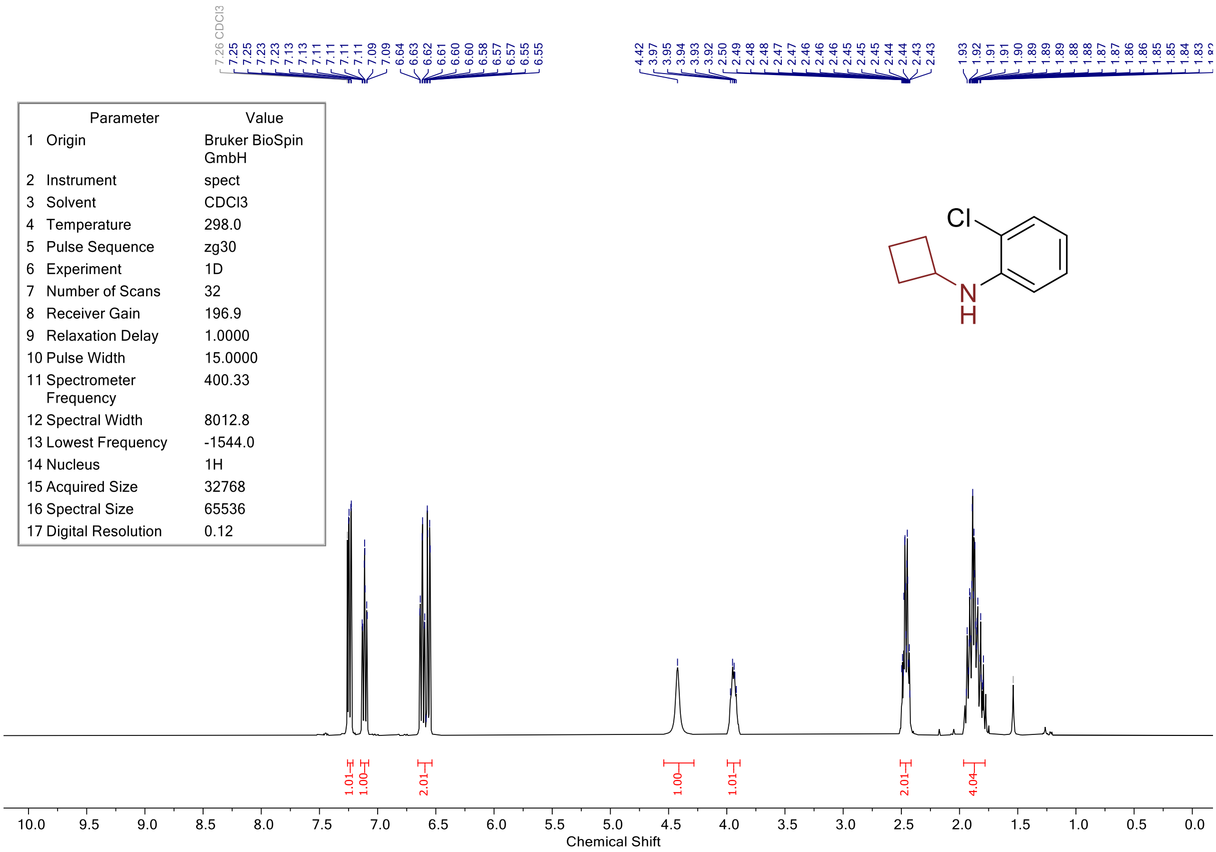

Figure S88. ^1H NMR spectrum of **35**.

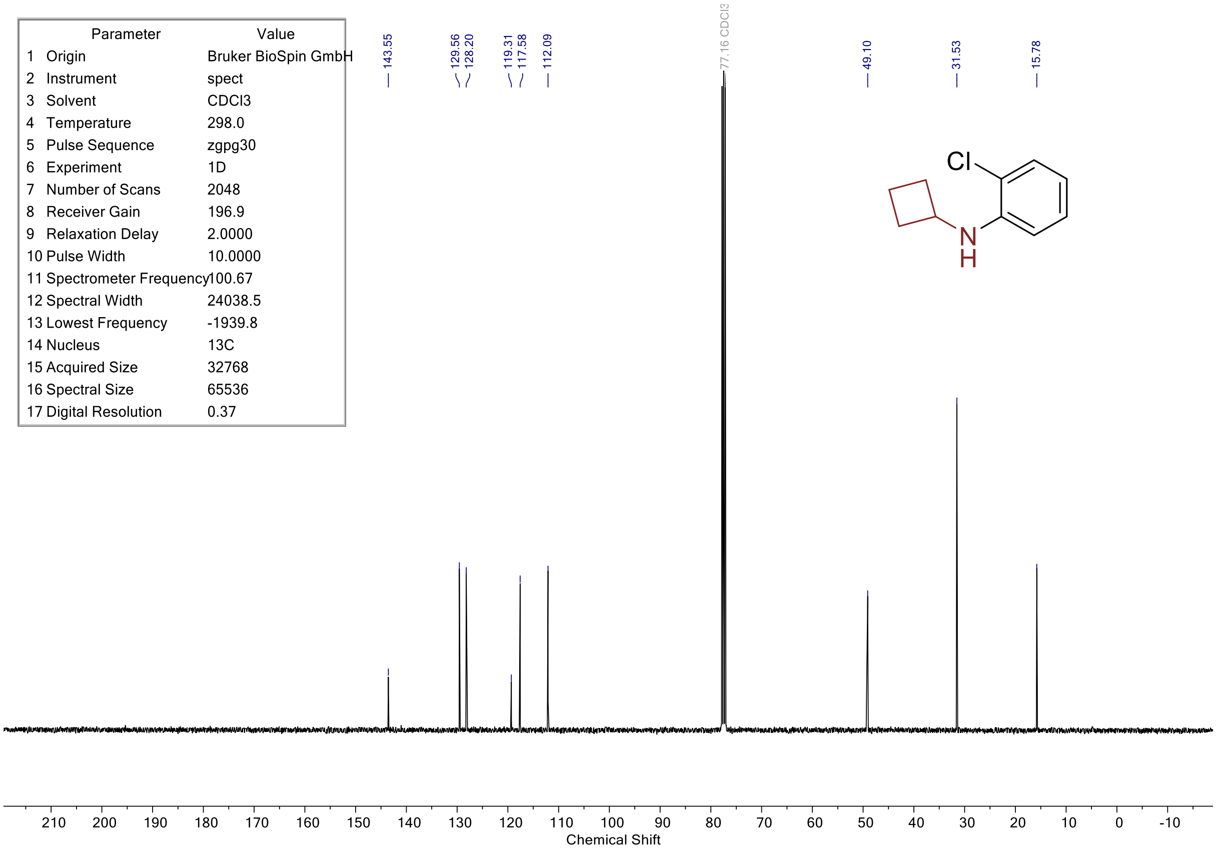

Figure S89. $^{13}C\{^{1}H\}$ NMR spectrum of **35**.

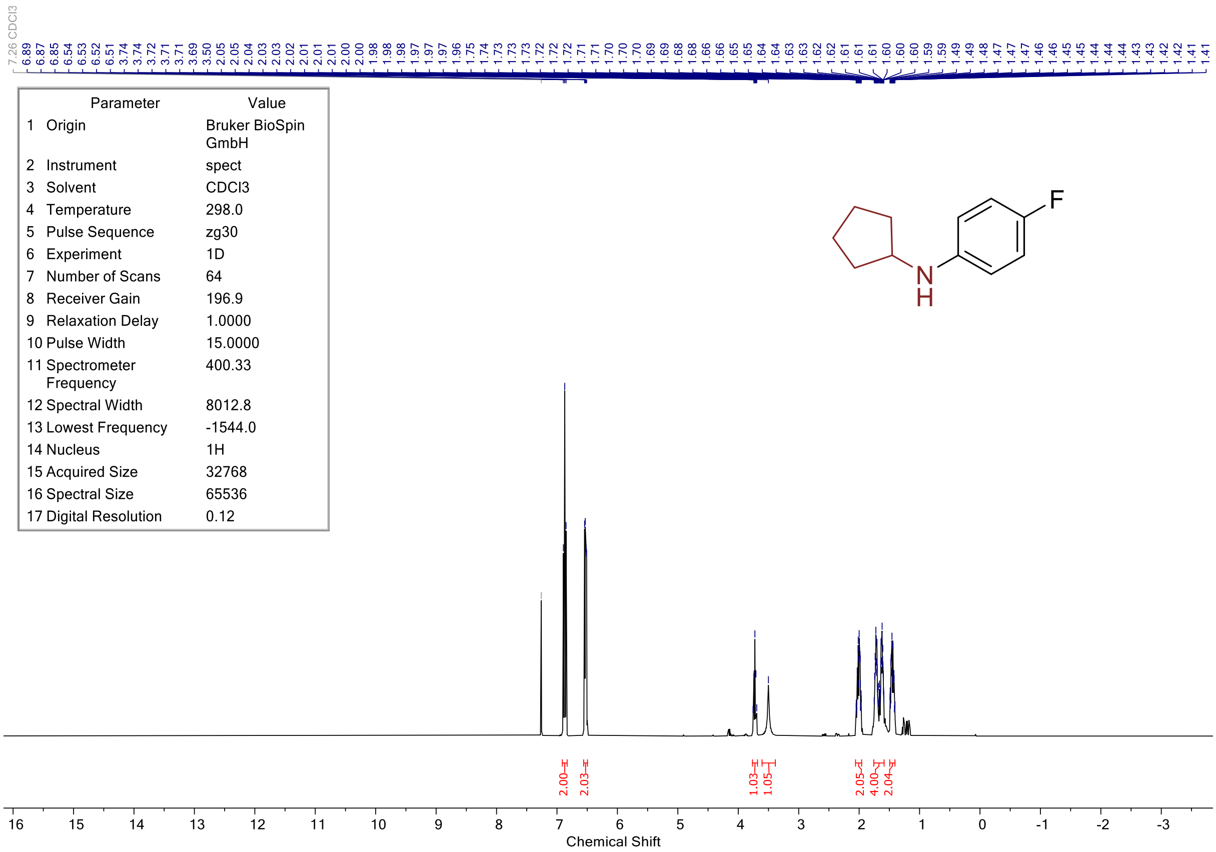

Figure S90. ^{1}H NMR spectrum of **36**.

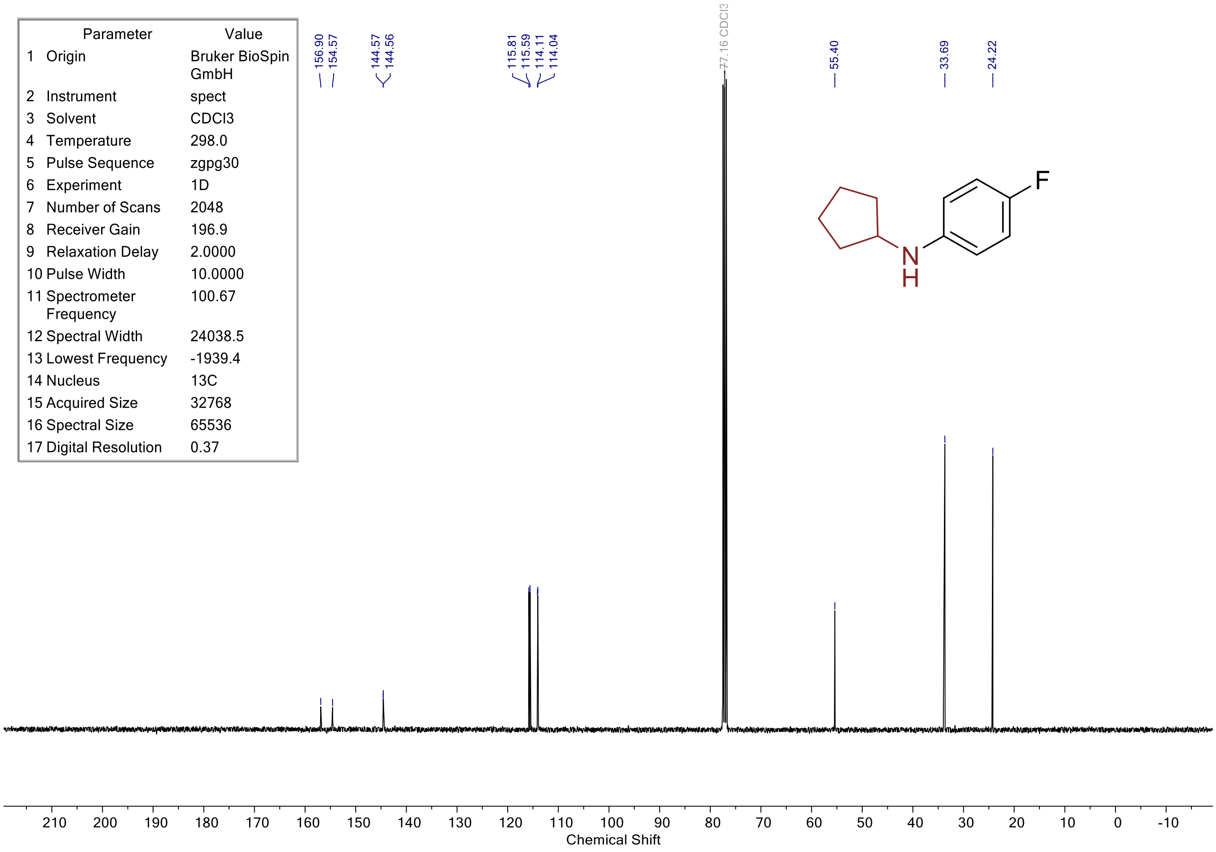

Figure S91. $^{13}C\{^{1}H\}$ NMR spectrum of **36**.

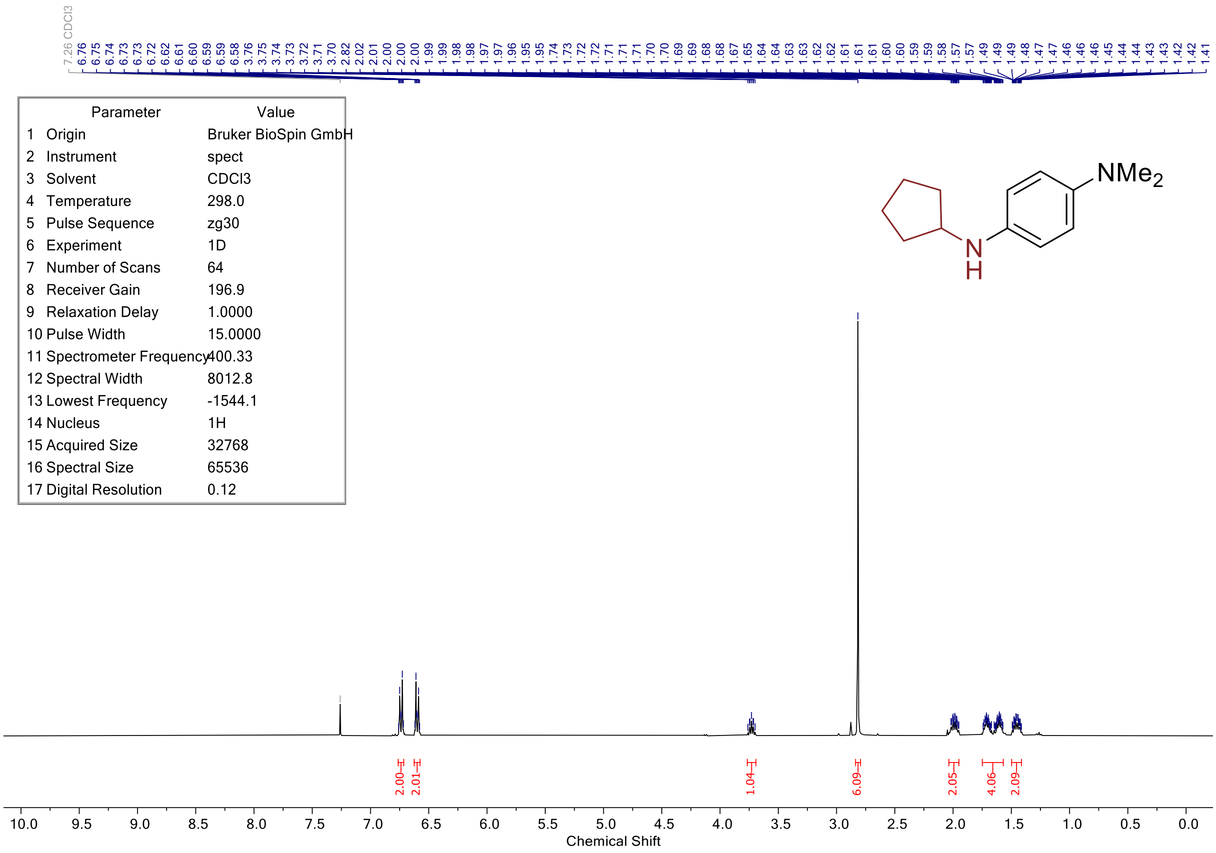

Figure S92. ^{1}H NMR spectrum of **37**.

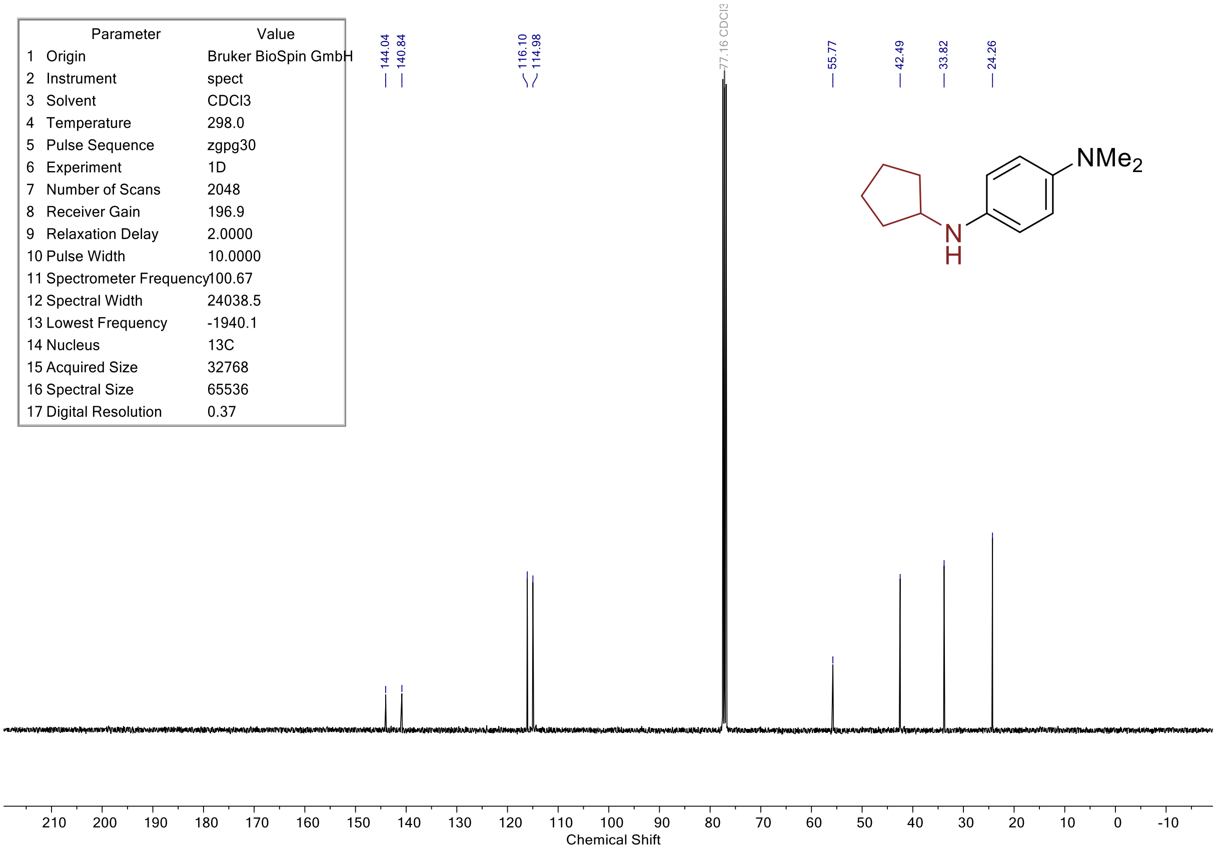

Figure S93. $^{13}C\{^{1}H\}$ NMR spectrum of **37**.

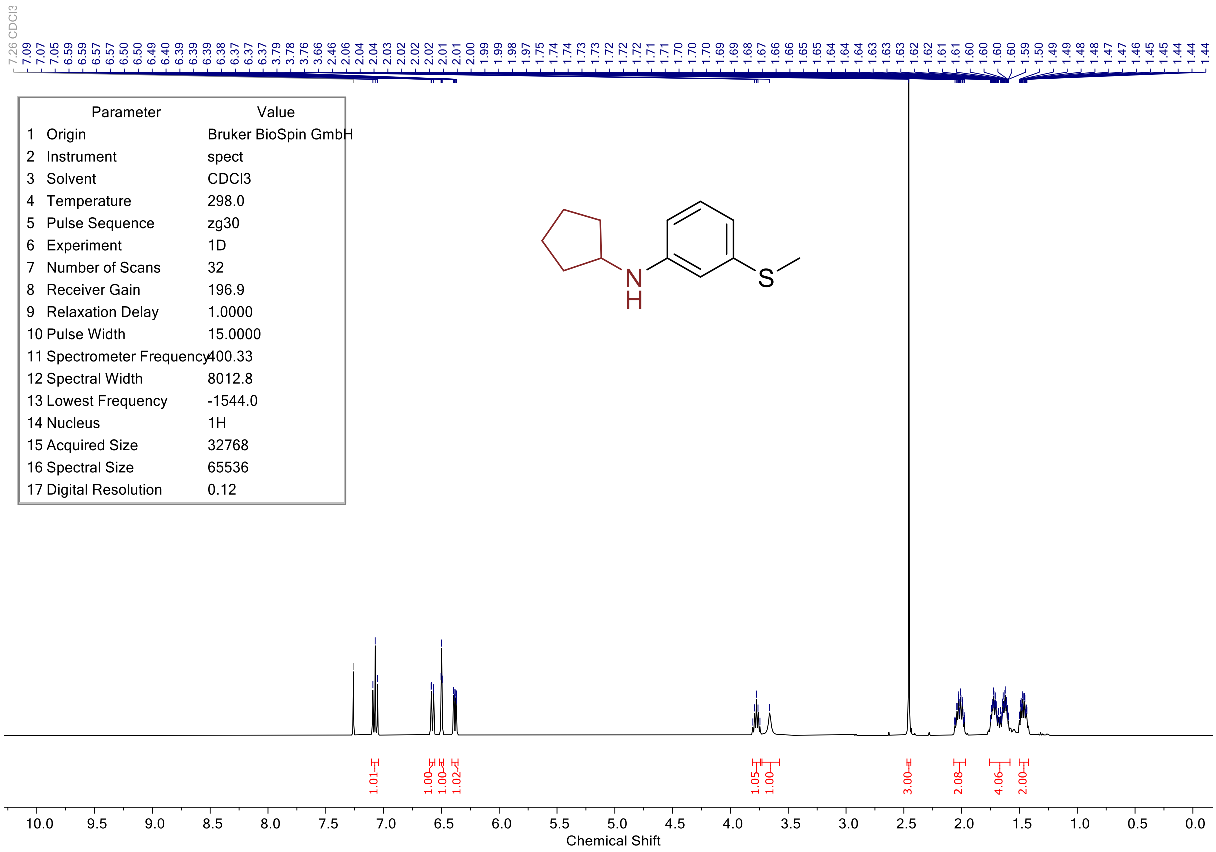

Figure S94. ^{1}H NMR spectrum of **38**.

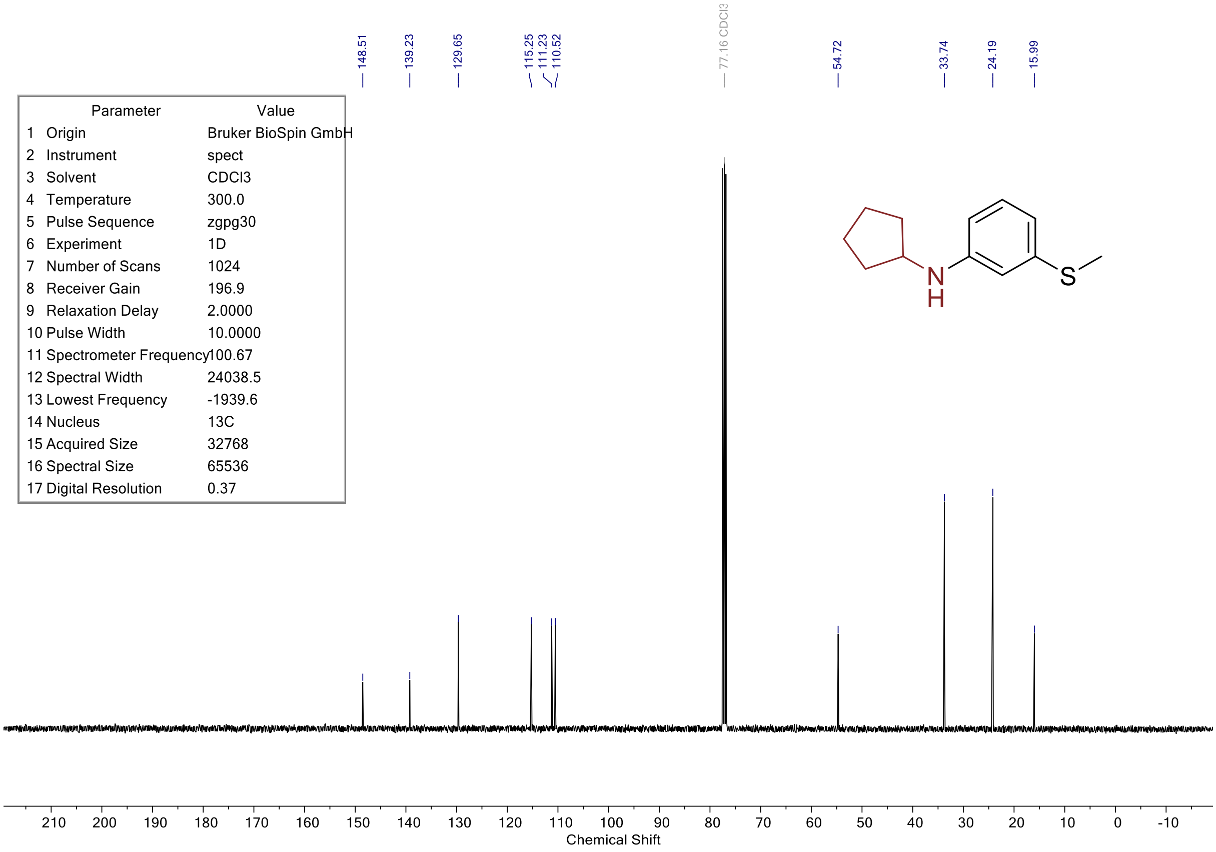

Figure S95. $^{13}C\{^{1}H\}$ NMR spectrum of **38**.

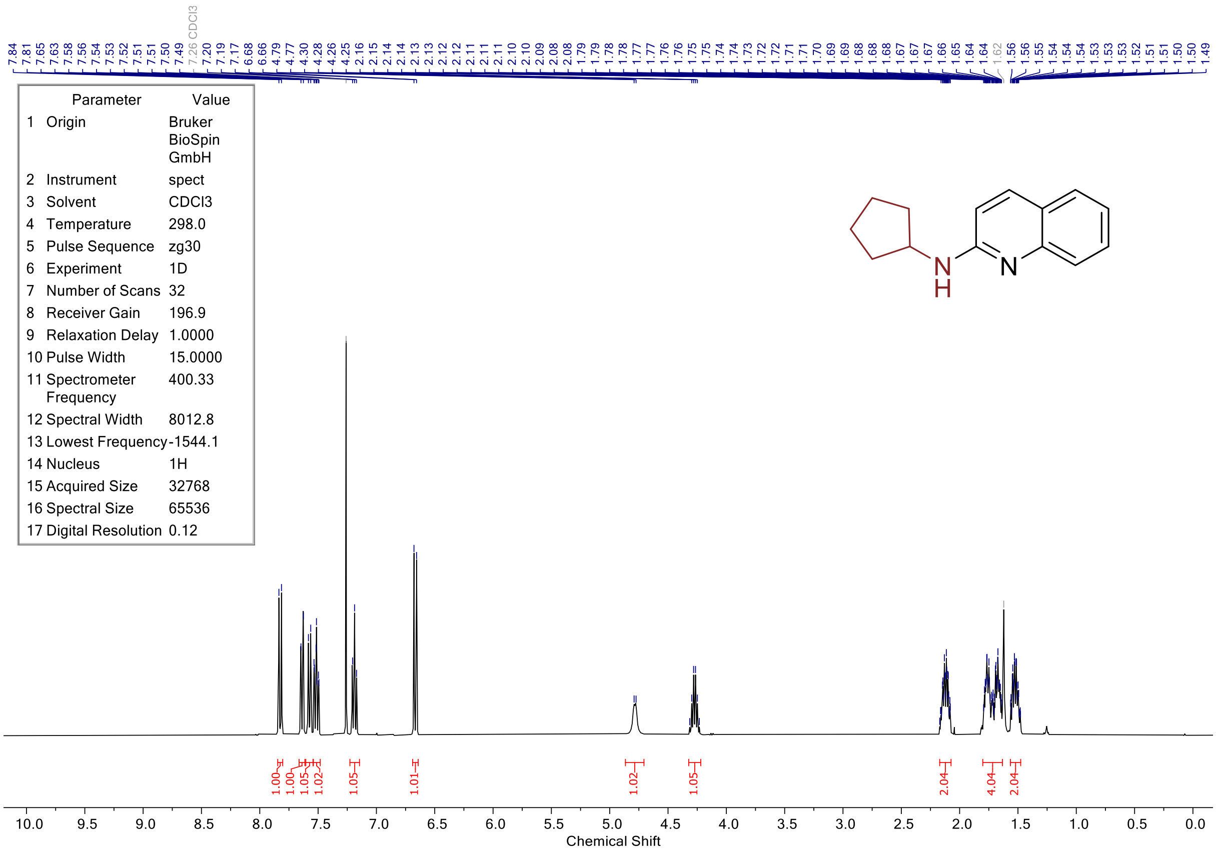

Figure S96. ^{1}H NMR spectrum of **39**.

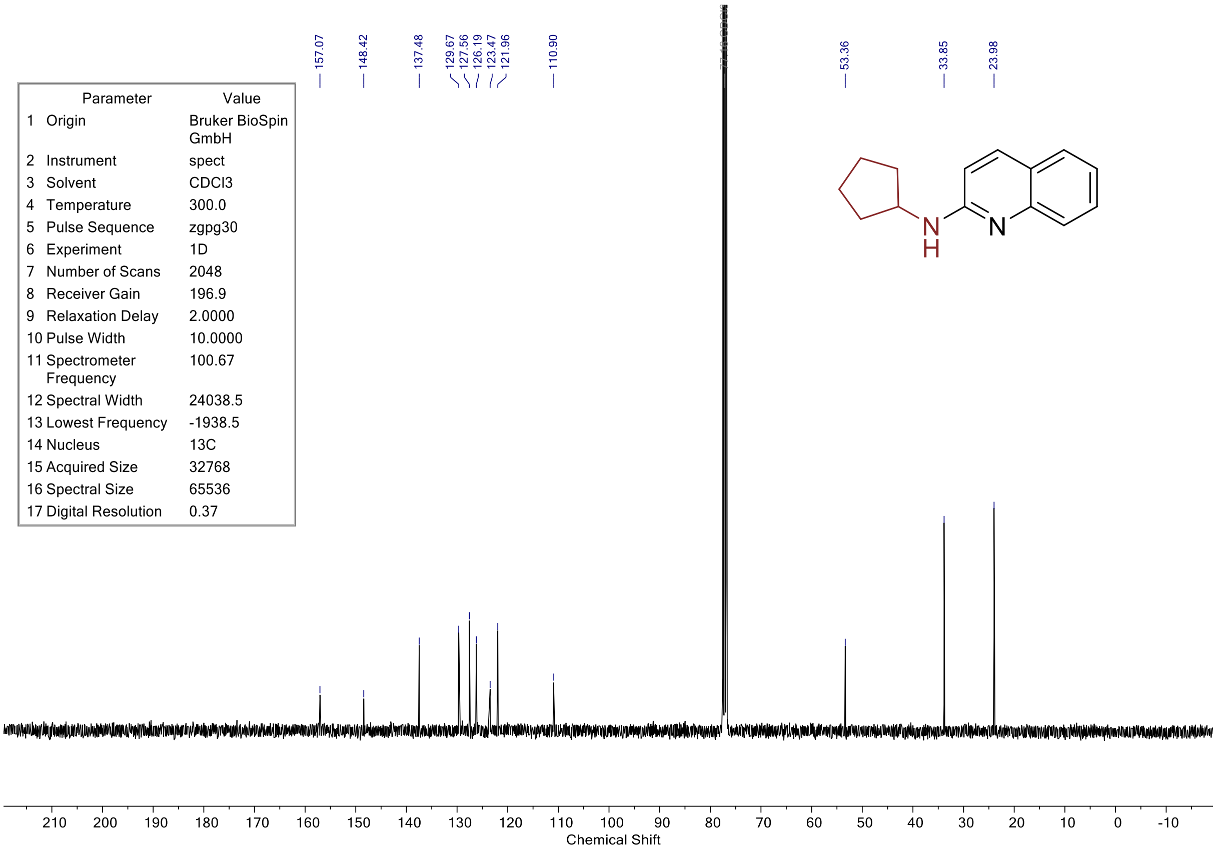

	Parameter	Value
1	Origin	Bruker BioSpin GmbH
2	Instrument	spect
3	Solvent	CDCl3
4	Temperature	300.0
5	Pulse Sequence	zgpg30
6	Experiment	1D
7	Number of Scans	2048
8	Receiver Gain	196.9
9	Relaxation Delay	2.0000
10	Pulse Width	10.0000
11	Spectrometer Frequency	100.67
12	Spectral Width	24038.5
13	Lowest Frequency	-1938.5
14	Nucleus	13C
15	Acquired Size	32768
16	Spectral Size	65536
17	Digital Resolution	0.37

Figure S97. $^{13}C\{^{1}H\}$ NMR spectrum of **39**.

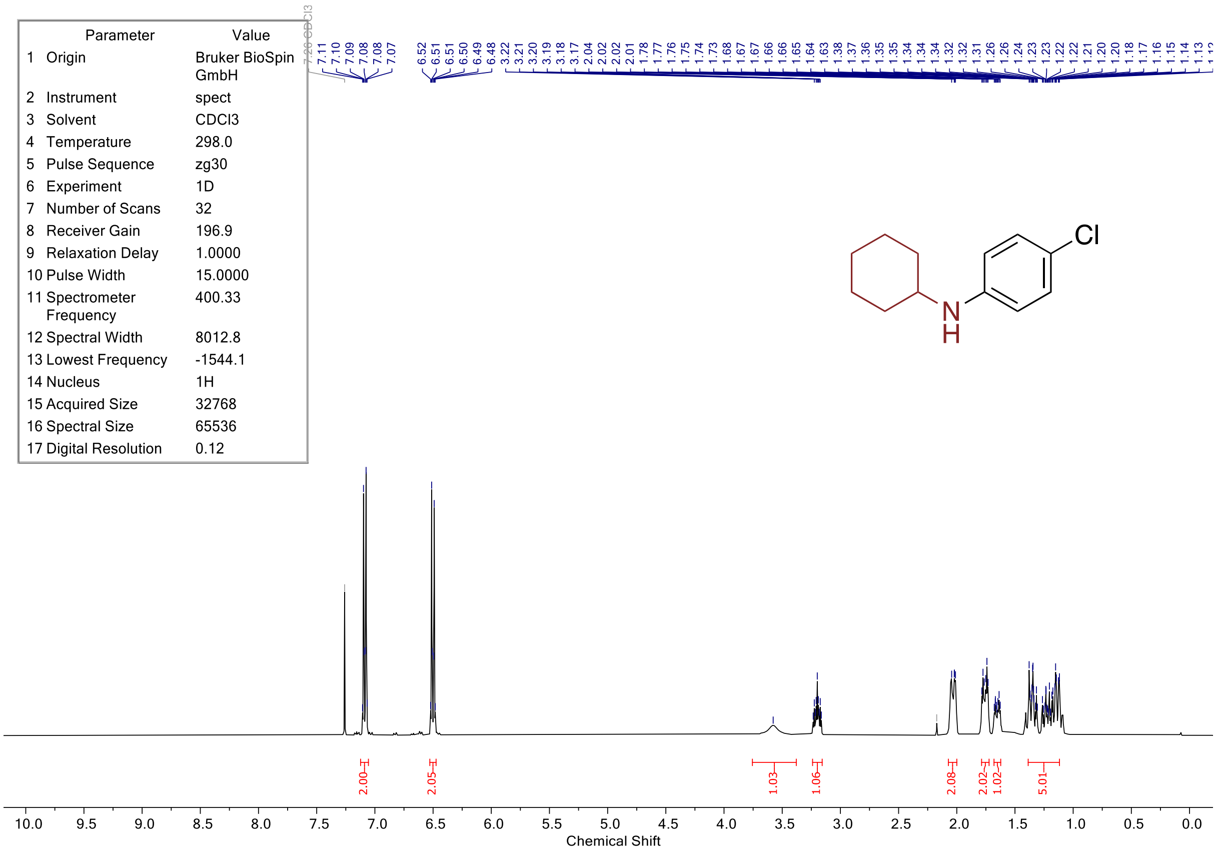

	Parameter	Value
1	Origin	Bruker BioSpin GmbH
2	Instrument	spect
3	Solvent	CDCl3
4	Temperature	298.0
5	Pulse Sequence	zg30
6	Experiment	1D
7	Number of Scans	32
8	Receiver Gain	196.9
9	Relaxation Delay	1.0000
10	Pulse Width	15.0000
11	Spectrometer Frequency	400.33
12	Spectral Width	8012.8
13	Lowest Frequency	-1544.1
14	Nucleus	1H
15	Acquired Size	32768
16	Spectral Size	65536
17	Digital Resolution	0.12

Figure S98. ^{1}H NMR spectrum of **40**.

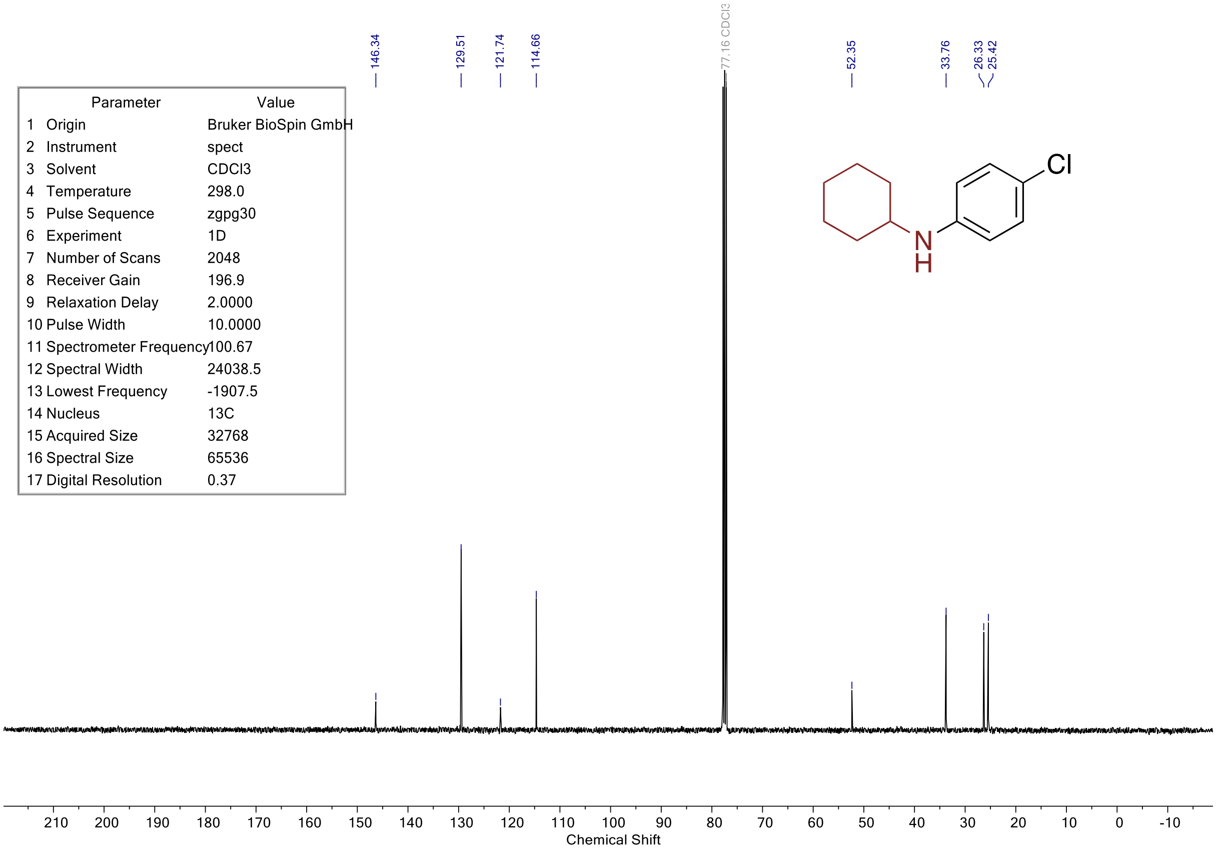

Figure S99. $^{13}C\{^{1}H\}$ NMR spectrum of **40**.

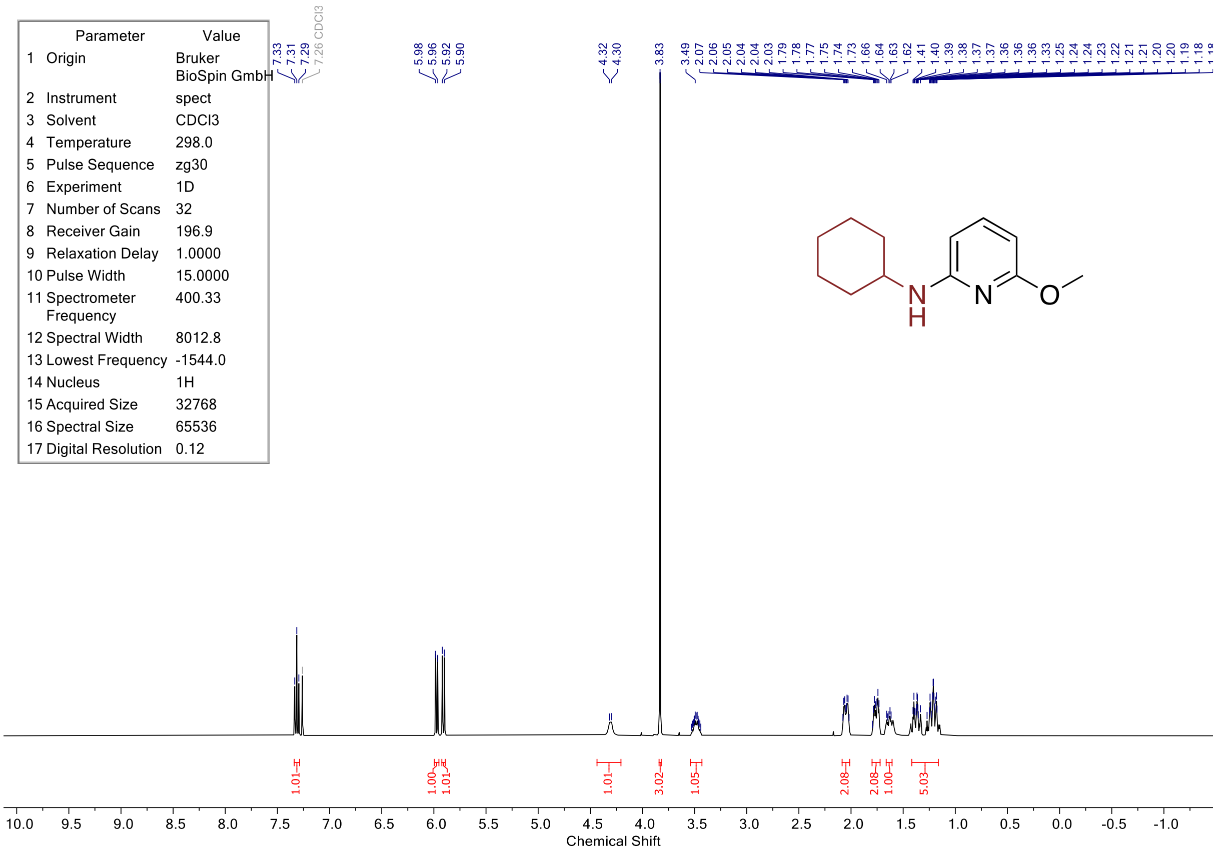

Figure S100. ^{1}H NMR spectrum of **41**.

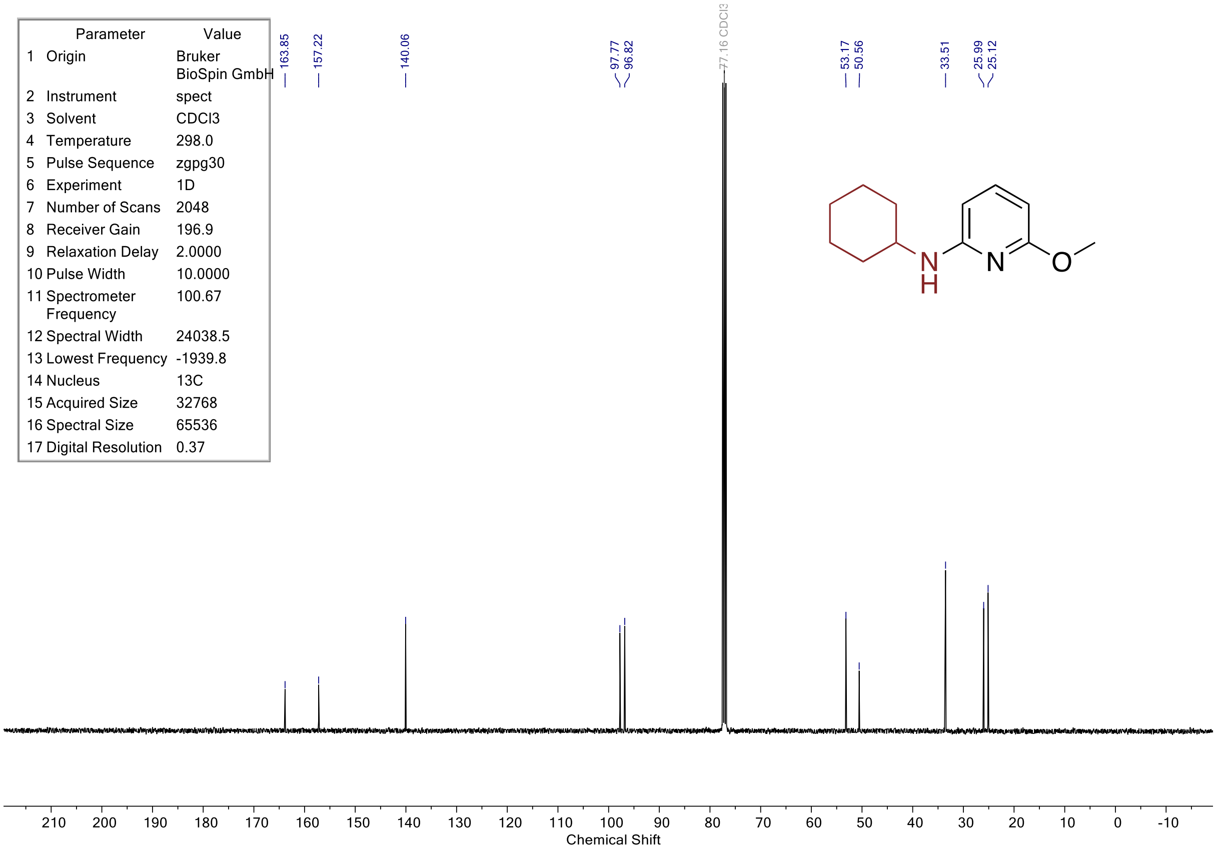

Figure S101. $^{13}C\{^{1}H\}$ NMR spectrum of **41**.

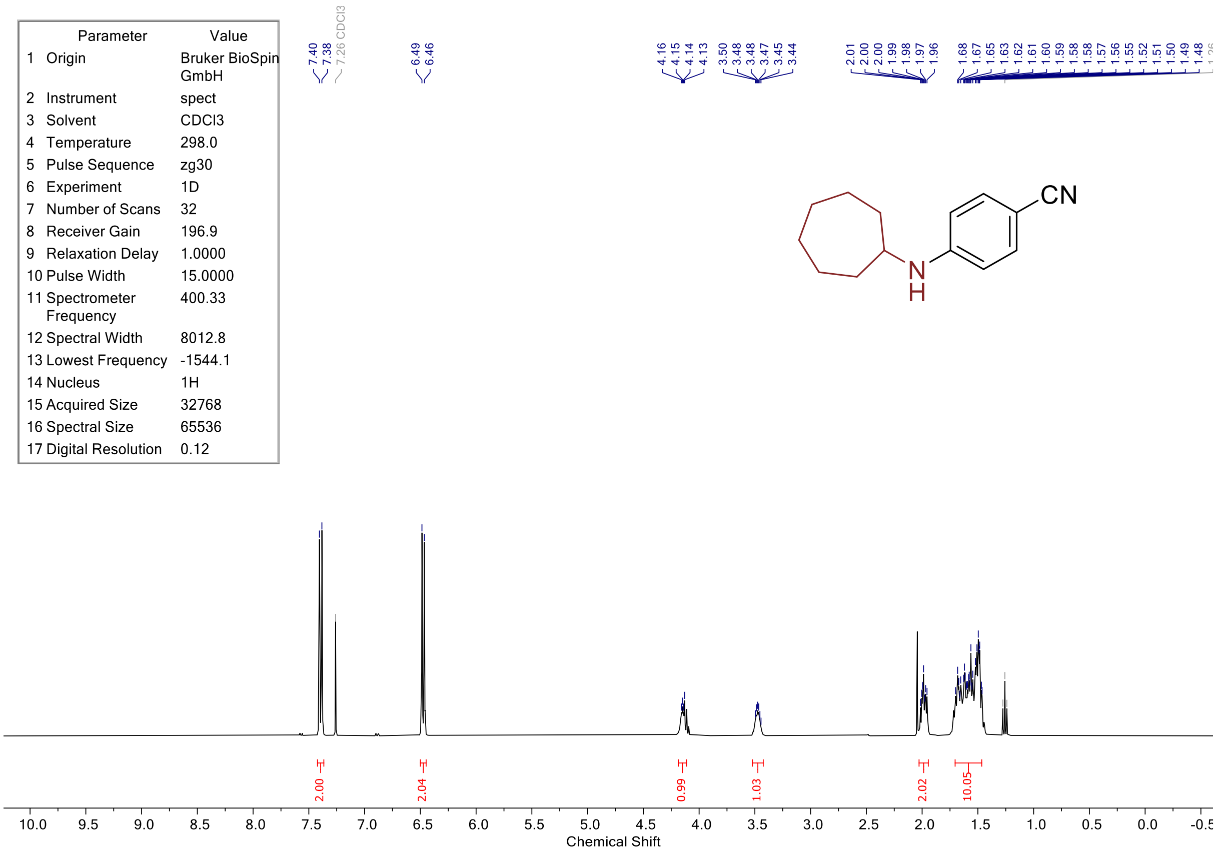

Figure S102. ^{1}H NMR spectrum of **42**.

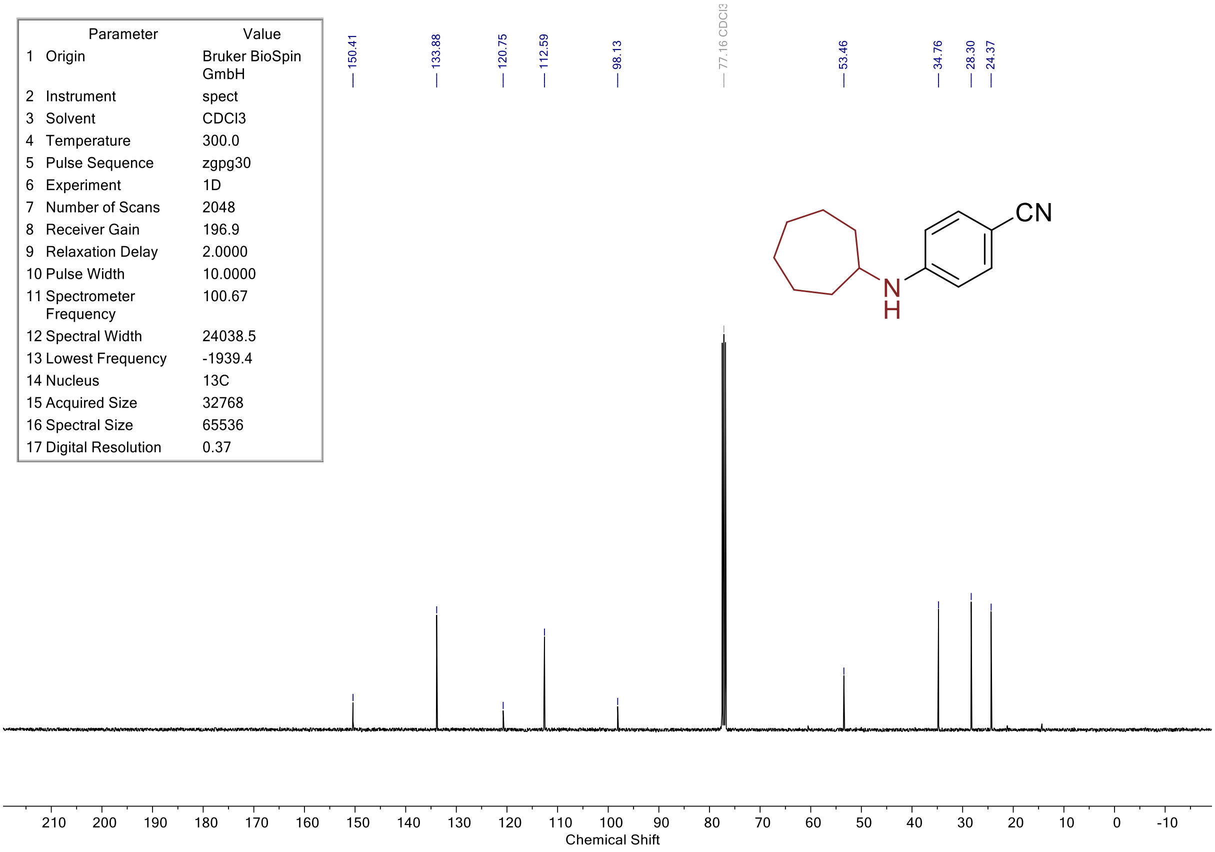

Parameter		Value
1	Origin	Bruker BioSpin GmbH
2	Instrument	spect
3	Solvent	CDCl3
4	Temperature	300.0
5	Pulse Sequence	zgpg30
6	Experiment	1D
7	Number of Scans	2048
8	Receiver Gain	196.9
9	Relaxation Delay	2.0000
10	Pulse Width	10.0000
11	Spectrometer Frequency	100.67
12	Spectral Width	24038.5
13	Lowest Frequency	-1939.4
14	Nucleus	13C
15	Acquired Size	32768
16	Spectral Size	65536
17	Digital Resolution	0.37

Figure S103. $^{13}C\{^{1}H\}$ NMR spectrum of **42**.

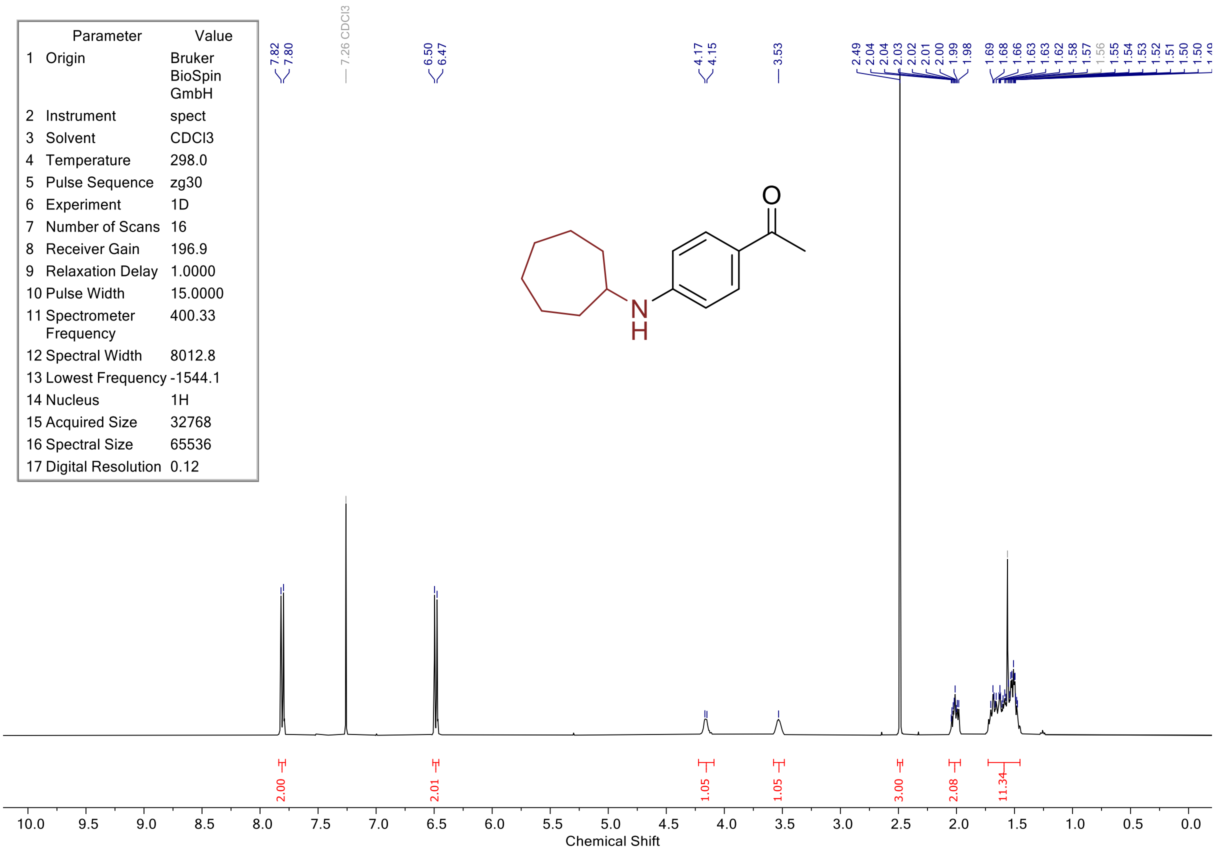

Parameter		Value
1	Origin	Bruker BioSpin GmbH
2	Instrument	spect
3	Solvent	CDCl3
4	Temperature	298.0
5	Pulse Sequence	zg30
6	Experiment	1D
7	Number of Scans	16
8	Receiver Gain	196.9
9	Relaxation Delay	1.0000
10	Pulse Width	15.0000
11	Spectrometer Frequency	400.33
12	Spectral Width	8012.8
13	Lowest Frequency	-1544.1
14	Nucleus	1H
15	Acquired Size	32768
16	Spectral Size	65536
17	Digital Resolution	0.12

Figure S104. ^{1}H NMR spectrum of **43**.

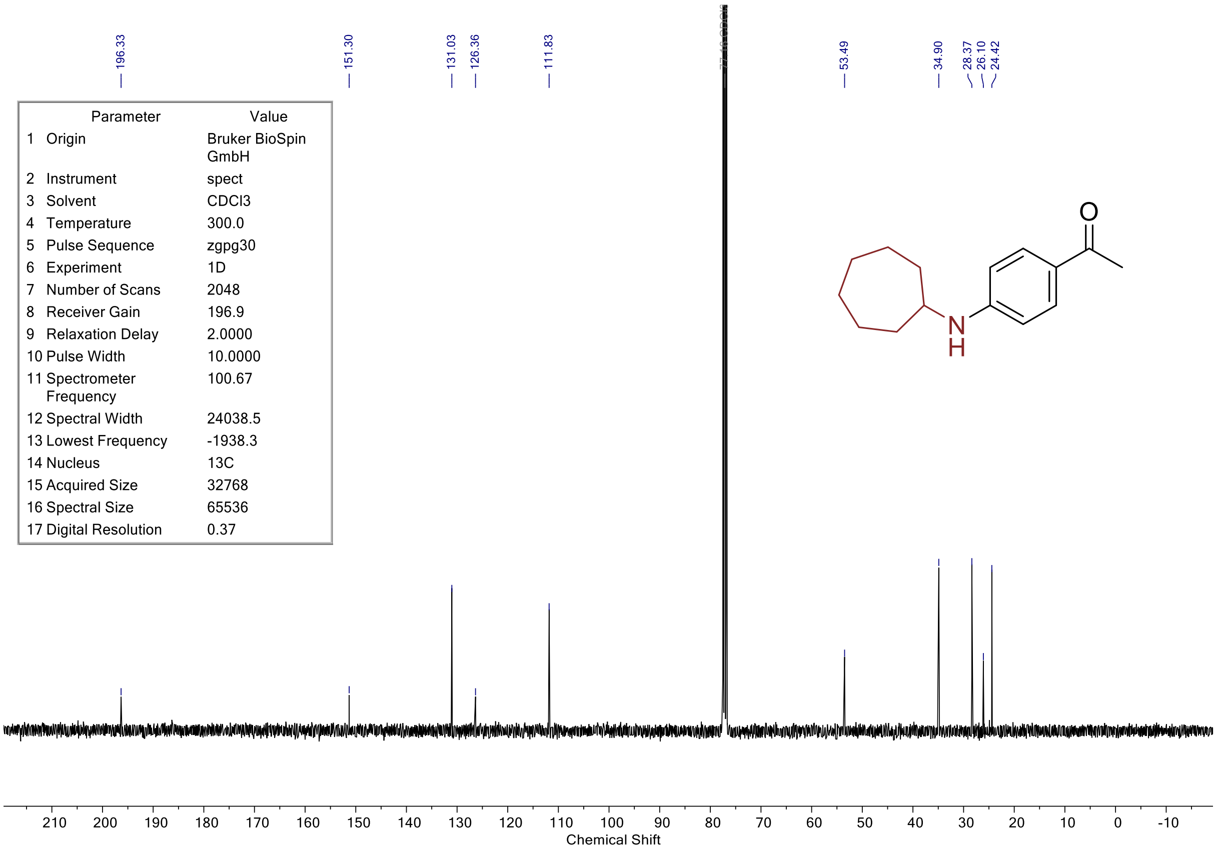

Figure S105. $^{13}C\{^{1}H\}$ NMR spectrum of **43**.

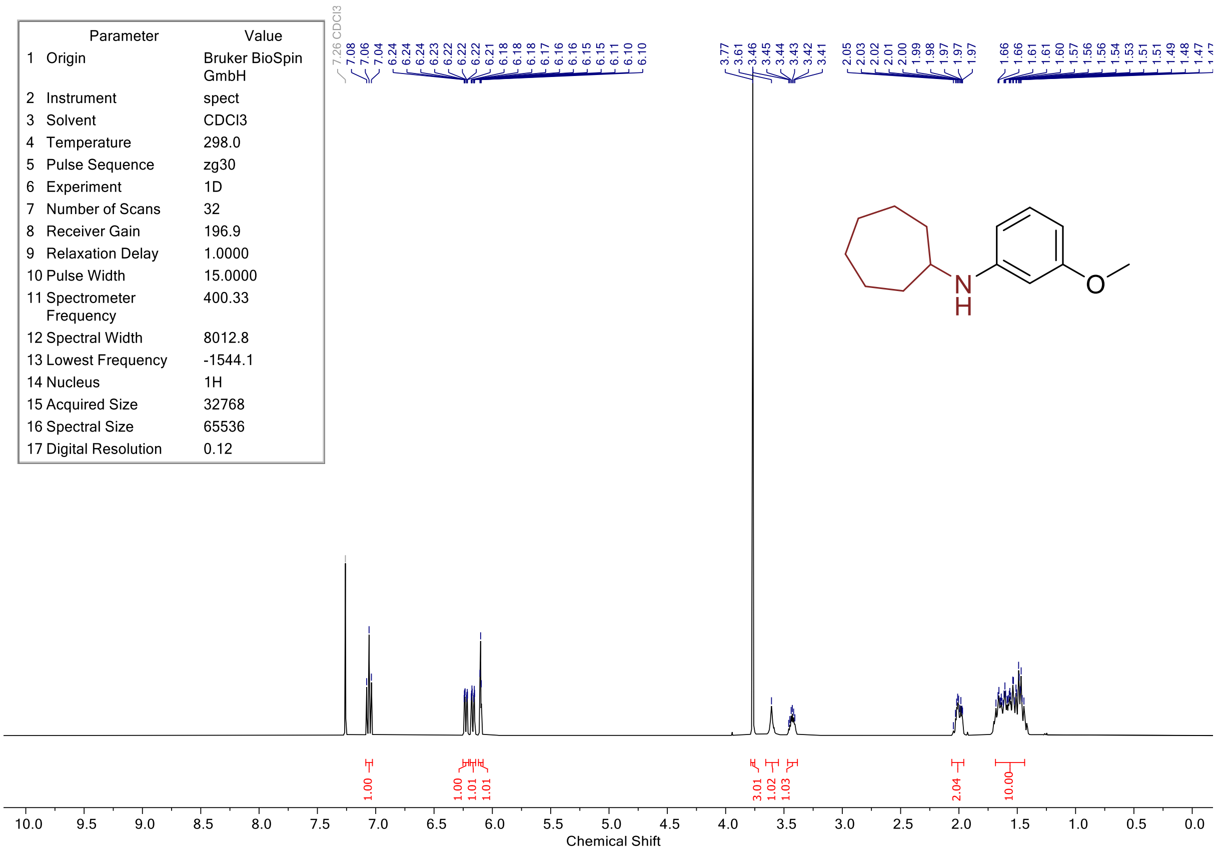

Figure S106. ^{1}H NMR spectrum of **44**.

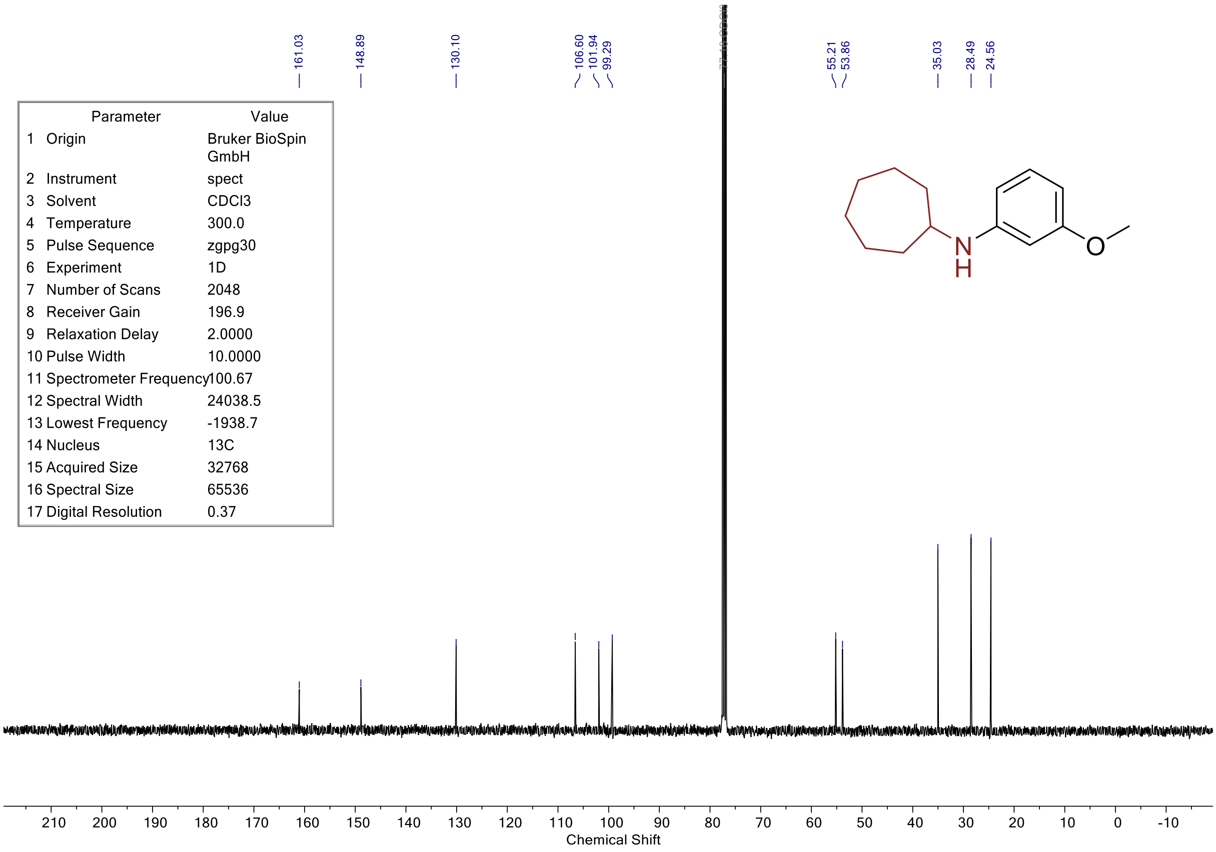

Figure S107. $^{13}C\{^{1}H\}$ NMR spectrum of **44**.

5 Crystal Structure Determination

L5: One reflection (4 20 0) was ignored by OMIT restrain.

L5-Rh(acac)CO: Highly disordered dichloromethane solvent molecules were treated by using PLATON/SQUEEZE routine.

L5-AuCl: The structure was refined as an inversion twin.

Table S11. Data collection and structure refinement details for compound **L5**, **L5-AuCl** and **L5-Rh(acac)CO**.

Compound	L5	L5-AuCl	L5-Rh(acac)CO
CCDC No.	2449591	2449593	2449592
Formula	$C_{46}H_{72}P_2$	$C_{42}H_{70}AuCl_5P_2$	$C_{47}H_{75}Cl_2O_3P_2Rh$
Formula weight [g · mol^{-1}]	686.97	1011.13	923.82
Temperature [K]	100(2)	100(2)	100(2)
Wavelength [Å]	1.54184	1.54184	1.54184
Crystal system	Orthorhombic	Orthorhombic	Triclinic
Space group	Pba_2	$P2_12_12_1$	$P\bar{1}$
a [Å]	31.3271(3)	10.64810(10)	10.91540(10)
b [Å]	22.1147(2)	18.98550(10)	12.61250(10)
c [Å]	11.40710(10)	21.28230(10)	35.0275(2)
α [°]	90	90	98.9920(10)
β [°]	90	90	90.5430(10)
γ [°]	90	90	94.8220(10)
Volume [Å^3]	7902.72(13)	4302.42(5)	4744.88(7)
Z	8	4	4
Calc. density [Mg·m^{-3}]	1.155	1.561	1.293
μ [mm^{-1}]	1.209	10.197	4.866
F(000)	3024	2064	1960
Crystal dimensions [mm]	0.550 x 0.310 x 0.270	0.140 x 0.050 x 0.040	0.340 x 0.220 x 0.180
Theta range [°]	2.446 to 67.076	3.119 to 76.826	2.555 to 67.078
Index range	-37 <= h <= 36,<br>-12 <= k< = 26,<br>-13 <= l< = 13	-13 <= h <= 12,<br>-23 <= k< = 23,<br>-22 <= l< = 26	-10<=h<=13,<br>-15<=k<=15,<br>-41<=l<=41
Reflections collected	30905	55748	59317
Independent reflections	11718 [R(int) = 0.0449]	8894 [R(int) = 0.0664]	16852 [R(int) = 0.0355]
Completeness [%] to theta	99.9	100	99.7
Absorption correction	Gaussian	Gaussian	Gaussian
Max. and min. transmission	1.000 and 0.178	0.970 and 0.428	1.000 and 0.249
Refinement method	Full-matrix least-squares on F^2	Full-matrix least-squares on F^2	Full-matrix least-squares on F^2
Data/Restraints/Parameter	11718 / 1 / 868	8894 / 0 / 453	16852 / 0 / 997
Goodness-of-fit on F^2	1.097	1.079	1.036
Final R indices [I>2sigma(I)]	R1 = 0.0444,<br>wR2 = 0.1143	R1 = 0.0303,<br>wR2 = 0.798	R1 = 0.0471,<br>wR2 = 0.1333
R indices (all data)	R1 = 0.0469,<br>wR2 = 0.1161	R1 = 0.0314,<br>wR2 = 0.0805	R1 = 0.0486,<br>wR2 = 0.1347
Largest diff. peak and hole [e · Å^{-3}]	0.579 and -0.263	1.166 and -0.810	1.894 and -1.716

Table S12. Data collection and structure refinement details for compounds **3**, **7** and **10-O₂**.

Compound	3	7	10-O₂
CCDC No.	2449590	2449588	2449587
Formula	$C_{10}H_{10}N_2$	$C_{12}H_{15}NO_2$	$C_9H_{10}ClNO_2$
Formula weight [g · mol^{-1}]	158.20	205.25	199.63
Temperature [K]	100(2)	100(2)	100(2)
Wavelength [Å]	1.54184	1.54184	1.54184
Crystal system	Orthorhombic	Triclinic	Monoclinic
Space group	$P2_12_12_1$	$P\bar{1}$	$P2_1/c$
a [Å]	7.1566(6)	9.1225(2)	11.3288(2)
b [Å]	8.8922(8)	11.1582(3)	11.5505(2)
c [Å]	13.3149(13)	11.9400(2)	6.95810(10)
α [°]	90	97.448(2)	90
β [°]	90	95.703(2)	90.243(2)
γ [°]	90	112.894(2)	90
Volume [Å^3]	847.33(13)	1095.16(5)	910.48(3)
Z	4	4	4
Calc. density [Mg·m^{-3}]	1.240	1.245	1.456
μ [mm^{-1}]	0.591	0.683	3.443
F(000)	336	440	416
Crystal dimensions [mm]	0.177 x 0.121 x 0.077	0.364 x 0.222 x 0.203	0.344 x 0.284 x 0.072
Theta range [°]	5.984 to 67.063	3.785 to 67.072	3.902 to 67.068
Index range	-8 <= h <= 5,<br>-8 <= k< = 10,<br>-15 <= l< = 15	-10 <= h <= 10,<br>-13 <= k< = 13,<br>-10 <= l< = 14	-13 <= h <= 13,<br>-10 <= k< = 13,<br>-8 <= l< = 8
Reflections collected	2542	12024	9640
Independent reflections	1345 [R(int) = 0.0334]	3915 [R(int) = 0.0304]	1633 [R(int) = 0.0435]
Completeness [%] to theta	98.6	100.0	100.0
Absorption correction	Gaussian	Gaussian	Gaussian
Max. and min. transmission	1.000 and 0.775	1.000 and 0.377	1.000 and 0.240
Refinement method	Full-matrix least-squares on F^2	Full-matrix least-squares on F^2	Full-matrix least-squares on F^2
Data/Restraints/Parameter	1345 / 0 / 112	3915 / 0 / 280	1633 / 0 / 123
Goodness-of-fit on F^2	1.068	1.062	1.063
Final R indices [I>2sigma(I)]	R1 = 0.0382,<br>wR2 = 0.0978	R1 = 0.0337,<br>wR2 = 0.0876	R1 = 0.0293,<br>wR2 = 0.0798
R indices (all data)	R1 = 0.0414,<br>wR2 = 0.1000	R1 = 0.0371,<br>wR2 = 0.0904	R1 = 0.0312,<br>wR2 = 0.0811
Largest diff. peak and hole [e · Å^{-3}]	0.182 and -0.217	0.205 and -0.183	0.208 and -0.236

Table S13. Data collection and structure refinement details for compounds **23**, **24-O_2**and **31**.

Compound	23	24-O_2	31
CCDC No.	2449589	2449584	2449586
Formula	$C_8H_{10}N_2$	$C_9H_{12}N_2O_3$	$C_9H_{12}N_2$
Formula weight [g · mol^{-1}]	134.18	196.21	148.21
Temperature [K]	100(2)	100(2)	100(2)
Wavelength [Å]	1.54184	1.54184	1.54184
Crystal system	Monoclinic	Monoclinic	Orthorhombic
Space group	$P2_1/c$	$P2_1/n$	$P2_12_12_1$
a [Å]	9.6445(2)	8.6364(2)	8.18590(10)
b [Å]	8.43700(10)	7.0675(2)	9.86020(10)
c [Å]	9.7506(2)	15.5253(3)	9.92650(10)
α [°]	90	90	90
β [°]	109.938(2)	91.081(2)	90
γ [°]	90	90	90
Volume [Å^3]	745.86(3)	947.46(4)	801.214(15)
Z	4	4	4
Calc. density [Mg·m^{-3}]	1.195	1.375	1.229
μ [mm^{-1}]	0.575	0.876	0.581
F(000)	288	416	320
Crystal dimensions [mm]	0.275 x 0.148 x 0.064	0.274 x 0.103 x 0.029	0.376 x 0.247 x 0.172
Theta range [°]	4.878 to 67.017	5.701 to 67.065	6.327 to 66.820
Index range	-11 <= h <= 11, -10 <= k< = 9, -11 <= l< = 9	-10 <= h <= 10, -8 <= k< = 7, -18 <= l< = 18	-7 <= h <= 9, -11 <= k< = 11, -11 <= l< = 11
Reflections collected	4679	6016	8513
Independent reflections	1331 [R(int) = 0.0323]	1690 [R(int) = 0.0382]	1433 [R(int) = 0.0354]
Completeness [%] to theta	100.0	99.6	100.0
Absorption correction	Gaussian	Gaussian	Gaussian
Max. and min. transmission	1.000 and 0.440	1.000 and 0.767	1.000 and 0.369
Refinement method	Full-matrix least-squares on F^2	Full-matrix least-squares on F^2	Full-matrix least-squares on F^2
Data/Restraints/Parameter	1331 / 0 / 95	1690 / 0 / 134	1433 / 0 / 104
Goodness-of-fit on F^2	1.072	1.053	1.095
Final R indices [I>2sigma(I)]	R1 = 0.0327, wR2 = 0.0867	R1 = 0.0336, wR2 = 0.0928	R1 = 0.0303, wR2 = 0.0775
R indices (all data)	R1 = 0.0352, wR2 = 0.0896	R1 = 0.0369, wR2 = 0.0952	R1 = 0.0306, wR2 = 0.0776
Largest diff. peak and hole [e · Å^{-3}]	0.180 and -0.156	0.192 and -0.217	0.182 and -0.138

Table S14. Data collection and structure refinement details for compound **40**.

Compound	41
CCDC No.	2449585
Formula	$C_{15}H_{21}NO$
Formula weight [g · mol^{-1}]	231.33
Temperature [K]	100(2)
Wavelength [Å]	1.54184
Crystal system	Monoclinic
Space group	$P2_1/n$
a [Å]	13.63020(10)
b [Å]	14.17620(10)
c [Å]	13.78410(10)
α [°]	90
β [°]	97.0710(10)
γ [°]	90
Volume [Å^3]	2643.17(3)
Z	8
Calc. density [Mg·m^{-3}]	1.163
μ [mm^{-1}]	0.557
F(000)	1008
Crystal dimensions [mm]	0.339 x 0.242 x 0.161
Theta range [°]	4.304 to 67.080
Index range	-16 <= h <= 16, -13 <= k< = 16, -16 <= l< = 16
Reflections collected	31836
Independent reflections	4729 [R(int) = 0.0394]
Completeness [%] to theta	100.0
Absorption correction	Gaussian
Max. and min. transmission	1.000 and 0.399
Refinement method	Full-matrix least-squares on F^2
Data/Restraints/Parameter	4729 / 0 / 315
Goodness-of-fit on F^2	1.027
Final R indices [I>2sigma(I)]	R1 = 0.0345, wR2 = 0.0876
R indices (all data)	R1 = 0.0372, wR2 = 0.0899
Largest diff. peak and hole [e · Å^{-3}]	0.185 and -0.207

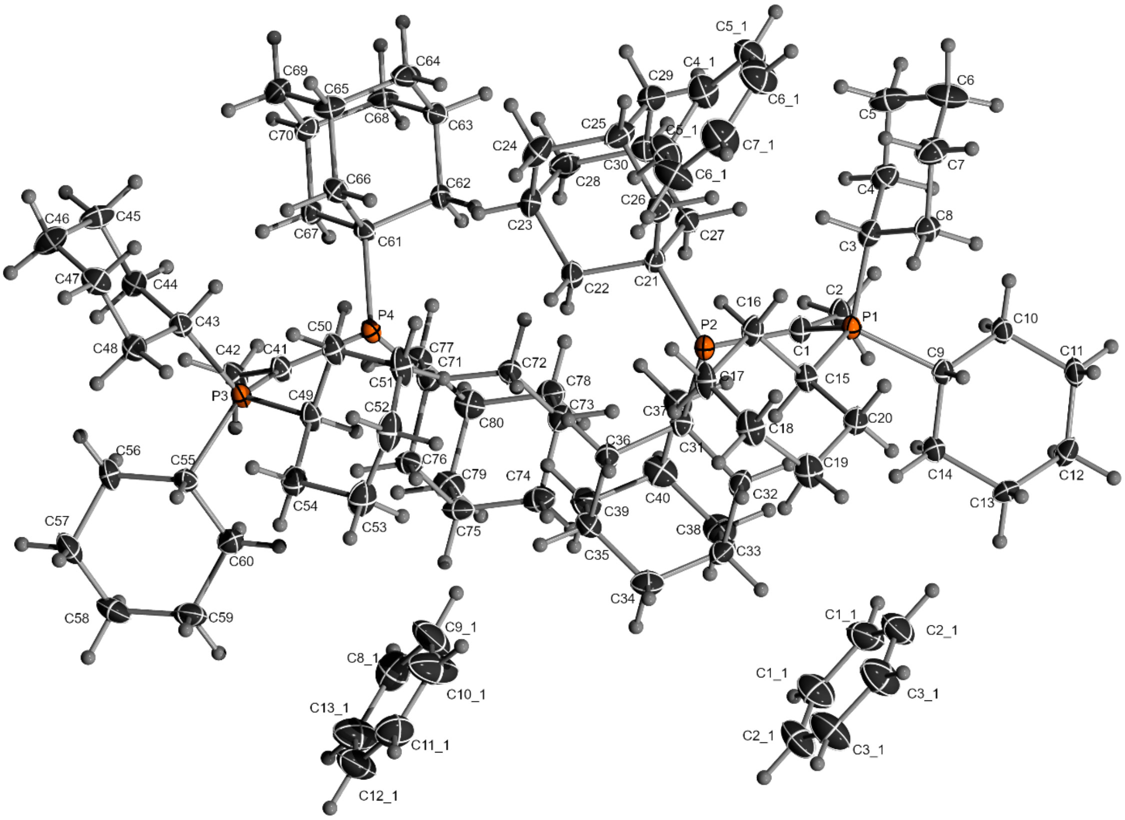

Figure S108. ORTEP Plot of **L5**. Ellipsoids with 50% radii probability.

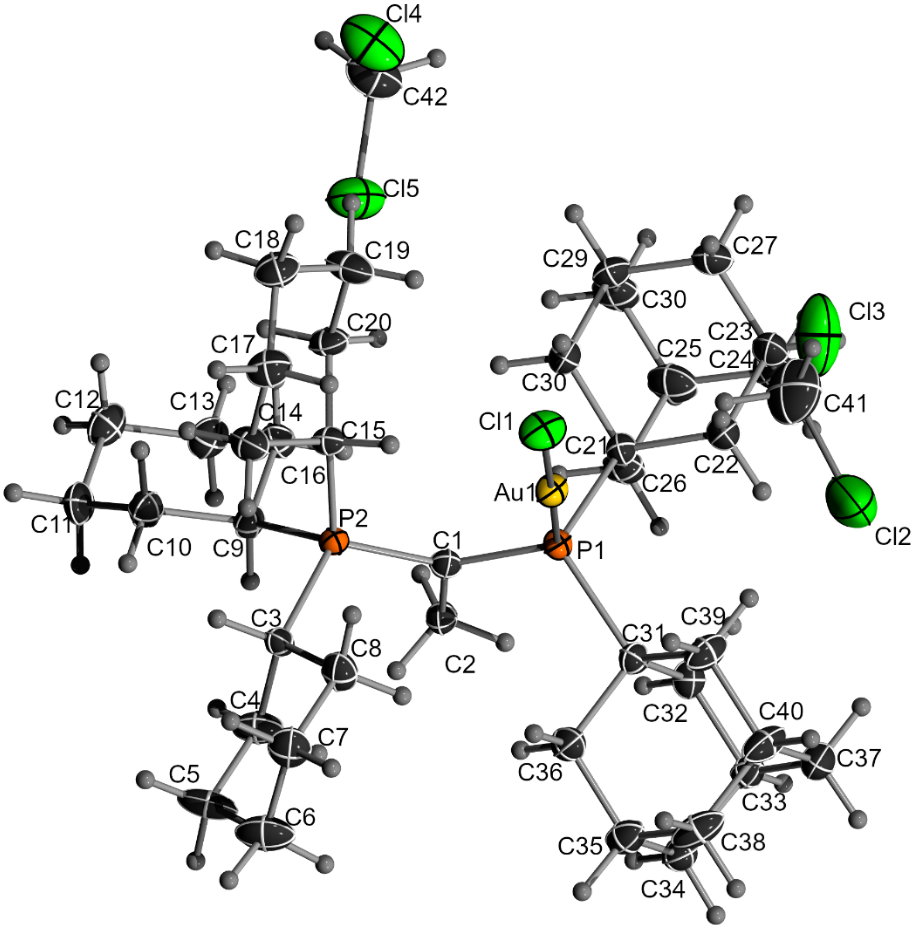

Figure S109. ORTEP Plot of **L5-AuCl**. Ellipsoids with 50% radii probability.

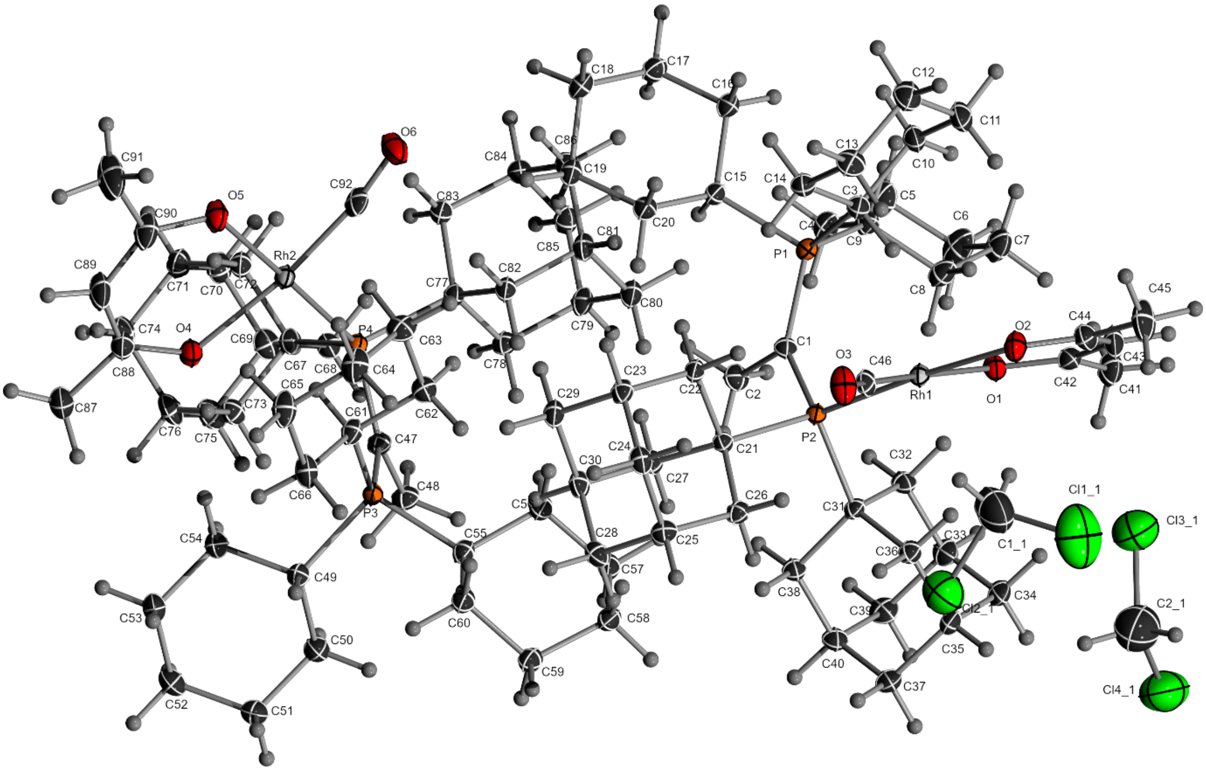

Figure S110. ORTEP Plot of **L5-Rh(acac)CO**. Ellipsoids with 50% radii probability.

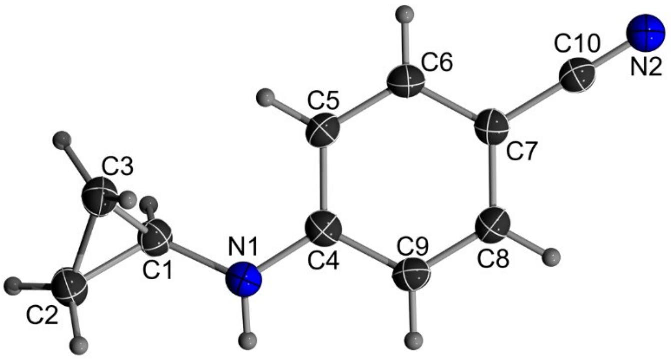

Figure S111. ORTEP Plot of **3**. Ellipsoids with 50% radii probability.

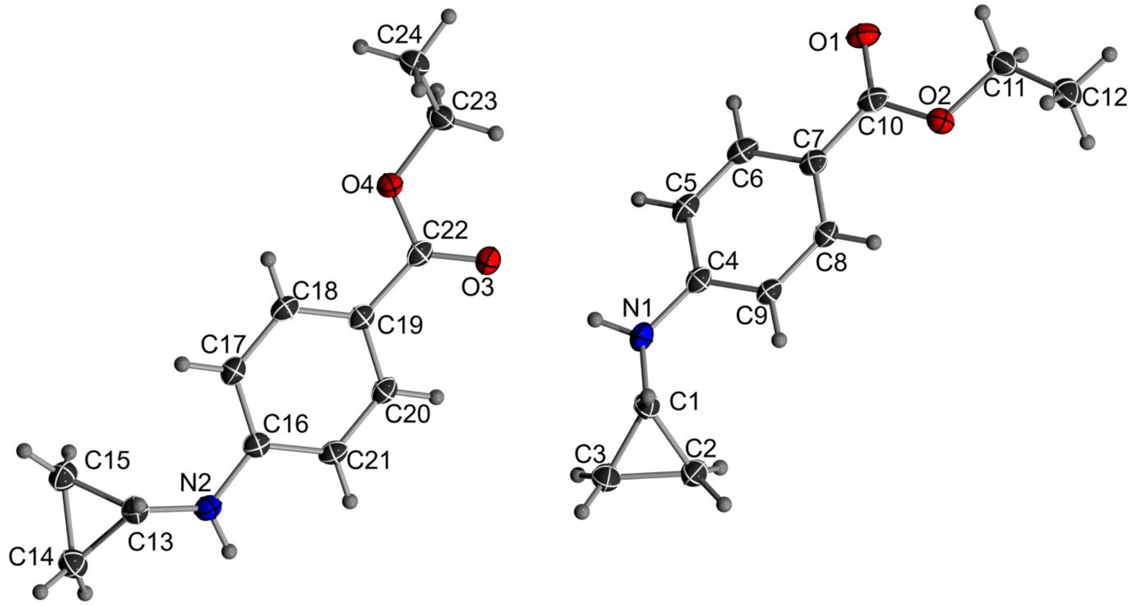

Figure S112. ORTEP Plot of **7**. Ellipsoids with 50% radii probability.

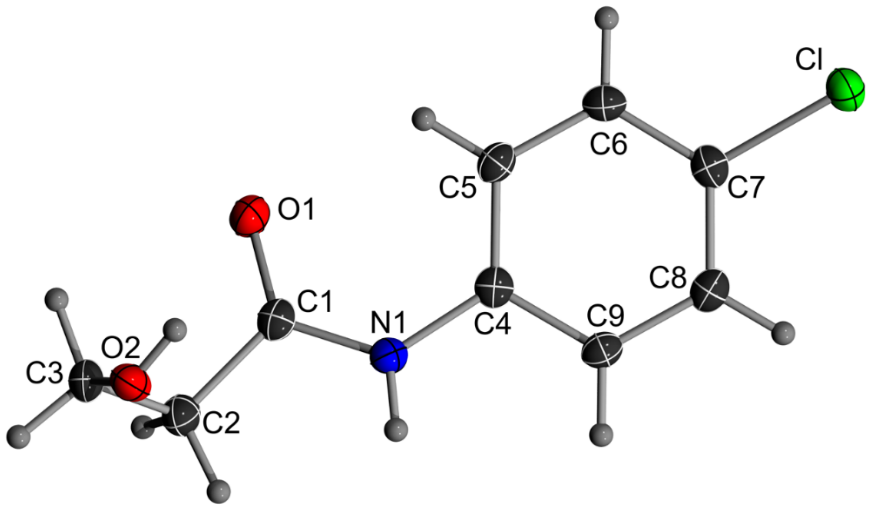

Figure S113. ORTEP Plot of **10-O$_2$**. Ellipsoids with 50% radii probability.

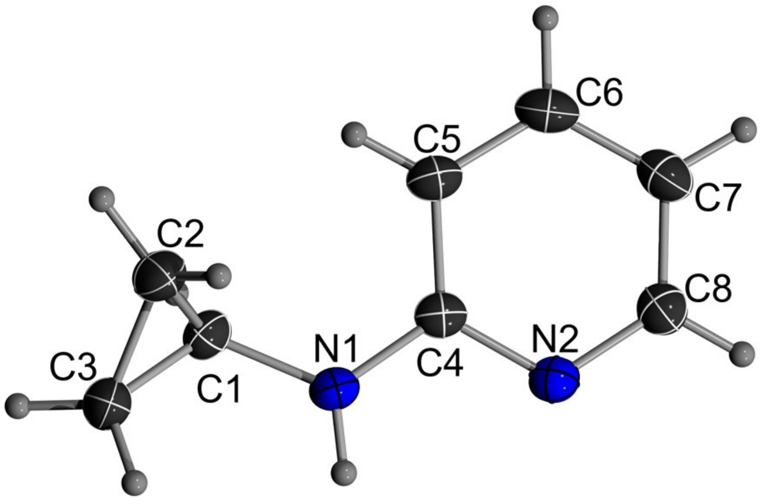

Figure S114. ORTEP Plot of **23**. Ellipsoids with 50% radii probability.

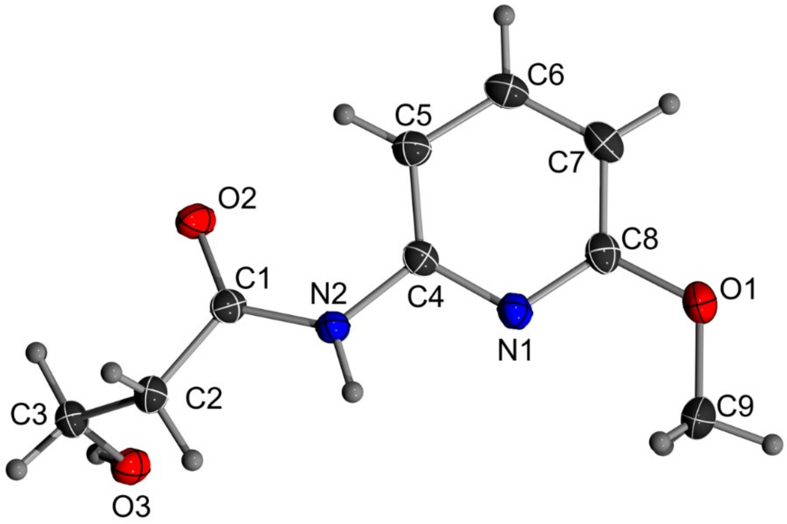

Figure S 115. ORTEP Plot of **24-O$_2$**. Ellipsoids with 50% radii probability.

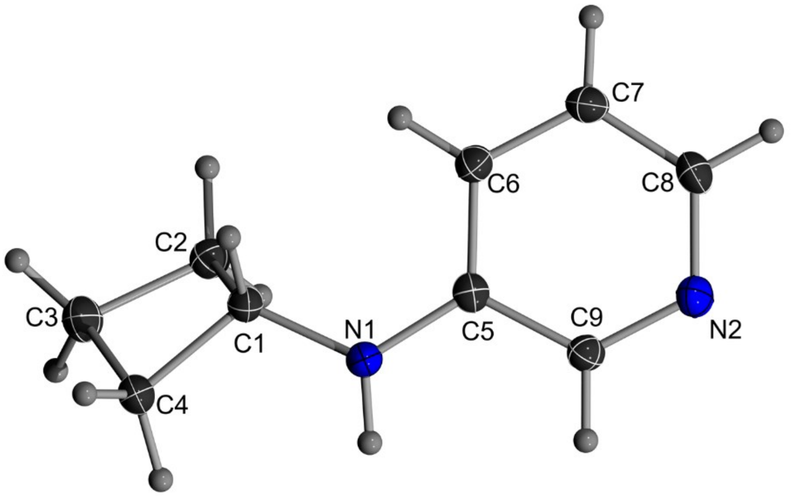

Figure S116. ORTEP Plot of **31**. Ellipsoids with 50% radii probability.

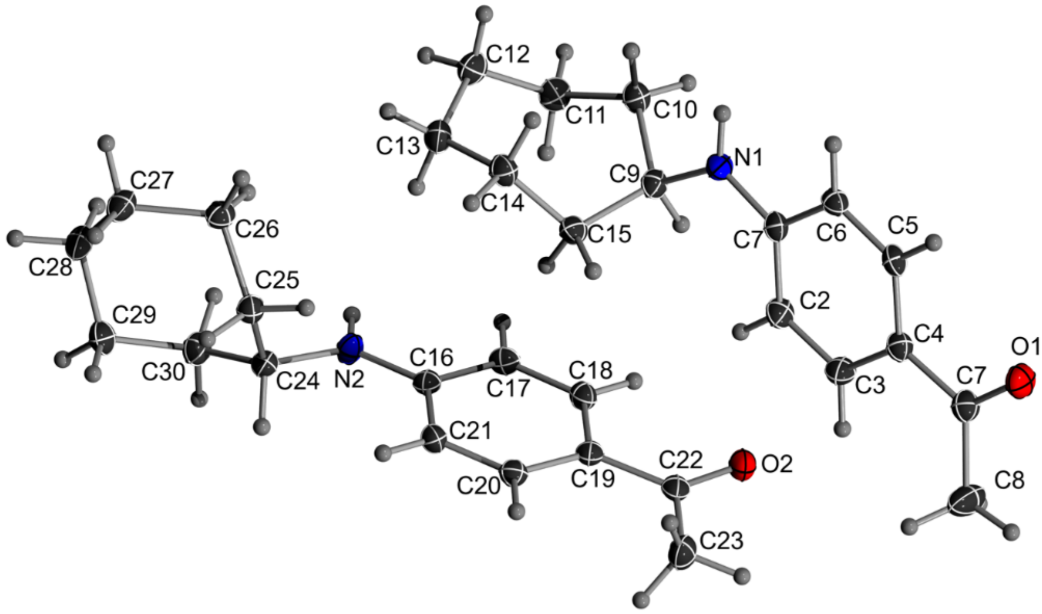

Figure S117. ORTEP Plot of **41**. Ellipsoids with 50% radii probability.

6 References

(1) Weber, P.; Scherpf, T.; Rodstein, I.; Lichte, D.; Scharf, L. T.; Gooßen, L. J.; Gessner, V. H. A Highly Active Ylide‐Functionalized Phosphine for Palladium‐Catalyzed Aminations of Aryl Chlorides. *Angew. Chem., Int. Ed.* **2019**, *58* (10), 3203–3207. DOI: 10.1002/anie.201810696.

(2) Rodstein, I.; Prendes, D. S.; Wickert, L.; Paaßen, M.; Gessner, V. H. Selective Pd-Catalyzed Monoarylation of Small Primary Alkyl Amines through Backbone-Modification in Ylide-Functionalized Phosphines (YPhos). *J. Org. Chem.* **2020**, *85* (22), 14674–14683. DOI: 10.1021/acs.joc.0c01771.

(3) Hu, X.-Q.; Lichte, D.; Rodstein, I.; Weber, P.; Seitz, A.-K.; Scherpf, T.; Gessner, V. H.; Gooßen, L. J. Ylide-Functionalized Phosphine (YPhos)–Palladium Catalysts: Selective Monoarylation of Alkyl Ketones with Aryl Chlorides. *Org. Lett.* **2019**, *21* (18), 7558–7562. DOI: 10.1021/acs.orglett.9b02830.

(4) Goebel, J. F.; Löffler, J.; Zeng, Z.; Handelmann, J.; Hermann, A.; Rodstein, I.; Gensch, T.; Gessner, V. H.; Gooßen, L. J. Computer‐Driven Development of Ylide Functionalized Phosphines for Palladium‐Catalyzed Hiyama Couplings. *Angew. Chem., Int. Ed.* **2023**, *62* (9), e202216160. DOI: 10.1002/anie.202216160.

(5) Hu, Z.; Wei, X.-J.; Handelmann, J.; Seitz, A.-K.; Rodstein, I.; Gessner, V. H.; Gooßen, L. J. Coupling of Reformatsky Reagents with Aryl Chlorides Enabled by Ylide‐Functionalized Phosphine Ligands. *Angew. Chem., Int. Ed.* **2021**, *60* (12), 6778–6783. DOI: 10.1002/anie.202016048.

(6) Löffler, J.; Kaiser, N.; Knyszek, D.; Krischer, F.; Jörges, M.; Feichtner, K.-S.; Gessner, V. H. P,N‐Coordinating Ylide‐Functionalized Phosphines (NYPhos): A Ligand Platform for the Selective Monoarylation of Small Nucleophiles. *Angew. Chem., Int. Ed.* **2024**, *63* (36). DOI: 10.1002/anie.202408947.

(7) Sheldrick, G. M. A short history of SHELX. *Acta Cryst. A* **2008**, *64* (1), 112–122. DOI: 10.1107/S0108767307043930.

(8) Sheldrick, G. M. Crystal structure refinement with SHELXL. *Acta Cryst. C* **2015**, *71* (1), 3–8. DOI: 10.1107/S2053229614024218.

(9) Sheldrick, G. M. SHELXT – Integrated space-group and crystal-structure determination. *Acta Cryst. A* **2015**, *71* (1), 3–8. DOI: 10.1107/S2053273314026370.

(10) Thorn, A.; Dittrich, B.; Sheldrick, G. M. Enhanced rigid-bond restraints. *Acta Cryst. A* **2012**, *68* (4), 448–451. DOI: 10.1107/S0108767312014535.

(11) Chen, L.; Ren, P.; Carrow, B. P. Tri(1-adamantyl)phosphine: Expanding the Boundary of Electron-Releasing Character Available to Organophosphorus Compounds. *J. Am. Chem. Soc.* **2016**, *138* (20), 6392–6395. DOI: 10.1021/jacs.6b03215. Published Online: May. 17, 2016.

(12) Cui, W.; Loeppky, R. N. The synthesis of N -arylcyclopropylamines via palladium-catalyzed C–N bond formation. *Tetrahedron* **2001**, *57* (15), 2953–2956. DOI: 10.1016/S0040-4020(01)00118-1.

(13) Tassone, J. P.; MacQueen, P. M.; Lavoie, C. M.; Ferguson, M. J.; McDonald, R.; Stradiotto, M. Nickel-Catalyzed N -Arylation of Cyclopropylamine and Related Ammonium Salts with (Hetero)aryl (Pseudo)halides at Room Temperature. *ACS Catal.* **2017**, *7* (9), 6048–6059. DOI: 10.1021/acscatal.7b02014.

(14) Maity, S.; Zhu, M.; Shinabery, R. S.; Zheng, N. Intermolecular 3+2 cycloaddition of cyclopropylamines with olefins by visible-light photocatalysis. *Angew. Chem., Int. Ed.* **2012**, *51* (1), 222–226. DOI: 10.1002/anie.201106162. Published Online: Nov. 23, 2011.

(15) Roscales, S.; Csákÿ, A. G. Synthesis of Mono-N-Methyl Aromatic Amines from Nitroso Compounds and Methylboronic Acid. *ACS omega* **2019**, *4* (9), 13943–13953. DOI: 10.1021/acsomega.9b01608. Published Online: Aug. 12, 2019.

(16) Luo, H.; Wang, G.; Feng, Y.; Zheng, W.; Kong, L.; Ma, Y.; Matsunaga, S.; Lin, L. Photoinduced Nickel‐Catalyzed Carbon‐Heteroatom Coupling**. *Chem. Eur. J.* **2023**, *29* (1), e202202385. DOI: 10.1002/chem.202202385.

(17) Arava, V.; Bandatmakuru, S. An Efficient Synthesis of N-Cyclopropylanilines by a Smiles Rearrangement. *Synthesis* **2013**, *45* (08), 1039–1044. DOI: 10.1055/s-0032-1318389.

(18) Rousseaux, S.; Liégault, B.; Fagnou, K. Palladium(0)-catalyzed cyclopropane C–H bond functionalization: synthesis of quinoline and tetrahydroquinoline derivatives. *Chem. Sci.* **2012**, *3* (1), 244–248. DOI: 10.1039/C1SC00458A.

(19) Gildner, P. G.; DeAngelis, A.; Colacot, T. J. Palladium-Catalyzed N-Arylation of Cyclopropylamines. *Org. Lett.* **2016**, *18* (6), 1442–1445. DOI: 10.1021/acs.orglett.6b00377. Published Online: Mar. 2, 2016.

(20) Mollari, L.; Valle-Amores, M. A.; Martínez-Gualda, A. M.; Marzo, L.; Fraile, A.; Aleman, J. Asymmetric synthesis of cyclic β-amino carbonyl derivatives by a formal 3 + 2 photocycloaddition. *Chem. Commun.* **2022**, *58* (9), 1334–1337. DOI: 10.1039/D1CC05867C. Published Online: Jan. 27, 2022.

(21) Nguyen, T. H.; Morris, S. A.; Zheng, N. Intermolecular 3+2 Annulation of Cyclopropylanilines with Alkynes, Enynes, and Diynes via Visible Light Photocatalysis. *Adv. Synth. Catal.* **2014**, *356* (13), 2831–2837. DOI: 10.1002/adsc.201400742.

(22) Hong, P.; Zhu, X.; Lai, X.; Gong, Z.; Huang, M.; Wan, Y. Room-Temperature CuI-Catalyzed N-Arylation of Cyclopropylamine. *J. Org. Chem.* **2024**, *89* (1), 57–67. DOI: 10.1021/acs.joc.3c01357. Published Online: Dec. 18, 2023.

(23) Wang, Q.; Xu, J.; Xu, Z.; Wang, Z.; Tao, X.; Ni, S.; Pan, Y.; Wang, Y. Catalyst-free electroreductive carboxylic acid–nitroarene coupling. *Green Chem.* **2023**, *25* (18), 7084–7091. DOI: 10.1039/D3GC02402D.

(24) Arava, V. R.; Bandatmakuru, S. R. Synthesis of N-substituted anilines via Smiles rearrangement. *Pharma. Chem.* **2013**, *5* (6), 12–27.

(25) Wang, J.; Zheng, N. The Cleavage of a C-C Bond in Cyclobutylanilines by Visible-Light Photoredox Catalysis: Development of a 4+2 Annulation Method. *Angew. Chem., Int. Ed.* **2015**, *54* (39), 11424–11427. DOI: 10.1002/anie.201504076. Published Online: Jul. 24, 2015.

(26) Zhu, Y.; Chen, S.; Zhou, Z.; He, Y.; Liu, Z.; Liu, Y.; Feng, Z. Iron/B2pin2 catalytic system enables the generation of alkyl radicals from inert alkyl C-O bonds for amine synthesis. *Chin. Chem. Lett.* **2024**, *35* (1), 108303. DOI: 10.1016/j.cclet.2023.108303.

(27) Saari, R.; Törmä, J.-C.; Nevalainen, T. Microwave-assisted synthesis of quinoline, isoquinoline, quinoxaline and quinazoline derivatives as CB2 receptor agonists. *Bioorg. Med. Chem.* **2011**, *19* (2), 939–950. DOI: 10.1016/j.bmc.2010.11.059. Published Online: Dec. 9, 2010.

(28) Jiang, D.; Fu, H.; Jiang, Y.; Zhao, Y. CuBr/rac-BINOL-catalyzed N-arylations of aliphatic amines at room temperature. *J. Org. Chem.* **2007**, *72* (2), 672–674. DOI: 10.1021/jo062060e.

(29) Abdel-Magid, A. F.; Carson, K. G.; Harris, B. D.; Maryanoff, C. A.; Shah, R. D. Reductive Amination of Aldehydes and Ketones with Sodium Triacetoxyborohydride. Studies on Direct and Indirect Reductive Amination Procedures(1). *J. Org. Chem.* **1996**, *61* (11), 3849–3862. DOI: 10.1021/jo960057x.
